# Self-Assembly of Stimuli-Responsive [2]Rotaxanes by Amidinium Exchange

**DOI:** 10.1021/jacs.1c05230

**Published:** 2021-09-24

**Authors:** Oleg Borodin, Yevhenii Shchukin, Craig C. Robertson, Stefan Richter, Max von Delius

**Affiliations:** † Institute of Organic Chemistry, Ulm University, Albert-Einstein-Allee 11, 89081 Ulm, Germany; ‡ Department of Chemistry, University of Sheffield, Brook Hill, Sheffield S3 7HF, U.K.

## Abstract

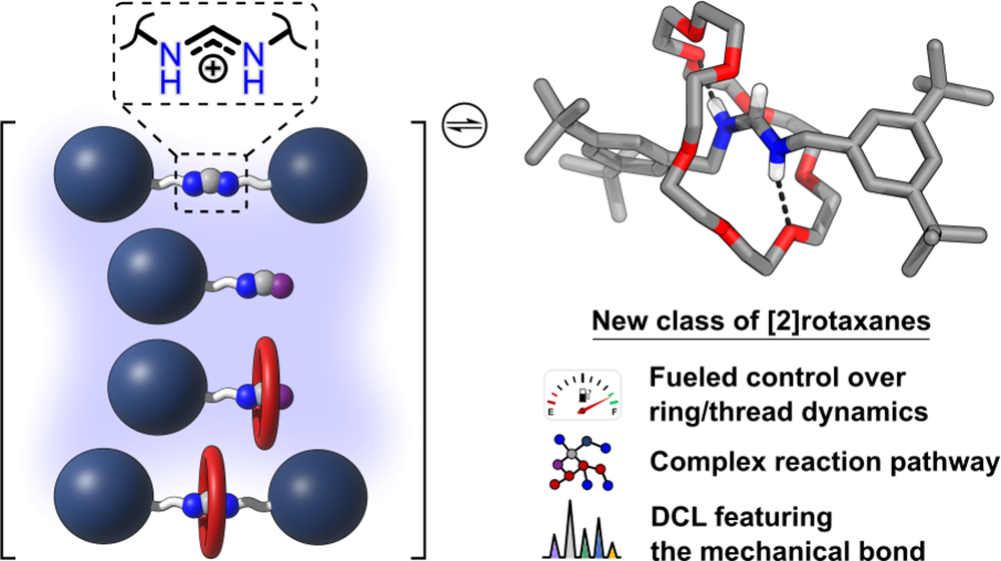

Advances in supramolecular chemistry are often underpinned by the development of fundamental building blocks and methods enabling their interconversion. In this work, we report the use of an underexplored dynamic covalent reaction for the synthesis of stimuli-responsive [2]rotaxanes. The formamidinium moiety lies at the heart of these mechanically interlocked architectures, because it enables both dynamic covalent exchange and the binding of simple crown ethers. We demonstrated that the rotaxane self-assembly follows a unique reaction pathway and that the complex interplay between crown ether and thread can be controlled in a transient fashion by addition of base and fuel acid. Dynamic combinatorial libraries, when exposed to diverse nucleophiles, revealed a profound stabilizing effect of the mechanical bond as well as intriguing reactivity differences between seemingly similar [2]rotaxanes.

## Introduction

Over the past two decades, [2]rotaxanes have found diverse uses, ranging from (stereo)selective synthesis and catalysis^1^ to molecular machines,^2^ the stabilization of reactive groups,^1c,2b,3^ sequence-specific peptide synthesis,^4^ supramolecular medicinal chemistry,^5^ materials chemistry,^6^ and optoelectronics.^3i,7^ Future progress on these frontiers will likely depend on the development of methods for the synthesis of new types of mechanically interlocked compounds.^8^ Recent examples to this end include a concave–convex π–π-template approach,^9^ the strategic use of covalent templates based on C–Si^10^ or C–O bonds,^11^ organometallic macrocycles,^3h^ a metal-free active template approach,^12^ and hydrogen bond and halogen bond assisted anion templation.^3j,13^

Dynamic covalent chemistry (DCvC)^14^ has been employed extensively for the preparation of [2]rotaxanes, [2]catenanes, and more complex mechanically interlocked architectures (MIAs).^3j,14e,15^ Arguably the most popular reversible organic reaction for the preparation of MIAs has been the condensation of aldehydes with primary amines that gives rise to imines.^15f,15h,15k,16^ This dynamic covalent reaction has however often been followed by a reduction step, giving rise to a classic pairing of rotaxane chemistry, namely the combination of a secondary ammonium ion thread with a crown ether-type ring.^17^

**Figure 1:**
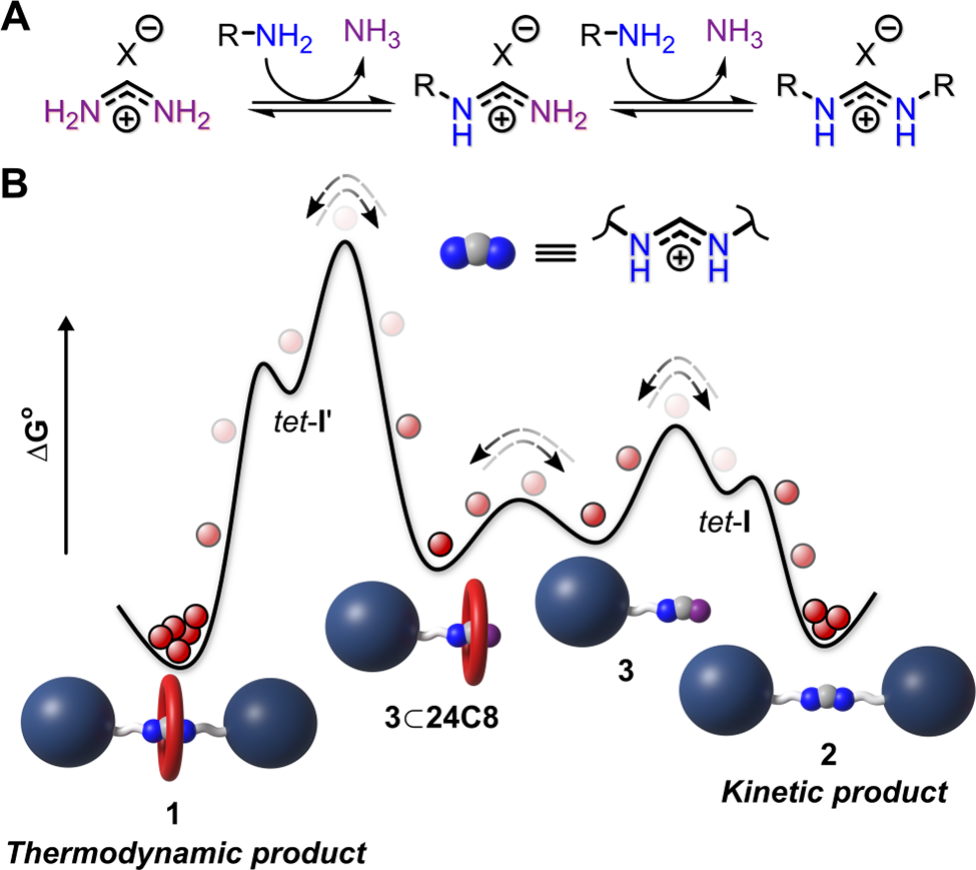
(A) General scheme for twofold amidinium exchange starting from formamidinium salts. (B) Qualitative free energy diagram for the self-assembly [2]rotaxanes by amidinium exchange. **24C8**: 24-crown-8. *tet*-**I**: tetrahedral intermediates. For the full reaction mechanism see Supporting Information, Scheme S16.

Herein we report the application of the dynamic covalent reaction amidinium exchange^18^ (Figure 1A) for the self-assembly of [2]rotaxanes, which exhibit many features of the established ammonium/crown ether systems. In contrast to previous dynamic covalent syntheses of rotaxanes,^15d−15f,15j,15k,16b,19^ the formamidinium moiety serves both as a binding site for the ring (e.g., 24-crown-8) and as a platform for dynamic covalent exchange (Figure 1B), which makes this synthesis approach comparatively minimalistic and endows the reaction products with unusual properties. For instance, the mechanical bond between the crown ether and the *N*,*N’*-disubstituted formamidinium ion renders the latter much less susceptible to nucleophilic attack and dramatically slows down the interconversion between the two geometrical isomers (*E*,*E* and *E*,*Z*). We explored these features in depth by coupling the amidinium/amidine acid–base equilibrium to the fuel acid trichloroacetic acid (TCA) and by coupling dynamic combinatorial libraries (DCL) comprising six rotaxanes with the irreversible action of *N*-nucleophiles that cause the disassembly of the rotaxanes.

## Results and Discussion

### Synthesis and Characterization of Formamidinium [2]Rotaxanes

**Figure 2:**
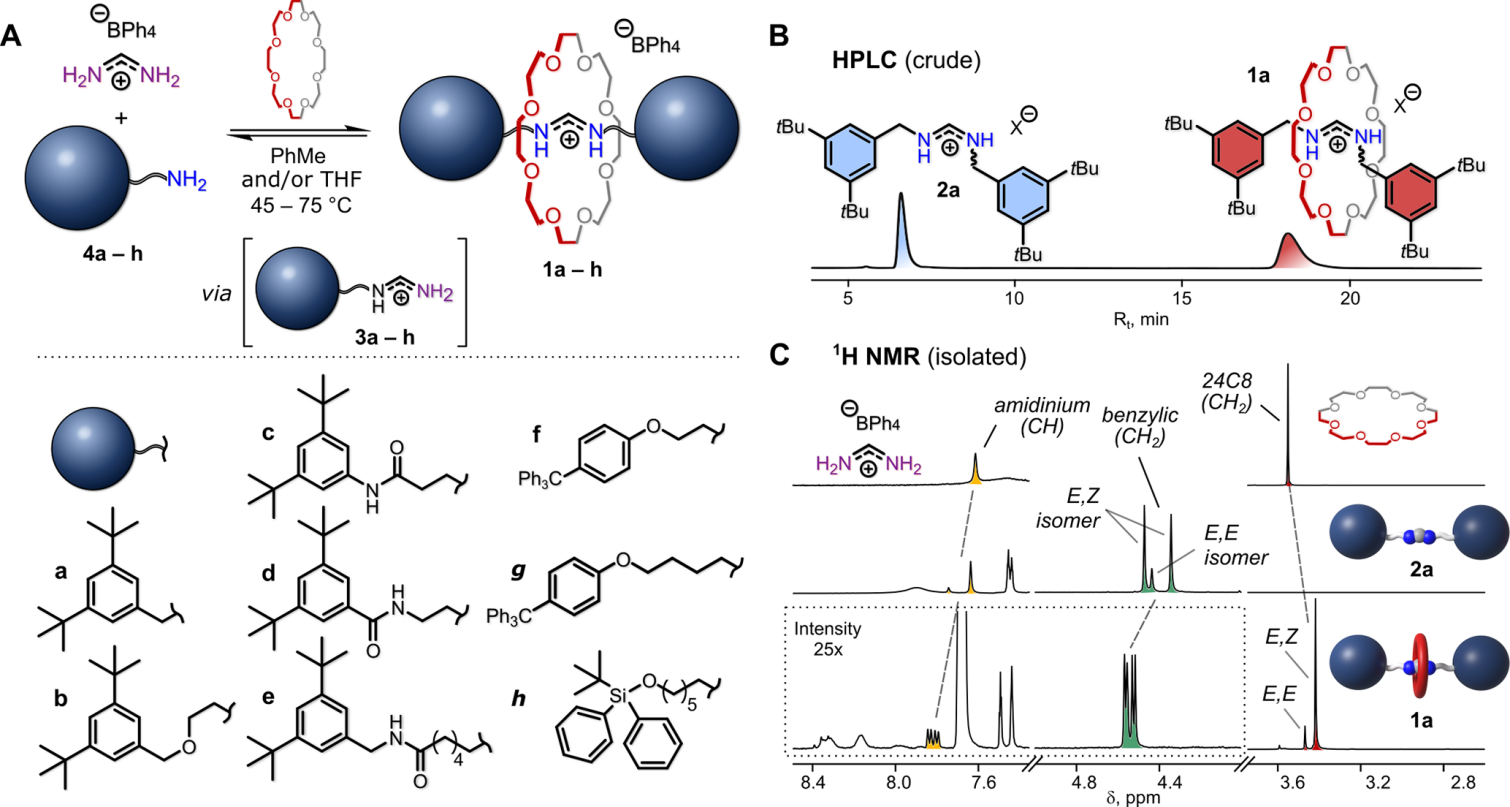
(A) Rotaxane self-assembly by amidinium exchange and scope of the reaction. Yields: **1a**, 36%^*a*^ (brsm: 50%); **1b**, 21%^*a*^; **1c**, 16%^*a*^ (as BArF salt); **1d**,15%^*a*^; **1e**, 15%^*a*^; **1f**, 20%^*a*^; **1g** 42%^*b*^; **1h**, 26%^*b*^; **1i** (2f27C9), 10%^*b*^; **1j** (2h⊂27C9), 12%^*b*^ (*a*, isolated yield; *b*, HPLC yield). (B) Representative HPLC chromatogram of a crude reaction mixture at equilibrium. (C) ^1^H NMR stack plot (400 MHz, CD_3_CN, 295 K) of starting materials (formamidinium tetraphenylborate and 24-crown-8), isolated thread **2a** (anion: BPh_4_^–^) and isolated rotaxane **1a** (anion: BArF^–^). Both **1a** and **2a** exist in solution as a mixture of *E*,*E*- and *E*,*Z*-isomers (characteristic peaks are indicated). For chemical structure of these isomers, see Figure 3A.

Inspired by the seminal work of Petitjean and co-workers,^18d^ we wondered if a crown ether could be coordinated during amidinium exchange, potentially affording a new type of [2]rotaxane. We also noticed recent work by Leigh and co-workers on a metal-free active template synthesis that is based on the reaction of primary amines with electrophiles inside the cavity of a crown ether.^12b,12c^ We therefore investigated the reaction between formamidinium tetraphenylborate (**FA·BPh**_**4**_) and primary amines such as 3,5-di-*tert*-butylbenzylamine (**4a**) in the presence of 24-crown-8 (**24C8**; see Figure 2A for a general scheme). Having observed the corresponding rotaxanes **1** by tandem mass spectrometry in exploratory experiments (Supporting Information, Section 5), we proceeded with an optimization of the synthesis of **1a** and decided to vary solvent, stoichiometry, reaction temperature, base, and nucleophilic catalysts (Supporting Information, Section 3). The reaction progress was monitored by LCMS (Figure 2B). The highest molar percentage of **1a** vs **2a** (61%) was achieved when formamidinium salt, amine **4a**, and **24C8** were combined in a molar ratio of 1:2:2 in toluene at 75 °C for 3 days (Table S6). At room temperature, the reaction in THF or MeCN afforded **1a** in similar amounts (molar percentage of **1a** vs **2a** > 40%), even though the reaction took 20–40 days in this case (Tables S1–S3).

Because amidinium exchange is a reversible reaction and rotaxane **1a** and thread **2a** are not the only components of the reaction mixture (*vide infra*), rotaxane yields (isolated or HPLC) were generally lower than the above-mentioned molar percentage of 61% and fell into the range 10%–42% for the eight investigated amine stoppers (**4a**–**h**, Figure 2A). Isolation of the reaction products was achieved either by semipreparative HPLC or by preparative TLC, and for rotaxane **1a** we demonstrated that starting material **4a** could be recovered (yield based on recovered starting material: 50%). When the larger crown ether **27C9** was used, lower yields were observed, presumably due to weaker binding of **27C9** to the amidinium template (Supporting Information, Section 4). All isolated rotaxanes (**1a**–**f**) were fully characterized by ^1^H and ^13^C NMR spectroscopy (Supporting Information, Section 4; Figures S99–S110), and their mechanically interlocked nature was confirmed by tandem mass spectrometry (Supporting Information, Section 5). The ^1^H NMR spectrum of **1a** (Figure 2C), as a representative example of the amidinium rotaxanes, features benzylic CH_2_ signals and amidinium CH signals that are shifted downfield, compared to the corresponding signals of thread **2a**, which is indicative of interactions of those protons with the crown ether. Moreover, the amidinium CH signal in **1a** (*E*,*Z* isomer, *vide infra*) appears as a doublet-of-doublets, indicating that the exchange of amidinium NH protons is slow on the NMR time scale due to hydrogen bonding with the macrocycle.

### Formamidinium *E*,*E* and *E*,*Z* Isomers: Effect of the Mechanical Bond in Solution and the Solid State

In solution, both **1a** and **2a** exist as a mixture of *E,Z*- and *E,E*-isomers, as can be clearly seen from the ^1^H NMR spectra (Figure 2C; see Supporting Information, Section 8 for NMR studies of the isomerization equilibria). In solution and for the case of weakly coordinating counterions (e.g., PF_6_^–^ or BArF^–^), amidinium ions adopt mainly the *E*,*Z*-configuration (Supporting Information, Sections 8.1 and 8.2) and this rule applies to both rotaxanes **1** and threads **2**. However, there is a striking difference between isomerization rates between the threads and their mechanically interlocked analogues: while noninterlocked amidinium ions undergo relatively fast *E,E*/*E,Z* isomerization, with a rate constant about 1.3 s^–1^ (Figures S51–S53; Table S23) at room temperature, isomerization in the amidinium rotaxanes is extremely slow: 0.006 s^–1^ at room temperature (Figures S51, S54, S56; Table S23).

The binding of shape-complementary anions is known to affect the *E,E*/*E,Z* equilibrium. For instance, it has been established that amidines strongly bind carboxylic acids (*K*_a_ ≈ 10^8^ M^–1^ in CDCl_3_)^18d,20^ and, in the case of *N*,*N’*-disubstituted amidines, form rigid salt bridges with well-defined *E,E* geometry.^18d^ This property of amidines has been widely used in the field of supramolecular chemistry and materials science to construct sophisticated molecular architectures.^21^ In agreement with this precedence, we found that *N*,*N′*-disubstituted amidinium ions (e.g., threads **2**) with weakly coordinating anions exhibit mainly *E,Z* geometry in solution and that addition of carboxylates led to immediate and full switching to the *E,E* geometry and formation of the salt bridge (Scheme S20, Figure S58). In stark contrast, addition of a carboxylate to a solution of rotaxane **1a** did not induce any significant shift from the *E,Z* to the *E,E* isomer, even though ^1^H NMR spectroscopy indicated unspecific binding between carboxylate and **1a** (Scheme S21, Figure S59). This unusual behavior of mechanically interlocked amidinium ions indicates that adopting the *E,E* geometry is highly unfavorable in the presence of a surrounding crown ether.

**Figure 3:**
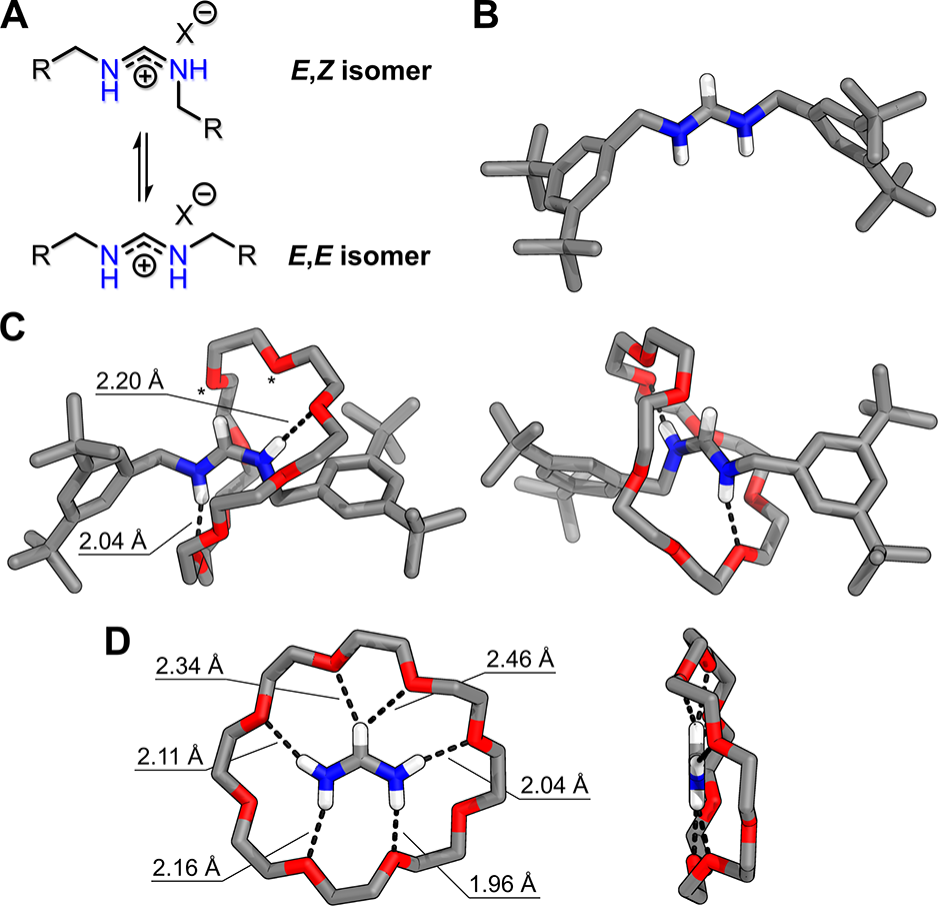
(A) Structure of the formamidinium *E*,*Z* and *E*,*E* isomers. (B) Crystal structure of thread **2a** (anion: BPh_4_^–^). (C) Crystal structure of rotaxane **1a** (anion: BArF^–^); two different views. In the left structure, two oxygen atoms that form weak hydrogen bonds C–H···O (2.47 and 2.76 Å) with the amidinium moiety are marked with asterisks. (D) Crystal structure of a hydrogen-bonded complex between formamidinium and **24C8** (anion: BPh_4_^–^); two different views. Counterions and nonamidinium hydrogen atoms are omitted for clarity. Dotted lines represent hydrogen bonds.

In the solid state, literature reports suggest that *N*,*N′*-disubstituted formamidinium ions adopt an *E,E*-configuration (Figure 3A).^22^ Therefore, we were curious whether the mechanically interlocked crown ether also alters this fundamental structural property of the formamidinium ion. To our delight, we were able to obtain single crystals of both thread **2a** (Figure 3B; BPh_4_^–^ salt) and the corresponding rotaxane **1a** (Figure 3C, BArF^–^ salt), and investigate their structures by X-ray diffraction. As expected, thread **2a** crystallized predominantly as the *E*,*E* isomer (Supporting Information, Section 11). However, rotaxane **1a** crystallized exclusively in the *E*,*Z*-configuration, which confirmed our previous conclusion about the high instability of the *E,E*-isomer inside the cavity of the crown ether ring. In **1a**, the amidinium moiety forms two N–H···O hydrogen bonds with the crown ether (H···O bond lengths are 2.04 and 2.20 Å). It is worth mentioning that the amidinium C–H group also forms a weak bifurcated C–H···O hydrogen bond with the crown ether (H···O bond lengths are 2.47 Å and 2.76 Å). This bifurcated hydrogen bond is, however, much longer (on average) than the corresponding bifurcated C–H···O hydrogen bonds in a complex between our formamidinium (**FA**) starting material and **24C8** (H···O bond lengths are 2.34 Å and 2.46 Å), where **FA** can perfectly fit inside the cavity of **24C8** and form in total five hydrogen bonds (including the three-centered one) with the crown ether (Figure 3D).

### Controlling Rotaxane Thermodynamics and Kinetics by Addition of a Chemical Fuel

We reasoned that deprotonation of the amidinium moiety in our rotaxanes would essentially shut down binding of the crown ether ring to the thread, which would be analogous to rotaxanes based on secondary ammonium ions. Indeed, upon addition of strong base NBu_4_OH to rotaxane **1a**, we observed by ^1^H NMR spectroscopy that benzylic, *tert*-butyl, and crown ether signals turned into broad singlets, indicative of relatively fast exchange between possible configurational and (co)conformational isomers on the NMR time scale (Schemes S17 and S22, Figure S60). Given the difficulty of deprotonation of ammonium-based rotaxanes,^23^ the interconversion between the isomers might be further facilitated by the tautomeric equilibria induced by residual amounts of the base (HO^–^).^24^ As expected, addition of acetic acid (1 equiv) restored the sharp signals belonging to the original *E,Z*- and *E,E*-isomers, which due to the mechanical bond undergo slow exchange (*vide supra*).

The rotaxanes reported herein therefore exist in two states:

(i) The protonated amidinium state, where the ring is associated with the thread (*K*_a_ ca. 30 M^–1^ in CD_3_CN was determined for a comparable pseudorotaxane; Supporting Information, Section 6.3) and *E,Z*/*E,E* isomerization is slow.
(ii) The deprotonated state of the thread, where binding between amidine and crown ether is negligible (*K*_a_ < 1 M^–1^ in CD_3_CN was estimated by host–guest titration with a pseudorotaxane; Supporting Information, Section 6.4) and configurational (*E*/*Z*) and/or (co)conformational dynamics are much faster compared to the protonated state.

While acid–base control of binding is a common feature of [2]rotaxanes, not least those based on secondary ammonium ions, acid–base control of thread isomerization is underexplored, because most of the studies of rotaxanes focus on either ring shuttling or pirouetting motions.^1c,25^ The new rotaxane motif described herein therefore exhibits a feature that has made the crown ether/ammonium couple very popular especially for the design of molecular machines,^26^ while also offering something different.

**Figure 4:**
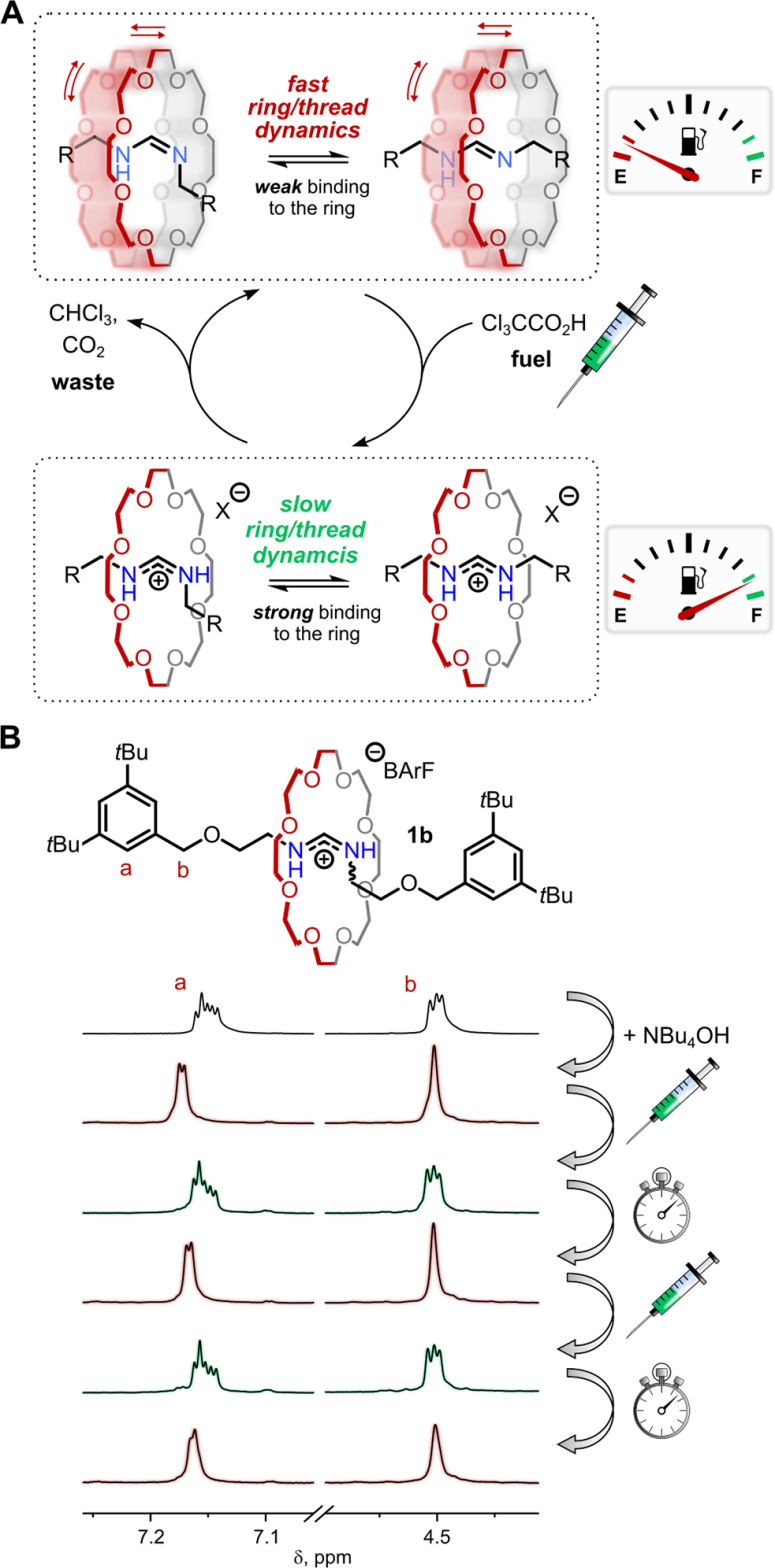
(A) Transient switching between two states of the amidinium rotaxane: (1) deprotonated rotaxane with fast geometry switching and (2) protonated rotaxane with slow *E*/*Z* isomerization due to hydrogen bonding to the crown ether ring. (B) Changes in ^1^H NMR spectrum (400 MHz, CD_2_Cl_2_, 295 K) of rotaxane **1b** (6 mM) upon deprotonation and subsequent addition of chemical fuel −Cl_3_CCO_2_H (0.22 M solution in CD_2_Cl_2_, 1.0 equiv). Waiting time after addition of the fuel: 1 h at 40 °C. Top spectrum corresponds to the initial protonated form of **1b** (anion: BArF^–^).

Inspired by recent reports from the groups of Takata, Di Stefano, Leigh, and Schmittel,^27^ we decided to investigate whether a chemical fuel—trichloroacetic acid (TCA)—could be used to generate the strongly binding protonated state in a transient fashion. According to this reasoning, the amidine rotaxane, where the interconversion between configurational and (co)conformational isomers is fast, would be protonated by TCA, thus bringing the rotaxane into the state where interconversion between *E,Z*- and *E,E*-isomers is slowed by the ring. The trichloroacetate anion would then gradually decompose into CO_2_ and CHCl_3_, while the rotaxane is deprotonated into the original state (Figure 4A). In a proof-of-concept experiment, we added TCA to deprotonated rotaxane **1b** and observed in the ^1^H NMR spectrum the expected splitting of broad singlets corresponding to benzylic, aromatic, and *tert*-butyl protons (Figure 4B; Scheme S23, Figures S61, S62). After 1 h at 40 °C, the original broad singlets were restored, indicating that the rotaxane was again in the deprotonated form. This cycle could be repeated at least three times, indicating that the underlying chemistry is sufficiently robust to find uses in molecular machines.

### Complexity of the Reaction Pathway toward the Amidinium [2]Rotaxanes

While optimizing the reaction conditions of the rotaxane synthesis by the amidinium exchange, we were initially puzzled by the observation that rotaxane yields did not exceed 30–40% despite our best optimization efforts. Careful analysis of the reaction pathway, including host–guest titrations of key intermediate species, helped to shed light on our difficulties to obtain yields greater than 40%.

During the rotaxane self-assembly, **24C8** strongly binds to the unsubstituted formamidinium ion (*K*_a_ = 9.5 × 10^3^ M^–1^ in CD_3_CN at 295 K; Supporting Information, Section 6.1; crystal structure, Figure 3D) and less strongly to the key reaction intermediate–half-thread **3** (*K*_a_ ≈ 10^2^ M^–1^ in CD_3_CN at 295 K; Supporting Information, Section 6.2; for general structure, see Figure 2A). This leads to the decreased reactivity of the amidinium moiety toward amines, since **24C8** sterically hinders the electrophilic reaction center on the amidinium moiety and presumably also reduces its electrophilicity due to hydrogen bonding. As a consequence, the free thread forms much faster than the rotaxane. However, the reversibility of amidinium exchange eventually leads to rotaxane formation, since *N*,*N’*-disubstituted amidinium ions exhibit (weak) binding to **24C8** (*K*_a_ ca. 30 M^–1^ in CD_3_CN at 295 K; Supporting Information, Section 6.3), thus making the rotaxane a thermodynamic product.

**Figure 5:**
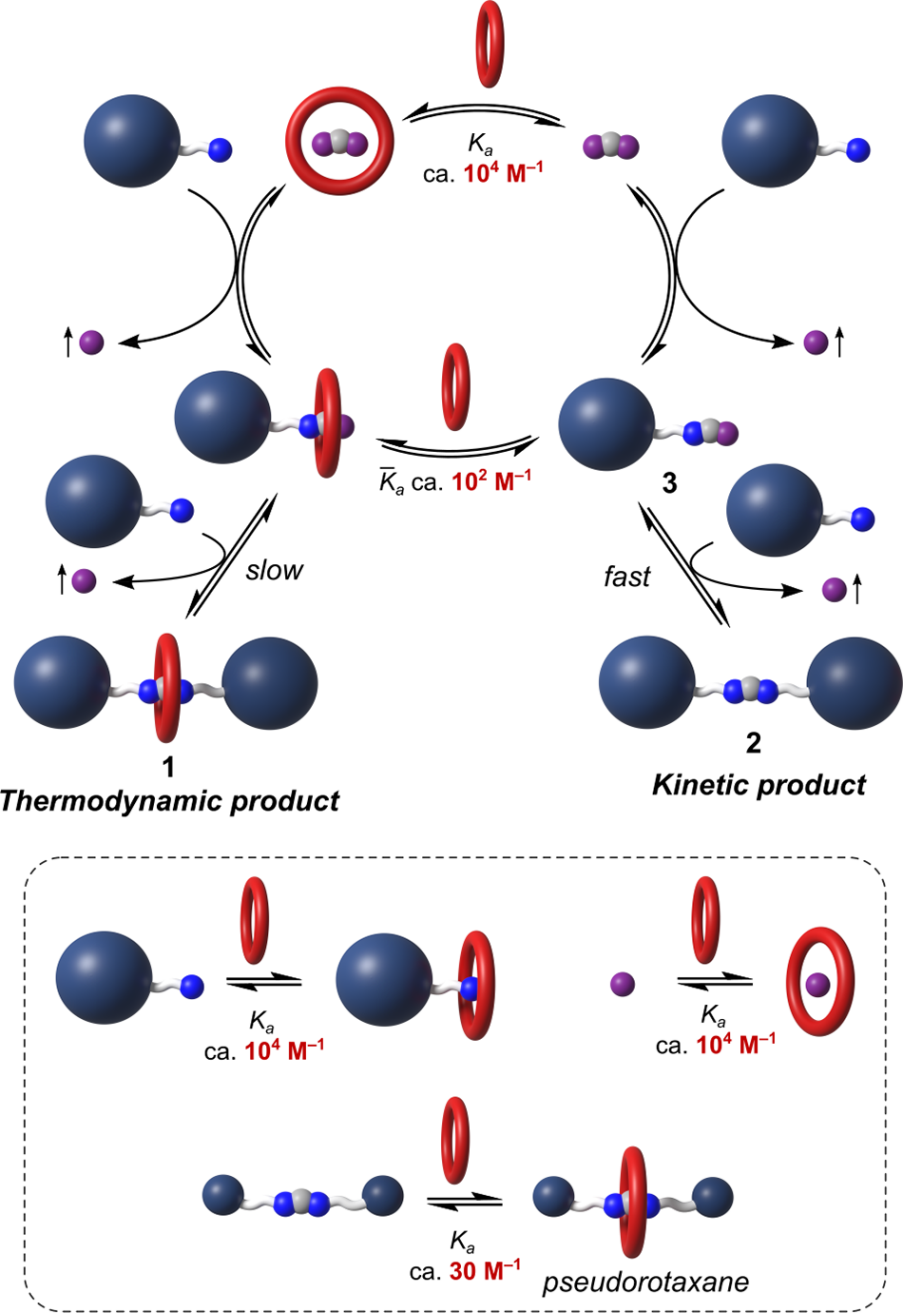
Proposed reaction pathway toward the amidinium rotaxanes, and overview on relevant host–guest equilibria. Acid–base equilibria are omitted for clarity. Complexation between **24C8** and nitrogen containing species (NH_3_, primary amines, amidines) must mainly occur while nitrogen atoms are protonated. Association constants (*K*_a_) for complexes NH_4_^+^⊂**24C8**, **FA**⊂**24C8**, and **3a**⊂**24C8** were determined in CD_3_CN by host–guest titrations (Supporting Information, section 6). Association constant for a complex between protonated primary amine and **24C8** was estimated as an average between *K*_a_ for NH_4_^+^⊂**24C8** and for the reported complex between a secondary ammonium ion and **24C8**.^30^

Even though the reasoning from the last paragraph might explain the slow rate of rotaxane formation, it does *not* explain why the amidinium rotaxanes cannot be formed in higher yields. To understand this issue, we thought that perhaps all chemical transformations that occur during self-assembly need to be considered (Figure 5). Conjugate acids of both primary amine starting materials (**4**) and NH_3_ as a byproduct of the reaction are more effective at binding **24C8** than amidinium species (Supporting Information, Section 6.5; Figure S33).^28^ Moreover, acid–base equilibria between different nitrogen-containing species might affect not only the outcome of the host–guest equilibria but also the kinetics of amidinium exchange (considering that amidinium exchange is fast only when amidines are protonated). Overall, the reaction network comprises more than 15 different equilibria (dynamic covalent, host–guest, acid–base, and *E,Z*/*E,E*), which is unusually complex for a rotaxane synthesis and puts the moderate observed yields into a different perspective.

To better understand the reaction pathway toward amidinium rotaxanes, we performed a series of experiments starting with an investigation of amidinium substrates other than **FA·BPh**_**4**_ (formamidinium acetate (**FA·OAc**), *N*,*N’*-diphenylformamidinium tetrafluoroborate, and tetrakis[3,5-bis(trifluoromethyl)phenyl]borate (**DPFA·BF**_**4**_ and **DPFA·BArF**), *N*,*N’*-dimethylformamidinium tetraphenylborate (**DMFA·BPh**_**4**_), and *N*,*N′*-dibenzylformamidinium tetraphenylborate (**S1**)). Under standard conditions, rotaxane **1a** formed either in trace amounts (in the case of **FA·OAc** and **DPFA·BF**_**4**_) or in very low yield (in the case of **DMFA·BPh**_**4**_, **DPFA·BArF**, and **S1**) (Supporting Information, Sections 7.3.1–7.3.4). The amidinium exchange reaction affording noninterlocked products still proceeded smoothly from all four starting materials. In the case of **DMFA·BPh**_**4**_ and **FA·OAc**, we also tested the possibility to approach the same equilibria starting either from rotaxane **1a** and MeNH_2_ or from the evolving reaction mixture and an acetate salt, respectively (Table S18 and Figure S49). In both cases, we obtained near-identical equilibrium mixtures, indicating that these systems are under thermodynamic control.

We also found that release of NH_3_ has positive, yet not an essential effect on the yield of rotaxane formation (Table S14): reactions where NH_3_ was released by regularly opening the reaction vessel afforded higher rotaxane yields (by ∼50%) than reactions where the vessel was kept tightly closed during the course of the reaction.

The evidence described above suggests the following conclusions: (i) the rotaxane synthesis by amidinium exchange is mostly governed by thermodynamics; (ii) a successful rotaxane synthesis requires a weakly coordinating counterion, otherwise one of the thermodynamic driving forces (i.e., binding of **24C8** to the thread) becomes too small; (iii) loss of NH_3_ represents an irreversible driving force for the forward rotaxane synthesis, but at the same time complete loss of NH_3_ is an obstacle for conversion of the thread to the rotaxane at the end of the reaction. There is a close analogy here to the disulfide chemistry pioneered by Sanders and Otto.^29^ These DCLs start from dithiols and are dynamic for a very long time until all thiols are oxidized by air to disulfides, when dynamic exchange comes to a halt.

To investigate whether the third conclusion could be leveraged to increase rotaxane yields, we decided to add ammonia to the crude self-assembly products, to test if the thread would further convert into the rotaxane. Addition of NH_3_ (0.5 M solution in THF) to the crude reaction mixture, however, led only to insignificant changes of the rotaxane amount (Figures S40, S42) suggesting that the global equilibrium in the reaction system was shifted not only toward **1a** but also toward other species (half-thread **3a** and amine **4a**). The rotaxane yield could however be significantly improved (up to 47%), when we used an ammonia surrogate—(Me_3_Si)_2_NH, which is known to slowly hydrolyze to NH_3_*in situ* (Figure S43). The prospect to improve rotaxane yields by addition of (Me_3_Si)_2_NH will be investigated systematically in future studies.

**Figure 6:**
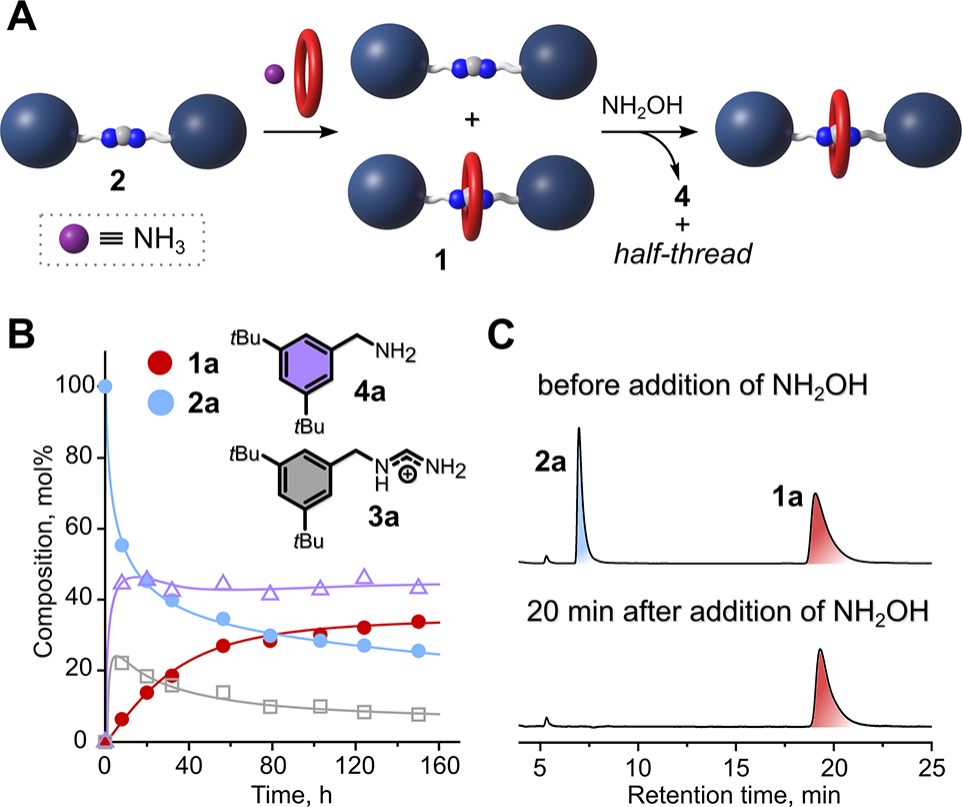
(A) Schematic representation of the conversion of the thread into the rotaxane and further selective degradation of the remaining thread. (B) HPLC monitoring of the conversion of thread **2a** into rotaxane **1a**. Reaction conditions: 1.0 equiv of **2a** (0.2 M), 2.6 equiv of **24C8**, 2.0 equiv of NH_3_; solvent = PhMe/THF (4:6 v/v); ∼65 °C. The lines are used to guide the eye. (C) HPLC chromatograms of the synthesis of **1a** before and after addition of NH_2_OH (50% in H_2_O, 1.25 eq. with respect to **FA·BPh**_**4**_, room temperature).

We wondered if the rotaxane formation was possible starting from thread, crown ether, and NH_3_. If so, this would provide solid evidence for the reversibility of all steps in the reaction pathway. We carried out the reaction between thread **2a**, **24C8** (2.6 equiv) and NH_3_ (2.0 equiv) and monitored the reaction progress by HPLC (Figure 6A and 6B, Figure S41). We found that rotaxane **1a** did gradually form and the equilibrium was reached after 6 days. Interestingly, the amount of amine **4a** reached a steady state after a few hours, while the amounts of thread **2a** and half-thread **3a** gradually decreased. Overall, the evolution of the reaction was very similar to that of a rotaxane synthesis starting from **FA·BPh**_**4**_, amine **4a**, and **24C8** (Figure S40). Unexpectedly, the conversion of the thread to the rotaxane was also possible in the presence of **24C8** and amine **4a**, hence without addition of NH_3_ (Table S20, entries 1 and 3; Scheme S13). This result indicates that the proposed reaction pathway (Figure 1B and Figure 5) is not the only possible one (Scheme S16), and covalent capture of the ring may occur as a result of precoordination of the ring to the amine^12b^ or the thread.

Taking all the aforementioned evidence together, we conclude that the self-assembly of formamidinium rotaxanes can not be attributed to a passive template or to a metal-free active template approach,^12b,12c^ even though it possesses features of both. Most notably, during our amidinium rotaxane self-assembly, the crown ether ring *inhibits* the thread forming reaction, while the rotaxane can form only due to dynamic covalent exchange (Figure S50). The phenomenon that a rotaxane synthesis succeeds despite the ring component inhibiting covalent capture is rather rare and has been reported by the groups of Vögtle and Schalley.^31^

### Dynamic Combinatorial Libraries of Amidinium [2]Rotaxanes

In the rotaxanes reported herein, the amidinium moiety serves both as a binding site for the ring and as a platform for dynamic covalent exchange. This unusual architecture allows the macrocycle to affect not only supramolecular properties (e.g., amidinium anion binding) but also the kinetics of amidinium exchange (Supporting Information, Section 9.1).

A remarkable example of such a kinetic effect was observed when a crude reaction mixture after rotaxane synthesis was allowed to react with *N*-nucleophiles. Addition of strong nucleophile hydroxylamine (NH_2_OH), to such a mixture of thread **2a** and rotaxane **1a**, led to complete degradation of the thread, while leaving the rotaxane intact (Figure 6A and 6C). Significant degradation of the rotaxane was only observed after prolonged reaction times (Figure S40), confirming that the crown ether ring hinders the reactive electrophilic carbon of the amidinium moiety and further supporting the mechanistic regime described above. It is worth mentioning that selective degradation of the amidinium thread in the presence of the corresponding rotaxane also facilitated chromatographic purification of the latter, since both amidinium species are highly polar and have similar retention on a stationary phase (especially on normal phase, i.e. silica gel).

**Figure 7:**
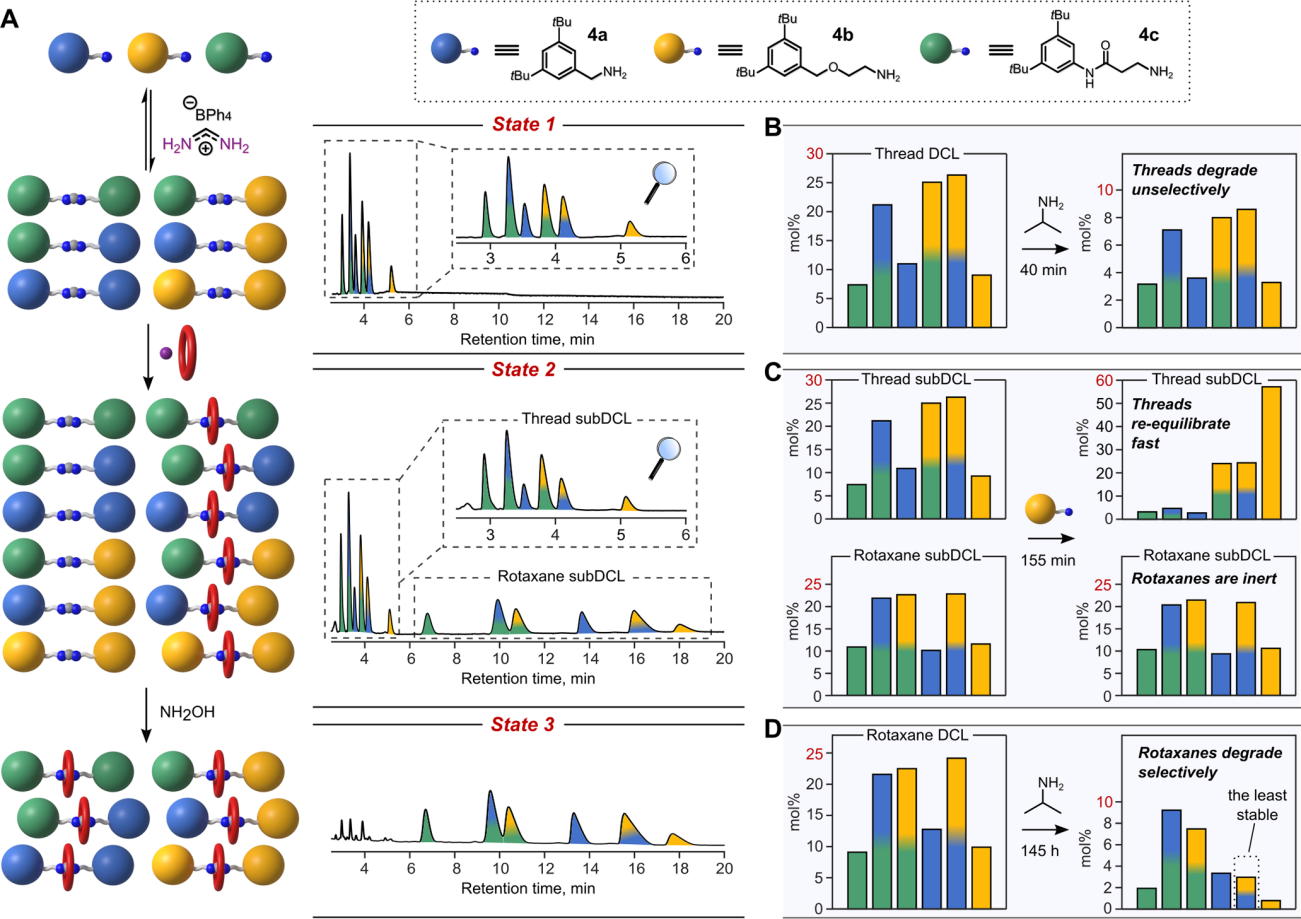
Synthesis and selective degradation of a dynamic combinatorial library (DCL) of amidinium rotaxanes. (A) Cartoon representation of the performed chemical transformations and corresponding HPLC chromatograms of the crude reaction mixtures. Reaction conditions: 1st step = 37 μmol of **FA·BPh**_**4**_, 25 μmol of each amine, 500 μL of THF, 60 °C, 2 h; 2nd step = 74 μmol of **24C8**, 56 μmol of NH_3_ (0.5 M in THF), 70 μL of PhMe, 70 °C, 100 h; 3rd step = 37 μmol NH_2_OH (50% in H_2_O), rt, 10 min. (B–D) Bar graphs showing changes in the distribution of the DCL members after subjecting each DCL state to an external chemical stimulus, i.e., *N*-nucleophiles (for details, see Supporting Information, Section 9.2). For these experiments, amidinium DCLs were first isolated by semipreparative HPLC and used as formate salts. In panel C, a roughly equimolar mixture of two subDCLs was used; molar percentages of the library members were calculated separately for each subDCL.

To the best of our knowledge, DCLs of MIMs, where the dynamic covalent moiety responsible for forming the thread, while also acting as binding site for the ring, have not been reported to date. We therefore proceeded with an exploration of dynamic combinatorial libraries (DCL) featuring formamidinium-based MIMs (Supporting Information, Section 9.2). In a proof-of-concept study, we used three different primary amines—**4a**, **4b**, **4c**—to prepare a DCL comprising six amidinium threads (Figure 7A, State 1). This DCL was converted into a larger DCL consisting of two sublibraries—one subDCL of threads and one subDCL of rotaxanes (Figure 7A, State 2; Figures S67, S68)—and it turned out that the two subDCLs have completely different kinetic properties. The “thread subDCL” can further “evolve” by interacting with newly introduced chemical species, while the “rotaxane subDCL” is essentially a silent observer. For instance, addition of amine **4b** led to upregulation of thread **2b** and downregulation of the other threads (Figure 7C, Figure S71). At the same time, the composition of the rotaxane subDCL remained unchanged. In another experiment, addition of NH_2_OH led to fast degradation of the thread subDCL, while all the members of the rotaxane subDCL remained intact (Figure 7A, State 3).^32^

The obtained rotaxane DCL was subsequently subjected to reactions with bulky nucleophiles (e.g., isopropylamine or *tert*-butylamine) over prolonged time. Even though each rotaxane degraded by at least 50%, to our surprise, some DCL members demonstrated much higher kinetic stability (e.g., blue-green rotaxane) than others (e.g., blue-yellow rotaxane) (Figure 7D, Figures S69, S70). When considering the differences in reactivity between rotaxanes, interestingly, we did not find a correlation with the space that is available for the crown ether along the axle. Interestingly, a similar DCL of the threads (State 1) did not reveal any selectivity toward reaction with an external, bulky nucleophile (Figure 7B, Figure S69), confirming that that the mechanical bond impacts not only the thermodynamic stability of the DCL members but also their (kinetic) reactivity.

## Conclusions

In conclusion, we describe a new approach for the dynamic self-assembly of [2]rotaxanes, which is based on an underexplored dynamic covalent reaction: amidinium exchange. The reaction appears to be relatively general and affords a wide range of symmetrical [2]rotaxanes in acceptable yields (up to 50% brsm yield) that may be further improved based on preliminary work using NH_3_ surrogate (Me_3_Si)_2_NH. Paradoxically, the dynamic covalent nature of the reaction facilitates and prevents the rotaxane formation at the same time. On the one hand, the crown ether ring kinetically impedes the nucleophilic attack of the amidinium electrophilic carbon by a primary amine, thus turning the product forming step into the slowest step of the entire reaction network. On the other hand, it is the reversibility of amidinium exchange, which allows the whole reaction system to converge toward MIMs as the dominant products, even when the reaction is started from the isolated thread.

Amidinium rotaxanes represent an extreme case of the mechanical bond affecting fundamental kinetics and thermodynamics of a functional group.^1c,2b,3a−3g,33^ The rotaxanes described herein not only are surprisingly stable toward hydrolysis and the reaction with other nucleophiles but they also exhibit unusual *E,E*/*E,Z* isomer ratios and the interconversion between these isomers is significantly slowed down due to the presence of the interlocked crown ether. Binding of the ring can be controlled by addition of acid/base, and by using a fuel acid this process was successfully carried out in a transient manner. Overall, formamidinium/**24C8** rotaxanes represent a dynamic covalent equivalent to the well-established secondary-amine/**24C8** rotaxanes, which is why we expect uses in molecular machinery, “smart” materials, and systems chemistry.

## Supporting Information Available

General experimental procedures, synthesis and characterization data, MS/MS data, host–guest titrations, mechanistic studies, *E*,*E*/*E*,*Z* isomerization studies, crystallographic data (PDF)

## Accession Codes

CCDC 2076127–2076129 contain the supplementary crystallographic data for this paper. These data can be obtained free of charge via www.ccdc.cam.ac.uk/data_request/cif, or by emailing data_request@ccdc.cam.ac.uk, or by contacting The Cambridge Crystallographic Data Centre, 12 Union Road, Cambridge CB2 1EZ, UK; fax: +44 1223 336033.

## Abbreviations

BArF: tetrakis[3,5-bis(trifluoromethyl)phenyl]borate
DCL: dynamic combinatorial library
DCvC: dynamic covalent chemistry
EXSY: exchange spectroscopy
HPLC: high-performance liquid chromatography
MeCN: acetonitrile
MIA: mechanically interlocked architecture
MIM: mechanically interlocked molecule
NMR: nuclear magnetic resonance
PhMe: toluene
TCA: trichloroacetic acid
THF: tetrahydrofuran
TLC: thin layer chromatography
24C8: 24-crown-8
27C9: 27-crown-9
