# Supplementary material for: Self-Assembly of Stimuli-Responsive [2]Rotaxanes by Amidinium Exchange
Source: J Am Chem Soc. 2021 Sep 24;143(40):16448–57. doi: 10.1021/jacs.1c05230 (PMC8517971; doi:10.1021/jacs.1c05230)
Supplement: Supplementary file 1 — ja1c05230_si_001.pdf [file ja1c05230_si_001.pdf]

Supporting Information

Self-assembly of stimuli-responsive [2]rotaxanes by amidinium exchange

Oleg Borodin,[a] Yevhenii Shchukin,[a] Craig C. Robertson,[b] Stefan Richter[a] and Max von Delius[a*]

a) Institute of Organic Chemistry, Ulm University, Albert-Einstein-Allee 11, 89081 Ulm, Germany.

b) Department of Chemistry, University of Sheffield, Brook Hill, Sheffield, S3 7HF, U.K.

E-Mail: max.vondelius@uni-ulm.de

Contents

1. General methods and abbreviations 3

2. Synthesis of building blocks for the amidinium [2]rotaxanes 4

 2.1 Synthesis of substrates for amidinium exchange 4

 2.2 Synthesis of crown ethers 5

 2.3 Synthesis of primary amines 6

 2.4 Synthesis of threads for amidinium (pseudo/semi)rotaxanes 15

3. Optimization of the self-assembly of amidinium [2]rotaxanes 18

4. Synthesis of the amidinium [2]rotaxanes 21

5. Tandem mass spectra of the amidinium [2]rotaxanes 32

6. Binding studies: NMR titrations 36

 6.1 Binding of 24-crown-8 to formamidinium ion 36

 6.2 Binding of 24-crown-8 to *N*-3,5-di-*tert*-butylbenzylformamidinium ion (3a) 38

 6.3 Binding of 24-crown-8 to *N,N'*-dibenzylformamidinium ion (S1) 41

 6.4 Binding of 24-crown-8 to *N,N'*-dibenzylformamidine 45

 6.5 Binding of 24-crown-8 to ammonium ion 47

7. Studies of the reaction pathway of the amidinium [2]rotaxanes self-assembly 49

 7.1. Kinetic studies of the self-assembly of [2]rotaxanes via amidinium exchange 50

 7.2. Influence of NH_3 release/addition during the reaction on the rotaxane yield 52

 7.3. Rotaxane yield dependence on formamidinium salt, crown ether and amine 54

 7.4. Comparison between reaction pathways of passive template, active template and "inhibitive" template approaches toward rotaxane self-assembly 63

 7.5. Mechanism of the rotaxane self-assembly by amidinium exchange 64

8. *E,E*/*E,Z* isomerization in (mechanically interlocked) amidinium ions 65

 8.1. Isomerization thermodynamics in S1 and S1⊂24C8 65

 8.2 Isomerization kinetics and thermodynamics in 1a and 2a 66

 8.3 Chemically fueled control over isomerization rate in 1b 74

9. Dynamic covalent properties of the amidinium [2]rotaxanes 77

 9.1. Impact of the mechanical bond on the amidinium exchange 77

 9.2. Dynamic combinatorial libraries (DCL) of the amidinium [2]rotaxanes 80

10. NMR spectra 84

11. X-ray crystallographic data 104

12. References 110

1. General methods and abbreviations

All commercially available reagents and solvents were purchased from Sigma Aldrich, Alfa Aesar, ACROS Organics, TCI, Fluorochem or VWR and were used without further purification.

NMR spectra were recorded on Bruker Avance 400 or Bruker Avance 500 (^{1}H: 400 or 500 MHz; ^{13}C: 101 MHz) spectrometers at 295 K (if not otherwise specified) and referenced to the residual solvent peak (^{1}H: $CDCl_3$, 7.26 ppm; CD_3CN, 1.94 ppm, DMSO-d_6, 2.50 ppm; CD_2Cl_2, 5.32 ppm; ^{13}C: $CDCl_3$, 77.16 ppm; CD_3CN, 1.32 ppm, DMSO-d_6, 39.52 ppm, CD_2Cl_2, 53.84 ppm). Chemical shifts (δ) are denoted in ppm. High-resolution mass spectra (including tandem mass spectra) were recorded either on Bruker SolariX FT-ICR or Agilent QTOF 6546 mass spectrometer (ESI, positive polarity; solvent: acetonitrile).

All samples for HRMS or LCMS analysis were typically prepared by diluting 1-3 µL of the analyzed solution (e.g., a reaction mixture) in 1 mL of LCMS-grade acetonitrile. This extent of dilution was sufficient to stop (or slow down) the dynamic covalent exchange in order to reliably quantify the analytes by HPLC. LCMS analysis of the rotaxane self-assemblies and purification of the rotaxanes **1c** – **1e** were performed on Shimadzu LCMS-2020 using Ascentis® C8 HPLC columns (analytical column – 10 cm × 4.6 mm, particle size – 3 µm; semi-preparative column – 25 cm × 10 mm, particle size – 5 µm) or Kinetex® C18 HPLC column (analytical column – 10 cm × 4.6 mm, particle size – 2.6 µm). Isocratic elution was applied using 90% mobile phase B and 10% mobile phase A at 50 or 60 °C and flow rate 1.0 or 1.2 mL/min (analytical HPLC) and 3.0 mL/min (semi-preparative HPLC). Mobile phase A: 0.023 M HCO_2NH_4 and 0.0019 M HCO_2H in H_2O. Mobile phase B: acetonitrile. HPLC monitoring was performed with a photodiode array detector at λ = 220 nm.

List of abbreviations

24C8	24-crown-8
27C9	27-crown-9
BArF	tetrakis[3,5-bis(trifluoromethyl)-phenyl]borate
Boc	*tert*-butoxycarbonyl
DB24C8	dibenzo-24-crown-8
DBU	1,8-diazabicyclo[5.4.0]undec-7-ene
DCL	dynamic combinatorial library
DIPEA	diisopropylethylamine
DMAP	4-*N*,*N*-dimethylaminopyridine
DMF	*N*,*N*-dimethylformamide
DMFA	*N*,*N'*-dimethylformamidinium
DMSO	dimethyl sulfoxide
DPFA	*N*,*N'*-diphenylformamidinium
EtOAc	ethyl acetate
EXSY	exchange spectroscopy
FA	formamidinium
HPLC	high-performance liquid chromatography
HRMS	high-resolution mass spectrometry
LCMS	liquid chromatography – mass spectrometry
MeCN	acetonitrile
NMR	nuclear magnetic resonance
PhMe	toluene
r.t.	room temperature
TBAI	tetrabutylammonium iodide
TBDMS	*tert*-butyldimethylsilyl
TBTU	2-(1H-benzotriazole-1-yl)-1,1,3,3-tetramethylaminium tetrafluoroborate
TCA	trichloroacetic acid
TFA	trifluoroacetic acid
THF	tetrahydrofuran
TLC	thin layer chromatography

2. Synthesis of building blocks for the amidinium [2]rotaxanes

2.1 Synthesis of substrates for amidinium exchange

Formamidinium tetraphenylborate (FA·BPh$_4$)

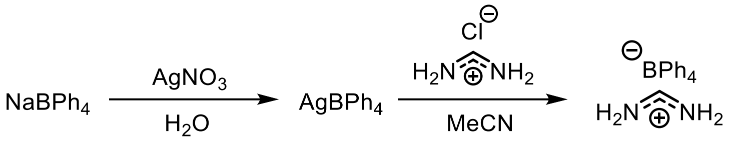

A solution of NaBPh$_4$ (2.00 g, 5.84 mmol, 1.0 eq.) in H_2O (30 mL) was filtered through a 0.45 µm syringe filter and then mixed with a solution of AgNO$_3$ (1.99 g, 11.7 mmol, 2.0 eq.) in H_2O (12 mL). The white precipitate was filtered off, washed with water and resuspended in MeCN (100 mL). Formamidinium chloride (468 mg, 5.88 mmol, 1.0 eq.) was added to the suspension and the reaction mixture was stirred at r.t. overnight in the absence of light. The precipitate was removed by filtration; the filtrate was collected and concentrated under reduced pressure to dryness. The white residue was re-dissolved in MeCN (~25 mL), the solution was filtered through a 0.45 µm syringe filter and mixed with PhMe (~70 mL). The resulting solution was kept in a fridge (+4 °C) overnight. Colorless needles of **FA·BPh$_4$** (1.29 g, 3.54 mmol, 61% over 2 steps) were filtered off and dried under high vacuum.

Note. Alternatively, the product can be obtained by mixing aqueous solutions of NaBPh$_4$ and formamidinium chloride, followed by filtration and thorough drying of the resulting white precipitate.

^{1}H NMR (400 MHz, DMSO-*d$_6$*) δ 8.78 (bs, 4H, NH$_2$ *amidinium*), 7.85 (s, 1H, CH *amidinium*), 7.17 (m, 8H, CH *BPh$_4$*), 6.92 (t, *J* = 7.4 Hz, 8H, CH *BPh$_4$*), 6.78 (t, *J* = 7.2 Hz, 4H, CH *BPh$_4$*).
^{13}C NMR (101 MHz, DMSO-*d$_6$*) δ 163.37 (q, $^1J_{B\text{–}C}$ = 49.4 Hz, C$_{Ar}$ *BPh$_4$*), 157.20, 135.54 (m, C$_{Ar}$ *BPh$_4$*), 125.31 (q, *J* = 2.5 Hz, C$_{Ar}$ *BPh$_4$*), 121.53 (C$_{Ar}$ *BPh$_4$*).
HRMS (ESI) (for BPh_4^-): *m/z* 319.16727 $[M]^-$ (calculated for $C_{24}H_{20}B^-$ 319.16635).

***N,N'*-diphenylformamidinium tetrakis[3,5-bis(trifluoromethyl)phenyl]borate (DPFA·BArF)**

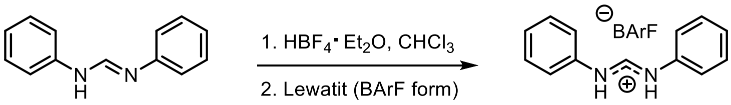

To a stirred solution of *N,N'*-diphenylformamidine (90 mg, 0.46 mmol, 1.0 eq.) in CHCl$_3$ (2 mL), HBF$_4$·Et$_2$O (68 µL, 0.46 mmol, 1.0 eq.) was added at r.t. The resulting heterogeneous mixture was diluted with CHCl$_3$ (1 mL), white crystalline solid of *N,N'*-diphenylformamidinium tetrafluoroborate (**DPFA·BF$_4$**) was filtered off and dried under high vacuum (104 mg, 0.37 mmol, 80%).

DPFA·BF$_4$ (17.5 mg, 61.6 µmol) was dissolved in MeCN (3 mL) and stirred with Lewatit® MonoPlus M500 (BArF form, see preparation in Section 4, General procedure) for 15 min at r.t. The solution was transferred to a new portion of the resin and stirred again for 15 min. The solution was filtered and the solvent was removed from the filtrate under reduced pressure to afford **DPFA·BArF** as yellowish glassy solid (60.0 mg, 56.6 µmol, 92%).

^{1}H NMR (400 MHz, CD$_3$CN)* δ 9.72 (s, 2H, NH *amidinium*), 8.57 (s, 1H, CH *amidinium E,E or E,Z*), 8.40 (s, 1H, CH *amidinium E,Z or E,E*), 7.78 – 7.20 (m, 22H, CH *BArF* + CH *Ph*).
*In solution, the product exists as a mixture of *E,E* and *E,Z* isomers. It was not possible to assign the amidinium CH signals to a specific isomer.

^{13}C NMR (101 MHz, CD_3CN) δ 162.70 (q, $^{1}J_{B-C}$ = 49.8 Hz, C_{Ar} *BArF*), 154.16, 152.97, 137.12, 136.85, 135.73 (C_{Ar} *BArF)*, 131.77, 131.12, 131.00, 130.95, 130.02 (q, $^{2}J_{C-F}$ = 31.3 Hz, C_{Ar} *BArF)*, 129.59, 128.94, 126.77, 125.55 (q, $^{1}J_{C-F}$ = 272 Hz, *CF_3 BArF*), 121.59, 121.21, 118.74 (m, C_{Ar} *BArF*).
HRMS (ESI): *m/z* 197.10734 $[M+H]^+$ (calculated for $C_{13}H_{13}N_2^+$ 197.10732).

N,N'-dimethylformamidinium tetraphenylborate (DMFA·BPh₄)

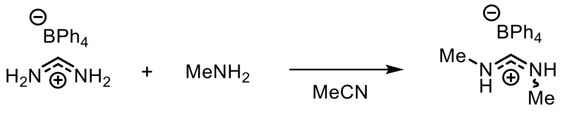

Methylamine (2.0 M solution in THF, 115 µL, 0.23 mmol, 2.2 eq.) was added to a solution of **FA·BPh$_4$** (38 mg, 0.105 mmol, 1.0 eq.) in MeCN (900 µL). The reaction mixture was stirred in a tightly closed flask at r.t. for 30 min, followed by reflux (open air) for 30 min. After cooling down the reaction mixture to r.t., an additional amount of methylamine (2.0 M solution in THF, 50 µL, 0.10 mmol, 0.95 eq.) was added and the mixture was stirred at r.t. for 30 min. All volatiles were removed under reduced pressure to afford the product as white crystalline solid (37 mg, 0.094 mmol, 90%).

^{1}H NMR (400 MHz, CD_3CN)* 7.41 (s, 1H, CH *amidinium*), 7.32 – 7.25 (m, 8H, CH *BPh_4*), 7.01 (t, *J* = 7.4 Hz, 8H, CH *BPh_4*), 6.86 (t, *J* = 7.2 Hz, 4H, CH *BPh_4*), 3.00 (s, 3H, CH_3), 2.77 (s, 3H, CH_3).
*In solution, the product exists mainly as *E,Z* isomer.
^{13}C NMR (100 MHz, $CDCl_3$) δ 164.80 (q, $^{1}J_{B-C}$ = 49.5 Hz, C_{Ar} *BPh_4*), 157.06, 136.70 (m, C_{Ar} *BPh_4*), 126.62 (q, *J* = 2.7 Hz, C_{Ar} *BPh_4*), 122.79, 33.72, 28.98.
HRMS (ESI): *m/z* 73.07587 $[M+H]^+$ (calculated for $C_3H_9N_2^+$ 73.07602).

2.2 Synthesis of crown ethers

24-crown-8 (24C8)

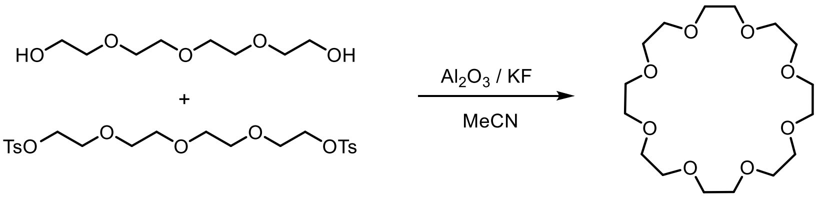

24C8 was synthesized according to ref. 1[1] and purified according to ref. 2.[2]

A solution of tetraethylene glycol (5.32 g, 27.4 mmol, 1.0 eq.) in anhydrous MeCN (40 mL) was added to a solution of tetraethylene glycol di(*p*-toluenesulfonate) (13.76 g, 27.4 mmol, 1.0 eq.) in anhydrous MeCN (120 mL) at 0 °C. Potassium fluoride on Al_2O_3 support[3] (type B, 19.9 g containing 7.96 g/137 mmol/5.0 eq. KF) was added and the resulting suspension was stirred under Ar atmosphere at r.t. for 4 days. The solid was filtered off, washed with MeCN and the solvent was removed from the filtrate under reduced pressure. The crude product was extracted from the obtained residue with hot *n*-hexane (7×70 mL). The solvent was removed under reduced pressure from the combined extracts and the resulting colorless oil was dissolved in MeCN (5 mL). The solution was filtered through a 0.45 µm syringe filter and kept then at –20 °C overnight. MeCN was removed from the colorless crystals of **24C8**·MeCN complex with a Pasteur pipette. The crystals were rapidly washed with cold (ca. –20 °C) MeCN (2×1 mL). Drying the crystals (which melted above 0 °C) on a rotary evaporator (T_{bath} = 55 °C, p ≈ 3 mbar) and then under high vacuum at 50 °C afforded 24-crown-8 as colorless oil (1.58 g, 4.48 mmol, 16%).

^{1}H NMR (400 MHz, $CDCl_3$) δ 3.68 (s, 32H).
^{13}C NMR (101 MHz, $CDCl_3$) δ 70.99.

HRMS (ESI): *m/z* 375.19875 $[M+Na]^+$ (calculated for $C_{16}H_{32}NaO_8^+$ 375.19894).

27-crown-9 (27C9)

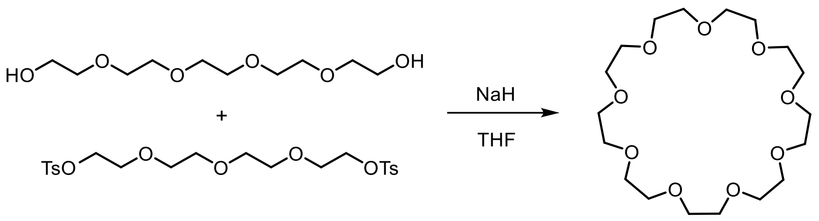

27C9 was synthesized according to a reported procedure.[4]

A 500 mL three-necked round-bottom flask was charged with NaH (0.800 g 60% dispersion in mineral oil, 20.0 mmol, 4.0 eq.), which was subsequently washed with n-hexane under nitrogen atmosphere. Anhydrous THF (75 mL) was added to the flask and the resulting suspension was cooled down to 0 °C. A solution of pentaethylene glycol (1.19 g, 5.00 mmol, 1.0 eq.) in THF (50 mL) was added dropwise to the stirred suspension. The cooling bath was removed and a solution of tetraethylene glycol di(*p*-toluenesulfonate) (2.51 g, 5.00 mmol, 1.0 eq.) in THF (90 mL) was added dropwise over 3 h. The reaction mixture was then stirred at r.t. under nitrogen atmosphere for 2 days. The ice-cooled reaction mixture was then carefully quenched with H_2O (2 mL). The reaction mixture was concentrated under reduced pressure. The aqueous residue was diluted with H_2O (25 mL) and the product was extracted with $CHCl_3$ (3×100 mL). The combined organic phase was dried with anhydrous $MgSO_4$ and the solvent was removed under reduced pressure. The crude product was purified by flash column chromatography on silica gel (CH_2Cl_2 → CH_2Cl_2/MeOH = 95:5) to afford the product as colorless oil (0.852 g, 2.15 mmol, 43%).

^{1}H NMR (400 MHz, $CDCl_3$) δ 3.67 (s, 36H).
^{13}C NMR (101 MHz, $CDCl_3$) δ 70.91.
HRMS (ESI): *m/z* 419.22495 $[M+Na]^+$ (calculated for $C_{18}H_{36}NaO_9^+$ 419.22515).

2.3 Synthesis of primary amines

2.3.1 Synthesis of 3,5-di-*tert*-butylbenzylamine (4a)

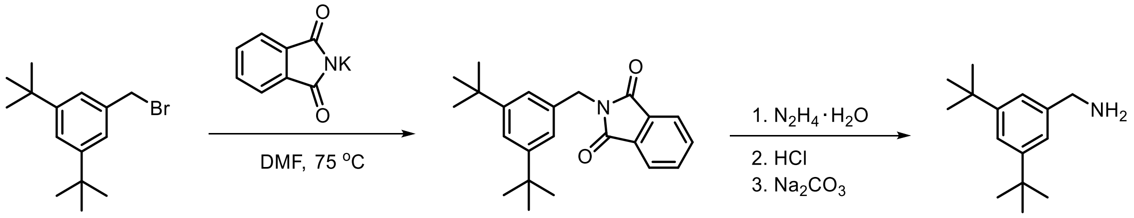

N-(3,5-Di-*tert*-butylbenzyl)phthalimide

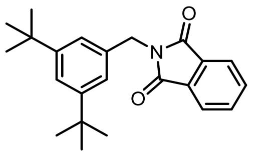

N-(3,5-di-*tert*-butylbenzyl)phthalimide was synthesized according to a reported procedure.[5]

A mixture of 3,5-di-*tert*-butylbenzyl bromide (2.50 g, 8.83 mmol, 1.0 eq.) and potassium phthalimide (1.96 g, 10.6 mmol, 1.2 eq.) was dissolved in anhydrous DMF (30 mL) and stirred at 75 °C under argon atmosphere for 6 h (TLC monitoring). DMF was removed under reduced pressure. Water was added to the residue and

the product was extracted with EtOAc (3×60 mL). The combined organic phase was washed with brine and dried over anhydrous Na_2SO_4. After removal of the solvent under reduced pressure, the crude product was recrystallized from MeOH to afford *N*-(3,5-di-*tert*-butylbenzyl)phthalimide as white crystalline solid (2.82 g, 8.07 mmol, 91%).

^{1}H NMR (400 MHz, $CDCl_3$) δ 7.84 (dd, *J* = 5.4, 3.0 Hz, 2H, CH *Ar*), 7.69 (dd, *J* = 5.5, 3.0 Hz, 2H, CH *Ar*), 7.34 (m, 3H, CH *Ar*), 4.82 (s, 2H, CH_2 *benzylic*), 1.31 (s, 18H, CH_3 *t-Bu*).
^{13}C NMR (101 MHz, $CDCl_3$) δ 168.24, 151.29, 135.69, 133.99, 132.38, 123.53, 123.41, 122.04, 42.34, 34.95, 31.57.

3,5-Di-*tert*-butylbenzylamine (4a)

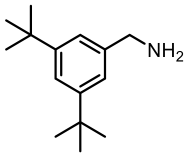

3,5-Di-*tert*-butylbenzylamine was synthesized according to the reported procedure.[5]

Hydrazine monohydrate (1.4 mL, 28.6 mmol, 4.0 eq.) was added to a suspension of *N*-(3,5-di-*tert*-butylbenzyl)phthalimide (2.50 g, 7.15 mmol, 1.0 eq.) in ethanol (45 mL). The reaction mixture was stirred under reflux for 20 min and cooled down to room temperature. After addition of 37% aq. HCl (3.6 mL), the mixture was refluxed for 15 min. Water (50 mL) was added and a white precipitate was filtered off. The precipitate was washed with water (30 mL) and combined filtrate was concentrated under reduced pressure, so that most of EtOH was removed. The resulting precipitate was collected and partially dissolved in CH_2Cl_2 (500 mL). The filtered solution was washed with sat. aq. Na_2CO_3 (2×50 mL), dried over $MgSO_4$ and the solvent was removed under reduced pressure to afford 3,5-di-*tert*-butylbenzylamine as yellowish oil that slowly crystallized (1.19 g, 5.42 mmol, 76%).

^{1}H NMR (400 MHz, $CDCl_3$) δ 7.33 (t, *J* = 1.9 Hz, 1H), 7.16 (d, *J* = 1.8 Hz, 2H), 3.87 (s, 2H), 1.34 (s, 18H).
^{13}C NMR (101 MHz, $CDCl_3$) δ 151.12, 142.69, 121.43, 121.04, 47.33, 35.00, 31.64.
HRMS (ESI): *m/z* 220.20618 $[M+H]^+$ (calculated for $C_{15}H_{26}N^+$ 220.20598).

2.3.2 Synthesis of 2-(3,5-di-*tert*-butylbenzyloxy)ethylamine (4b)

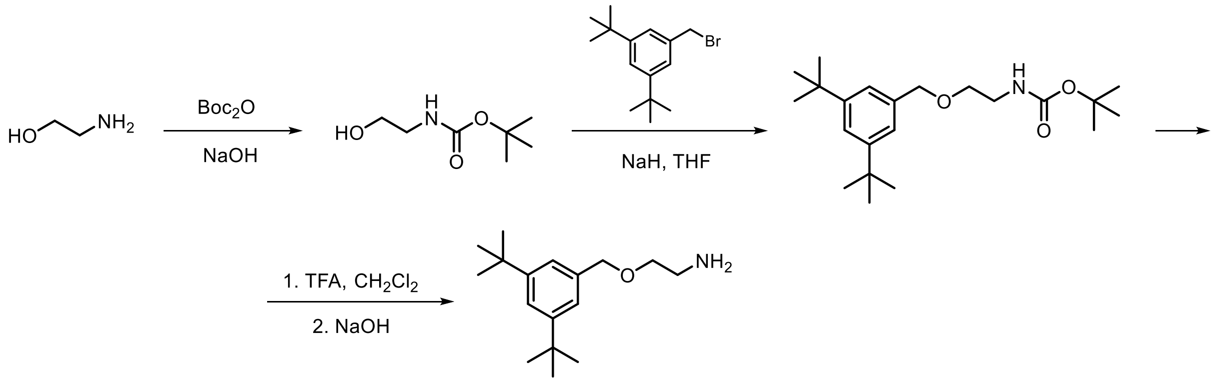

N-Boc-2-aminoethanol

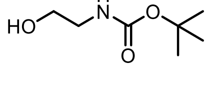

To a stirred solution of Boc_2O (6.26 g, 28.7 mmol, 1.1 eq.) in THF (100 mL), 2-aminoethanol (1.58 mL, 26.1 mmol, 1.0 eq.) was added followed by a solution of NaOH (2.09 g, 52.2 mmol, 2.0 eq.) in H_2O (50 mL). The reaction mixture was stirred at r.t. for 3 h. THF was removed under reduced pressure. Saturated aq. NH_4Cl (100 mL) was added to the residue and the product was extracted with CH_2Cl_2 (4×30 mL). The combined organic phase was dried over Na_2SO_4 and the solvent was removed under reduced pressure. The crude product was purified by flash column chromatography on silica gel (cyclohexane/EtOAc = 9:1 ⟶ 3:7) to afford the product as colorless liquid (3.30 g, 20.5 mmol, 79%).

^{1}H NMR (400 MHz, $CDCl_3$) δ 4.91 (s, 1H, NH), 3.71 (q, *J* = 5.0 Hz, 2H, CH_2), 3.29 (q, *J* = 5.4 Hz, 2H, CH_2), 2.28 (s, 1H, OH), 1.45 (s, 9H, CH_3 *t-Bu*).*

*^{1}H NMR data obtained is in accordance with the reported NMR data for *N*-Boc-2-aminoethanol.[6]

***N*-Boc-2-(3,5-di-*tert*-butylbenzyloxy)ethylamine**

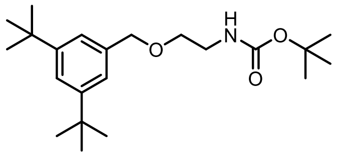

A solution of N-Boc-2-aminoethanol (455 mg, 2.82 mmol, 2.0 eq.) in anhydrous THF (7 mL) was cooled down to 0 °C. Sodium hydride (68 mg 60% suspension in mineral oil, 2.82 mmol, 2.0 eq.) was carefully added and the mixture was stirred under Ar atmosphere for 30 min. A solution of 3,5-di-*tert*-butylbenzyl bromide (400 mg, 1.41 mmol, 1.0 eq.) in anhydrous THF (3 mL) was then added followed by TBAI (521 mg, 1.41 mmol, 1.0 eq.). The reaction mixture was stirred under Ar atmosphere at room temperature overnight. After cooling down the reaction mixture to 0 °C, it was quenched with H_2O (0.5 mL). The solvent was removed under reduced pressure. The obtained residue was dissolved in a mixture of CH_2Cl_2 (20 mL) and H_2O (30 mL); the layers were then separated. The aqueous phase was extracted with CH_2Cl_2 (2×20 mL). The combined organic phases were dried over Na_2SO_4 and the solvent was removed under reduced pressure. The crude product was purified by flash column chromatography on silica gel (cyclohexane ⟶ cyclohexane/EtOAc = 8:2) to afford the product as colorless oil that slowly crystallized (404 mg, 1.11 mmol, 79%).

^{1}H NMR (400 MHz, $CDCl_3$) δ 7.37 (t, *J* = 1.9 Hz, 1H, CH *Ar*), 7.17 (d, *J* = 1.8 Hz, 2H, CH *Ar*), 4.92 (s, 1H, NH), 4.51 (s, 2H, CH_2 *benzylic*), 3.56 (t, *J* = 5.1 Hz, 2H, CH_2), 3.35 (q, *J* = 5.4 Hz, 2H, CH_2), 1.44 (s, 9H, CH_3 *Boc*), 1.33 (s, 18H, CH_3 *t-Bu Ar*).
^{13}C NMR (101 MHz, $CDCl_3$) δ 156.11, 151.07, 137.19, 122.20, 122.06, 79.32, 73.94, 69.42, 40.60, 34.96, 31.62, 28.55.
HRMS (ESI): *m/z* 386.26677 $[M+Na]^+$ (calculated for $C_{22}H_{37}NNaO_3^+$ 386.26657).

2-(3,5-di-*tert*-butylbenzyloxy)ethylamine (4b)

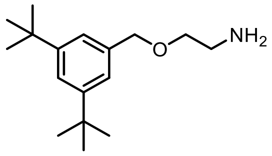

N-Boc-2-(3,5-di-*tert*-butylbenzyloxy)ethylamine (398 mg, 1.09 mmol) was dissolved in CH_2Cl_2 (2 mL). TFA (1 mL) was added and the reaction mixture was stirred at r.t. for 45 min. All volatiles were removed under reduced pressure. The residue was dissolved in CH_2Cl_2 (15 mL) and the resulting solution was washed with 1 M aq. NaOH (2×5 mL). The organic phase was dried over Na_2SO_4 and the solvent was removed under reduced pressure to afford the product as yellowish oil (277 mg, 1.05 mmol, 96%). The obtained amine was used without further purification.

^{1}H **NMR** (400 MHz, $CDCl_3$) δ 7.36 (t, *J* = 1.9 Hz, 1H, CH *Ar*), 7.18 (d, *J* = 1.8 Hz, 2H, CH *Ar*), 4.52 (s, 2H, CH_2 *benzylic*), 3.54 (t, *J* = 5.2 Hz, 2H, CH_2O), 2.91 (t, *J* = 5.2 Hz, 2H, CH_2N), 1.38 (s, 2H, NH_2), 1.33 (s, 18H, CH_3 *t-Bu Ar*).
^{13}C **NMR** (101 MHz, $CDCl_3$) δ 150.97, 137.44, 122.24, 121.94, 74.01, 72.76, 42.15, 34.95, 31.61.
HRMS (ESI): *m/z* 264.23233 $[M+H]^+$ (calculated for $C_{17}H_{30}NO^+$ 264.23219).

2.3.3 Synthesis of 3-amino-*N*-(3,5-di-*tert*-butylphenyl)propanamide (4c)

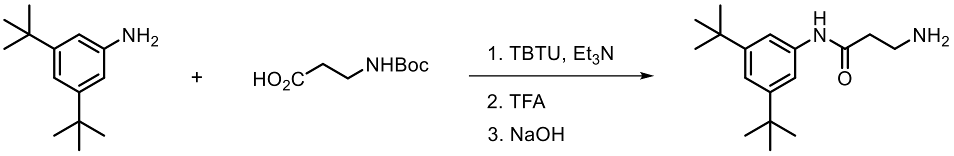

***tert*-Butyl (3-((3,5-di-*tert*-butylphenyl)amino)-3-oxopropyl)carbamate**

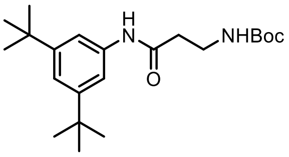

N-Boc-β-alanine (702 mg, 3.71 mmol, 1.0 eq.) was dissolved in CH_2Cl_2 (10 mL). After sequential addition of NEt_3 (1.00 mL, 7.20 mmol, 1.9 eq.) and TBTU (1.39 g, 4.32 mmol, 1.2 eq.), the mixture was stirred under Ar atmosphere at r.t for 30 min. After addition of 3,5-di-*tert*-butylaniline (887 mg, 4.32 mmol, 1.2 eq.), the reaction mixture was stirred overnight. The solvent was removed under reduced pressure and the residue was dissolved in EtOAc (40 mL). The organic phase was washed with an ice-cold 10% aq. solution of citric acid (1×5 mL), saturated aq. solution of $NaHCO_3$ (1×10 mL), water (5×5 mL) and dried over Na_2SO_4. After solvent removal, the crude product was purified by flash column chromatography on silica gel (cyclohexane/EtOAc = 95:5 ⟶ 6:4) to afford the product as white solid (1.36 g, 3.59 mmol, 97%).

^{1}H **NMR** (400 MHz, $CDCl_3$) δ 7.40 (bs, 1H, NH), 7.38 (d, *J* = 1.6 Hz, 2H, CH *Ar*), 7.18 (t, *J* = 1.6 Hz, 1H, CH *Ar*), 5.17 (bs, 1H, NH), 3.50 (q, *J* = 6.1 Hz, 2H, CH_2N), 2.60 (t, *J* = 5.8 Hz, 2H, CH_2CO), 1.44 (s, 9H, CH_3 *Boc*), 1.32 (s, 18H, CH_3 *t-Bu Ar*).
^{13}C **NMR** (101 MHz, $CDCl_3$) δ 169.70, 156.42, 151.80, 137.31, 118.69, 114.52, 79.64, 37.67, 36.61, 35.07, 31.53, 28.55.
HRMS (ESI): *m/z* 399.26166 $[M+Na]^+$ (calculated for $C_{22}H_{36}N_2NaO_3^+$ 399.26181).

3-Amino-*N*-(3,5-di-*tert*-butylphenyl)propanamide (4c)

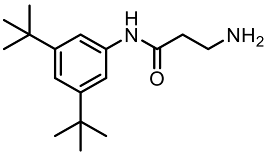

TFA (2.0 mL) was added to a solution of Boc-protected 3-amino-*N*-(3,5-di-*tert*-butylphenyl)propanamide (1.36 g, 3.59 mmol) in CH_2Cl_2 (25 mL). The reaction mixture was stirred at r.t. for 3 h. All volatiles were removed under reduced pressure. The residue was dissolved in CH_2Cl_2 (65 mL) and the resulting solution was washed with 1 M aq. NaOH (2×35 mL). The organic phase was dried over Na_2SO_4 and the solvent was removed under reduced pressure to afford the product as grayish solid (1.00 g, 3.60 mmol, quantitative yield). The obtained amine was used without further purification.

^{1}H NMR (400 MHz, $CDCl_3$) δ 9.54 (bs, 1H, NHCO), 7.40 (d, *J* = 1.7 Hz, 2H, CH *Ar*), 7.15 (t, *J* = 1.8 Hz, 1H, CH *Ar*), 3.12 (t, *J* = 5.8 Hz, 2H, CH_2N), 2.47 (t, *J* = 5.8, 2H, CH_2CO), 1.49 (bs, 2H, NH_2), 1.32 (s, 18H, CH_3 *t-Bu*).
^{13}C NMR (101 MHz, $CDCl_3$) δ 170.94, 151.59, 137.86, 118.20, 114.59, 39.13, 38.12, 35.01, 31.52.
HRMS (ESI): *m/z* 277.22791 $[M+H]^+$ (calculated for $C_{17}H_{29}N_2O^+$ 277.22744).

2.3.4 Synthesis of *N*-(2-aminoethyl)-3,5-di-*tert*-butylbenzamide (4d)

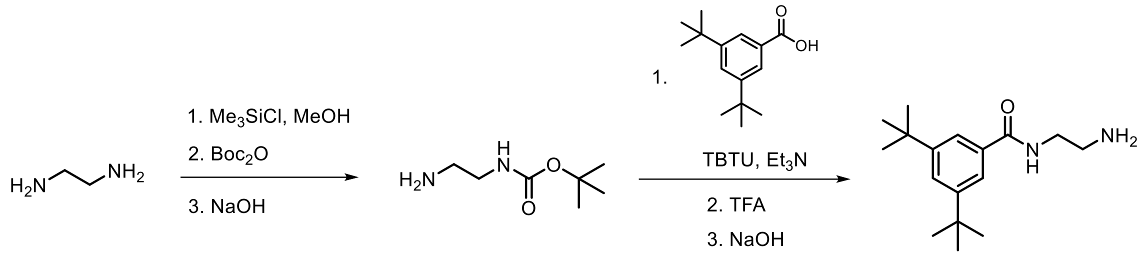

N-Boc-ethylenediamine

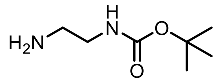

N-Boc-ethylenediamine was synthesized according to the reported procedure.[7]

Trimethylsilyl chloride (3.83 mL, 30.1 mmol, 1.0 eq.) was added dropwise to a solution of ethylenediamine (2.01 mL, 30.1 mmol, 1.0 eq.) in MeOH (12 mL) at 0 °C. The mixture was allowed to warm up to r.t. and water (2 mL) was then added followed by a solution of $(Boc)_2O$ (6.58 g, 30.2 mmol, 1.0 eq.) in MeOH (6 mL). After 1 h of stirring, the mixture was diluted with water (100 mL) and washed with diethyl ether (2×75 mL). Then, pH of the aqueous phase was set to ~12 with a 2 M aq. solution of NaOH. The solution was extracted with CH_2Cl_2 (3×50 mL) and the combined phase was dried over Na_2SO_4. Evaporation of the solvent under reduced pressure afforded *N*-Boc-ethylenediamine as colorless oil (1.49 g, 9.32 mmol, 31 %).

^{1}H NMR (400 MHz, $CDCl_3$)* δ 4.95 (bs, 1H, NHCO), 3.14 (q, *J* = 5.9 Hz, 2H, <u>CH_2</u>NHCO), 2.77 (t, *J* = 6.0 Hz, 2H, <u>CH_2</u>NH_2), 1.42 (s, 9H, CH_3 *Boc*), 1.18 (s, 2H, NH_2).
*^{1}H NMR data obtained is in accordance with the reported NMR data for *N*-Boc-ethylenediamine.[8]

tert-Butyl-(2-(3,5-di-*tert*-butylbenzamido)ethyl)carbamate

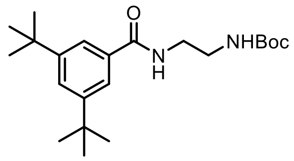

3,5-Di-*tert*-butylbenzoic acid (803 mg, 3.43 mmol, 1.0 eq.) was dissolved in anhydrous CH_2Cl_2 (10 mL). After sequential addition of NEt_3 (0.96 mL, 6.86 mmol, 2.0 eq.) and TBTU (1.32 g, 4.11 mmol, 1.2 eq.), the mixture was stirred under Ar at r.t. for 30 min. After addition of *N*-Boc-ethylenediamine (746 mg, 4.11 mmol, 1.2 eq.), the reaction mixture was stirred overnight. The white precipitate (F1) was then filtered off. The filtrate was concentrated under reduced pressure and the residue (F2) was purified by flash column chromatography on silica gel (cyclohexane/EtOAc = 9:1 → 7:3). The precipitate F1 was dissolved in EtOAc (20 mL). The organic phase was washed with sodium bicarbonate solution (2×5 mL), water (3×7 mL), brine (1×7 mL), dried over Na_2SO_4 and the solvent was removed under reduced pressure. After combination of both fractions (F1 and F2), *tert*-butyl-(2-(3,5-di-*tert*-butylbenzamido)ethyl)carbamate was obtained as white solid (1.17 g, 3.11 mmol, 90%).

^{1}**H NMR** (400 MHz, $CDCl_3$) δ 7.67 (bs, 2H, CH *Ar*), 7.55 (t, *J* = 1.8 Hz, 1H, CH *Ar*), 7.23 (bs, 1H, NH), 4.99 (bs, 1H, NH), 3.57 (m, 2H, CH_2), 3.40 (m, 2H, CH_2), 1.42 (s, 9H, CH_3 *Boc*), 1.35 (s, 18H, CH_3 *t-Bu Ar*).
^{13}**C NMR** (101 MHz, $CDCl_3$) δ 168.80, 157.62, 151.23, 133.77, 125.66, 121.34, 80.00, 42.19, 40.51, 35.12, 31.53, 28.47.
HRMS (ESI): *m/z* 377.27992 $[M+H]^+$ (calculated for $C_{22}H_{37}N_2O_3^+$ 377.27987).

N-(2-aminoethyl)-3,5-di-*tert*-butylbenzamide (4d)

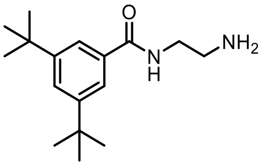

TFA (4.0 mL) was added to a solution of Boc-protected *N*-(2-aminoethyl)-3,5-di-*tert*-butylbenzamide (1.17 g, 3.11 mmol) in CH_2Cl_2 (30 mL). The reaction mixture was stirred at r.t. for 3 h. All volatiles were removed under reduced pressure. The residue was dissolved in CH_2Cl_2 (40 mL) and the resulting solution was washed with 1 M aq. NaOH (2×30 mL). The organic phase was dried over Na_2SO_4 and the solvent was removed under reduced pressure to afford the product as white solid (746 mg, 2.70 mmol, 87%). The obtained amine was used without further purification.

^{1}**H NMR** (400 MHz, $CDCl_3$) δ 7.60 (d, *J* = 1.9 Hz, 2H, CH *Ar*), 7.55 (t, *J* = 1.8 Hz, 1H, CH *Ar*), 6.65 (bs, 1H, NH), 3.51 (q, *J* = 5.8 Hz, 2H, $\underline{CH_2}$NHCO), 2.94 (t, *J* = 6.0 Hz, 2H, $\underline{CH_2}NH_2$), 1.34 (s, 18H, CH_3 *t-Bu Ar*).
^{13}**C NMR** (101 MHz, $CDCl_3$) δ 168.98, 151.32, 134.42, 125.69, 121.20, 42.73, 41.57, 35.10, 31.52.
HRMS (ESI): *m/z* 277.22731 $[M+H]^+$ (calculated for $C_{17}H_{29}N_2O^+$ 277.22744).

2.3.5 Synthesis of 6-amino-*N*-(3,5-di-*tert*-butylbenzyl)hexanamide (4e)

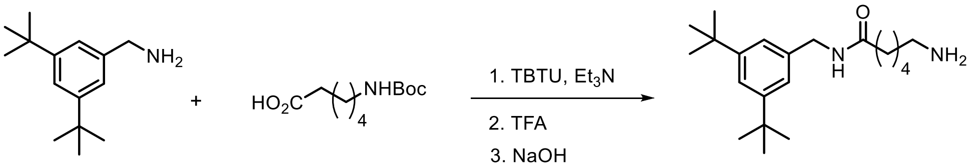

***tert*-Butyl (6-((3,5-di-*tert*-butylbenzyl)amino)-6-oxohexyl)carbamate**

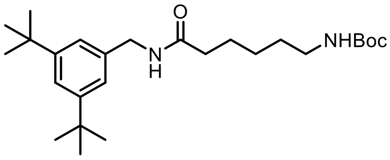

6-(Boc-amino)-caproic acid (412 mg, 1.78 mmol, 1.0 eq.) was dissolved in anhydrous CH_2Cl_2 (6 mL). After sequential addition of Et_3N (496 µL, 3.56 mmol, 2.0 eq.) and TBTU (687 mg, 2.14 mmol, 1.2 eq.), the mixture was stirred under Ar atmosphere at r.t. for 30 min. After addition of 3,5-di-*tert*-butylbenzylamine (470 mg, 2.14 mmol, 1.2 eq.), the reaction mixture was stirred overnight. The solvent was removed under reduced pressure and the residue was dissolved in EtOAc (55 mL). The organic phase was washed with an ice-cold 10% aq. solution of citric acid (1×5 mL), saturated solution of $NaHCO_3$ (1×10 mL), water (5×5 mL) and dried over Na_2SO_4. After solvent removal, the residue was purified by flash column chromatography on silica gel (cyclohexane/EtOAc = 9:1 → 6:4) to afford the product as colorless oil which crystallized upon cooling (675 mg, 1.56 mmol, 88%).

^{1}**H NMR** (400 MHz, $CDCl_3$) δ 7.35 (t, *J* = 1.8 Hz, 1H, CH *Ar*), 7.11 (d, *J* = 1.8 Hz, 2H, CH *Ar*), 5.74 (bs, 1H, NH), 4.56 (bs, 1H, NH), 4.42 (d, *J* = 5.5 Hz, 2H, CH_2 *benzylic*), 3.10 (q, *J* = 6.8 Hz, 2H, CH_2N), 2.21 (t, *J* = 7.5 Hz, 2H, CH_2CO), 1.69 (m, 2H, CH_2), 1.54 – 1.45 (m, 2H, CH_2), 1.43 (s, 9H, CH_3 *Boc*), 1.40 – 1.32 (m, 2H, CH_2), 1.31 (s, 18H, CH_3 *t-Bu Ar*).
^{13}**C NMR** (101 MHz, $CDCl_3$) δ 172.67, 156.13, 151.43, 137.46, 122.38, 121.80, 79.20, 44.39, 40.47, 36.76, 34.98, 31.57, 29.94, 28.55, 26.56.
HRMS (ESI): *m/z* 433.34226 $[M+H]^+$ (calculated for $C_{26}H_{45}N_2O_3^+$ 433.34247).

6-Amino-*N*-(3,5-di-*tert*-butylbenzyl)hexanamide (4e)

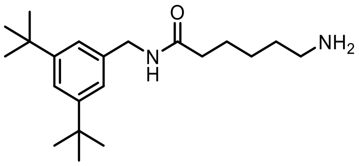

TFA (3.0 mL) was added to a solution of Boc-protected 6-amino-*N*-(3,5-di-*tert*-butylbenzyl)hexanamide (675 mg, 1.56 mmol) in CH_2Cl_2 (10 mL). The reaction mixture was stirred at r.t. overnight. All volatiles were then removed under reduced pressure. The residue was dissolved in CH_2Cl_2 (20 mL) and the resulting solution was washed with 1 M aq. NaOH (2×10 mL). The organic phase was dried over Na_2SO_4 and the solvent was removed under reduced pressure to afford the product as colorless oil (366 mg, 1.10 mmol, 71%). The obtained amine was used without further purification.

^{1}**H NMR** (400 MHz, $CDCl_3$) δ 7.35 (t, *J* = 1.9 Hz, 1H, CH *Ar*), 7.11 (d, *J* = 1.8 Hz, 2H, CH *Ar*), 5.77 (bs, 1H, NH), 4.42 (d, *J* = 5.5 Hz, 2H, CH_2 *benzylic*), 2.69 (t, *J* = 6.9 Hz, 2H, CH_2N), 2.22 (t, *J* = 7.5 Hz, 2H, CH_2CO), 1.74 – 1.63 (m, 2H, CH_2), 1.67 (bs, 2H, NH_2), 1.47 (m, 2H, CH_2), 1.38 (m, 2H, CH_2), 1.31 (s, 18H, CH_3 *t-Bu Ar*).
^{13}**C NMR** (101 MHz, $CDCl_3$) δ 172.75, 151.44, 137.51, 122.38, 121.79, 44.38, 42.00, 36.86, 34.98, 33.30, 31.58, 26.65, 25.71.
HRMS (ESI): *m/z* 333.28963 $[M+H]^+$ (calculated for $C_{21}H_{37}N_2O^+$ 333.29004).

2.3.6 Synthesis of 2-(4-tritylphenoxy)ethylamine (4f) and 4-(4-tritylphenoxy)butylamine (4g)

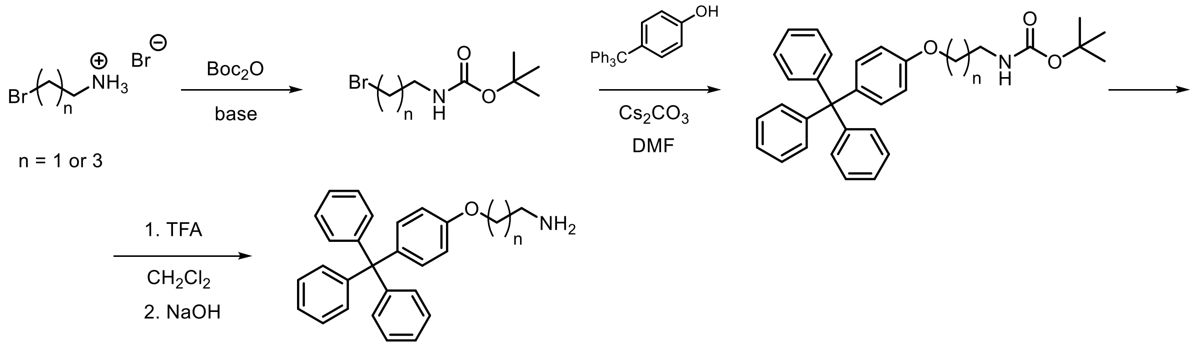

N-Boc-2-bromoethylamine

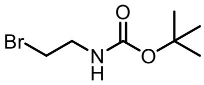

To a stirred solution of Boc_2O (1.50 g, 6.87 mmol, 1.1 eq.) in 1,4-dioxane/H_2O mixture (26 mL / 14 mL respectively), 2-bromoethylamine hydrobromide (1.28 g, 6.25 mmol, 1.0 eq.) was added followed by a solution of NaOH (0.55 g, 13.7 mmol, 2.2 eq.) in H_2O (15 mL). The reaction mixture was stirred at r.t. for 6 h. The solvent was removed under reduced pressure. Saturated aq. NH_4Cl (70 mL) was added to the residue

and the product was extracted with CH_2Cl_2 (3×30 mL). The combined organic phases were dried over Na_2SO_4 and the solvent was removed under reduced pressure. The crude product was purified by flash column chromatography on silica gel (cyclohexane → cyclohexane/EtOAc = 9:1) to afford the product as colorless oil (745 mg, 3.32 mmol, 53%).

^{1}H NMR (400 MHz, $CDCl_3$) δ 4.94 (bs, 1H, NH), 3.54 (m, 2H, CH_2Br), 3.46 (m, 2H, CH_2N), 1.45 (s, 9H, CH_3 *t-Bu*).
^{13}C NMR (100 MHz, $CDCl_3$) δ 155.72, 85.30, 79.96, 42.48, 32.94, 28.47.

_N_-Boc-2-(4-tritylphenoxy)ethylamine

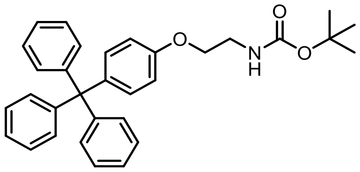

To a solution of *N*-Boc-2-bromoethylamine (734 mg, 3.28 mmol, 1.0 eq.) in anhydrous DMF (5 mL), 4-tritylphenol (1.21 g, 3.60 mmol, 1.1 eq.) was added followed by Cs_2CO_3 (3.20 g, 9.83 mmol, 3.0 eq.). The reaction mixture was stirred under Ar atmosphere at r.t. for 3 days. The solvent was removed under reduced pressure. The residue was solubilized in EtOAc/H_2O mixture (170 mL / 40 mL). The organic layer was separated and the aqueous phase was extracted with EtOAc (2×20 mL). The combined organic phase was washed with H_2O (2×15 mL), brine and dried over Na_2SO_4. The solvent was removed under reduced pressure and the crude product was purified by flash column chromatography on silica gel (cyclohexane → cyclohexane/EtOAc = 8:2) to afford the pure product as white solid (1.04 g, 2.17 mmol, 66%).

^{1}H NMR (400 MHz, $CDCl_3$) δ 7.31 – 7.15 (m, 15H, CH *Tr*), 7.11 (d, *J* = 8.9 Hz, 2H, CH *Ar*), 6.77 (d, *J* = 8.9 Hz, 2H, CH *Ar*), 5.01 (bs, 1H, NH), 3.98 (t, *J* = 5.1 Hz, 2H, CH_2O), 3.51 (q, *J* = 5.4 Hz, 2H, CH_2N), 1.45 (s, 9H, CH_3 *t-Bu*).
^{13}C NMR (100 MHz, $CDCl_3$) δ 156.60, 155.98, 147.08, 139.54, 132.38, 131.20, 127.56, 125.99, 113.33, 79.59, 67.14, 64.42, 40.22, 28.52.
HRMS (ESI): *m/z* 502.23500 $[M+Na]^+$ (calculated for $C_{32}H_{33}NNaO_3^+$ 502.23527).

2-(4-Tritylphenoxy)ethylamine (4f)

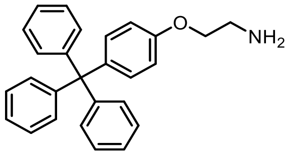

N-Boc-2-(4-tritylphenoxy)ethylamine (1.00 g, 2.08 mmol) was dissolved in CH_2Cl_2 (10 mL). TFA (3.0 mL) was added and the reaction mixture was stirred at r.t. for 2 h. All volatiles were removed under reduced pressure. The residue was dissolved in CH_2Cl_2 (150 mL) and the resulting solution was washed with 10% aq. NaOH (3×20 mL). The organic phase was washed with H_2O (30 mL) and dried over Na_2SO_4. The solvent was removed under reduced pressure to afford the product as white solid (694 mg, 1.83 mmol, 88%). The obtained amine was used without further purification.

^{1}H NMR (400 MHz, CD_2Cl_2) δ 7.32 – 7.16 (m, 15H, CH *Tr*), 7.13 (d, *J* = 8.9 Hz, 2H, CH *Ar*), 6.80 (d, *J* = 8.9 Hz, 2H, CH *Ar*), 3.94 (t, *J* = 5.2 Hz, 2H, CH_2O), 3.01 (t, *J* = 5.2 Hz, 2H, CH_2N), 1.35 (bs, 2H, NH_2).
^{13}C NMR (100 MHz, CD_2Cl_2) δ 157.45, 147.57, 139.46, 132.45, 131.36, 127.86, 126.19, 113.71, 70.83, 64.73, 42.00.
HRMS (ESI): *m/z* 380.20084 $[M+H]^+$ (calculated for $C_{27}H_{26}NO^+$ 380.20089).

N-Boc-4-bromobutylamine

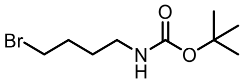

N-Boc-4-bromobutylamine was synthesized according to a reported procedure.[9]

To a stirred solution of Boc_2O (1.13 g, 5.18 mmol, 1.0 eq.) in CH_2Cl_2 (6 mL), 4-bromobutylamine hydrobromide (1.21 g, 5.18 mmol, 1.0 eq.) was added and the resulting mixture was cooled to 0 °C. Triethylamine (1.45 mL, 10.4 mmol, 2.0 eq.) was added dropwise and the reaction mixture was stirred at r.t. for 10 h. The reaction mixture was diluted with CH_2Cl_2 (30 mL). The organic phase was washed with H_2O (2×10 mL), dried over Na_2SO_4 and the solvent was removed under reduced pressure. The crude product was purified by flash column chromatography on silica gel (petrol ether → petrol ether/EtOAc = 93:7) to afford the product as colorless oil (528 mg, 2.09 mmol, 40%).

^{1}H NMR (400 MHz, $CDCl_3$)* 4.53 (bs, 1H, NH), 3.43 (t, *J* = 6.7 Hz, 2H, CH_2Br), 3.16 (q, *J* = 6.6 Hz, 2H, CH_2N), 1.89 (m, 2H, CH_2), 1.64 (m, 2H, CH_2), 1.44 (s, 9H, CH_3 *t-Bu*).

*^{1}H NMR data obtained is in accordance with the reported NMR data for *N*-Boc-4-bromobutylamine.[10]

N-Boc-4-(4-tritylphenoxy)butylamine

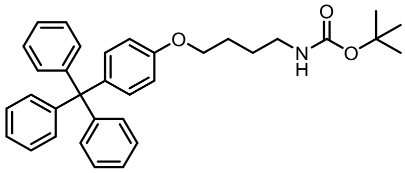

To a solution of *N*-Boc-4-bromobutylamine (527 mg, 2.09 mmol, 1.0 eq.) in anhydrous DMF (3 mL), 4-tritylphenol (736 mg, 2.19 mmol, 1.05 eq.) was added followed by Cs_2CO_3 (1.95 g, 5.97 mmol, 3.0 eq.). The reaction mixture was stirred under Ar atmosphere at r.t. for 3 days. The reaction mixture was then poured into H_2O (100 mL). The white precipitate was filtered off, washed with H_2O followed by MeOH and dried under high vacuum to afford the product as white solid (0.98 g, 1.93 mmol, 92%).

^{1}H NMR (400 MHz, $CDCl_3$) δ 7.29 – 7.15 (m, 15H, CH *Ar Tr*), 7.10 (d, *J* = 8.9 Hz, 2H, CH *Ar*), 6.76 (d, *J* = 8.9 Hz, 2H, CH *Ar*), 4.61 (bs, 1H, NH), 3.95 (t, *J* = 6.1 Hz, 2H, CH_2O), 3.19 (q, *J* = 6.7 Hz, 2H, CH_2N), 1.80 (m, 2H, CH_2), 1.72 – 1.62 (m, 2H, CH_2), 1.45 (s, 9H, CH_3 *t-Bu*).
^{13}C NMR (101 MHz, $CDCl_3$) δ 156.98, 156.13, 147.18, 139.08, 132.33, 131.25, 127.55, 125.97, 113.33, 79.27, 67.44, 64.43, 40.44, 28.57, 27.02, 26.76.
HRMS (ESI): *m/z* 530.26670 $[M+Na]^+$ (calculated for $C_{34}H_{37}NNaO_3^+$ 530.26657).

4-(4-tritylphenoxy)butylamine (4g)

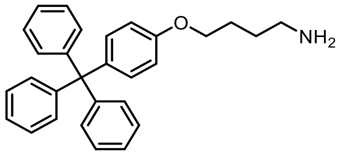

N-Boc-2-(4-tritylphenoxy)butylamine (903 mg, 1.78 mmol) was dissolved in CH_2Cl_2 (15 mL). TFA (3.0 mL) was added and the reaction mixture was stirred at r.t. for 3 h. All volatiles were removed under reduced pressure. The residue was dissolved in CH_2Cl_2 (150 mL) and the resulting solution was washed with 10% aq. NaOH (3×30 mL). The organic phase was washed with H_2O (40 mL) and dried over Na_2SO_4. The solvent was removed under reduced pressure and the crude product was purified by flash column chromatography on silica gel

(CH_2Cl_2 → CH_2Cl_2/MeOH = 95:5, the eluent contained 2% NEt_3) to afford 4-(4-tritylphenoxy)butylamine as white solid (704 mg, 1.73 mmol, 97%).

^{1}H NMR (400 MHz, $CDCl_3$) δ 7.30 – 7.16 (m, 15H, CH *Tr*), 7.11 (d, *J* = 8.9 Hz, 2H, CH *Ar*), 6.78 (d, *J* = 8.9 Hz, 2H, CH *Ar*), 3.97 (t, *J* = 6.4 Hz, 2H, CH_2O), 2.78 (t, *J* = 7.1 Hz, 2H, CH_2N), 1.82 (m, 2H, CH_2), 1.63 (m, 2H, CH_2), 1.24 (bs, 2H, NH_2).
^{13}C NMR (100 MHz, $CDCl_3$) δ 157.06, 147.16, 138.95, 132.29, 131.22, 127.51, 125.94, 113.31, 67.69, 64.40, 42.09, 30.52, 26.84.
HRMS (ESI): *m/z* 408.23204 $[M+H]^+$ (calculated for $C_{29}H_{30}NO^+$ 408.23219).

2.3.7 Synthesis of *O*-TBDMS-6-amino-1-hexanol (4h)

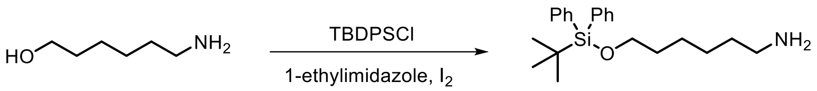

The procedure was adopted from ref. 11.[11]

6-Amino-1-hexanol (352 mg, 3.00 mmol, 1.0 eq.) and crystalline I_2 (2.28 g, 9.00 mmol, 3.0 eq.) were dissolved in anhydrous CH_2Cl_2 (9 mL). To a stirred solution, 1-ethylimidazole (0.87 mL, 9.00 mmol, 3.0 eq.) and *tert*-butyldiphenylsilyl chloride (0.96 mL, 3.30 mmol, 1.1 eq.) were added sequentially. The reaction mixture was stirred at room temperature under argon atmosphere for 1 h (TLC monitoring). The solvent was then removed under reduced pressure. Dark oily residue was dissolved in EtOAc (40 mL) and the solution was washed with 10% aq. $Na_2S_2O_3$ (4×15 mL, till I_2 is fully consumed) and sat. aq. Na_2CO_3 (2×10 mL). The organic phase was dried over anhydrous Na_2SO_4 and the solvent was removed under reduced pressure. The crude product was purified by flash column chromatography (CH_2Cl_2 → CH_2Cl_2/MeOH = 85:15) to afford *O*-TBDPS-6-amino-1-hexanol as yellowish oil (632 mg, 1.78 mmol, 59%).

^{1}H NMR (400 MHz, $CDCl_3$) δ 7.69 – 7.64 (m, 4H, CH *Ar*), 7.45 – 7.34 (m, 6H, CH *Ar*), 3.66 (t, *J* = 6.5 Hz, 2H, CH_2O), 2.66 (t, *J* = 7.0 Hz, 2H, CH_2N), 1.57 (m, *J* = 6.6 Hz, 2H, CH_2), 1.47 – 1.23 (m, 6H, CH_2), 1.05 (s, 11H, CH_3 *t-Bu* + NH_2).
^{13}C NMR (101 MHz, $CDCl_3$) δ 135.71, 134.29, 129.63, 127.71, 64.03, 42.39, 34.05, 32.68, 27.01, 26.77, 25.81, 19.36.
HRMS (ESI): *m/z* 356.24031 $[M+H]^+$ (calculated for $C_{22}H_{34}NOSi^+$ 356.24042).

2.4 Synthesis of threads for amidinium (pseudo/semi)rotaxanes

***N,N'*-dibenzylformamidinium tetraphenylborate** (S1)

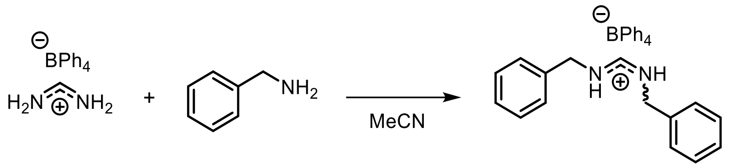

Benzylamine (56 µL, 0.51 mmol, 2.8 eq.) was added to a solution of **FA·BPh₄** (67 mg, 0.18 mmol, 1.0 eq.) in MeCN (2.5 mL). The reaction mixture was stirred under reflux for 30 min and MeCN was removed under reduced pressure. The residue was re-dissolved in MeCN (0.6 mL) and PhMe (6 mL) was added to the solution. The resulting mixture was kept overnight at +4 °C. Colorless needles were filtered off to afford the pure product (57 mg, 0.10 mmol, 57%).

^{1}**H NMR** (400 MHz, CD_3CN)* δ 8.02 (bs, 2H, NH *amidinium*), 7.85 (s, 1H, CH *amidinium*), 7.45 – 7.21 (m, 18H, CH *Ar: BPh₄ + amidinium Ph*), 6.99 (t, *J* = 7.6 Hz, 8H, CH *Ar BPh₄*), 6.84 (t, *J* = 7.2 Hz, 4H, CH *Ar BPh₄*), 4.57 (s, 2H, CH_2 *benzylic E,Z*), 4.53 (bs, 4H, CH_2 *benzylic E,E*), 4.46 (s, 2H, CH_2 *benzylic E,Z*).

*The NMR spectrum contains signals of both isomers of the product – *E,Z* and *E,E*. The integrals for each isomer specified in parentheses are treated independently.

^{13}**C NMR** (101 MHz, CD_3CN) δ 164.78 (q, $^{1}J_{B-C}$ = 49.3 Hz, C_{Ar} *BPh₄*), 155.95, 136.49 (C_{Ar} *BPh₄*), 134.63, 129.93, 129.83, 129.42, 129.35, 128.78, 128.56, 126.63 (q, *J* = 2.5 Hz, C_{Ar} *BPh₄*), 122.79 (C_{Ar} *BPh₄*), 51.63, 46.61.

HRMS (ESI): *m/z* 225.13921 $[M+H]^+$ (calculated for $C_{15}H_{17}N_2^+$ 225.13862).

N,N'-bis(3,5-di-*tert*-butylbenzyl)formamidinium tetraphenylborate (2a)

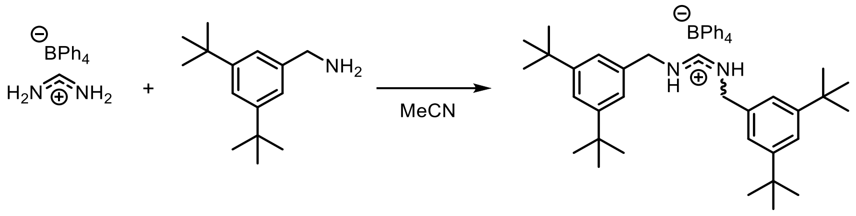

Solid **FA·BPh₄** (65 mg, 0.18 mmol, 1.0 eq.) was added to a solution of 3,5-di-*tert*-butylbenzylamine (118 mg, 0.54 mmol, 3.0 eq.) in MeCN (3.0 mL). The reaction mixture was stirred under reflux for 45 min and MeCN was then removed under reduced pressure. The residue was re-dissolved in MeCN (0.3 mL) and PhMe (3 mL) was added to the solution. The resulting mixture was kept overnight at –20 °C. Colorless needles were filtered off and subjected to the second crystallization (addition of PhMe to a $CHCl_3$ solution of the product) to afford an analytically pure *N,N'*-bis(3,5-di-*tert*-butylbenzyl)formamidinium tetraphenylborate (55 mg, 0.072 mmol, 40%).

^{1}**H NMR** (400 MHz, CD_3CN)* δ 7.90 (bs, 2H, NH *amidinium*), 7.74 (s, 1H, CH *amidinium E,E*), 7.64 (s, 1H, CH *amidinium E,Z*), 7.45 (m, 2H, CH *Ar E,E + E,Z*), 7.33 – 7.25 (m, 8H, CH *Ar BPh₄*), 7.18 (m, 4H, CH *Ar E,E*), 7.16 (d, *J* = 1.7 Hz, 2H, CH *Ar E,Z*), 7.14 (d, *J* = 1.7 Hz, 2H, CH *Ar E,Z*), 7.00 (t, *J* = 7.4 Hz, 8H, CH *Ar BPh₄*), 6.85 (t, *J* = 7.2 Hz, 4H, CH *Ar BPh₄*), 4.47 (s, 2H, CH_2 *benzylic E,Z*), 4.44 (s, 4H, CH_2 *benzylic E,E*), 4.34 (s, 2H, CH_2 *benzylic E,Z*), 1.32 (s, 36H, CH_3 *t-Bu E,E*), 1.31 (s, 18H, CH_3 *t-Bu E,Z*), 1.29 (s, 18H, CH_3 *t-Bu E,Z*).

*The NMR spectrum contains signals of both isomers of the product – *E,Z* and *E,E*. The integrals for each isomer specified in parentheses are treated independently.

^{13}**C NMR** (101 MHz, CD_3CN) δ 164.79 (q, $^{1}J_{B-C}$ = 49.3 Hz, C_{Ar} *BPh₄*), 155.50, 152.58, 136.70 (C_{Ar} *BPh₄*), 135.98, 135.70, 134.09, 126.63 (q, *J* = 2.7 Hz, C_{Ar} *BPh₄*), 123.53, 123.43, 123.40, 123.34, 123.07, 122.98, 122.79 (C_{Ar} *BPh₄*), 52.32, 52.01, 47.05, 35.58, 31.64, 31.61.

HRMS (ESI): *m/z* 449.38953 $[M+H]^+$ (calculated for $C_{31}H_{49}N_2^+$ 449.38903).

N-(3,5-di-*tert*-butylbenzyl)formamidinium tetraphenylborate (3a)

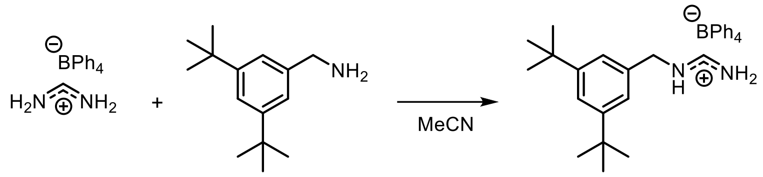

Solid **FA·BPh₄** (91 mg, 0.25 mmol, 1.0 eq.) and 3,5-di-*tert*-butylbenzylamine (121 mg, 0.55 mmol, 2.2 eq.) were dissolved in MeCN (2.0 mL). The mixture was stirred under reflux for 30 min and MeCN was then removed under reduced pressure. The residue was re-dissolved in MeCN (0.3 mL) and PhMe (4 mL) was added to the solution. The resulting mixture was kept overnight at –20 °C. Colorless needles were filtered off to afford the product of double exchange **2a** (98 mg). The mother liquor was concentrated under reduced pressure (to 1/3 of the initial volume), followed by addition of cyclohexane (2 mL) and the resulting solution

was kept overnight at −20 °C. A white solid was filtered off to afford *N*-(3,5-di-*tert*-butylbenzyl)formamidinium tetraphenylborate (61 mg, 0.11 mmol, 43%).

<u>*Note*</u>. Crystallization of pure **3a** can be tedious and poorly reproducible. Therefore, in an alternative procedure, **3a** can be obtained by reacting amine **4a** with excess of formamidinium chloride (3-5 eq.) in EtOH, followed by isolation using reversed-phase chromatography (e.g., semi-preparative HPLC).

^{1}H NMR (400 MHz, DMSO-d_6)* δ 9.91 (bs, 1H, NH *amidinium*), 9.14 (bs, 2H, NH *amidinium*), 8.12 (s, 1H, CH *amidinium E,E*), 7.98 (s, 1H, CH *amidinium E,Z*), 7.37 (s, 1H, CH *Ar E,E* + *E,Z*), 7.22 – 7.11 (m, 10H, CH *Ar BPh$_4$* & CH *Ar E,E* + *E,Z*), 6.92 (t, *J* = 7.2 Hz, 8H, CH *Ar BPh$_4$*), 6.78 (t, *J* = 7.0 Hz, 4H, CH *Ar BPh$_4$*), 4.49 (s, 2H, CH_2 *benzylic E,E*), 4.44 (s, 2H, CH_2 *benzylic E,Z*), 1.28 (s, 18H, CH_3 *t-Bu E,E* + *E,Z*).

*The NMR spectrum contains signals of both isomers of the product – *E,Z* and *E,E*. The integrals for each isomer specified in parentheses are treated independently.

^{13}C NMR (100 MHz, CD_3CN) δ 158.19*, 155.49*, 152.68, 136.72, 126.63, 123.51, 123.45, 123.10, 122.78, 52.03*, 46.67, 31.58, 27.61.

* Low intensity signals revealed from HSQC spectrum.

HRMS (ESI): *m/z* 247.21703 $[M+H]^+$ (calculated for $C_{16}H_{27}N_2^+$ 247.21688).

3. Optimization of the self-assembly of amidinium [2]rotaxanes

General synthesis procedure

If not otherwise specified, the rotaxane self-assemblies (Scheme S1) were performed in a closed screw-cap 1.5 mL HPLC vial at r.t. under continuous stirring. The vial was charged with a formamidinium salt (in this case **FA·BPh$_4$**) and **24C8**. The solvent (1/2 of the total volume) was added and the resulting suspension was stirred for 2-3 min. A solution of a primary amine (in this case **4a**) in the solvent (1/2 of the total volume) was then added and the reaction was stirred for several days. Typical solvent volumes were in the range 100–300 µL.

Reaction monitoring

The reaction progress was monitored by HPLC-MS (Figure S1). For HPLC-MS analysis, an aliquot of the reaction mixture (1 µL) was diluted with MeCN (1 mL) and the diluted solution (3-5 µL) was injected for HPLC analysis. The reaction efficiency was evaluated by calculating a *molar percentage* (***χ***) of the rotaxane (*rot*) in its mixture with the free thread (*thr*) after a certain period of time. The molar percentage ***χ*** was calculated based on the areas under chromatographic peaks corresponding to the rotaxane and the free thread: $\chi = \frac{c(rot)}{c(rot)+c(thr)} \times 100\% = \frac{A(rot)}{A(rot)+A(thr)} \times 100\%$, where *c* – molar concentration of the corresponding species, *A* – area under a chromatographic peak. For these largely *qualitative* optimization studies, it was assumed that molar absorptivities of the rotaxane and the free thread are roughly equal. This was later confirmed for the pair **1a** and **2a** (see Figure S38); the slopes of the corresponding calibration curves differ by factor 1.2 that reflects relative difference in molar absorptivities of **1a** and **2a**. However, among all synthesized rotaxanes (**1a** – **1j**), we expect the pair **1a**/**2a** to have the largest difference in molar absorptivities due to very close proximity of the crown ether and the chromophores (stoppers). For selected self-assemblies quantitative HPLC ("HPLC yields") was performed, please also refer to Sections 4 and 7.

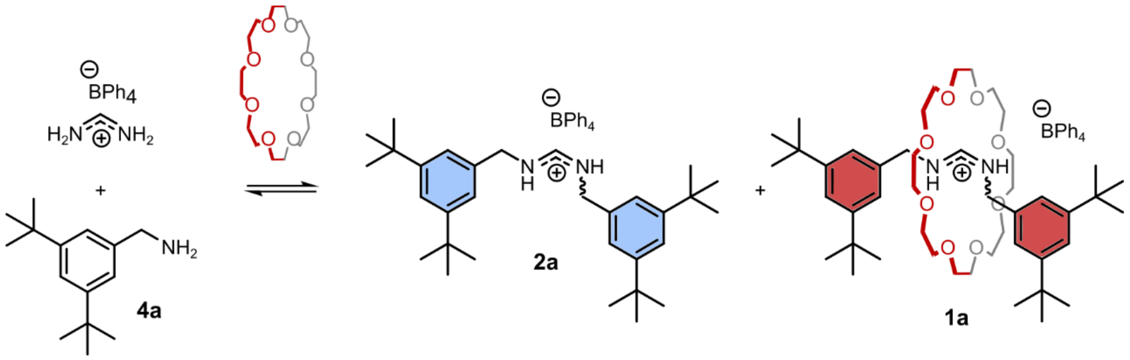

Scheme S1. Self-assembly of rotaxane **1a** used for optimization of the reaction conditions.

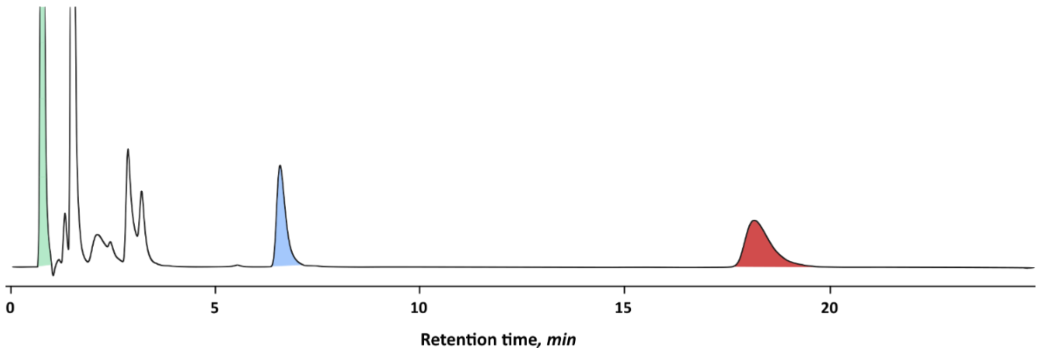

Figure S1. Representative HPLC chromatogram of a reaction mixture of the rotaxane self-assembly by amidinium exchange. This particular HPLC trace corresponds to the rotaxane self-assembly in PhMe at 80 °C after 46 h (67µmol **FA·BPh$_4$**, 140 µmol 3,5-di-*tert*-butylbenzylamine and 230 µmol **24C8** in 290 µL PhMe). Red peak: the rotaxane. Blue peak: the free thread. Greenish peak: BPh_4^-.

Table S1. Influence of the solvent on the amidinium rotaxane molar percentage χ. Reaction conditions: 1.0 eq. **FA·BPh$_4$** (0.2 M), 2.2 eq. amine, 2.0 eq. **24C8**, r.t. Molar percentage χ of rotaxane **1a** was determined on the 6th day of the reaction. Dielectric constant ε, Z, $E_T(30)$ and π* values are different measures of solvent polarity. The Donor numbers (DN) characterize ability of a solvent to act as a Lewis base. Parameters α and β are a measure of solvent's ability to act as a hydrogen bond donor or acceptor to a solute respectively. Values of π*, α and β were taken from Ref. 12;[12] ε – ref. 13;[13] DN – ref. 14;[14] ET(30) – ref. 15.[15]

Entry	Solvent	ε	$E_T(30)$	π^*	Donor number (*DN*)	α	β	χ, %
1	PhMe	2.4	34	0.54	0.1	0.00	0.11	10
2	$CHCl_3$	4.8	39	0.58	4.0	0.44	0.00	10
3	THF	7.5	37	0.58	20	0.00	0.55	27
4	Pyridine	12	40	0.87	33	0.00	0.64	13†
5	MeCN	37	46	0.75	14	0.19	0.31	27‡

†3.0 eq. amine was used.

‡After 40 days, χ reached 46% and HPLC yield was 29% (determined using 1,2,4,5-tetramethylbenzene as an internal standard).

Table S2. Influence of the 24C8 amount on the amidinium rotaxane molar percentage χ. Reaction conditions: 1.0 eq. **FA·BPh$_4$** (0.2 M), 2.2 eq. amine, solvent – THF, r.t. Molar percentage χ of rotaxane **1a** was determined on the 6th day of the reaction.

Entry	Amount of 24C8, eq.	χ, %
1	0.5	6
2	1.0	20
3	1.5	26
4	2.0	27
5	4.0*	26**
6	7.0*	22

*Due to large amount of **24C8**, concentration of **FA·BPh$_4$** was ~0.15 M (solvent volume in all cases was kept constant).

**After 51 days, χ reached 54% and HPLC yield was 24% (determined using 1,2,4,5-tetramethylbenzene as an internal standard).

Table S3. Influence of the amine amount on the amidinium rotaxane molar percentage χ. Reaction conditions: 1.0 eq. **FA·BPh$_4$** (0.2 M), 2.0 eq. **24C8**, solvent – THF, r.t. Molar percentage of the rotaxane χ was determined on the 6th day of the reaction.

Entry	Amount of the amine, eq.	χ, %
1	1.5	18
2	2.2	27
3	3.0	30*
4	7.0	14

* After 28 days, χ reached 49% and HPLC yield was 36% (determined using 1,2,4,5-tetramethylbenzene as an internal standard).

Table S4. Influence of the concentration on the amidinium rotaxane molar percentage χ. Reaction conditions: 1.0 eq. **FA·BPh$_4$**, 2.0 eq. amine, 3.0 eq. **24C8**, solvent – THF, r.t. Molar percentage of the rotaxane χ was determined on the 6th day of the reaction.

Entry	Concentration of FA·BPh$_4$, M	χ, %
1	0.03	< 5

Table S5. Influence of bases/nucleophilic catalysts on the amidinium rotaxane molar percentage χ. Reaction conditions: 1.0 eq. **FA·BPh₄** (0.2 M), 3.0 eq. amine, 2.0 eq. **24C8**. Molar fraction of the percentage χ was determined on the 6th day of the reaction.

Entry	Additive	Solvent	χ, %
1	DMAP (0.1 eq.)	PhMe	11
2	DMAP (0.1 eq.)	MeCN	24
3	DMAP (0.5 eq.)	THF	26
4	DMAP (1.0 eq.)	THF	25
5	DIPEA (0.5 eq.)	THF	21
6	DIPEA (1.0 eq.)	THF	22
7	DBU (1.0 eq.)	THF	2

Table S6. Influence of temperature on the amidinium rotaxane molar percentage χ. Reaction conditions: 1.0 eq. **FA·BPh₄** (0.2 M), 3.0 eq. amine. All reactions were performed open-air.

Entry	Solvent	Reaction temperature, °C	Amount of 24C8, eq.	Time, hours	χ, %
1	THF	55	2.0	24	22
2	MeCN	80	2.0	6	4
3	PhMe	90	2.0	3	17
4	PhMe	75	2.0	45	53*
5	PhMe	80	1.5	48	26
6	PhMe	80	1.0	48	14
7	PhMe	80	0.5	48	7
8	n-heptane	90	2.0	6	18

*2.0 eq. amine was used. After 3 days, χ reached 61%. Similar result could be achieved in THF/PhMe mixture (6:4 v/v) at 65 °C (see kinetic studies in Section 7.2.1).

4. Synthesis of the amidinium [2]rotaxanes

General procedure

Method A. A 1.5 mL screw-cap HPLC vial was charged with **24C8** (2.0 eq.) and **FA·BPh$_4$** (1.0 eq.). A solvent (1/2 of the total volume*) was added and the resulting suspension was stirred for 2 min. A solution of a primary amine (2.0 – 3.0 eq.) in the solvent (1/2 of the total volume) was added (alternatively – solid amine was first added followed by the solvent) and the reaction mixture was stirred at 45–75 °C for 1–5 days (a needle was inserted into HPLC vial's cap in order to release NH_3 that was formed during the reaction). The reaction mixture was diluted with THF (the same volume as the original volume of the reaction solvent) and NH_2OH (50% aqueous solution, 1.0 eq.) was added. The mixture was stirred for 10 min at r.t. All volatiles were removed under reduced pressure and the rotaxane was isolated by preparative TLC or semi-preparative HPLC (see the conditions in General methods, Section 1). In case of HPLC purification, collected fractions containing the target rotaxane were concentrated under reduced pressure. The obtained residue was partitioned in CH_2Cl_2/H_2O mixture (3 mL / 1 mL). The layers were separated and the organic layer was washed with H_2O (1×0.5 mL) and passed through a Pasteur pipette filled with anhydrous Na_2SO_4. The solvent was removed under reduced pressure to afford a formate salt of the rotaxane.

Method B. In a 1.5 mL screw-cap HPLC vial, a solution (0.2 M) of a primary amine (2.0 – 2.5 eq.) in THF was prepared. **FA·BPh$_4$** (1.0 eq.) was added, followed by 3 Å molecular sieves, and the resulting mixture was stirred in a closed vial at r.t. for 2 h. All volatiles were removed under reduced pressure and the residue was dissolved in PhMe (2/5 of the total volume*). **24C8** was added, followed by NH_3 (0.5 M solution in THF, 1.5 eq.). The reaction mixture was stirred in a tightly closed HPLC or screw-cap vial at 70 °C for 5 days. Further isolation of the product was the same as in Method A (including addition of NH_2OH).

*Final total concentration of formamidinium species was 0.2 M.

Anion exchange with BArF$^-$

<u>*Preparation of the anion exchange resin in BArF form.*</u> Lewatit® MonoPlus M500 anion exchange resin (Cl^- form, 5 g) was washed sequentially with H_2O, aqueous solution of NH_4PF_6 (10 g in 100 mL H_2O), H_2O, H_2O/MeCN mixture (1:1 by volume), solution of NaBArF (1.0 g) in H_2O/MeCN (50 mL), H_2O/MeCN mixture (1:1 by volume). Afterwards, the resin was washed with MeCN and blow-dried with air.

<u>*Anion exchange*</u>. The rotaxane (anion: HCO_2^- or BPh_4^-) solution (5 – 10 mM) in MeCN was stirred for 10 min at r.t. with Lewatit® resin (BArF form). The solution was transferred to a new portion of the resin and was stirred again for 10 min. The solution was filtered and the solvent was removed under reduced pressure to afford the amidinium rotaxane as BArF salt.

Rotaxane 1a

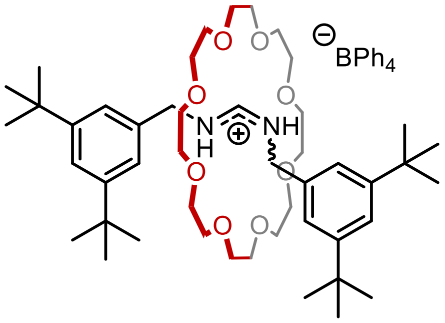

Rotaxane **1a** (anion: BPh_4^-; 49 mg, 44 µmol, 36%) was obtained according to Method A. Reaction conditions: 2.0 eq. amine **4a**, solvent – PhMe, 75 °C, 3 days. Isolation: preparative TLC (silica gel, CH_2Cl_2/MeOH = 99:1).

For NMR analysis and further experiments, **1a** was converted to BArF form.

^{1}H NMR (500 MHz, $CDCl_3$)* δ 8.41 (t, *J* = 13.5 Hz, 1H, CH *amidinium E,E*), 8.34 (m, 1H, NH), 8.05 (bs, 1H, NH), 7.71 – 7.69 (m, 8H, CH *BArF*), 7.67 (m, 1H, CH *amidinium E,Z*), 7.52 (s, 4H, CH *BArF*), 7.46 (t, *J* = 1.8 Hz, 1H, CH *Ar E,Z*), 7.41 (t, *J* = 1.8 Hz, 1H, CH *Ar E,E*), 7.40 (t, *J* = 1.8 Hz, 1H, CH *Ar E,Z*), 7.17 (d, *J* = 1.8 Hz, 2H, CH *Ar E,Z*), 7.14 (d, *J* = 1.8 Hz, 2H, CH *Ar E,Z*), 7.11 (d, *J* = 1.8 Hz, 1H, CH *Ar E,E*), 4.54 (d, 2H, CH_2 *benzylic E,E*) 4.53 (d, *J* = 5.4 Hz, 2H, CH_2 *benzylic E,Z*), 4.44 (d, *J* = 5.5 Hz, 2H, CH_2 *benzylic E,Z*), 3.48 (s, 32H, CH_2 *24C8 E,E*), 3.42 (s, 32H, CH_2 *24C8 E,Z*), 1.33 (s, 18H, CH_3 *t-Bu E,Z*), 1.31 (s, 36H, CH_3 *t-Bu E,E*), 1.31 (s, 18H, CH_3 *t-Bu E,Z*).

*The NMR spectrum contains signals from both isomers of the rotaxane – *E,Z* and *E,E*. The integrals for each isomer specified in parentheses are treated independently.

^{13}C NMR (126 MHz, $CDCl_3$) δ 161.85 (q, $^1J_{B-C}$ = 49.9 Hz, C_{Ar} *BArF*), 159.18, 153.30, 151.99, 151.92, 151.66, 134.95 (C_{Ar} *BArF)*, 134.14, 133.92, 129.04 (q, $^2J_{C-F}$ = 31.5 Hz, C_{Ar} *BArF)*, 124.71 (q, $^1J_{C-F}$ = 273 Hz, CF_3 *BArF*), 123.65, 123.38, 122.98, 122.61, 122.42, 122.06, 117.59 (m, C_{Ar} *BArF*), 70.72, 70.63, 53.02, 51.72, 46.61, 35.05, 35.04, 31.52, 31.50.

HRMS (ESI): *m/z* 801.60274 [M+H]$^+$ (calculated for $C_{47}H_{81}N_2O_8^+$ 801.59874).

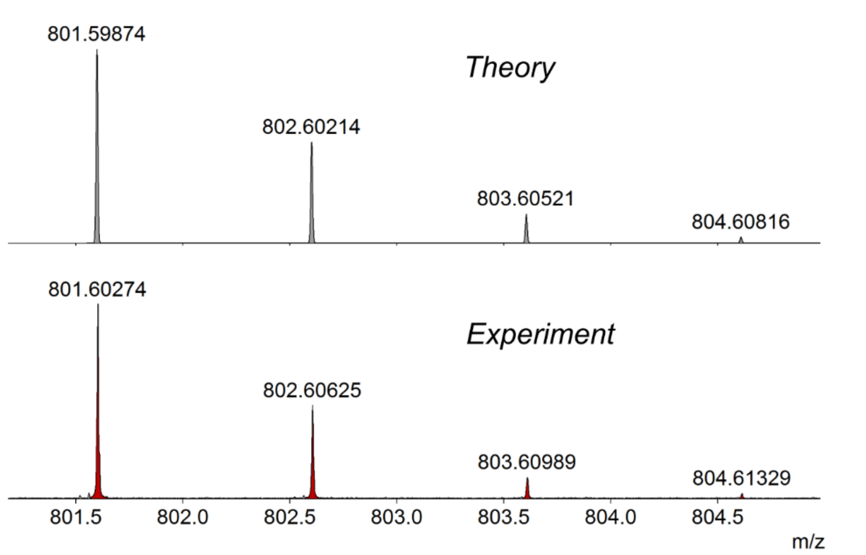

Figure S2. HRMS peaks of rotaxane **1a**: simulated (top) and experimental (bottom) isotopic patterns.

Rotaxane 1b

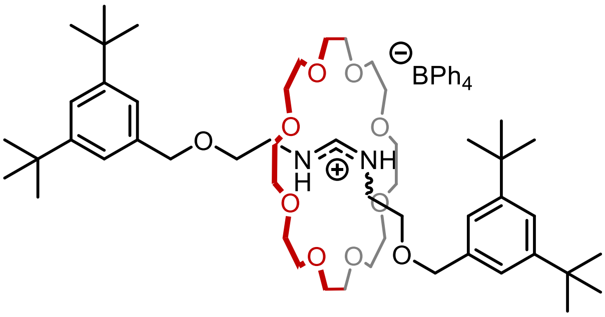

Rotaxane **1b** (anion: BPh_4^-; 38 mg, 31 µmol, 21%) was obtained according to Method A. Reaction conditions: 3.0 eq. amine **4b**, solvent – PhMe, 75 °C, 5 days. Isolation: preparative TLC (silica gel, CH_2Cl_2/MeOH = 97:3). For NMR analysis and further experiments, **1b** was converted to BArF form.

^{1}H NMR (500 MHz, CD_2Cl_2)* δ 8.09 (t, J = 13.4 Hz, 1H, CH *amidinium E,E*), 8.00 – 7.86 (m, 2H, NH), 7.75 – 7.70 (m, 8H, CH *BArF*), 7.70 – 7.59 (m, 1H, CH *amidinium E,Z* + NH?), 7.56 (m, 4H, CH *BArF*), 7.39 (t, J = 1.5 Hz, 2H, CH *Ar E,Z* + *E,E*), 7.19 – 7.12 (m, 4H, CH *Ar E,Z* + *E,E*), 4.511 (s, 2H, CH_2 *benzylic E,Z*) 4.506 (s, 2H, CH_2 *benzylic E,Z*), 4.501 (s, 2H, CH_2 *benzylic E,E*), 3.70 – 3.55 (m, 8H, CH_2), 3.54 – 3.46 (m, 32H, CH_2 *24C8 E,Z* + *E,E*), 1.314 (s, 18H, CH_3 *t-Bu E,Z*), 1.313 (s, 18H, CH_3 *t-Bu E,Z*), 1.307 (s, 18H, CH_3 *t-Bu E,E*).

*The NMR spectrum contains signals from both isomers of the rotaxane – *E,Z* and *E,E*. The integrals for each isomer specified in parentheses are treated independently.

^{13}C NMR (126 MHz, CD_2Cl_2) δ 162.17 (q, $^1J_{B-C}$ = 49.9 Hz, C_{Ar} *BArF*), 159.78, 155.58, 151.51, 151.48, 151.46, 137.22, 137.11, 135.22 (C_{Ar} *BArF)*, 129.30 (q, $^2J_{C-F}$ = 31.5 Hz, C_{Ar} *BArF)*, 125.01 (q, $^1J_{C-F}$ = 272 Hz, CF_3 *BArF*), 122.66, 122.54, 122.46, 117.88 (m, C_{Ar} *BArF*), 74.66, 74.57, 74.51, 71.16, 71.07, 69.05, 68.89, 67.45, 47.86, 47.26, 41.91, 35.12, 31.62.

HRMS (ESI): *m/z* 889.64873 $[M+H]^+$ (calculated for $C_{51}H_{89}N_2O_{10}^+$ 889.65117).

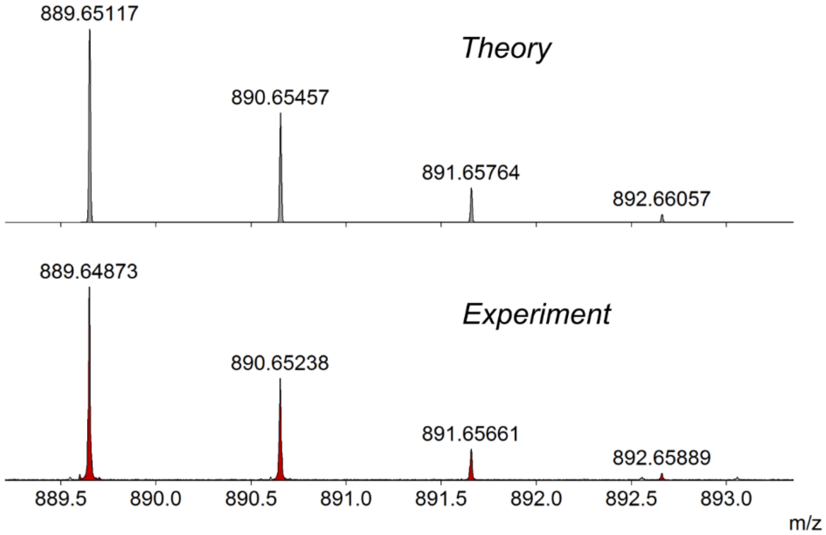

Figure S3. HRMS peaks of rotaxane **1b**: simulated (top) and experimental (bottom) isotopic patterns.

Rotaxane 1c

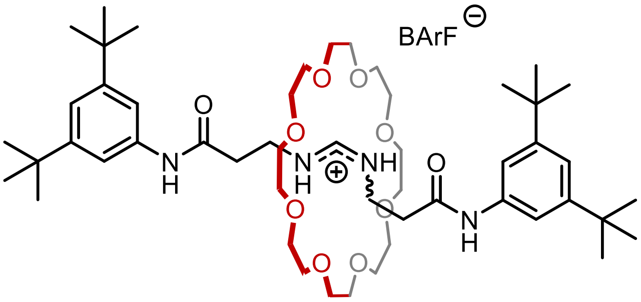

Rotaxane **1c** (anion: BArF⁻; 20 mg, 11 µmol, 16%) was obtained according to modified Method B. In a 1.5 mL screw-cap HPLC vial, a solution of amine **4c** (0.14 mmol, 2.0 eq.) in THF (300 µL) was prepared. **FA·BPh$_4$** (0.070 mmol, 1.0 eq.) was added and the resulting mixture was stirred in a closed vial at r.t. for 30 min. **24C8** (0.14 mmol, 2.0 eq.) was then added and the reaction mixture was stirred at 45 °C for 2 days (a needle was inserted into HPLC vial's cap). THF (300 µL) was added and the reaction mixture was further stirred in a closed vial at 45 °C for 5 days. Further isolation of the product was the same as in Method A (including addition of NH_2OH).

^{1}H NMR (400 MHz, $CDCl_3$)* δ 8.25 (m, 1H, NH *amide*), 8.09 – 7.76 (m, 2H, NH), 7.71 (m, 8H, CH *BArF*), 7.67 (m, 1H, CH *amidinium E,Z* + *E,E*), 7.53 (bs, 5H, CH *BArF* + NH), 7.36 (m, 4H, CH *Ar E,Z*), 7.34 (d, J = 1.7 Hz, 4H, CH *Ar E,E*), 7.20 (m, 4H, CH *Ar E,Z* + *E,E*), 3.70 (m, 4H, CH_2N *E,Z* + *E,E*) 3.55 (s, 32H, CH_2 *24C8 E,E*), 3.53 (s, 32H, CH_2 *24C8 E,Z*) 2.69 (m, 4H, CH_2 *CH_2CO E,Z* + *E,E*), 1.30 (m, 36H, CH_3 *t-Bu E,Z* + *E,E*).

*The NMR spectrum contains signals from both isomers of the rotaxane – *E,Z* and *E,E*. The integrals for each isomer specified in parentheses are treated independently.

^{13}C NMR (126 MHz, $CDCl_3$) δ 168.62, 167.76, 161.83 (q, $^1J_{B\text{–}C}$ = 49.9 Hz, C_{Ar} *BArF*), 158.71, 155.19, 152.09, 152.00, 151.92, 137.40, 137.10, 137.02, 134.95 (C_{Ar} *BArF)*, 129.06 (q, $^2J_{C\text{–}F}$ = 31.7 Hz, C_{Ar} *BArF)*, 124.71 (q, $^1J_{C\text{–}F}$ = 273 Hz, CF_3 *BArF*), 119.00, 118.93, 118.76, 117.59 (m, C_{Ar} *BArF*), 114.30, 114.26, 114.19, 70.93, 70.90, 43.15, 43.05, 38.23, 36.70, 36.41, 35.26, 35.09, 31.49.

HRMS (ESI): *m/z* 915.64020 [M+H]$^+$ (calculated for $C_{51}H_{87}N_4O_{10}^+$ 915.64167).

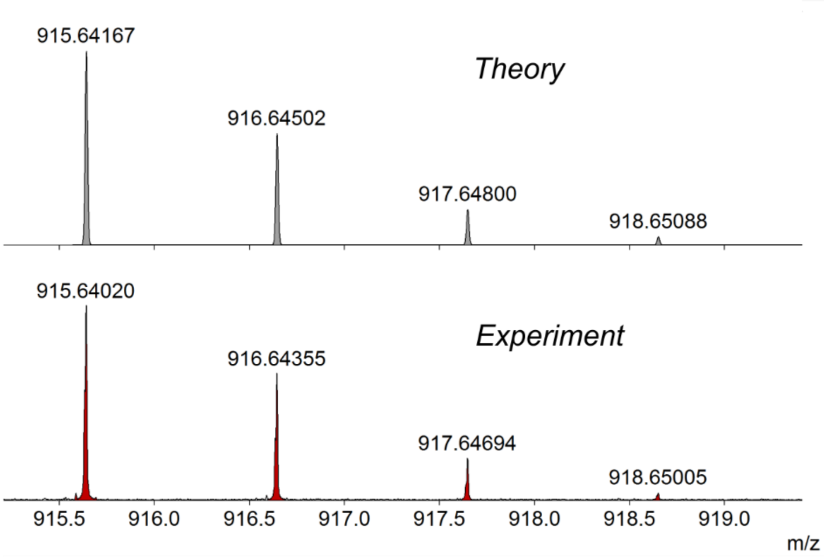

Figure S4. HRMS peaks of rotaxane **1c**: simulated (top) and experimental (bottom) isotopic patterns.

Rotaxane 1d

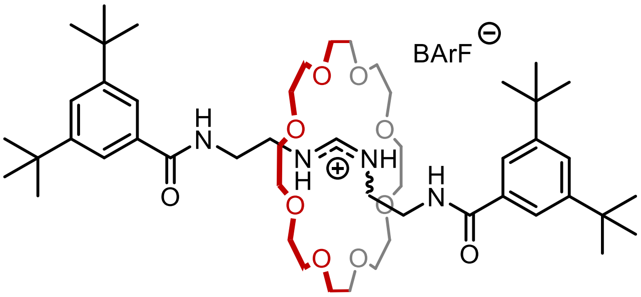

Rotaxane **1d** (anion: BArF⁻; 24 mg, 13 µmol, 15%) was obtained according to Method B. Reaction conditions: 2.5 eq. amine **4d**, 70 °C, 5 days. Isolation: semi-preparative HPLC.

^{1}H NMR (400 MHz, $CDCl_3$)* δ 8.39 (m, 1H, NH *amide E,Z* or *E,E*), 8.07 (m, 1H, NH *amide*? *E,E* or *E,Z*), 7.84 (m, 1H, NH?), 7.70 (m, 9H, CH *BArF* + NH?), 7.61 – 7.49 (m, 11H, CH Ar + CH *BArF* + CH *amidinium* + NH *amidinium*), 7.17 (t, *J* = 5.7 Hz, 1H, CH *Ar E,Z*), 7.10 (t, *J* = 5.6 Hz, 2H, CH *Ar E,E*), 7.06 (t, *J* = 5.8 Hz, 1H), 3.75 – 3.53 (m, 8H, CH_2 *E,Z* + *E,E*) 3.48 (s, 32H, CH_2 *24C8 E,E*), 3.44 (bs, 32H, CH_2 *24C8 E,Z*), 1.31 (m, 36H, CH_3 *t-Bu E,Z* + *E,E*).

*The NMR spectrum contains signals from both isomers of the rotaxane – *E,Z* and *E,E*. The integrals for each isomer specified in parentheses are treated independently.

^{13}C NMR (126 MHz, $CDCl_3$) δ 170.46, 170.18, 161.83 (q, $^1J_{B-C}$ = 49.9 Hz, C_{Ar} *BArF*), 159.56, 155.07, 151.72, 151.64, 151.60, 134.93 (C_{Ar} *BArF)*, 134.12, 133.45, 133.33, 129.03 (q, $^2J_{C-F}$ = 31.5 Hz, C_{Ar} *BArF)*, 126.53, 126.44, 126.32, 124.70 (q, $^1J_{C-F}$ = 273 Hz, CF_3 *BArF*), 121.59, 121.38, 121.31, 117.61 (m, C_{Ar} *BArF*), 70.71, 70.64, 47.73, 47.63, 42.73, 40.23, 40.21, 38.13, 35.10, 31.44, 31.42.

HRMS (ESI): *m/z* 915.63920 $[M+H]^+$ (calculated for $C_{51}H_{87}N_4O_{10}^+$ 915.64167).

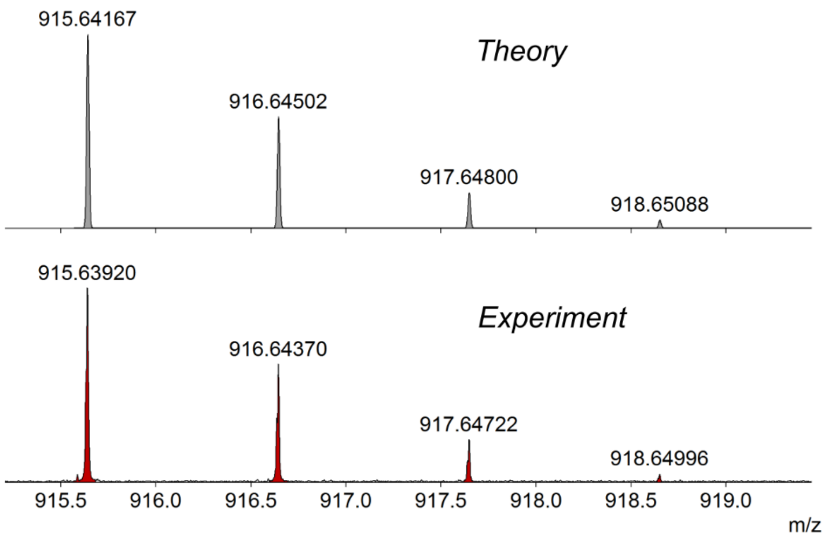

Figure S5. HRMS peaks of rotaxane **1d**: simulated (top) and experimental (bottom) isotopic patterns.

Rotaxane 1e

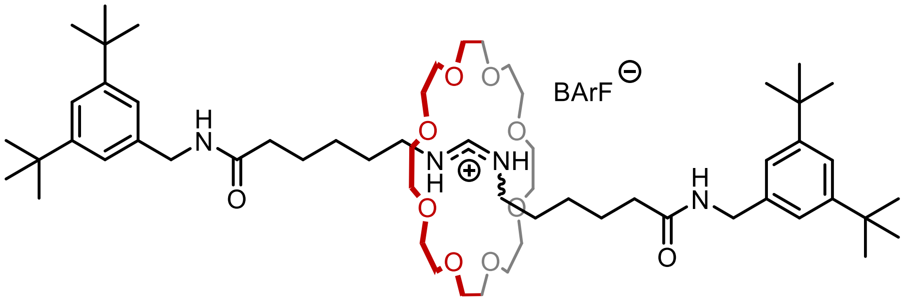

Rotaxane **1e** (anion: BArF$^-$; 27 mg, 14 µmol, 15%) was obtained according to Method A. Reaction conditions: 3.0 eq. amine **4e**, solvent – PhMe, 70 °C, 5 days. Isolation: semi-preparative HPLC.

^{1}H NMR (400 MHz, $CDCl_3$)* δ 8.05 (t, *J* = 13.5 Hz, 1H, CH *amidinium E,E*), 7.70 (m, 10H, CH *BArF* + CH *amidinium* + NH *amidinium*), 7.52 (bs, 4H, CH *BArF*), 7.41 – 7.31 (m, 3H, CH *Ar* + NH?), 7.13 – 7.03 (m, 4H, CH *Ar*), 5.89 (bs, 2H, NH *amide E,E*), 5.68 (m, 2H, NH *amide E,Z*), 4.45 – 4.34 (m, 4H, CH_2 *benzylic*), 3.69 – 3.42 (m, 32H, CH_2 *24C8*), 3.27 (m, 4H, CH_2N), 2.22 (m, 4H, CH_2CO), 1.70 (m, 4H, CH_2), 1.62 (m, 4H, CH_2), 1.42 (m, 4H, CH_2), 1.31 (m, 36H, CH_3 *t-Bu E,Z* + *E,E*).

*The NMR spectrum contains signals from both isomers of the rotaxane – *E,Z* and *E,E*. The integrals for each isomer specified in parentheses are treated independently.

^{13}C NMR (126 MHz, $CDCl_3$) δ 172.23, 161.83 (q, $^{1}J_{B\text{–}C}$ = 49.9 Hz, C_{Ar} *BArF*), 153.85, 151.61, 137.21, 134.93 (C_{Ar} *BArF*), 129.05 (q, $^{2}J_{C\text{–}F}$ = 31.5 Hz, C_{Ar} *BArF*), 124.69 (q, $^{1}J_{C\text{–}F}$ = 273 Hz, CF_3 *BArF*), 122.34, 122.25, 122.02, 117.60 (m, C_{Ar} *BArF*), 70.95, 70.81, 70.04, 47.88, 46.84, 44.54, 41.44, 36.32, 35.00, 31.55, 31.44, 30.00, 29.94, 27.64, 26.72, 26.25, 25.24, 25.20.

HRMS (ESI): *m/z* 1027.76378 [M+H]$^+$ (calculated for $C_{59}H_{103}N_4O_{10}^+$ 1027.76687).

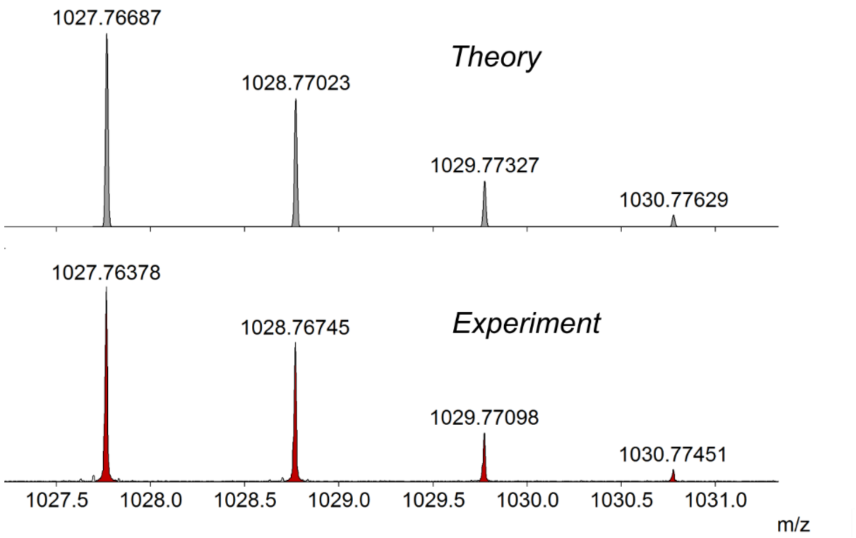

Figure S6. HRMS peaks of rotaxane **1e**: simulated (top) and experimental (bottom) isotopic patterns.

Rotaxane 1f

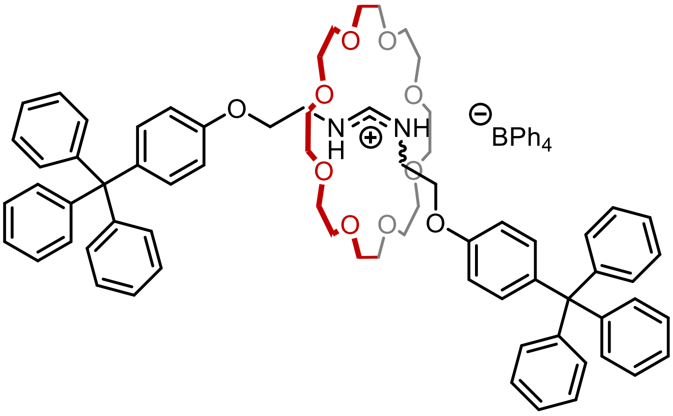

Rotaxane **1f** (anion: BPh_4^-; 20 mg, 14 µmol, 20%) was obtained according to Method A. Reaction conditions: 2.0 eq. amine **4f**, solvent – THF, 45 °C, 4 days. Isolation: preparative TLC (silica gel, CH_2Cl_2/MeOH = 97:3). For NMR analysis and further experiments, **1f** was converted to BArF form.

^{1}H NMR (400 MHz, $CDCl_3$)* δ 8.26 – 8.15 (m, 2H, CH *amidinium E,E* + NH *amidinium*), 8.01 (m, 1H, NH), 7.89 (m, 1H, NH), 7.74 – 7.63 (m, 9H, CH *BArF* + CH *amidinium E,Z*), 7.52 (bs, 4H, CH *BArF*), 7.32 – 7.11 (m, 34H, CH *Ar*), 6.75 (t, *J* = 8.6 Hz, 4H, CH *Ar*), 4.16 (t, *J* = 5.2 Hz, 2H, CH_2O *E,Z*), 4.07 (m: 2H, CH_2O *E,Z* + 4H, CH_2O *E,E*), 3.74 (m: 2H, CH_2N *E,Z* + 4H, CH_2N *E,E*), 3.66 (q, *J* = 5.3 Hz, 2H, CH_2N *E,Z*), 3.47 (s, 32H, CH_2 *24C8 E,E*), 3.45 (s, 32H, CH_2 *24C8 E,Z*).

*The NMR spectrum contains signals from both isomers of the rotaxane – *E,Z* and *E,E*. The integrals for each isomer specified in parentheses are treated independently.

^{13}C NMR (126 MHz, $CDCl_3$) δ 161.83 (q, $^1J_{B-C}$ = 49.9 Hz, C_{Ar} *BArF*), 159.97, 156.16, 156.04, 155.82, 155.66, 146.98, 146.94, 140.55, 140.50, 140.36, 134.94 (C_{Ar} *BArF*), 132.60, 132.54, 131.13, 129.05 (q, $^2J_{C-F}$ = 31.5 Hz, C_{Ar} *BArF*), 128.08, 127.66, 127.43, 126.15, 124.70 (q, $^1J_{C-F}$ = 273 Hz, CF_3 *BArF*), 117.61 (m, C_{Ar} *BArF*), 113.45, 113.30, 113.23, 70.79, 70.67, 66.42, 65.87, 64.78, 64.51, 46.87, 46.58, 41.30.

HRMS (ESI): *m/z* 1121.58650 [M+H]$^+$ (calculated for $C_{71}H_{81}N_2O_{10}^+$ 1121.58857).

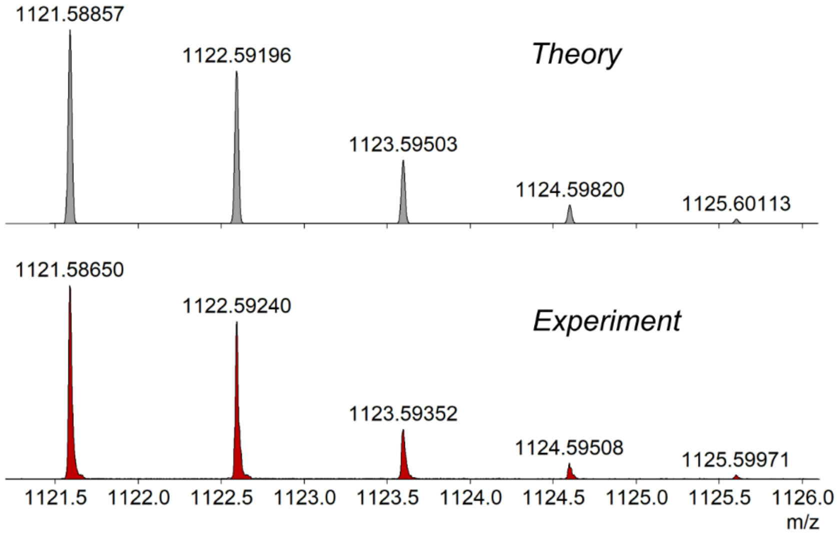

Figure S7. HRMS peaks of rotaxane **1f**: simulated (top) and experimental (bottom) isotopic patterns.

Rotaxane 1g

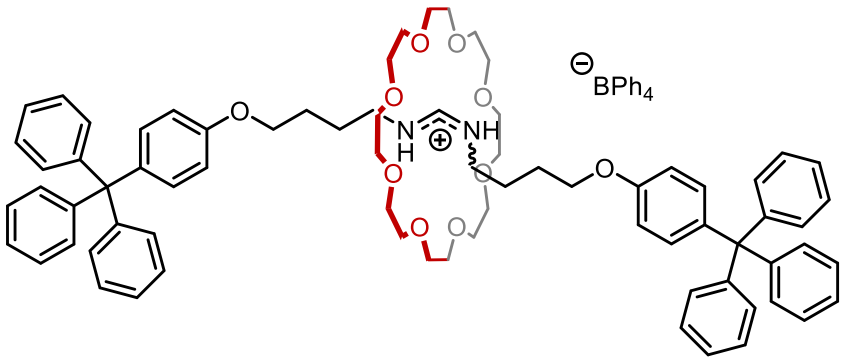

Rotaxane **1g** (HPLC yield: 42%) was synthesized according to Method A. Reaction conditions: 3.0 eq. amine **4g**, solvent – THF, 50 °C, 3 days. HPLC yield was calculated using 1,2,4,5-tetramethylbenzene as an internal standard and a corresponding calibration curve for thread **2g** (it was assumed that molar absorptivities of the rotaxane and the free thread are equal; see Section 3, "Reaction monitoring").

HRMS (ESI): *m/z* 1177.65000 $[M+H]^+$ (calculated for $C_{75}H_{89}N_2O_{10}^+$ 1177.65117).

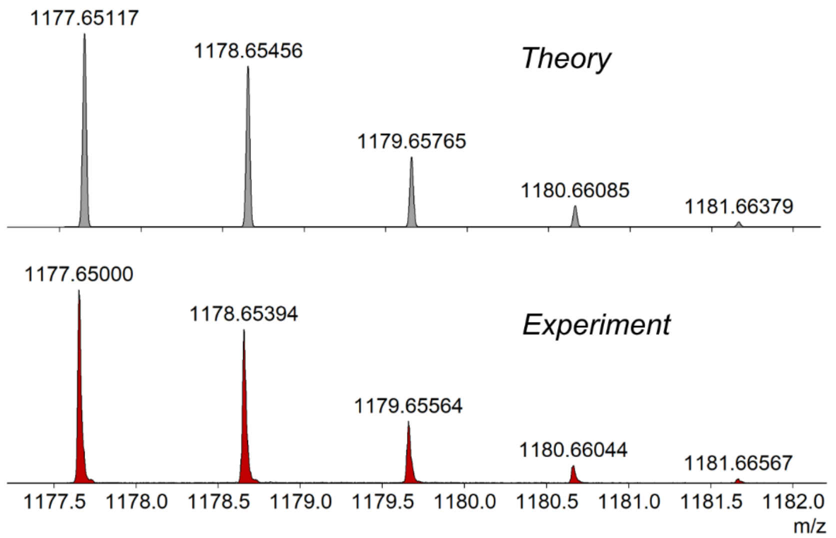

Figure S8. HRMS peaks of rotaxane **1g**: simulated (top) and experimental (bottom) isotopic patterns.

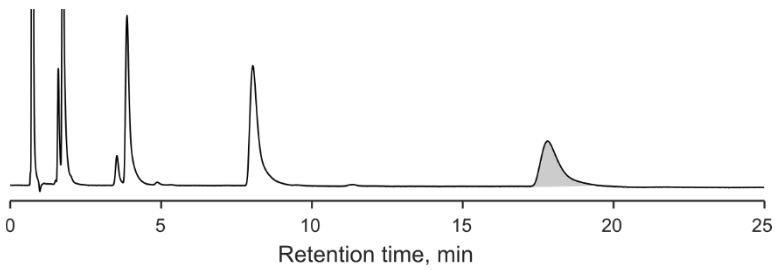

Figure S9. HPLC chromatogram of the reaction mixture of **1g** synthesis (3rd day). Shaded peak (grey) corresponds to rotaxane **1g**.

Rotaxane 1h

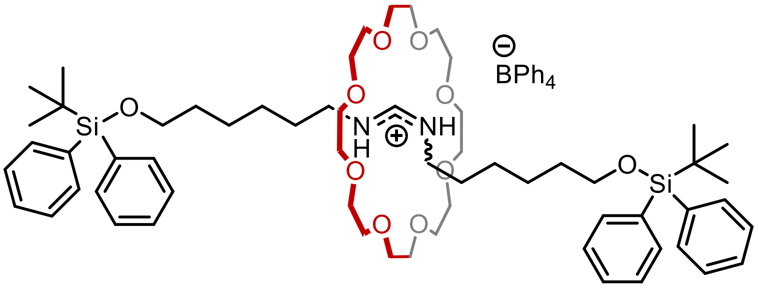

Rotaxane **1h** (HPLC yield: 26%) was synthesized according to Method A. Reaction conditions: 3.0 eq. amine **4h**, solvent – PhMe, 75 °C, 3 days. HPLC yield was calculated using 1,2,4,5-tetramethylbenzene as an internal standard and a corresponding calibration curve for thread **2h** (it was assumed that molar absorptivities of the rotaxane and the free thread are equal; see Section 3, “Reaction monitoring”).

HRMS (ESI): *m/z* 1073.65736 $[M+H]^+$ (calculated for $C_{61}H_{97}N_2O_{10}Si_2^+$ 1073.66763).

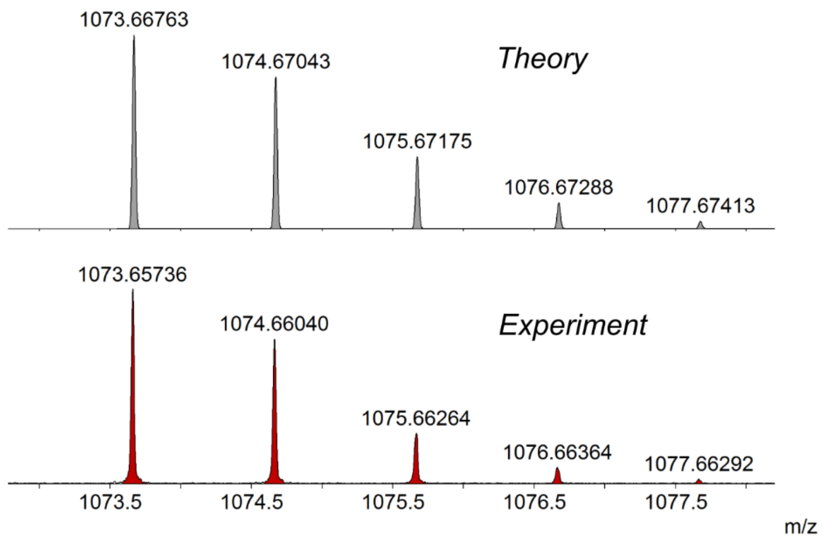

Figure S10. HRMS peaks of rotaxane **1h**: simulated (top) and experimental (bottom) isotopic patterns.

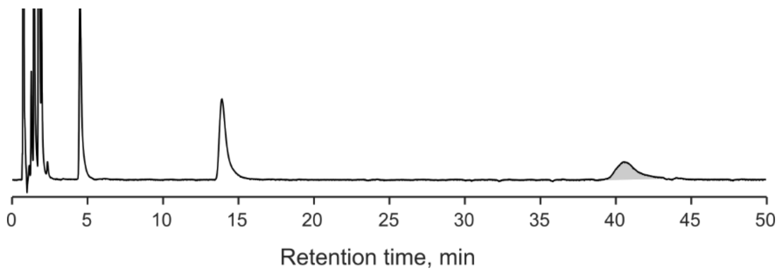

Figure S11. HPLC chromatogram of the reaction mixture of **1h** synthesis (3rd day). Shaded peak (grey) corresponds to rotaxane **1h**.

Rotaxane 1i

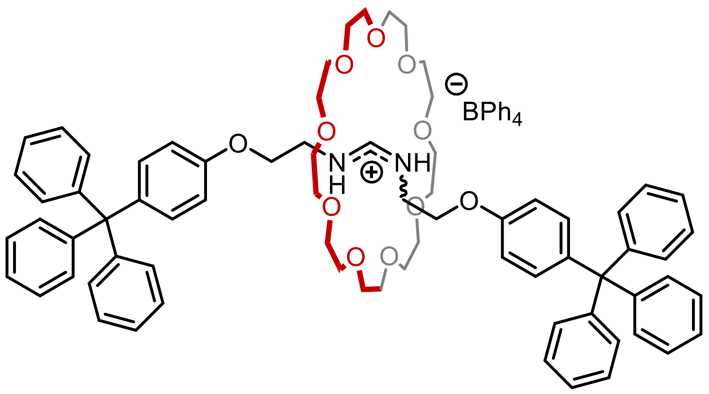

Rotaxane **1i** (HPLC yield: 10%) was synthesized according to Method A. Reaction conditions: 3.0 eq. amine **4f**, solvent – THF, 50 °C, 3 days. HPLC yield was calculated using 1,2,4,5-tetramethylbenzene as an internal standard and a corresponding calibration curve for thread **2f** (it was assumed that molar absorptivities of the rotaxane and the free thread are equal; see Section 3, "Reaction monitoring").

HRMS (ESI): *m/z* 1165.61468 $[M+H]^+$ (calculated for $C_{73}H_{85}N_2O_{11}^+$ 1165.61479).

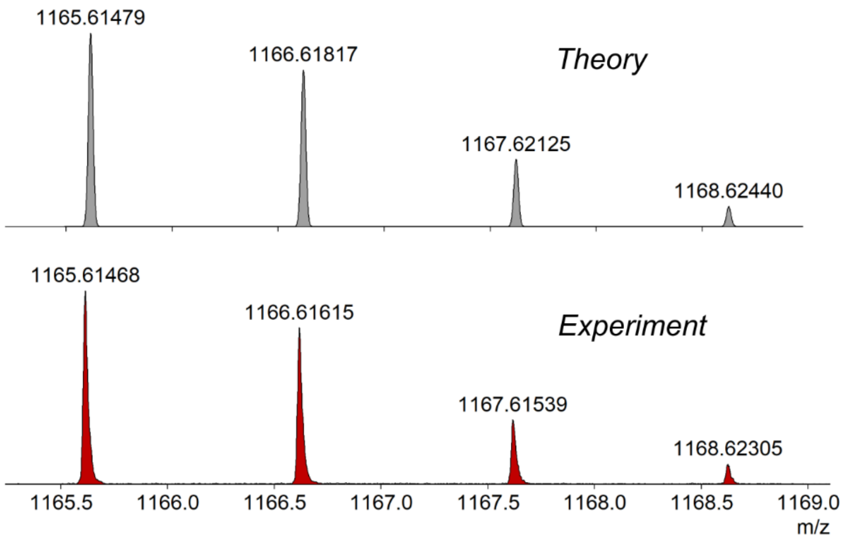

Figure S12. HRMS peaks of rotaxane **1i**: simulated (top) and experimental (bottom) isotopic patterns.

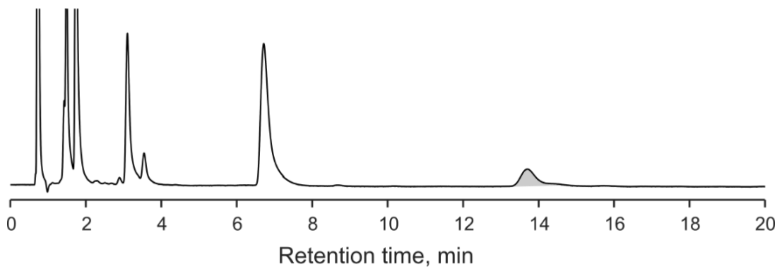

Figure S13. HPLC chromatogram of the reaction mixture of **1i** synthesis (3rd day). Shaded peak (grey) corresponds to rotaxane **1i**.

Rotaxane 1j

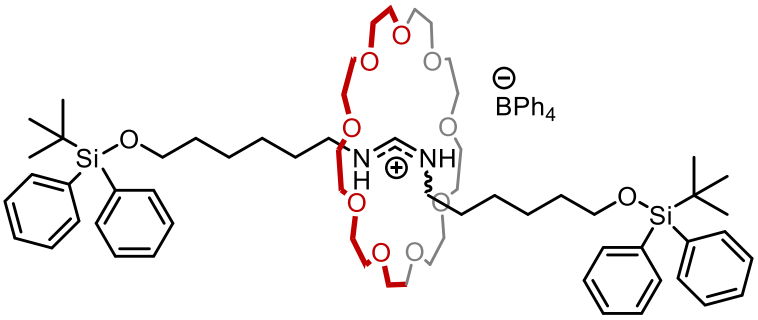

Rotaxane **1j** (HPLC yield: 12%) was synthesized according to Method A. Reaction conditions: 3.0 eq. amine **4h**, solvent – PhMe, 75 °C, 3 days. HPLC yield was calculated using 1,2,4,5-tetramethylbenzene as an internal standard and a corresponding calibration curve for thread **2h** (it was assumed that molar absorptivities of the rotaxane and the free thread are equal; see Section 3, "Reaction monitoring").

HRMS (ESI): *m/z* 1117.68652 $[M+H]^+$ (calculated for $C_{63}H_{101}N_2O_{11}Si_2^+$ 1117.69384).

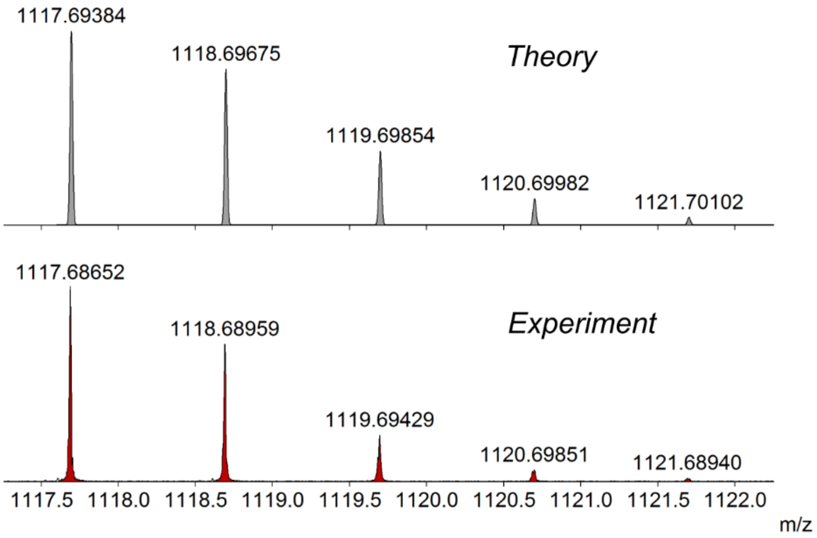

Figure S14. HRMS peaks of rotaxane **1j**: simulated (top) and experimental (bottom) isotopic patterns.

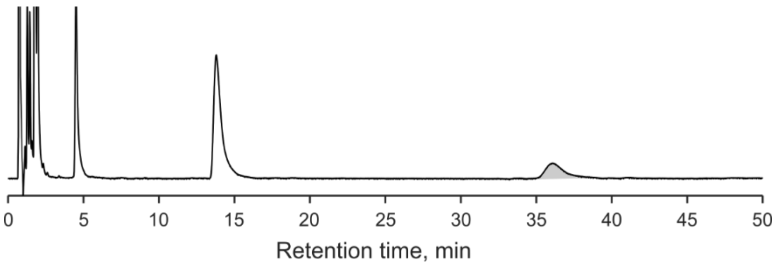

Figure S15. HPLC chromatogram of the reaction mixture of **1j** synthesis (3rd day). Shaded peak (grey) corresponds to rotaxane **1j**.

5. Tandem mass spectra of the amidinium [2]rotaxanes

MS-MS spectra of rotaxanes **1a-c**, **1e**, **1h**, **1j** were measured on Bruker SolariX FT-ICR mass spectrometer (collision gas - Ar). MS-MS spectra of rotaxanes **1d**, **1f**, **1g** and **1i** were measured on Agilent QTOF 6546 mass spectrometer (collision gas – N_2).

The samples were prepared by 20 000-fold dilution of 1 µL of a reaction mixture of the corresponding rotaxane self-assembly in MeCN. A control sample (Figure S17) was prepared in the following way: a free thread was synthesized by stirring a mixture of **FA·BPh$_4$** (14.6 mg, 40 µmol, 1.0 eq.) and 3,5-di-*tert*-butylbenzylamine (17.5 mg, 80 µmol, 2.0 eq.) in MeCN (200 µL) at r.t. for 3 days; 1 µL of the reaction mixture was diluted 20 000-fold in MeCN, and **24C8** (3.0 eq.) was added to the obtained sample prior to injection into the mass spectrometer.

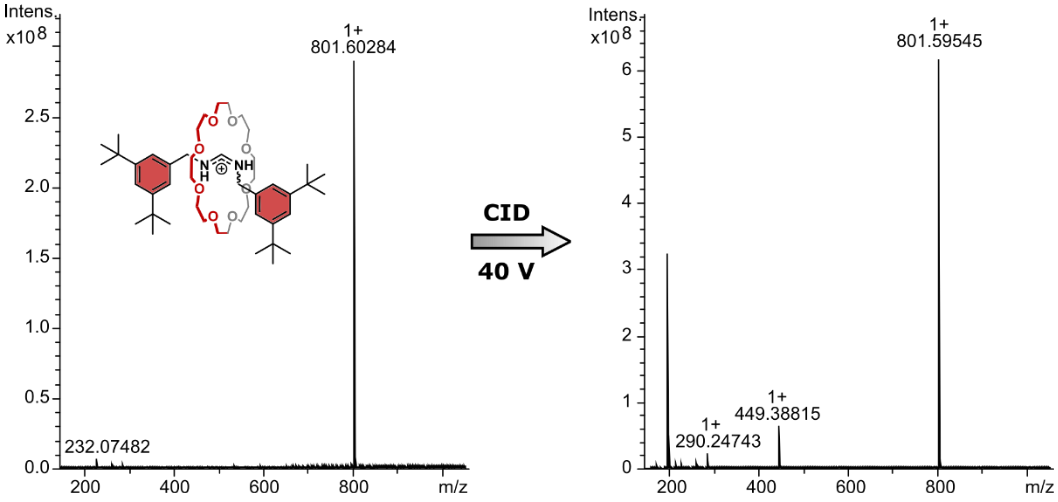

Figure S16. *Left*: Mass spectrum of **1a** (isolated ion) before fragmentation. *Right*: MS-MS spectrum of **1a** (collision energy – 40 V). CID – collision induced dissociation.

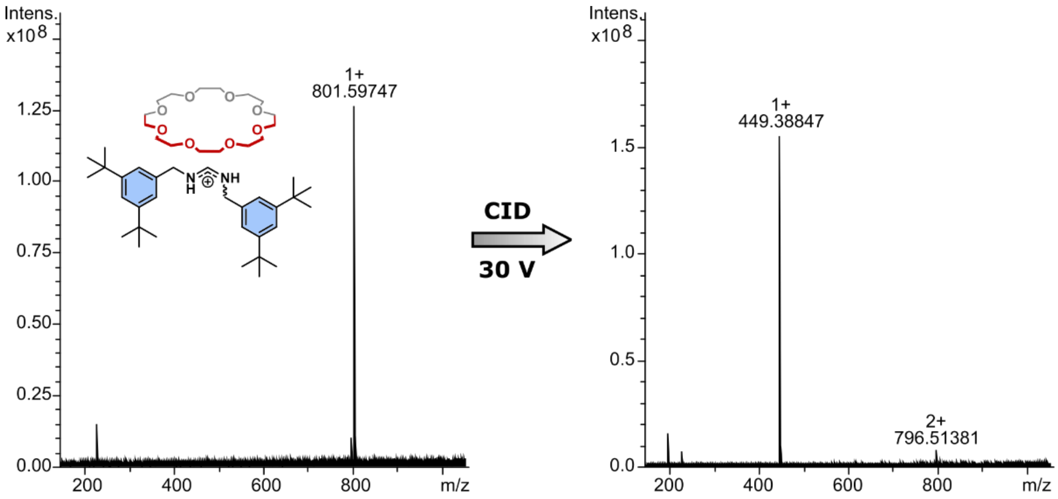

Figure S17. *Left*: Mass spectrum of the non-interlocked complex between **2a** and **24C8** (isolated ion) before fragmentation. *Right*: MS-MS spectrum of the non-interlocked complex (collision energy – 30 V). CID – collision induced dissociation.

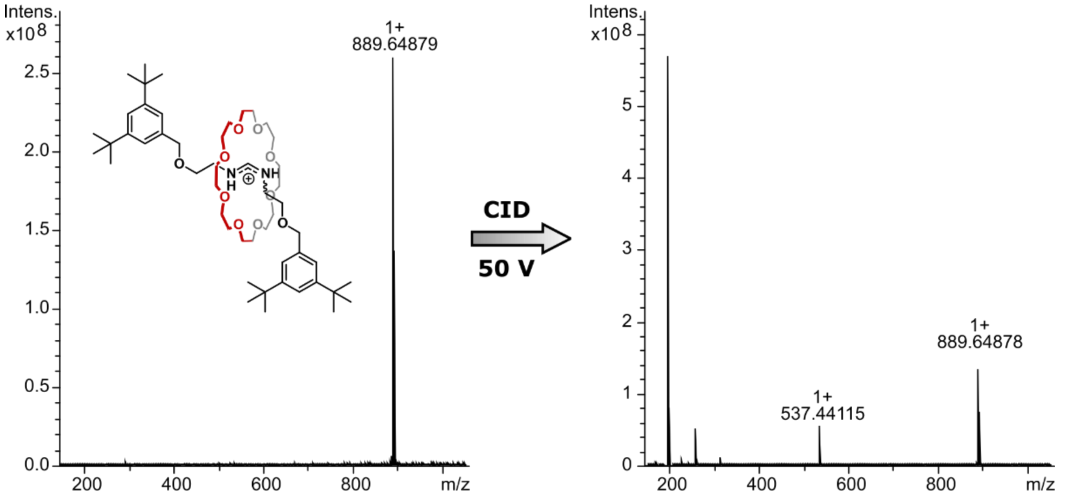

Figure S18. *Left*: Mass spectrum of **1b** (isolated ion) before fragmentation. *Right*: MS-MS spectrum of **1b** (collision energy – 50 V). CID – collision induced dissociation.

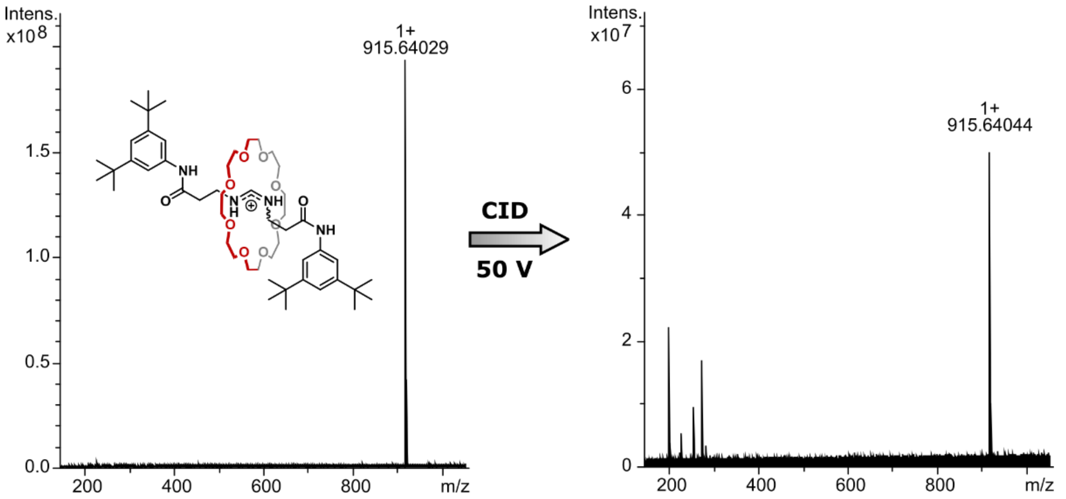

Figure S19. *Left*: Mass spectrum of **1c** (isolated ion) before fragmentation. *Right*: MS-MS spectrum of **1c** (collision energy – 50 V). CID – collision induced dissociation.

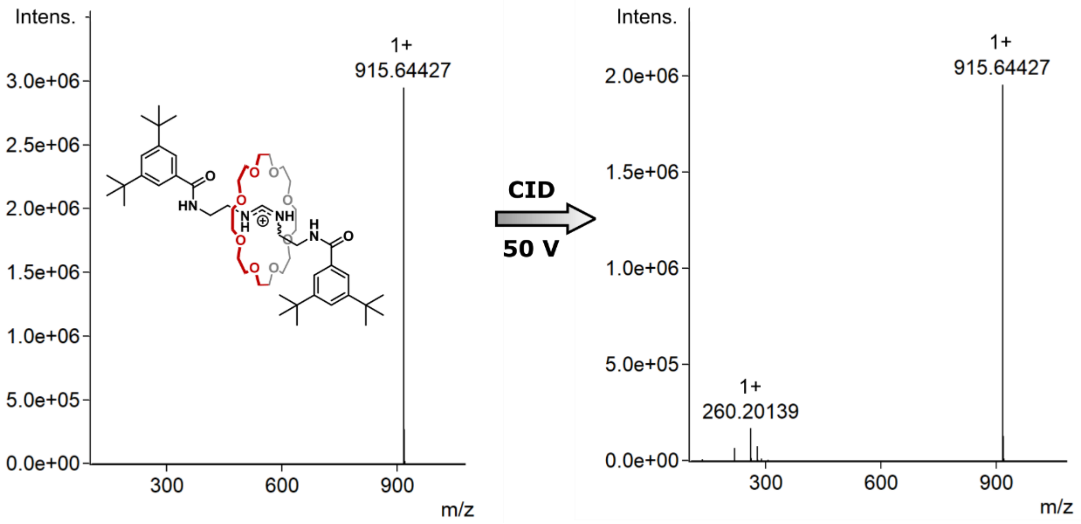

Figure S20. *Left*: Mass spectrum of **1d** (isolated ion) before fragmentation. *Right*: MS-MS spectrum of **1d** (collision energy – 50 V). CID – collision induced dissociation.

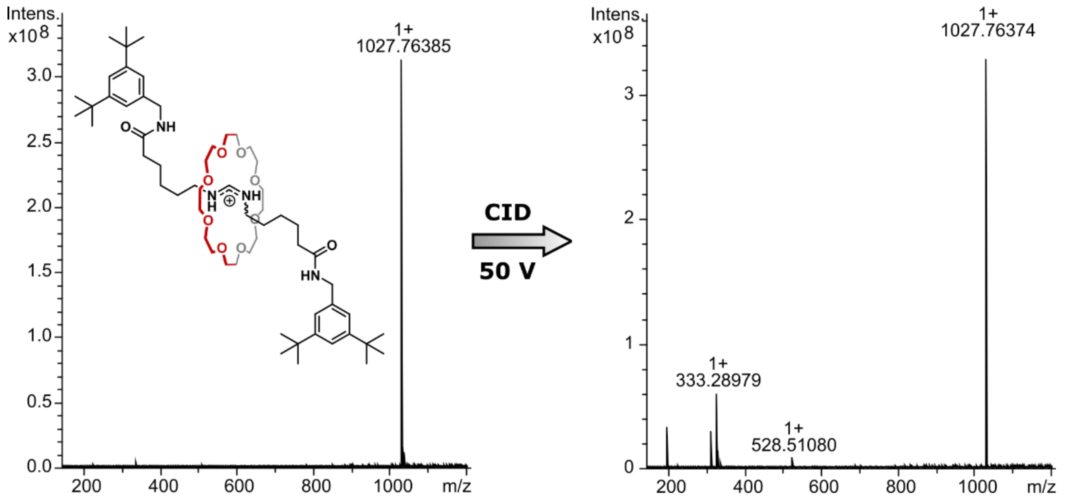

Figure S21. *Left*: Mass spectrum of **1e** (isolated ion) before fragmentation. *Right*: MS-MS spectrum of **1e** (collision energy – 50 V). CID – collision induced dissociation.

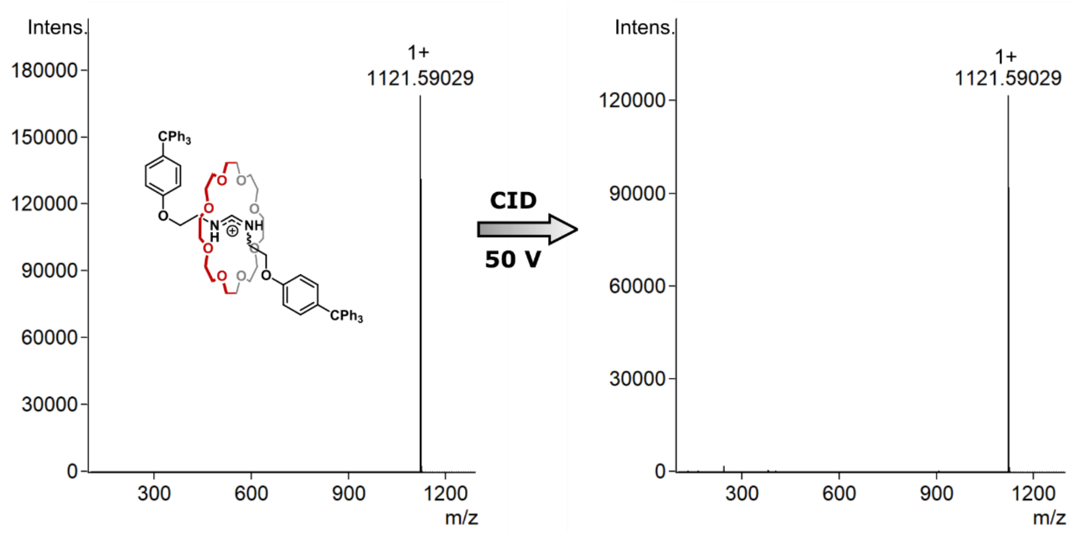

Figure S22. *Left*: Mass spectrum of **1f** (isolated ion) before fragmentation. *Right*: MS-MS spectrum of **1f** (collision energy – 50 V). CID – collision induced dissociation.

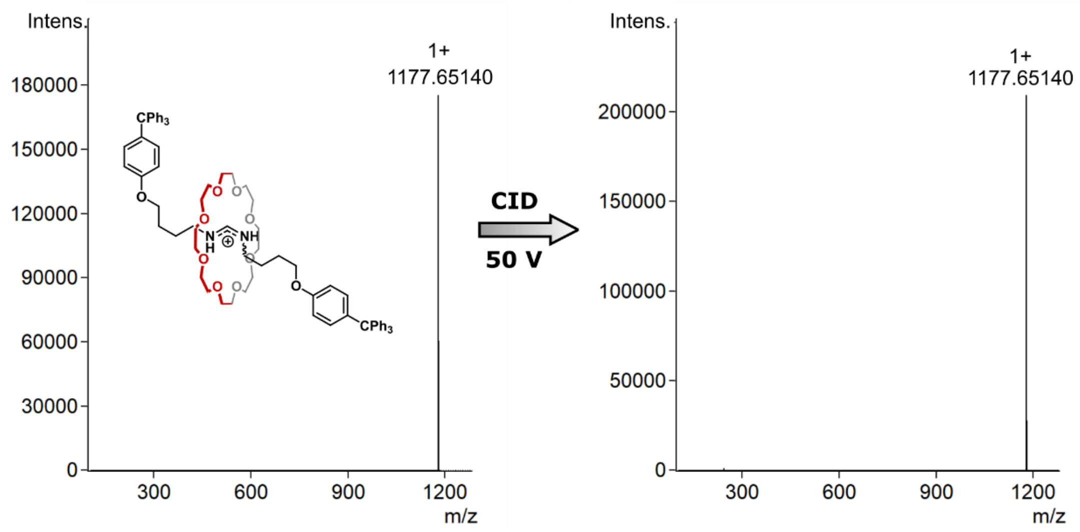

Figure S23. *Left*: Mass spectrum of **1g** (isolated ion) before fragmentation. *Right*: MS-MS spectrum of **1g** (collision energy – 50 V). CID – collision induced dissociation.

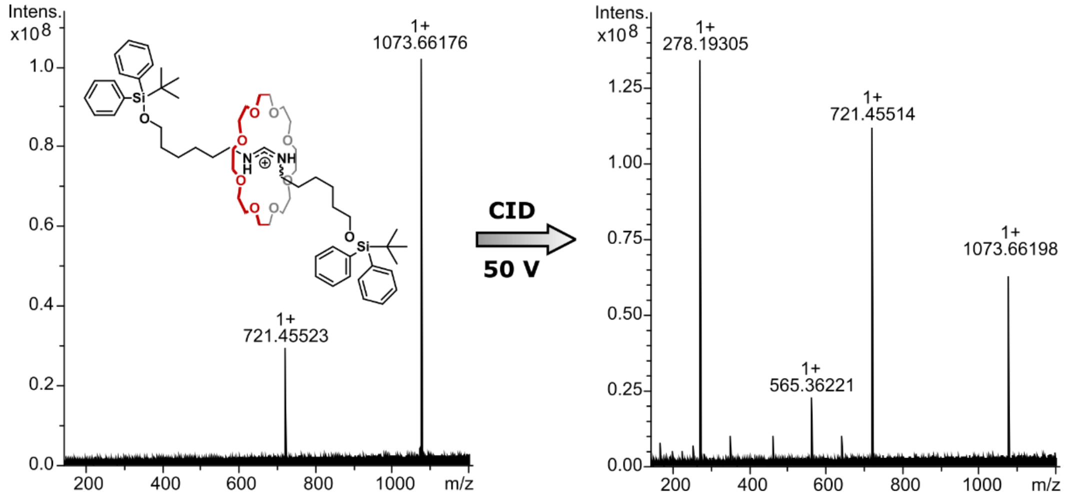

Figure S24. *Left*: Mass spectrum of **1h** (isolated ion) before fragmentation. *Right*: MS-MS spectrum of **1h** (collision energy – 50 V). CID – collision induced dissociation.

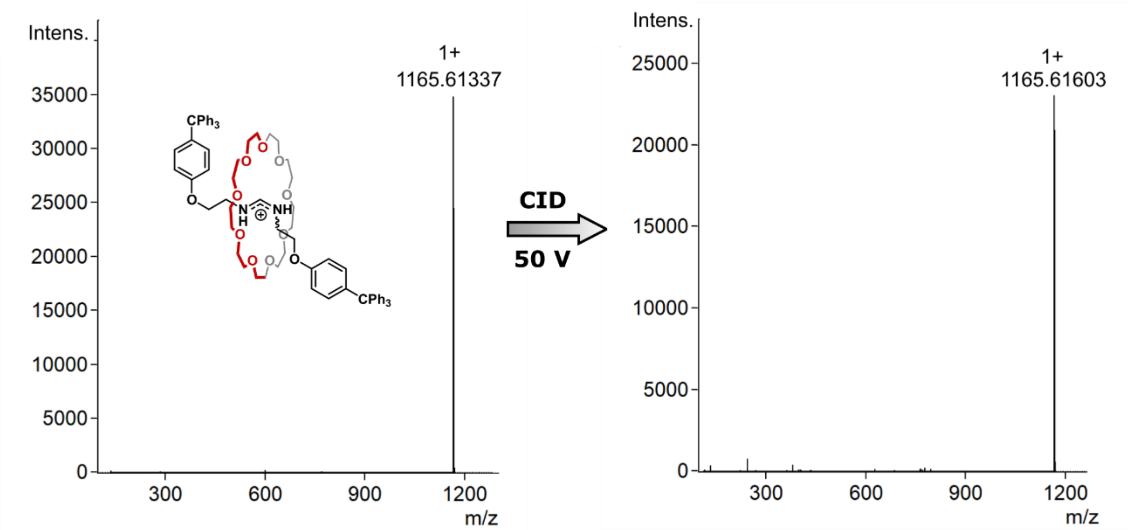

Figure S25. *Left*: Mass spectrum of **1i** (isolated ion) before fragmentation. *Right*: MS-MS spectrum of **1i** (collision energy – 50 V). CID – collision induced dissociation.

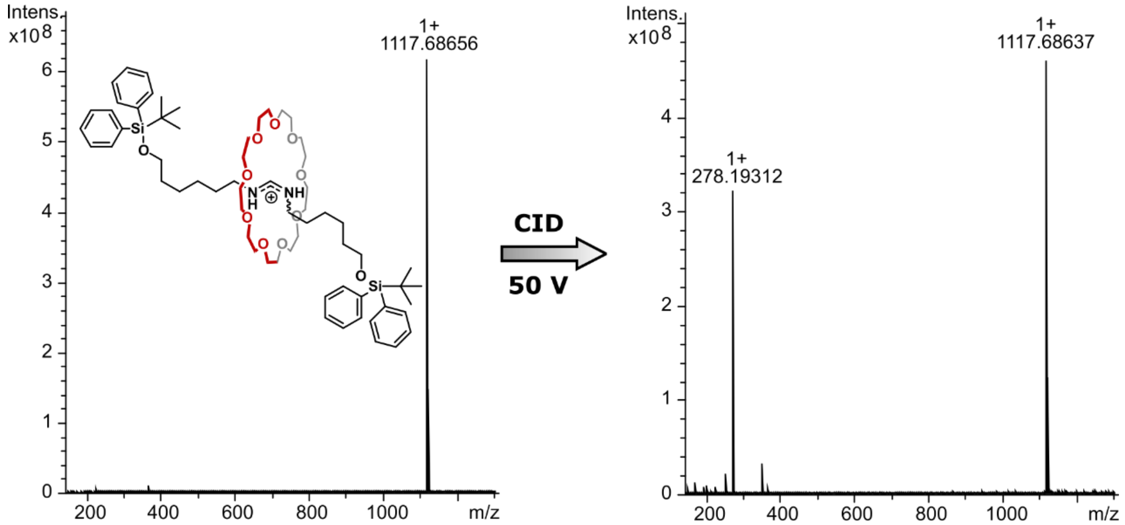

Figure S26. *Left*: Mass spectrum of **1j** (isolated ion) before fragmentation. *Right*: MS-MS spectrum of **1j** (collision energy – 50 V). CID – collision induced dissociation.

6. Binding studies: NMR titrations

Unless otherwise specified, NMR titrations were performed in CD_3CN at 295 K on 400 MHz NMR spectrometer. The concentration of guest species (i.e., formamidinium (**FA**), *N*-3,5-di-*tert*-butylbenzylformamidinium **3a** and *N,N'*-dibenzylformamidine) was kept constant over the course of titrations while the concentration of the host – 24-crown-8 (**24C8**) – was gradually increased. In case of NH_4^+ and **24C8**, the opposite procedure was applied: the concentration of **24C8** was kept constant, while concentration of NH_4^+ was gradually increased. Concentration of the guest species in stock solutions was determined by quantitative ^{1}H NMR (qNMR) using dimethyl terephthalate as a standard (TraceCERT® NMR standard purchased from Sigma-Aldrich). Prior to NMR titrations, stock solutions of the titrated species were diluted with CD_3CN in a 5 mm NMR tube to a desired concentration. Except for titration with NH_4^+⊂**24C8** host-guest system, exact concentration of **24C8** at each stage of titration was calculated based on tetraphenylborate signal at δ 6.84 ppm in ^{1}H NMR spectrum as an internal standard.

Titration data was fitted with the online calculator Bindfit (http://supramolecular.org). All raw data, calculated fits and related data can be accessed online (see the web links below).

Association constant for the pseudorotaxane formed from *N*,*N'*-dibenzylformamidinium **S1** and **24C8** was determined by the single-point method: equilibrium concentrations of all species participating in the equilibrium could be determined by integration of the corresponding ^{1}H NMR signals.[16] Dimethyl terephthalate was used as an internal standard.

6.1 Binding of 24-crown-8 to formamidinium ion

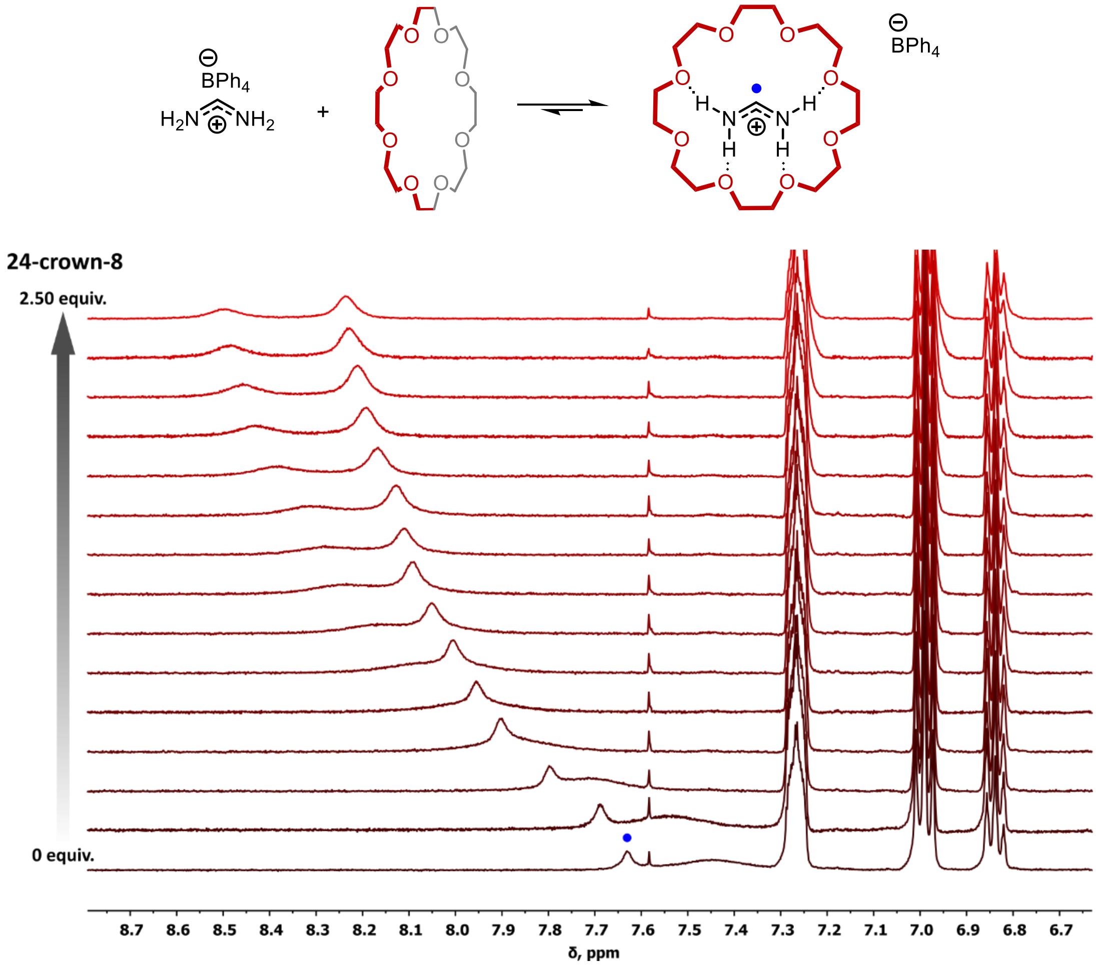

Figure S27. Representative ^{1}H NMR stack plot for titration of **FA·BPh₄** (2.0 mM) with **24C8** (0 – 2.50 eq.) in CD_3CN.

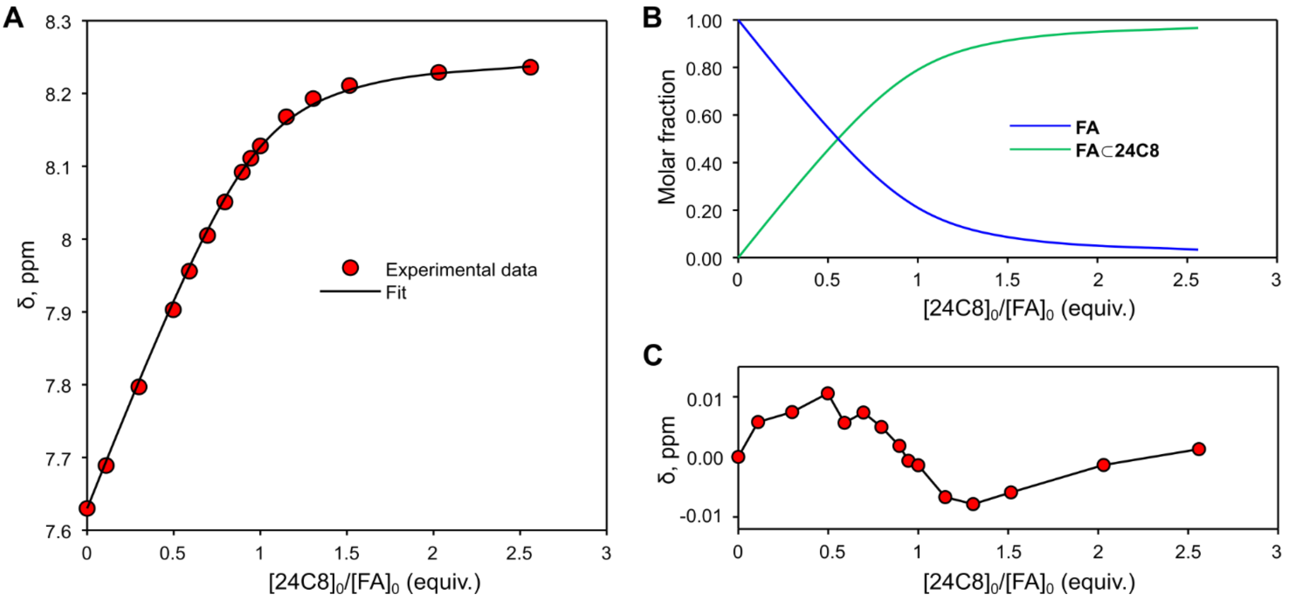

Figure S28. Representative titration data for **FA⊂24C8** host-guest system. Solvent: CD_3CN. [**FA**]$_0$ = 2.0 mM. (A) Fit of the titration data to 1:1 binding model. Fitting the data to 1:2 or 2:1 model resulted in high fit error and did not give reproducible results while analyzing repeated titrations. (B) Dependence of molar fractions of **FA** and **FA⊂24C8** on relative amount of **24C8**. (C) Residuals plot.

Table S7. Association constants for formamidinium and **24C8** obtained by fitting the titration data to 1:1 binding model. Fit method: Nelder-Mead. Solvent: CD_3CN. Temperature: 295 K. $s/\sqrt{n}$ – standard deviation of the mean, where s – standard deviation, n – number of measurements; $t_{(0.05,3)} \times s/\sqrt{n}$ – 95% confidence interval, where $t_{(0.05,\, n-1)}$ – Student's t at 95% confidence level.[17]

[FA·BPh$_4$]$_{tot}$, mM	K_a, M^{-1}	Fit error, %	$\bar{K}_a$, M^{-1}	$s/\sqrt{n}$, M^{-1}	$t_{(0.05,3)} \times s/\sqrt{n}$, M^{-1}
2.0	8970	± 8.6	9490	444	± 1410
2.0	8510	± 9.4			
2.5	10400	± 9.3			
3.0	10100	± 9.6			

Table S8. Links to the raw titration data, calculated fits and statistical information for the fits.

[FA·BPh$_4$]$_{tot}$, mM	*Web link*
2.0	http://app.supramolecular.org/bindfit/view/8c812370-2650-41c2-a69b-a082a8b9e8c7
2.0	http://app.supramolecular.org/bindfit/view/cb0fd736-a705-4392-94fc-0f1fc423c299
2.5	http://app.supramolecular.org/bindfit/view/c98715a0-eaa5-4e07-a3d8-65cad01b8214
3.0	http://app.supramolecular.org/bindfit/view/a5306f26-0170-4e4b-9138-dd591ed7ac79

6.2 Binding of 24-crown-8 to *N*-3,5-di-*tert*-butylbenzylformamidinium ion (3a)

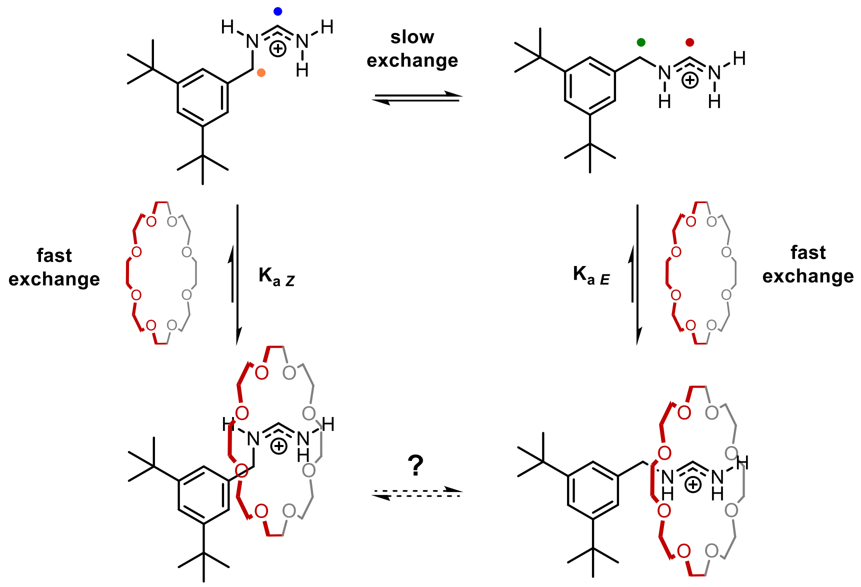

Scheme S2. Host-guest and isomerization equilibria present during titration of **3a** with **24C8**.

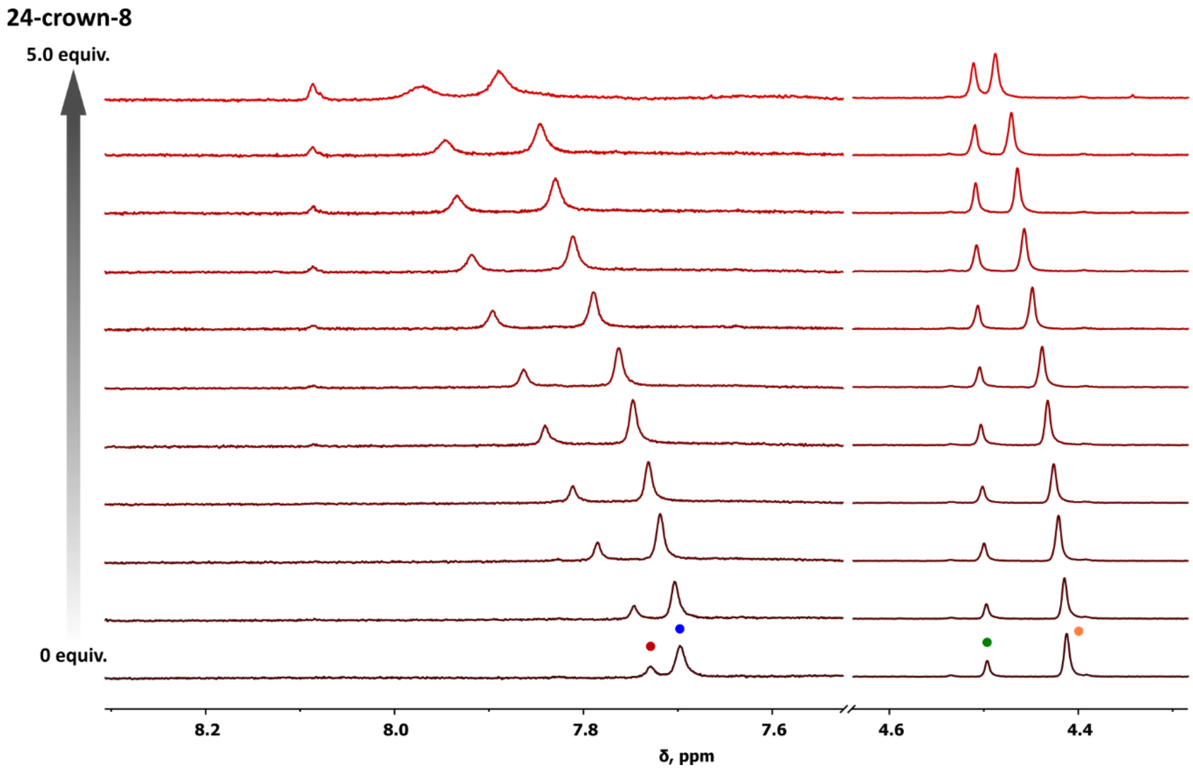

Figure S29. Representative ^{1}H NMR stack plot for titration of **3a** (3.0 mM) with **24C8** (0 – 5.0 eq. with respect to **3a**) in CD_3CN. Signal assignment to *E* or *Z* isomers is an educated guess based on comparison with ^{1}H NMR spectrum of **2a** in CD_3CN.

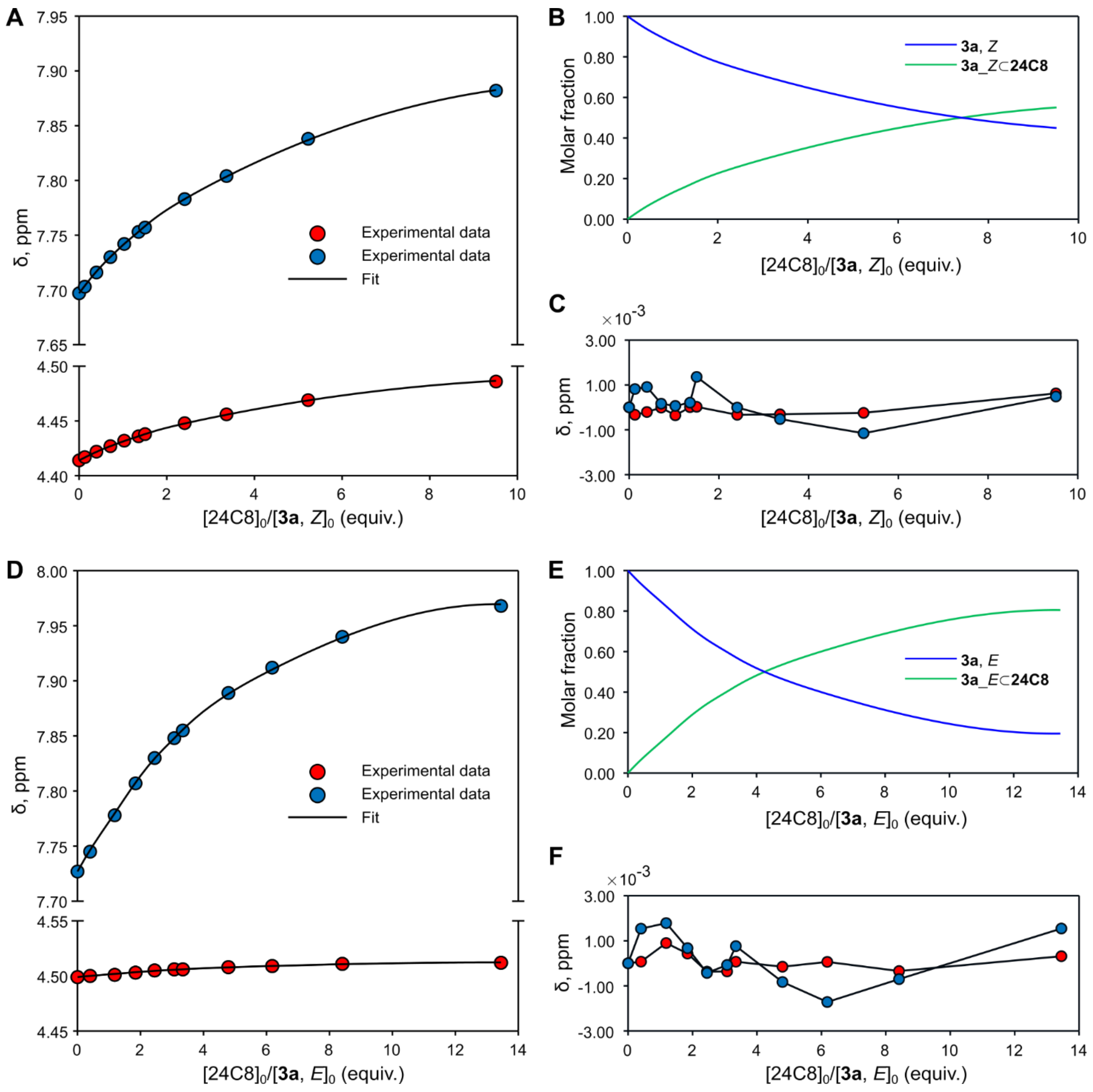

Figure S30. Representative titration data for **3a⊂24C8** host-guest system. Solvent: CD_3CN. [**3a**]$_0$ = 3.0 mM. (A,D) Fit of the titration data for (A) *Z* isomer and (D) *E* isomer of **3a** to 1:1 binding model. (B,E) Dependence of molar fractions of (B) **3a**_*Z* and **3a**_*Z*⊂**24C8** or (E) **3a**_*E* and **3a**_*E*⊂**24C8** on relative amount of **24C8**. (C,F) Residuals plots.

Important note. In order to fit the titration data to 1:1 binding model, the two host-guest equilibria (Scheme S2) were treated independently. Total concentrations of **3a**_*Z* and **3a**_*E* were calculated based on known total concentration of **3a** and molar ratio of **3a**_*Z* and **3a**_*E* (obtained from integration of the corresponding signals in ^{1}H NMR spectrum) at each step of the titration. On the other hand, the same total concentration of **24C8** was used to fit the data for both isomers of **3a**, yet it is clear that the true "total" concentration of **24C8** that has to be used for data fitting for each isomer must be smaller (since 1:1 binding model does not account for additional equilibria with host or guest; in our case – binding of **24C8** to the second isomer is not considered during data fitting). However, since only a small fraction of **24C8** is bound to **3a** at each step of the titration, a decrease in the concentration of **24C8** due to the competing binding can be considered (in the first approximation) as insignificant. Nevertheless, the obtained association constants (*vide infra*) should be considered *as a rough estimation rather than a precise value*.

Table S9. Approximate association constants for *N*-3,5-di-*tert*-butylbenzylformamidinium (**3a**) and **24C8** obtained by fitting the titration data according to 1:1 binding model. Fit method: Nelder-Mead. Solvent: CD_3CN. Temperature: 295 K. $s/\sqrt{n}$ – standard deviation of the mean, where *s* – standard deviation, *n* – number of measurements; $t_{(0.05,3)} \times s/\sqrt{n}$ – 95% confidence interval, where $t_{(0.05, n-1)}$ – Student's t at 95% confidence level.[17]

[3a]$_{tot}$, mM	K_Z, M^{-1}	K_E, M^{-1}	$\bar{K}_Z$, M^{-1}	$s/\sqrt{n}$, M^{-1}	$t_{(0.05,3)} \times s/\sqrt{n}$, M^{-1}	$\bar{K}_E$, M^{-1}	$s/\sqrt{n}$, M^{-1}	$t_{(0.05,3)} \times s/\sqrt{n}$, M^{-1}
3.0	83	281						
3.0	81	274	**86**	4	19	**285**	7	32
1.1	95	299						

Table S10. Links to the raw titration data, calculated fits and statistical information for the fits.

[3a]$_{tot}$, mM	***Web link***
3.0 (*Z*)	http://app.supramolecular.org/bindfit/view/7fc33aba-c611-4cfe-85a0-931f9c4ab71f
3.0 (*E*)	http://app.supramolecular.org/bindfit/view/76e7f9bf-65c0-4110-befa-8d16184dc6ed
3.0 (*Z*)	http://app.supramolecular.org/bindfit/view/68f2a4b3-ad5d-48ba-91ee-d300f0775f58
3.0 (*E*)	http://app.supramolecular.org/bindfit/view/186db8bc-9c03-47b2-866e-e6c9446e0454
1.1 (*Z*)	http://app.supramolecular.org/bindfit/view/82f50ff7-08e5-4bce-a2b1-fe2a5e475889
1.1 (*E*)	http://app.supramolecular.org/bindfit/view/815f8460-f4d7-44d1-844a-60d1fa40d297

6.3 Binding of 24-crown-8 to *N,N'*-dibenzylformamidinium ion (S1)

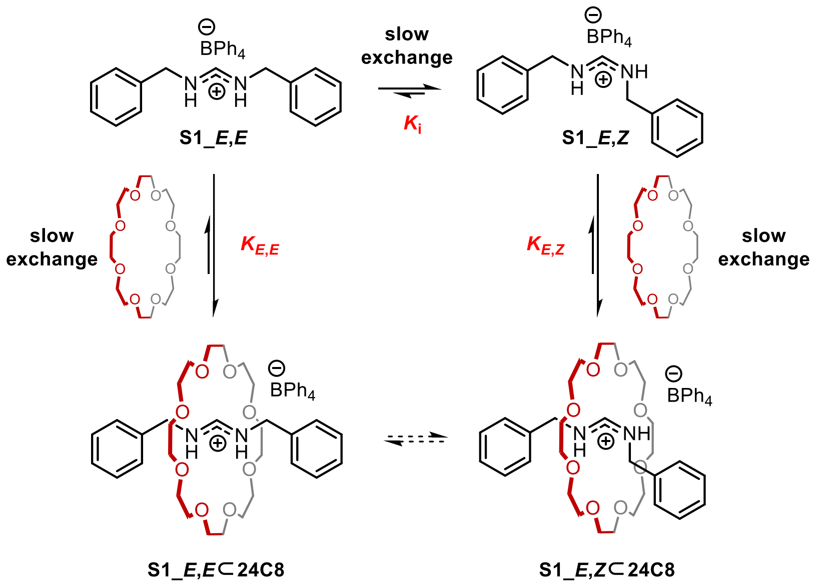

Scheme S3. Host-guest and isomerization equilibria present in the mixture of **S1** and **24C8**.

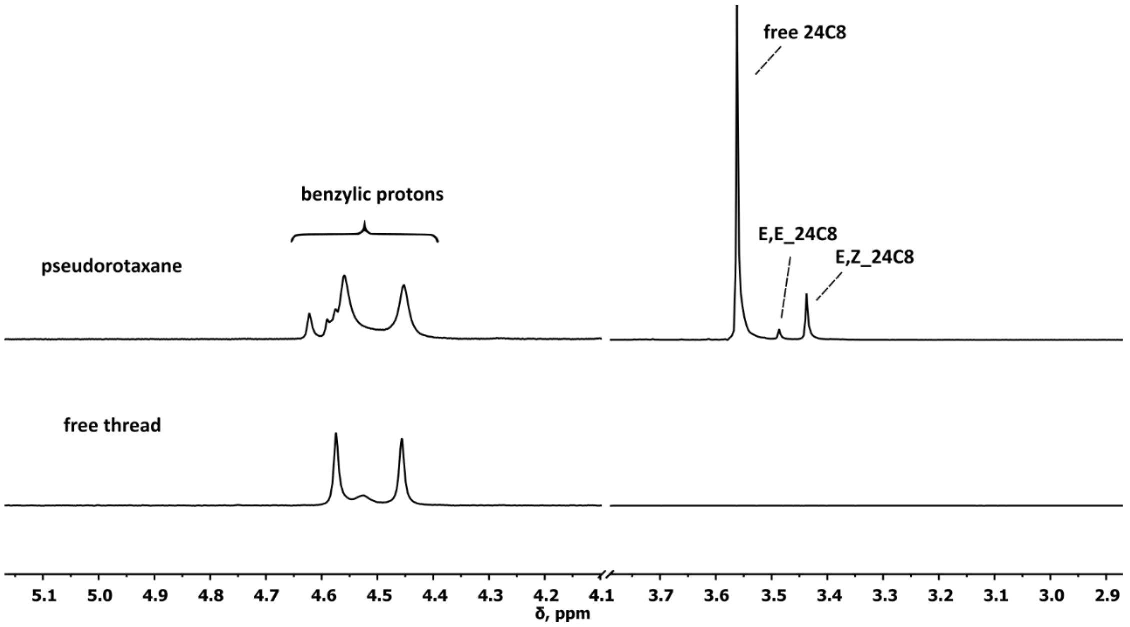

Figure S31. Representative partial ^{1}H NMR spectra of **S1** (10 mM in CD_3CN) at 295 K before (bottom) and after (top) addition of **24C8** (1.0 eq.). For convenience of representation, the right and the left part of the NMR spectra are not to scale (left part – 20x zoom with respect to the right part).

$$\mathbf{S1\_}E,E\subset\mathbf{24C8} \underset{}{\overset{K_{E,E}}{\rightleftharpoons}} \mathbf{S1\_}E,E \overset{K_i}{\rightleftharpoons} \mathbf{S1\_}E,Z \overset{K_{E,Z}}{\rightleftharpoons} \mathbf{S1\_}E,Z\subset\mathbf{24C8}$$

$$K_i = \frac{[\mathbf{S1}\_E,Z]}{[\mathbf{S1}\_E,E]} \qquad K_{E,Z} = \frac{[\mathbf{S1}\_E,Z \subset \mathbf{24C8}]}{[\mathbf{S1}\_E,Z][\mathbf{24C8}]} \qquad K_{E,E} = \frac{[\mathbf{S1}\_E,E \subset \mathbf{24C8}]}{[\mathbf{S1}\_E,E][\mathbf{24C8}]}$$

Due to slow exchange on the NMR timescale (association/dissociation and isomerization equilibria), equilibrium concentrations of both isomers of the pseudorotaxane – **S1**_*E*,*E*⊂**24C8** and **S1**_*E*,*Z*⊂**24C8** – as well as equilibrium concentration of the free **24C8** and total concentration c_{tot} of all isomers of complexed and free **S1** (thread) could be determined by integration of the corresponding signals in ^{1}H NMR spectrum. Equilibrium concentrations of both isomers of the free thread were calculated according to equations S1 and S2:

$$[\mathbf{S1}\_E,E]_{tot} = [\mathbf{S1}\_E,E] + [\mathbf{S1}\_E,E \subset \mathbf{24C8}]$$

$$[\mathbf{S1}\_E,Z]_{tot} = [\mathbf{S1}\_E,Z] + [\mathbf{S1}\_E,Z \subset \mathbf{24C8}]$$

$$c_{tot} = [\mathbf{S1}\_E,E]_{tot} + [\mathbf{S1}\_E,Z]_{tot}$$

$$c_{tot} = [\mathbf{S1}\_E,E] + K_i[\mathbf{S1}\_E,E] + [\mathbf{S1}\_E,E \subset \mathbf{24C8}] + [\mathbf{S1}\_E,Z \subset \mathbf{24C8}]$$

$$[\mathbf{S1}\_E,E] = \frac{c_{tot} - ([\mathbf{S1}\_E,E \subset \mathbf{24C8}] + [\mathbf{S1}\_E,Z \subset \mathbf{24C8}])}{1 + K_i} \qquad \text{(S1)}$$

$$[\mathbf{S1}\_E,Z] = K_i[\mathbf{S1}\_E,E] \qquad \text{(S2)}$$

Isomerization constant K_i could be calculated from the ^{1}H NMR spectrum of the free thread in CD_3CN via integration of benzylic signals of *E*,*E*- and *E*,*Z*-isomer. Binding constants $K_{E,Z}$ and $K_{E,E}$ were calculated as the mean of the constants obtained from separate measurements at different total concentrations of the thread (c_{tot}) and **24C8**.

Table S11. Association constants for *N*,*N'*-dibenzylformamidinium and **24C8** determined by the single-point method.[16] Solvent: CD_3CN. Temperature: 295 K. $s/\sqrt{n}$ – standard deviation of the mean, where *s* – standard deviation, *n* – number of measurements; $t_{(0.05,\,3)} \times s/\sqrt{n}$ – 95% confidence interval, where $t_{(0.05,\,n\text{-}1)}$ – Student's t at 95% confidence level.[17]

c_{tot}, mM	$[\mathbf{24C8}]_{tot}$, mM	$K_{E,Z}$, M^{-1}	$K_{E,E}$, M^{-1}	$\bar{K}_{E,Z}$, M^{-1}	$s/\sqrt{n}$, M^{-1}	$t_{(0.05,\,5)} \times s/\sqrt{n}$, M^{-1}	$\bar{K}_{E,E}$, M^{-1}	$s/\sqrt{n}$, M^{-1}	$t_{(0.05,\,5)} \times s/\sqrt{n}$, M^{-1}
3.0	3.0	28	32	24	1	± 3	32	1	± 2
3.0	4.6	28	31						
6.6	5.9	21	31						
10	5.0	21	29						
10	10	24	32						
10	15	24	35						

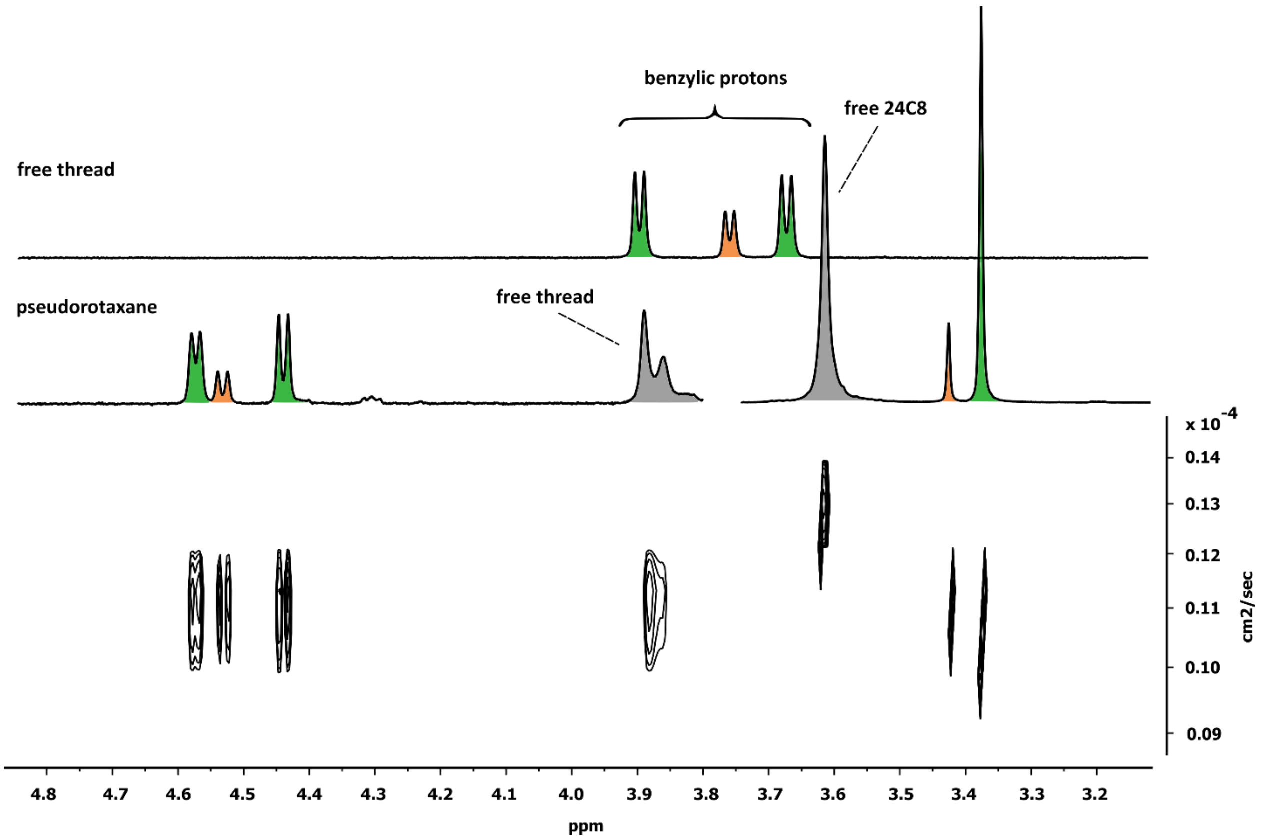

Figure S32. ^{1}H NMR stack plot of the spectra of **S1** (3 mM) in $CDCl_3/CD_3CN$ (95:5 by volume) at 295 K before (top) and after (bottom) addition of **24C8** (1.0 eq., 3 mM). In the lower spectrum, the right and the left part are not to scale (left part – 10x zoom with respect to the right part). Green signals correspond to *E,Z*-isomers and orange signals – to *E,E*-isomers. Underneath the NMR stack plot – a DOSY spectrum of the mixture of **S1** and **24C8**, which confirms the formation of the pseudorotaxane. We expect the free thread and the corresponding pseudorotaxane to have very close hydrodynamic radii and, consequently, diffusion coefficients. It is worth mentioning that, as expected, in $CDCl_3/CD_3CN$ (95:5 v/v), the binding constant for **S1** and **24C8** is higher compared to pure CD_3CN (more **24C8** is converted to pseudorotaxane).

6.3.1 Binding of 24-crown-8 to thread S1 and amine 4a: competition experiment

It is known that aliphatic amines and their ammonium salts can bind to crown ethers including **24C8**.[18] Such interactions may interfere with binding of **24C8** to the amidinium moiety and, eventually, with the overall rotaxane self-assembly by the amidinium exchange. In order to check if a primary amine interferes with association of **24C8** and *N,N'*-dialkylamidinium thread, 3,5-di-*tert*-butylbenzylamine **4a** (1.0 eq. with respect to **S1**, i.e. 6.9 µL of 0.35 M stock solution of the amine in CD_3CN) was added to a solution of **S1** (3 mM in CD_3CN, 800 µL) containing **24C8** (1.5 eq. with respect to **S1**, i.e. 3.6 mmol). Indeed, the amount of the pseudorotaxane decreased ~ 1.5-fold and the amine benzylic signal in ^1H NMR spectrum shifted downfield indicating that the amino group was partially protonated (Figure S33).

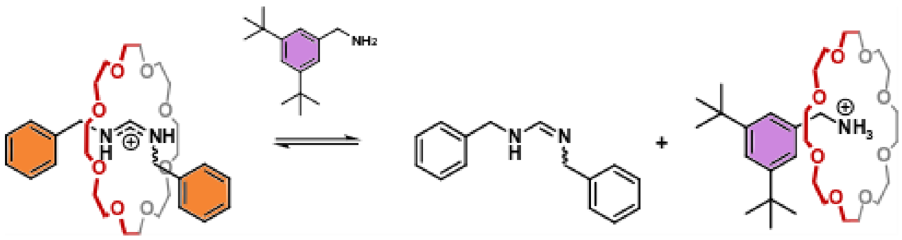

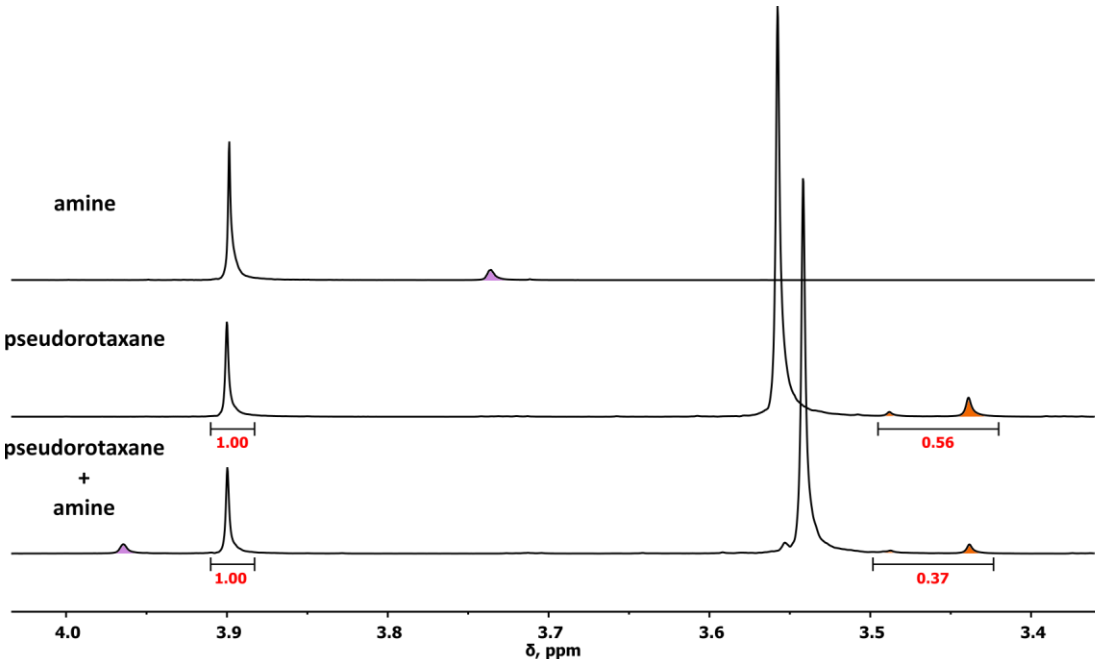

Figure S33. ^1H NMR (400 MHz, CD_3CN, 295 K) stack plot of the spectra of **4a** (top), a mixture of **S1** (1.0 eq., 3 mM) and **24C8** (1.5 eq., 4.5 mM) (middle), and the same mixture after addition of **4a** (1.0 eq., 3 mM). Orange signals: CH_2 signals of **24C8** in *E*,*E*- and *E*,*Z*-isomers of the pseudorotaxane. Purple signals: benzylic CH_2 signals of **4a**. A singlet at 3.9 ppm – CH_3 signal of the internal standard (dimethyl terephthalate).

6.4 Binding of 24-crown-8 to *N,N′*-dibenzylformamidine

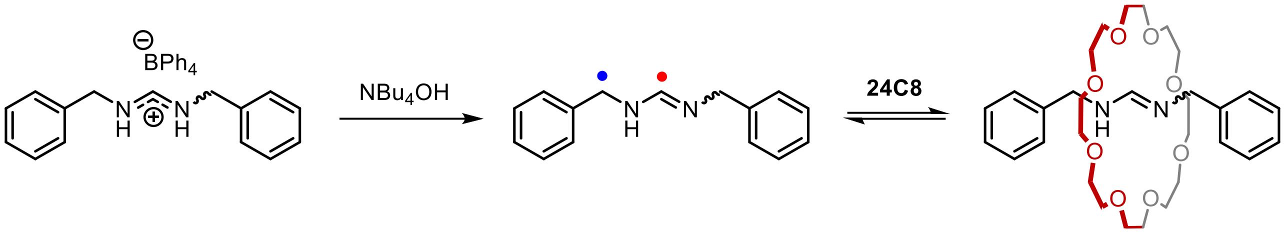

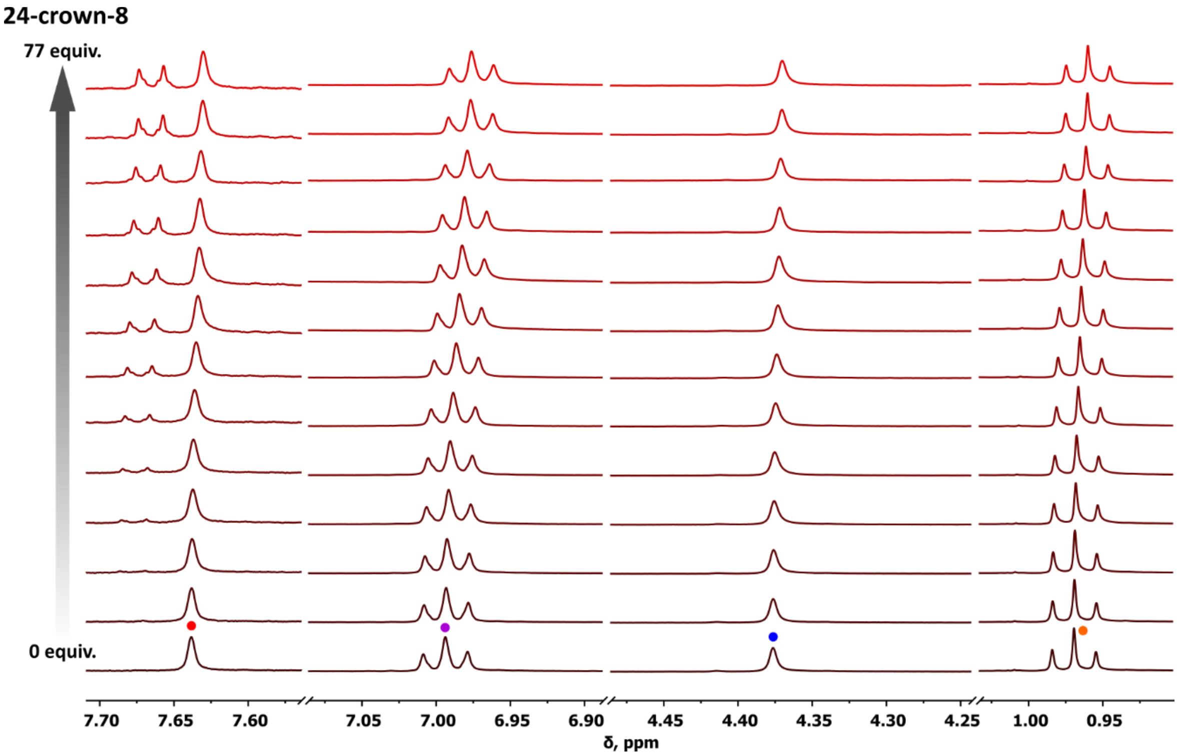

Figure S34. ^{1}H NMR (500 MHz, 300 K) stack plot for titration of *N*,*N′*-dibenzylformamidine (5.0 mM) with **24C8** (0 – 77 eq.) in CD_3CN. *N*,*N′*-Dibenzylformamidine was obtained by deprotonation of **S1** (BPh_4^- salt, 1.0 eq.) with NBu_4OH (1.0 M solution in MeOH, 1.0 eq.) in THF. After removal of all volatiles under reduced pressure and thorough drying under high vacuum, the residue containing *N*,*N′*-dibenzylformamidine and NBu_4BPh_4 was used as it is for the titration with **24C8**. ^{1}H NMR signals marked purple and orange correspond to CH protons from BPh_4^- and CH_3 protons from NBu_4^+ respectively. Peaks at 7.66 – 7.70 ppm are minor impurities from **24C8**.

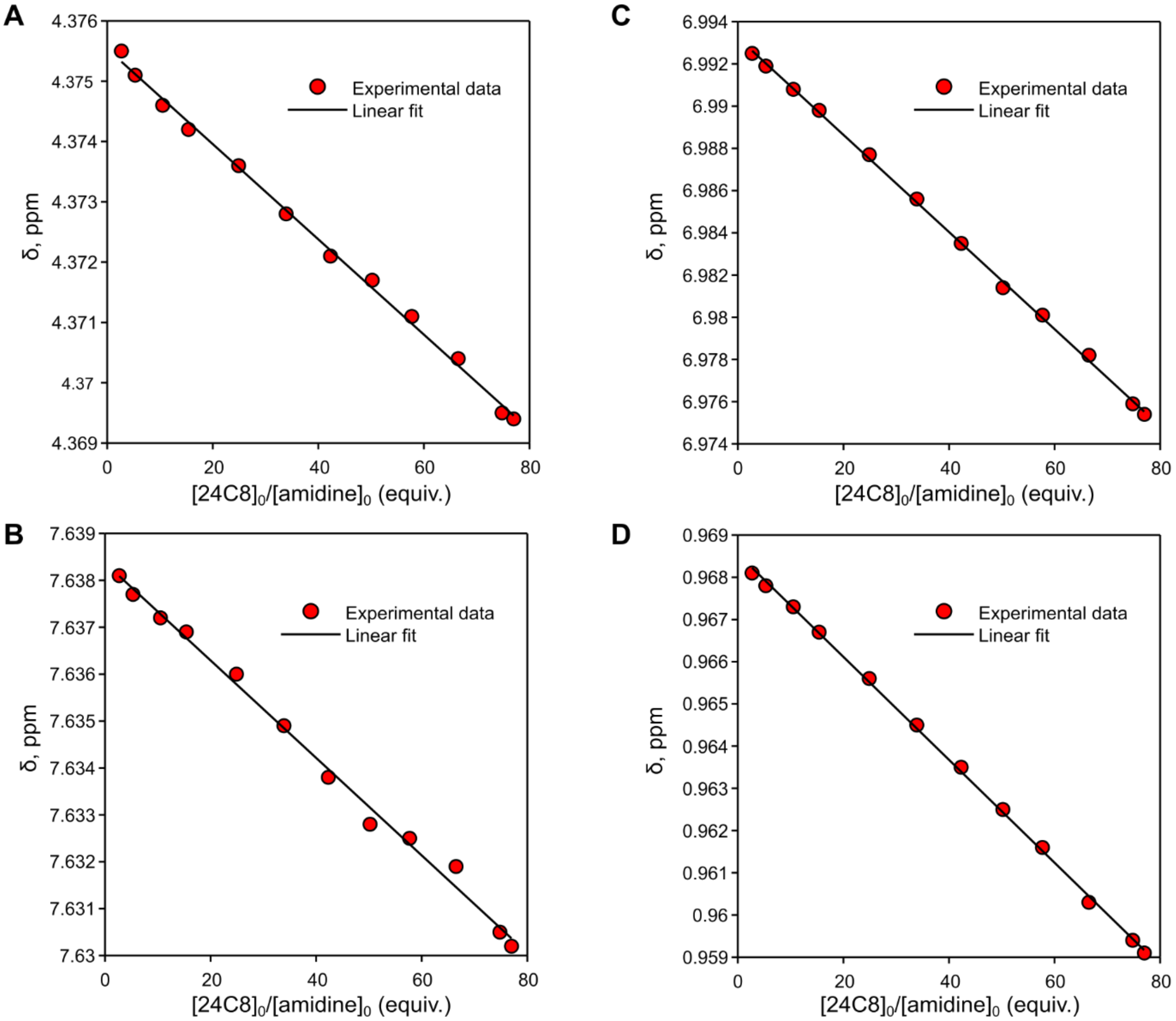

Figure S35. Changes of the chemical shift of (A) amidine benzylic CH_2 signals, (B) amidine CH signals, (C) BPh_4^- CH signals and (D) NBu_4^+ CH_3 signals upon addition of **24C8** (0 – 77 eq.) to *N*,*N'*-dibenzylformamidine (5.0 mM) in CD_3CN. The titration data could not be properly fitted to any binding model due to linear dependence of the chemical shift on the relative amount of the host species (**24C8**). Moreover, the signals from BPh_4^- and NBu_4^+ also showed linear dependence on the relative amount of **24C8** added. This evidence indicates that there is no specific binding between *N*,*N'*-dibenzylformamidine and **24C8** in CD_3CN and the observed change in chemical shift was mainly due to the change in medium polarity upon addition of **24C8** (at the end of the titration, the final content of **24C8** in the solution was 11 vol%).

6.5 Binding of 24-crown-8 to ammonium ion

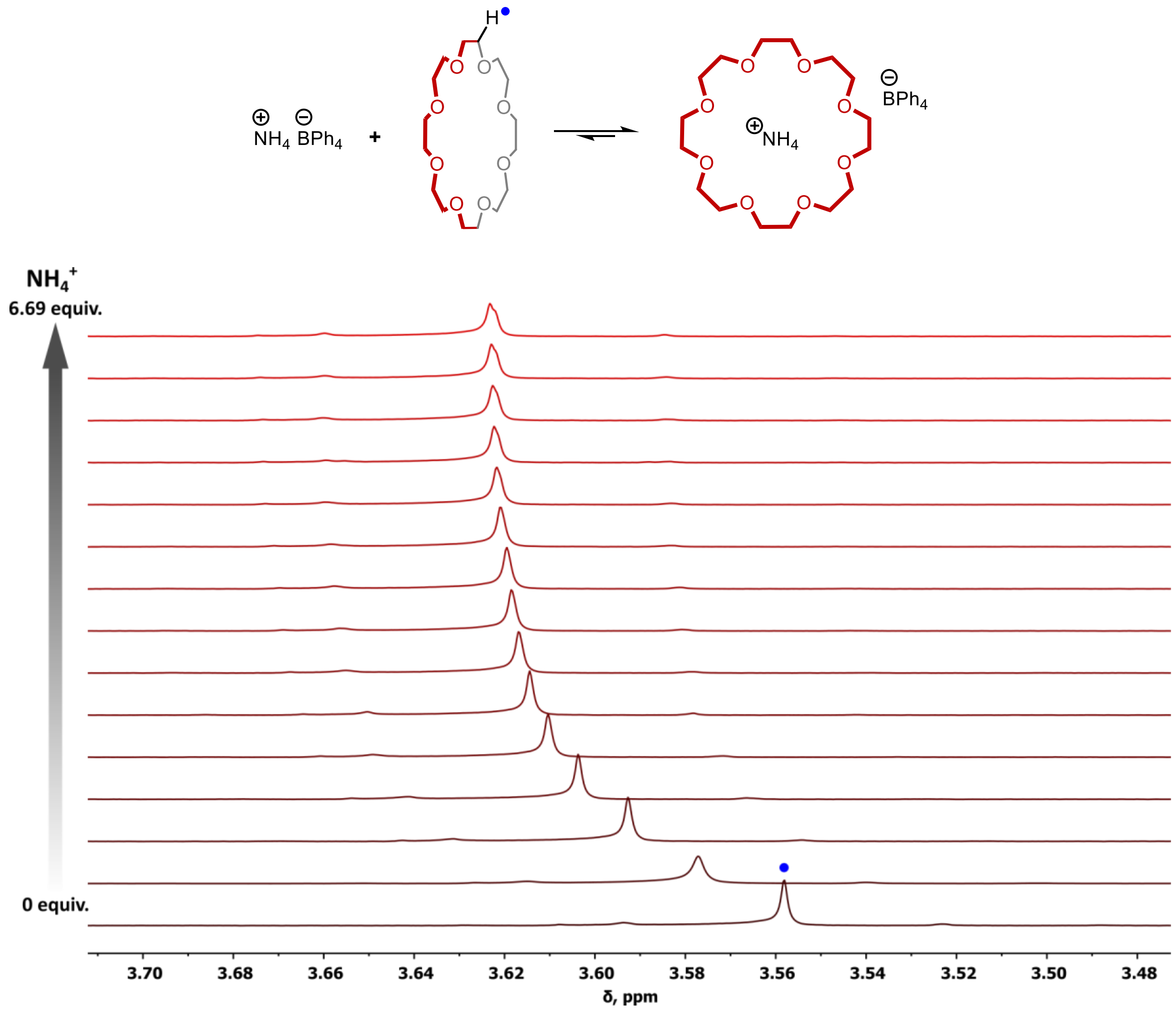

Figure S36. Representative ^{1}H NMR (500 MHz, 300 K) stack plot for titration of **24C8** (0.25 mM) with NH_4BPh_4 (0 – 6.69 equiv.) in CD_3CN. Peak asymmetry is due to poor shimming.

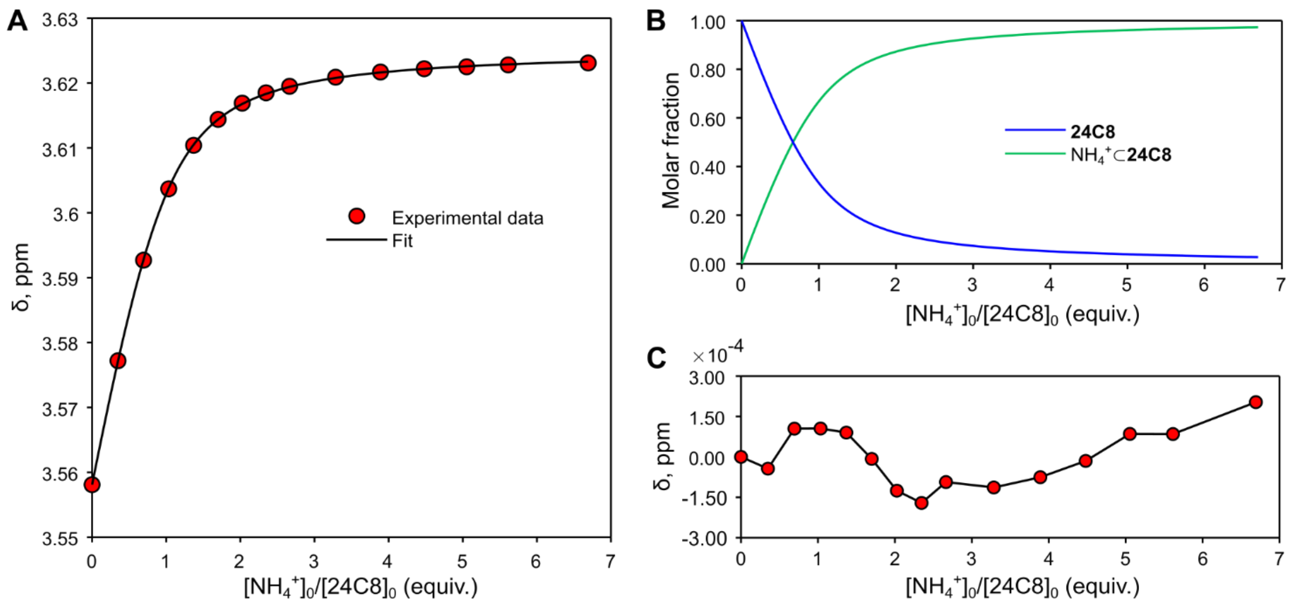

Figure S37. Representative titration data for NH_4^+⊂**24C8** host-guest system. Solvent: CD_3CN. [**24C8**]$_0$ = 0.25 mM. (A) Fit of the titration data to 1:1 binding model. (B) Dependence of molar fractions of **24C8** and NH_4^+⊂**24C8** on relative amount of NH_4BPh_4. (C) Residuals plot.

Table S12. Association constants for ammonium and **24C8** obtained by fitting the titration data to 1:1 binding model. Fit method: Nelder-Mead. Solvent: CD_3CN. Temperature: 300 K. $s/\sqrt{n}$ – standard deviation of the mean, where s – standard deviation, n – number of measurements; $t_{(0.05,3)} \times s/\sqrt{n}$ – 95% confidence interval, where $t_{(0.05,\,n\text{-}1)}$ – Student's t at 95% confidence level.[17]

[24C8]$_{tot}$, mM	K_a, M^{-1}	Fit error, %	$\overline{K}_a$, M^{-1}	$s/\sqrt{n}$, M^{-1}	$t_{(0.05,2)} \times s/\sqrt{n}$, M^{-1}
0.5	21000	± 5.5			
0.5	19300	± 5.6	21500	1460	± 6270
0.25	24300	± 1.2			

Table S13. Links to the raw titration data, calculated fits and statistical information for the fits.

[24C8]$_{tot}$, mM	*Web link*
0.5	http://app.supramolecular.org/bindfit/view/f82bbf7d-15d0-4338-91dd-a70c18ae2292
0.5	http://app.supramolecular.org/bindfit/view/a47125b1-6bbc-4a37-8fbf-4c03e6dc3f3e
0.25	http://app.supramolecular.org/bindfit/view/2a67b50f-fa9b-43e6-85c2-5ee7e713b8c9

7. Studies of the reaction pathway of the amidinium [2]rotaxanes self-assembly

All reactions described in this section were performed according to the general procedure (see Section 3, "General synthesis procedure") and were monitored by LCMS. HPLC yields and compositions of the reaction mixtures were calculated using calibration curves (Figure S38); internal standard – 1,2,4,5-tetramethylbenzene.

In case of kinetic studies at elevated temperature, the reactions were performed in 0.4 mL HPLC vial inserts placed inside screw-cap 1.5 mL HPLC vials; PhMe (400 µL) was present in the space between the insert and HPLC vial walls to provide better heat transfer between an oil/sand bath and the vial insert. Samples for LCMS analysis were prepared by unscrewing a cap of the HPLC vial, taking an aliquot (1 µL) from the reaction mixture with an Eppendorf pipette and tightly closing the vial. The aliquote was then diluted with 1 mL MeCN and subjected to LCMS analysis.

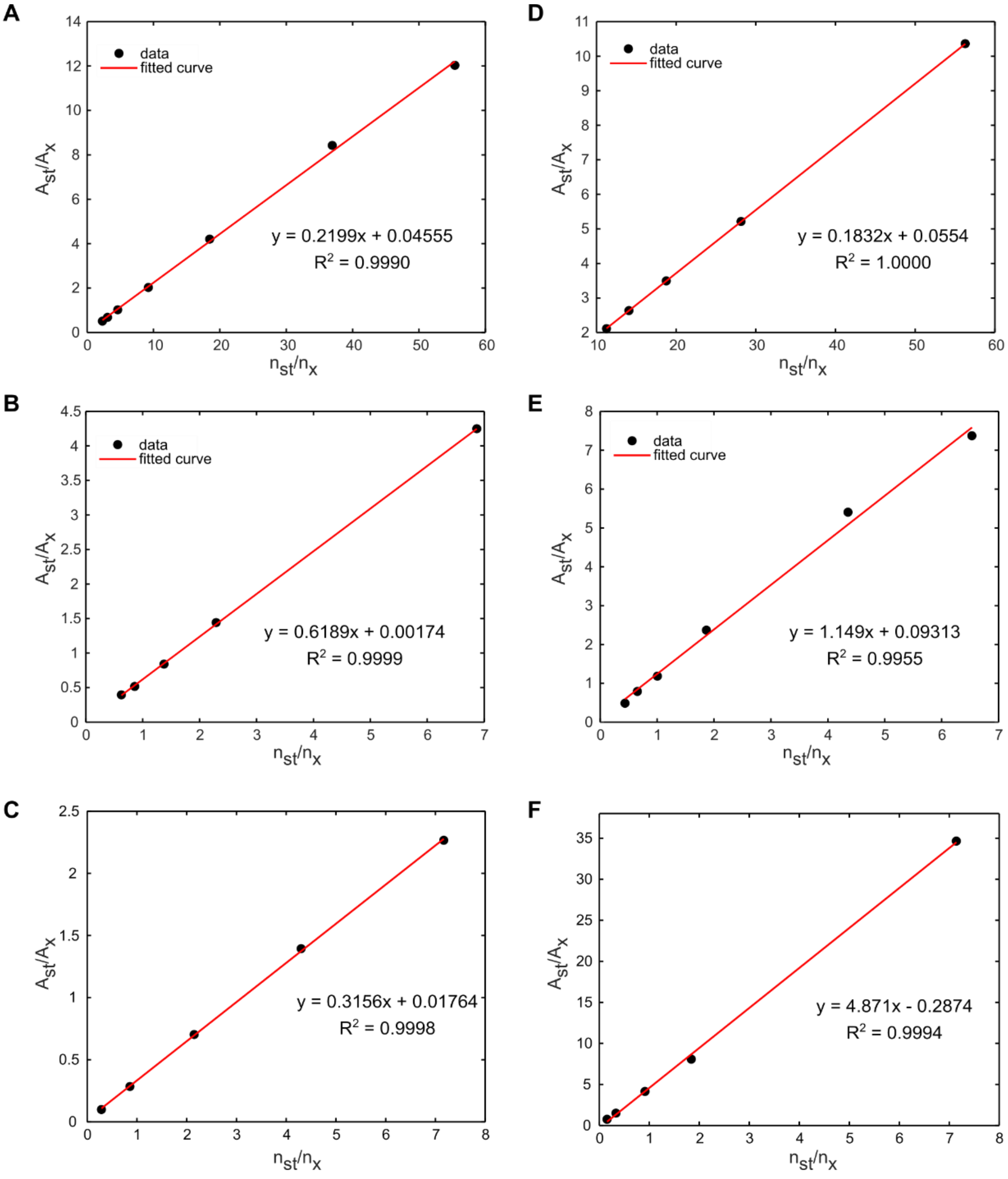

Figure S38. HPLC calibration curves for (A) thread **2a**, (B) half-thread **3a**, (C) *N*,*N*'-dibenzylformamidinium **S1**, (D) rotaxane **1a**, (E) amine **4a**, (F) benzylamine. Internal standard – 1,2,4,5-tetramethylbenzene. n_{st}/n_x – molar ratio between the standard and the calibrated compound; A_{st}/A_x – ratio of chromatographic peak areas of the standard and the calibrated compound.

7.1. Kinetic studies of the self-assembly of [2]rotaxanes via amidinium exchange

7.1.1 Rotaxane self-assembly starting from FA·BPh$_4$

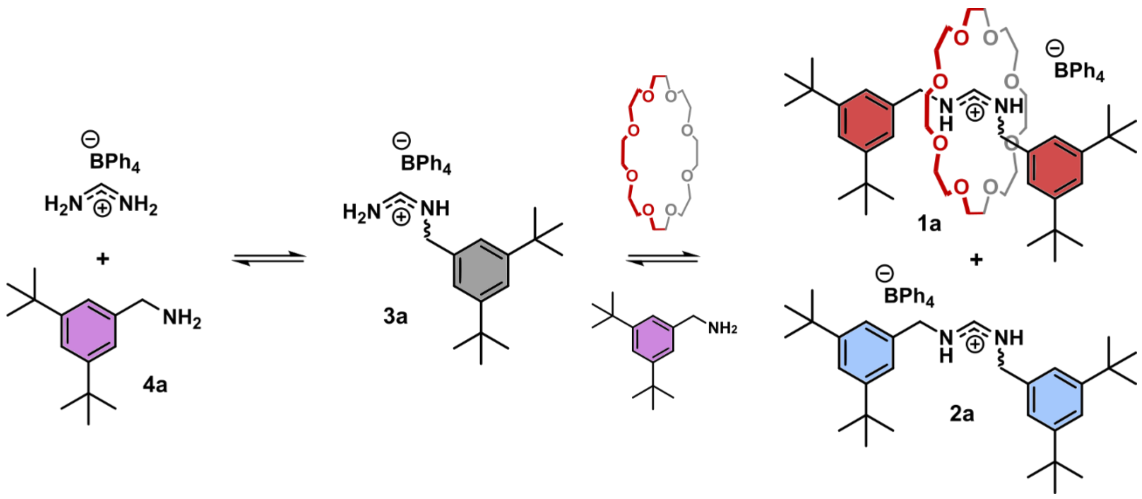

Scheme S4. Simplified reaction pathway towards rotaxane **1a** starting from **FA·BPh$_4$** and amine **4a**.

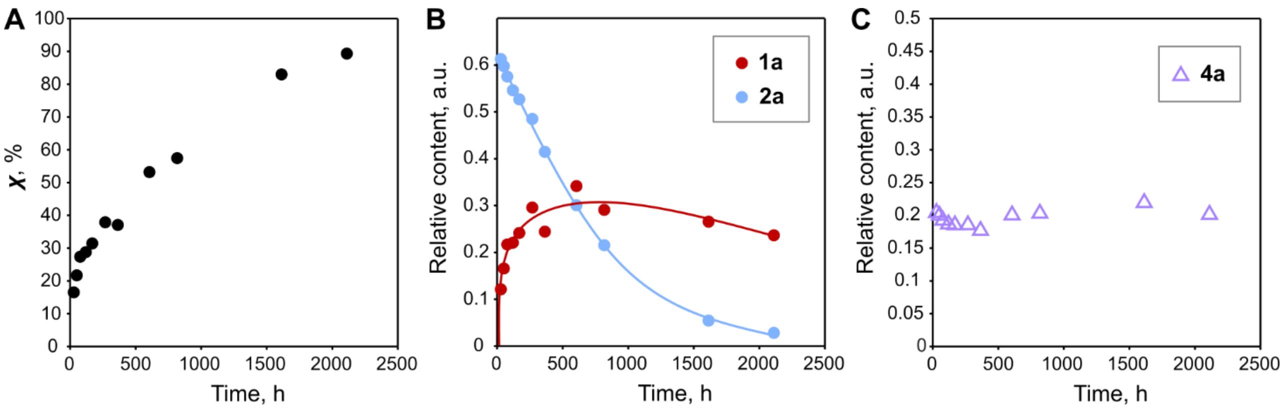

Figure S39. LCMS monitoring of the formation of rotaxane **1a** from **FA·BPh$_4$** and amine **4a** *at room temperature* over long time. Reaction conditions: 1.0 eq. **FA·BPh$_4$** (12.8 mg, 35 µmol, 0.2 M), 2.0 eq. **24C8** (24.7 mg, 70 µmol), 3.0 eq. amine **4a** (23.0 mg, 105 µmol); solvent –THF (150 µL); r.t. (A) Change of rotaxane molar percentage ***χ*** (for definition, see Section 3) over time. (B) Change of the relative content of **1a** and **2a** in the reaction mixture over time. The lines are shown to guide the eye. After 2111 h, NMR yield of **1a** based on **24C8** was 32% (16 mol% of **24C8** converted to **1a** based on integration of the crown ether CH_2 signals). (C) Change of relative content of **4a** in the reaction mixture over time. Relative content of all species (panels B and C) was calculated as a ratio between chromatographic peak areas of the species of interest and BPh_4^- as an internal standard. The data shown in the graphs suggests that even though rotaxane molar percentage ***χ*** keeps increasing, after ~500 h (20 days) decrease of the thread amount is not linked anymore to increase of the rotaxane amount. We attribute this to slow degradation (hydrolysis?) of the thread over extended periods of time, which clearly showcases much higher stability of **1a** compared to **2a**.

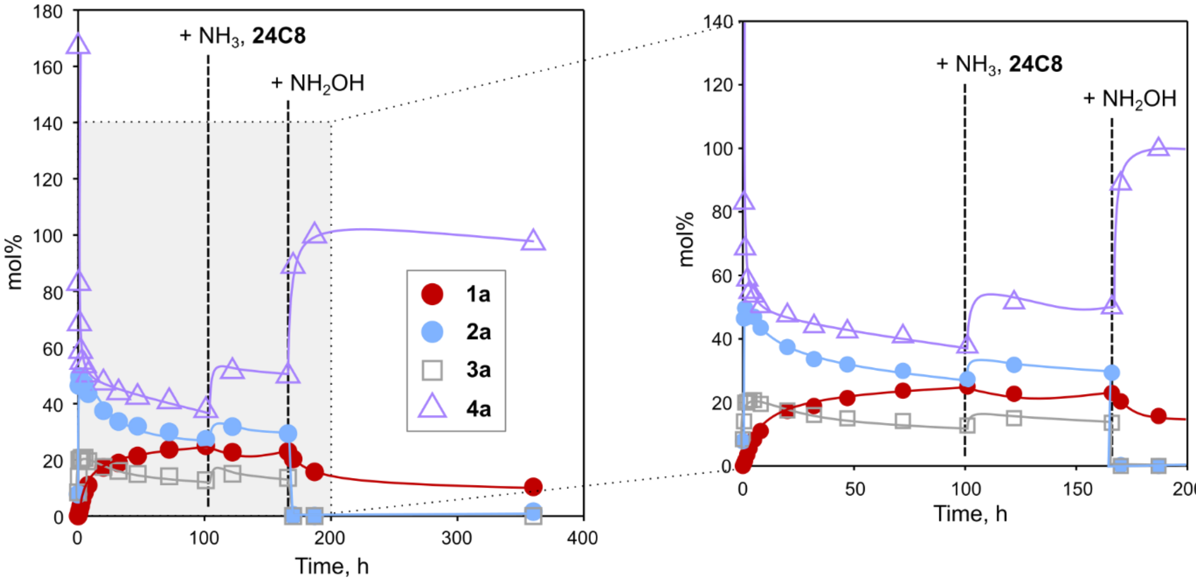

Figure S40. LCMS monitoring of the rotaxane **1a** self-assembly from **FA·BPh$_4$** and amine **4a** *at elevated temperature*. Reaction conditions: 1.0 eq. **FA·BPh$_4$** (13.5 mg, 37 µmol, 0.2 M), 2.0 eq. **24C8** (26.2 mg, 74 µmol), 2.0 eq. amine **4a** (16.3 mg, 74 µmol); solvent – PhMe/THF (4:6 v/v, total volume – 160 µL); oil bath temperature – 70 °C. After 101 h, **24C8** (0.3 eq. – amount that had been consumed to form **1a**) and NH_3 (0.8 eq., as 0.5 M solution in THF) were added. After next 65 h, NH_2OH (0.5 eq., 50% solution in H_2O) was added. Amounts of **1a**, **2a**, **3a** and **4a** (in mol% with respect to the initial amount of **FA·BPh$_4$**) were determined using 1,2,4,5-tetramethylbenzene as an internal standard. The lines are shown to guide the eye.

7.2.2. Conversion of the amidinium free thread to the rotaxane

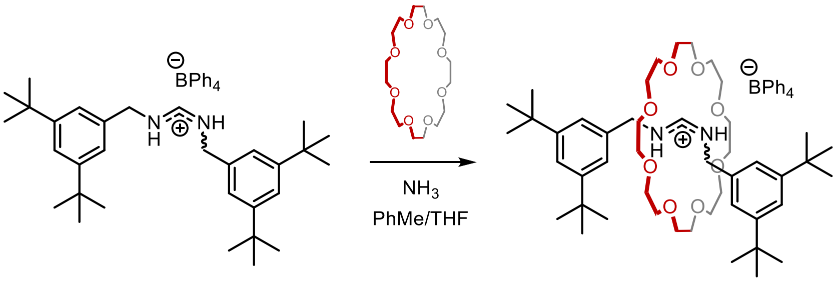

Scheme S5. Synthesis of rotaxane **1a** starting from **2a**, NH_3 and **24C8** used for kinetic studies (Figure S41).

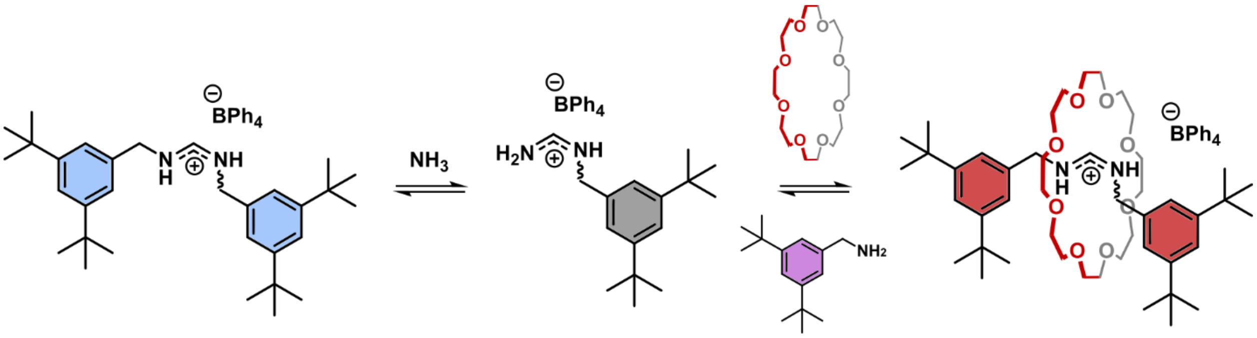

Scheme S6. Simplified reaction pathway towards rotaxane **1a** starting from thread **2a** and NH_3.

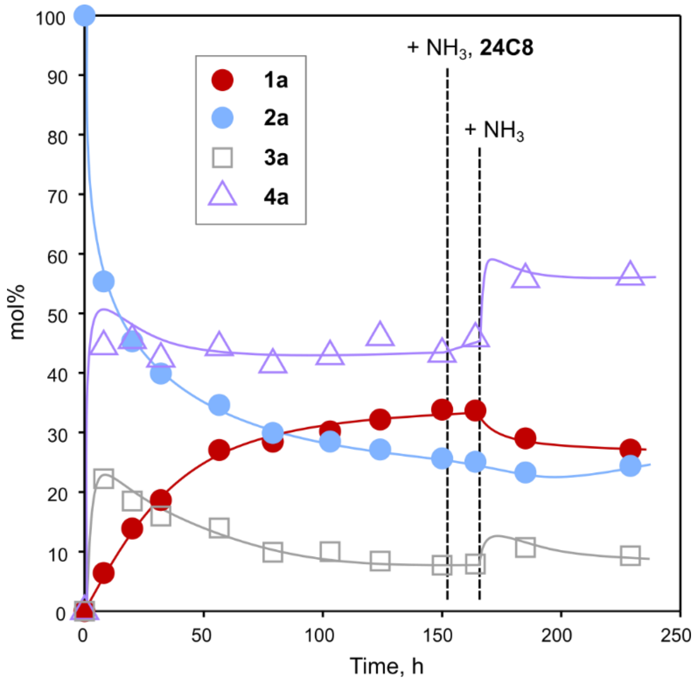

Figure S41. LCMS monitoring of the rotaxane **1a** self-assembly from **2a** and NH_3 *at elevated temperature*. Reaction conditions: 1.0 eq. **2a** (9.5 mg, 12 µmol, 0.2 M), 2.6 eq. **24C8** (11.1 mg, 31 µmol), 2.0 eq. NH_3 (0.5 M solution in THF, 47 µL); solvent – PhMe/THF (4:6 v/v); oil bath temperature – 70 °C. After 150 h, **24C8** (0.2 eq.) and NH_3 (0.2 eq., as 0.5 M solution in THF) were added. After next 14 h, NH_3 (0.7 eq., as 0.5 M solution in THF) was added again. Every time the reaction mixture was taken for LCMS analysis, the screw-cap HPLC vial was kept open (for 10–15 seconds) and small amount of NH_3 could escape the reaction vessel. Amounts of **1a**, **2a**, **3a** and **4a** (in mol% with respect to the initial amount of **2a**) were determined using 1,2,4,5-tetramethylbenzene as an internal standard. The lines are shown to guide the eye.

7.2. Influence of NH_3 release/addition during the reaction on the rotaxane yield

7.2.1. NH_3 release during the rotaxane synthesis

We hypothesized that one of the key driving forces of the rotaxane formation was release of NH_3 from the reaction system. Therefore, we decided to check if releasing NH_3 during the reaction (by regularly opening the reaction vessel) would facilitate rotaxane formation. Indeed, whenever we regularly opened screw-cap HPLC vials, where the rotaxane synthesis was carried out, we could achieve higher rotaxane yields compared to syntheses, where the HPLC vials were kept tightly closed all the time (Table S14).

Table S14. Content of rotaxane **1a** and thread **2a** in the reaction mixture of the rotaxane synthesis starting from **FA·BPh₄** and amine **4a**. For both entries, the reagent and solvent amounts as well as the temperature were the same as in the experiment from Figure S40. The only difference in the reaction conditions is indicated in the table. Amounts of **1a** and **2a** were determined using 1,2,4,5-tetramethylbenzene as an internal standard. The reactions were performed in duplicates (hence entries a and b).

Entry	Difference in reaction conditions	Thread, mol%	Rotaxane, mol%
1[a]	The reaction vessel was *tightly closed* during the reaction and opened for LCMS	38	20
1[b]	analysis only after 100 h.	38	20
2[a]	The reaction vessel was *regularly opened* to release NH_3 and take an aliquot of the	32	33
2[b]	reaction mixture for HPLC analysis.	32	30

7.2.2. NH_3 addition during the rotaxane synthesis

Knowing that NH_3 is important for conversion of the amidinium threads to the amidinium rotaxanes and taking into account that release of NH_3 from the reaction system also facilitates rotaxane formation, we decided to check if pulsed addition of substoichiometric amounts of NH_3 would result in increased yield of the rotaxane. When we started from a reaction mixture of the synthesis of **1a**, where equilibrium concentrations of **1a** and **2a** are mostly reached and substantial amount of NH_3 is removed from the reaction due to regular opening of the reaction vessel (Figure S40, ≈ 100 h), and regularly added NH_3 (0.5 M solution in THF), the amount of **1a** indeed slightly increased, but then remained unchanged (Figure S42). However, amount of **2a** constantly decreased, which was due to 1) conversion of **2a** into **3a** and 2) slow degradation of **2a** or **3a** (e.g., hydrolysis).

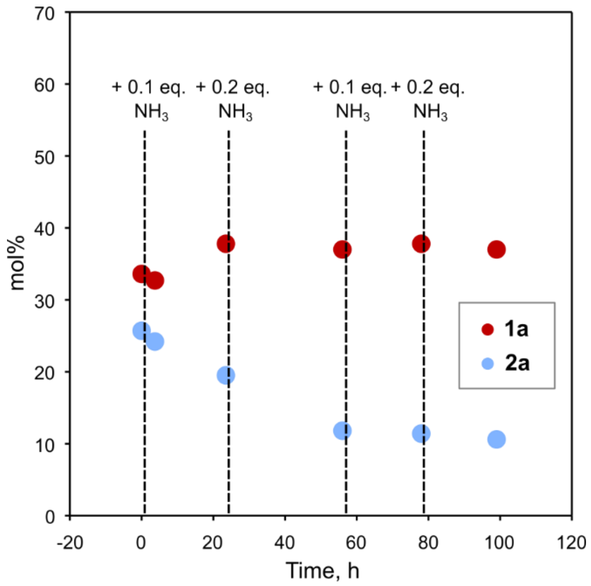

Figure S42. LCMS monitoring of the rotaxane **1a** self-assembly using pulsed additions of substoichiometric amounts of NH_3. Reaction conditions: 1.0 eq. **FA·BPh$_4$** (13.5 mg, 37 µmol, 0.2 M), 2.0 eq. **24C8** (26.2 mg, 74 µmol), 2.0 eq. amine **4a** (16.3 mg, 74 µmol); solvent – PhMe/THF (4:6 v/v, total volume – 160 µL); bath temperature – 70 °C. Time 0 h on the graph corresponds to 100 h after the standard synthesis (as in Figure S40). NH_3 was added as 0.5 M solution in THF. The reaction mixture was stirred at 70 °C (T_{bath}). A needle was inserted into the screw-cap HPLC vial cap in order to allow slow release of NH_3. Fresh portions of THF were occasionally added to maintain constant volume of the reaction mixture. Amounts of **1a** and **2a** (in mol% with respect to the initial amount of **FA·BPh$_4$**) were determined using 1,2,4,5-tetramethylbenzene as an internal standard.

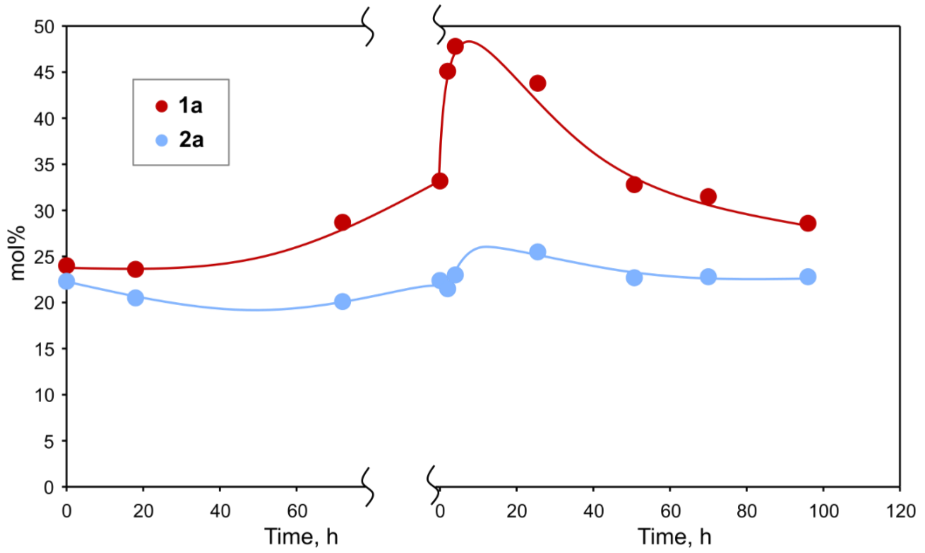

Figure S43. LCMS monitoring of the rotaxane **1a** self-assembly using $(Me_3Si)_2NH$ as a source of NH_3. Reaction conditions: 1.0 eq. **FA·BPh4** (13.5 mg, 37 µmol, 0.2 M), 2.0 eq. **24C8** (26.2 mg, 74 µmol), 2.0 eq. amine **4a** (16.3 mg, 74 µmol); solvent – PhMe/THF (4:6 v/v, total volume – 160 µL); bath temperature – 70 °C. Time 0 h on the left side of the graph corresponds to 100 h after the rotaxane synthesis in a tightly closed screw-cap vial and 1 day of stirring at 70 °C open air (the reaction mixture from Entry 1[a] in Table S14 was used). At first, 0.2 eq. $(Me_3Si)_2NH$ (1.6 µL) were added and the reaction mixture was stirred at 70 °C (T_{bath}) in a closed vial. After 72 h, the reaction mixture was cooled down to r.t. and kept for 6 days w/o stirring (break in the graph corresponds to this period). *At the second time 0 h*, 5 eq. $(Me_3Si)_2NH$ (40 µL) were added and the reaction mixture was stirred at 70 °C (T_{bath}) in a closed vial for 96 h. Amounts of **1a** and **2a** (in mol% with respect to the initial amount of **FA·BPh4**) were determined using 1,2,4,5-tetramethylbenzene as an internal standard. The lines are shown to guide the eye.

Conclusion. Addition of $(Me_3Si)_2NH$ did improve the rotaxane yield, however, prolonged stirring of the reaction mixture with excess of $(Me_3Si)_2NH$ led to decrease of the rotaxane amount. Even though it is not fully clear what caused the observed effect – released NH_3, change in the medium polarity (due to $(Me_3Si)_2NH$) or something else – this experiment demonstrates that further improvement of the yields of the amidinium rotaxanes is potentially possible.

7.3. Rotaxane yield dependence on formamidinium salt, crown ether and amine

7.3.1. DPFA as an exchange substrate

Initial attempts to synthesize amidinium [2]rotaxanes using *N,N'*-diphenylformamidinium (**DPFA**) (Scheme S7) were performed at room temperature (anion: BF_4^-). The reaction proceeds through the formation of the mono-exchange product (half-thread and semirotaxane, Scheme S8) and subsequent formation of two-fold exchange product (thread and/or rotaxane, Scheme S8). We observed fast formation of the mono-exchange product and slower formation of the thread, however, there was only trace amount of the rotaxane (Figure S44). This can be attributed to very low reactivity of the semirotaxane, where the amidinium electrophilic carbon is sterically hindered by the crown ether and, additionally, by the phenyl ring. Therefore, we carried out the rotaxane synthesis at elevated temperatures. This time we were able to observe the rotaxane formation, but in extremely low yields (Table S15, entries 2 and 3). We hypothesized that the anion – BF_4^- – might also be a reason for the low yields of rotaxane **1a**: BF_4^- can potentially compete with **24C8** for the binding to the amidinium moiety,[19] thus impeding formation of the semirotaxane. Indeed, with **DPFA·BArF** the reaction afforded a tangible amount of **1a**; however, the yield was still much lower than in case of **FA·BPh4**. Interestingly, the rotaxane molar percentage χ (see Section 3 for the definition) was 63% after 9 days (Table S15, Figure S45). This reflects higher thermodynamic stability of the rotaxane in comparison to the free thread. However, the total HPLC yield of both thread **2a** and rotaxane **1a** was only 22% (on the 9th day). This might indicate that both **DPFA** and the half-thread are kinetically and thermodynamically stabilized due to strong binding to **24C8**. In addition, the reaction by-product – aniline – is always present in the system

and cannot strongly bind to **24C8**. This means that aniline can also participate in the amidinium exchange, thus shifting the overall equilibrium towards starting materials.

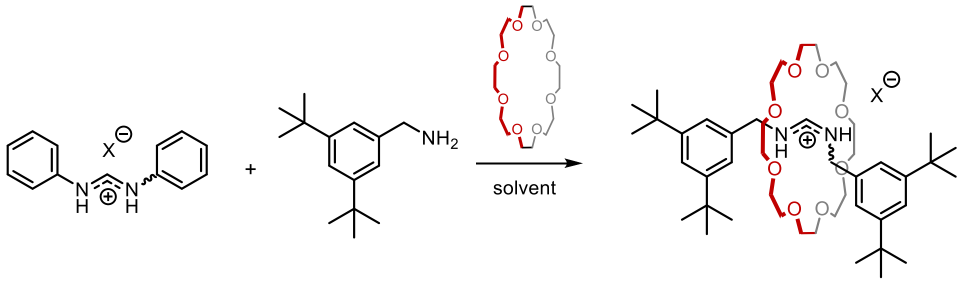

Scheme S7. Amidinium rotaxane synthesis starting from **DPFA**.

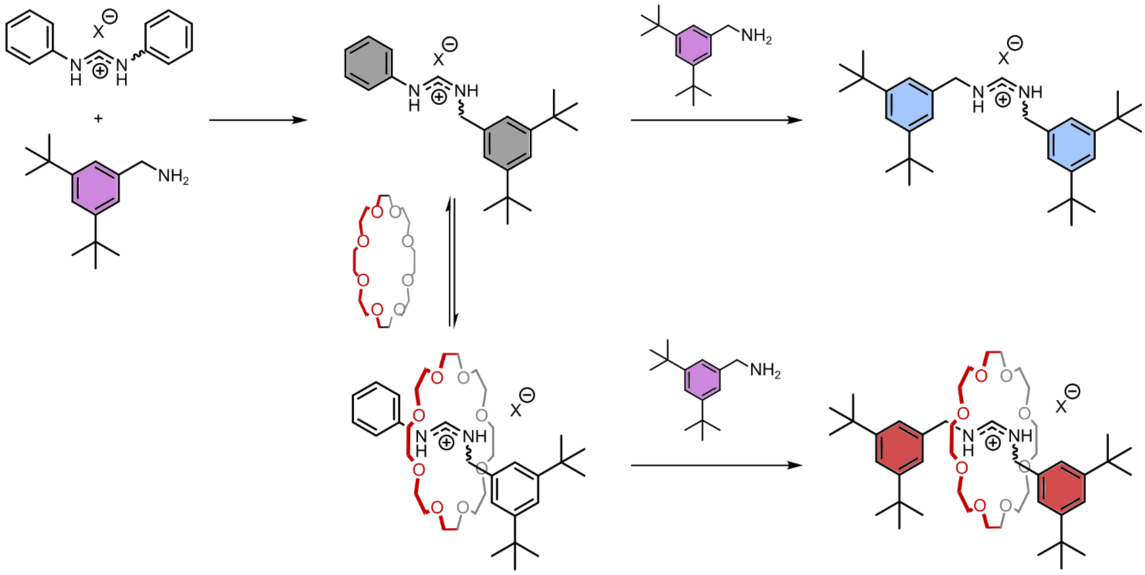

Scheme S8. Reaction pathway of the amidinium rotaxane synthesis using **DPFA** as an exchange substrate.

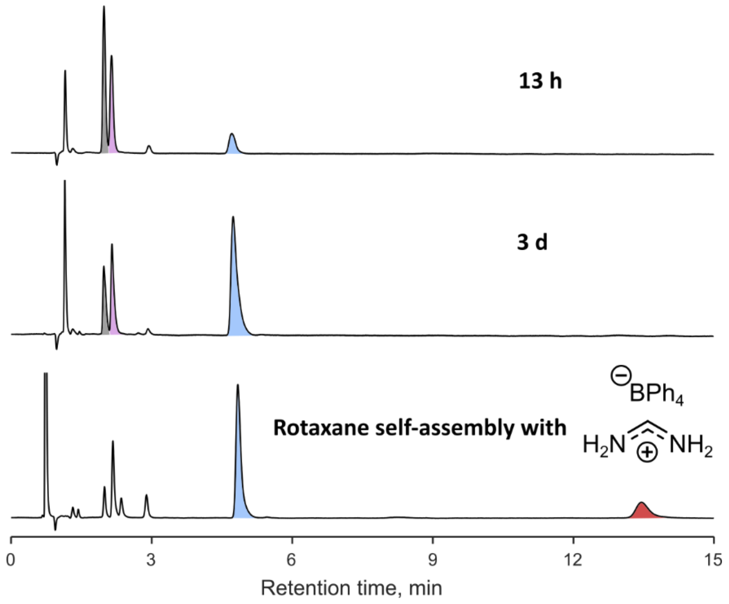

Figure S44. HPLC chromatograms of the attempted rotaxane self-assembly using *N*,*N'*-diphenylformamidinium tetrafluoroborate (**DPFA·BF₄**) as an exchange substrate. Reaction conditions: 1.0 eq. **DPFA·BF₄** (0.2 M), 3.0 eq. amine **4a**, 2.0 eq. **24C8**; solvent – THF; r.t. For comparison, an HPLC chromatogram of the successful rotaxane self-assembly is shown (bottom). For color code, see Scheme S8.

Table S15. Summary of attempted amidinium rotaxane syntheses using **DPFA** as an exchange substrate. HPLC yield was determined on the 3rd day using 1,2,4,5-tetramethylbenzene as an internal standard.

Entry	Temperature, °C	Solvent	Anion	Amine amount, equiv.	24C8 amount, equiv.	HPLC yield, %
1	23	THF	BF_4^-	3.0	2.0	trace amount
2	80	PhMe	BF_4^-	3.0	2.0	<5
3	65	PhMe/THF*	BF_4^-	2.0	2.0	<5
4	70	PhMe/THF*	$BArF^-$	2.0	2.0	9**

*4:6 by volume.
**After 9 days, the rotaxane yield was 14% and rotaxane molar percentage χ (see Section 3 for the definition) was 63%.

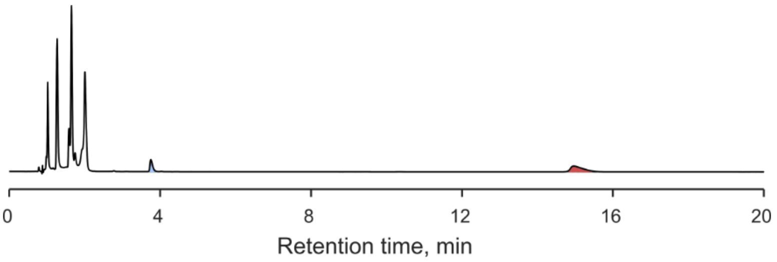

Figure S45. HPLC chromatogram of the rotaxane self-assembly (9th day) using *N*,*N'*-diphenylformamidinium tetrakis[3,5-bis(trifluoromethyl)phenyl]borate (**DPFA·BArF**) as an exchange substrate. Reaction conditions: 1.0 eq. **DPFA·BArF** (0.2 M), 2.0 eq. amine **4a**, 2.0 eq. **24C8**; solvent – PhMe/THF (4:6 by volume); 70 °C. For color code, see Scheme S8. **DPFA·BArF** was prepared from **DPFA·BF₄** according to the general procedure for ion exchange (Section 4).

7.3.2. DMFA as an exchange substrate

Amidinium rotaxane synthesis starting from **DMFA·BPh₄** (Scheme S9) either at room or elevated temperature was not successful (Table S16). For entry 2 (Table S16), composition of the key reaction species on the 3rd day of the reaction was the following: 1 mol% rotaxane **1a**, 8 mol% thread **2a**, 35 mol% half-thread (product of monoexchange) and 111 mol% of amine **4a** (all molar percentages with respect to the initial amount of **DMFA·BPh₄**). Release of $MeNH_2$ from the reaction mixture did not significantly improve the rotaxane yield (Table S17). We suggest two possible reasons for these observations. First, *N*,*N'*-dialkylformamidinium ions are less reactive toward benzylamines than unsubstituted formamidinium ions (Figure S47). This leads to low expected yields even of the non-interlocked product of the double exchange (**2a**) (hence, high amount of the unreacted amine). Second, in thermodynamical sense, **DMFA** and **2a** are very similar in terms of binding to **24C8** (the binding motif in both amidinium ions is virtually the same; the only difference between crown ether complexes of **DMFA** and **2a** is that in the latter case **24C8** is kinetically trapped). Since the rotaxane self-assembly by the amidinium exchange is under thermodynamic control, formation of substantial amounts of **1a** from **DMFA** is rather unlikely. We confirmed the fact that the reaction system operates under thermodynamic control by obtaining equilibrium mixtures of similar composition starting either from **DMFA**, **24C8** and **4a** or from **1a** and $MeNH_2$ (Scheme S9 and S10, Table S17 and S18).

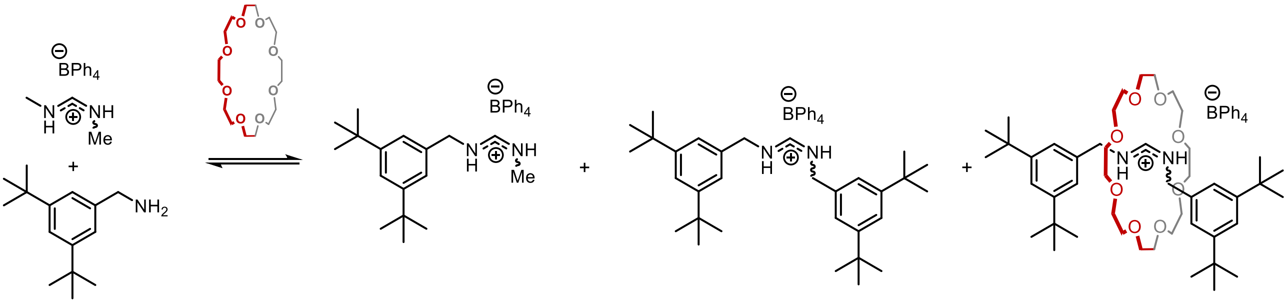

Scheme S9. Amidinium rotaxane synthesis starting from **DMFA**.

Table S16. Summary of amidinium rotaxane syntheses using *N,N'*-dimethylformamidinium tetraphenylborate (**DMFA·BPh$_4$**) as an exchange substrate. HPLC yield was determined on the 3rd day using 1,2,4,5-tetramethylbenzene as an internal standard.

Entry	Temperature, °C	Solvent	Amine amount, equiv.	24C8 amount, equiv.	HPLC yield, %
1	23	THF	3.0	2.0	<5
2	70	PhMe/THF*	2.0	2.0	<5**

*4:6 by volume.

Rotaxane molar percentage ***χ* (see Section 3 for the definition) was 10%.

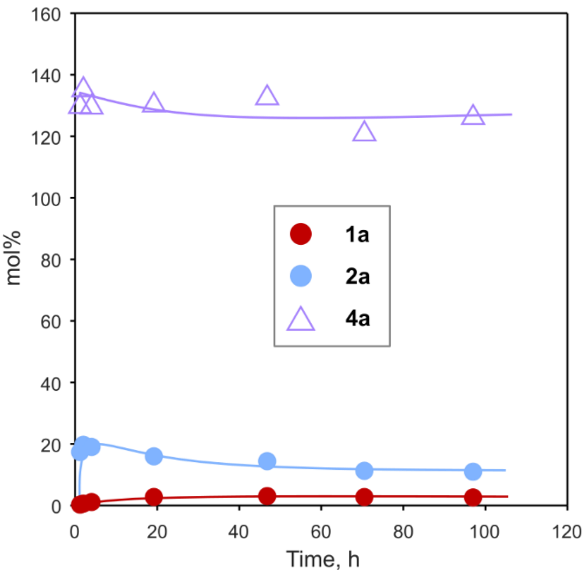

Figure S46. LCMS monitoring of the rotaxane **1a** self-assembly from **DMFA·BPh$_4$** and amine **4a** *at elevated temperature*. Reaction conditions: 1.0 eq. **DMFA·BPh$_4$** (14.5 mg, 37 µmol, 0.2 M), 2.0 eq. **24C8** (26.2 mg, 74 µmol), 2.0 eq. amine **4a** (16.3 mg, 74 µmol); solvent – PhMe/THF (4:6 v/v, total volume – 160 µL); bath temperature – 70 °C. Every time the reaction mixture was taken for LCMS analysis, the screw-cap HPLC vial was kept open (for 10–15 seconds) and small amount of $MeNH_2$ could escape the reaction vessel. Amounts of **1a**, **2a**, and **4a** (in mol% with respect to the initial amount of **DMFA·BPh$_4$**) were determined using 1,2,4,5-tetramethylbenzene as an internal standard. The lines are shown to guide the eye.

Table S17. Content of rotaxane **1a**, thread **2a**, half-thread and amine **4a** in the reaction mixture of the rotaxane synthesis starting from **DMFA·BPh$_4$** after 97 h. For both entries, the reagent and solvent amounts as well as the temperature were the same as in the experiment from Figure S40. The only difference in the reaction conditions is indicated in the table. Amounts of **1a**, **2a**, and **4a** (in mol% with respect to the initial amount of **DMFA·BPh$_4$**) were determined using 1,2,4,5-tetramethylbenzene as an internal standard.

Entry	Difference in reaction conditions	Amine, mol%	Half-thread, mol%*	Thread, mol%	Rotaxane, mol%
1	The reaction vessel was *tightly closed* during the reaction and opened for LCMS analysis only after 97 h.	116	36	8	1
2	The reaction vessel was *regularly opened* to release $MeNH_2$ and take an aliquot of the reaction mixture for HPLC analysis.	126	47	11	3

*The product of monoexchange, i.e. the amidinium ion containing both amine **4a** and $MeNH_2$ (Scheme S9). Approximate amounts of this product were calculated using the calibration curve for **3a**.

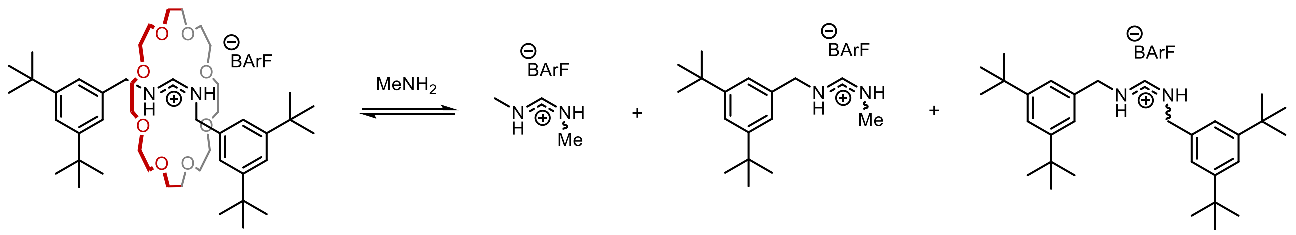

Scheme S10. Reaction of rotaxane **1a** with $MeNH_2$.

Table S18. Content of rotaxane **1a**, thread **2a**, half-thread and amine **4a** in the reaction mixture obtained from rotaxane **1a** and $MeNH_2$ (see Scheme S10 above). Reaction conditions: 1.0 eq. **1a·BArF** (10 mg, 6 µmol, 0.2 M), 1.0 eq. **24C8** (2.1 mg, 6 µmol), 2.1 eq. $MeNH_2$ (2.0 M solution in THF, 6.3 µL); solvent – PhMe/THF (4:6 v/v, total volume – 26 µL); bath temperature: 70 °C.

Time, h	Amine, mol%	Half-thread, mol%*	Thread, mol%	Rotaxane, mol%
97	123	47	7	9
118	115	40	5	9

*The product of monoexchange, i.e. the amidinium ion containing both amine **4a** and $MeNH_2$ (Scheme S9). Approximate amounts of this product were calculated using the calibration curve for **3a**.

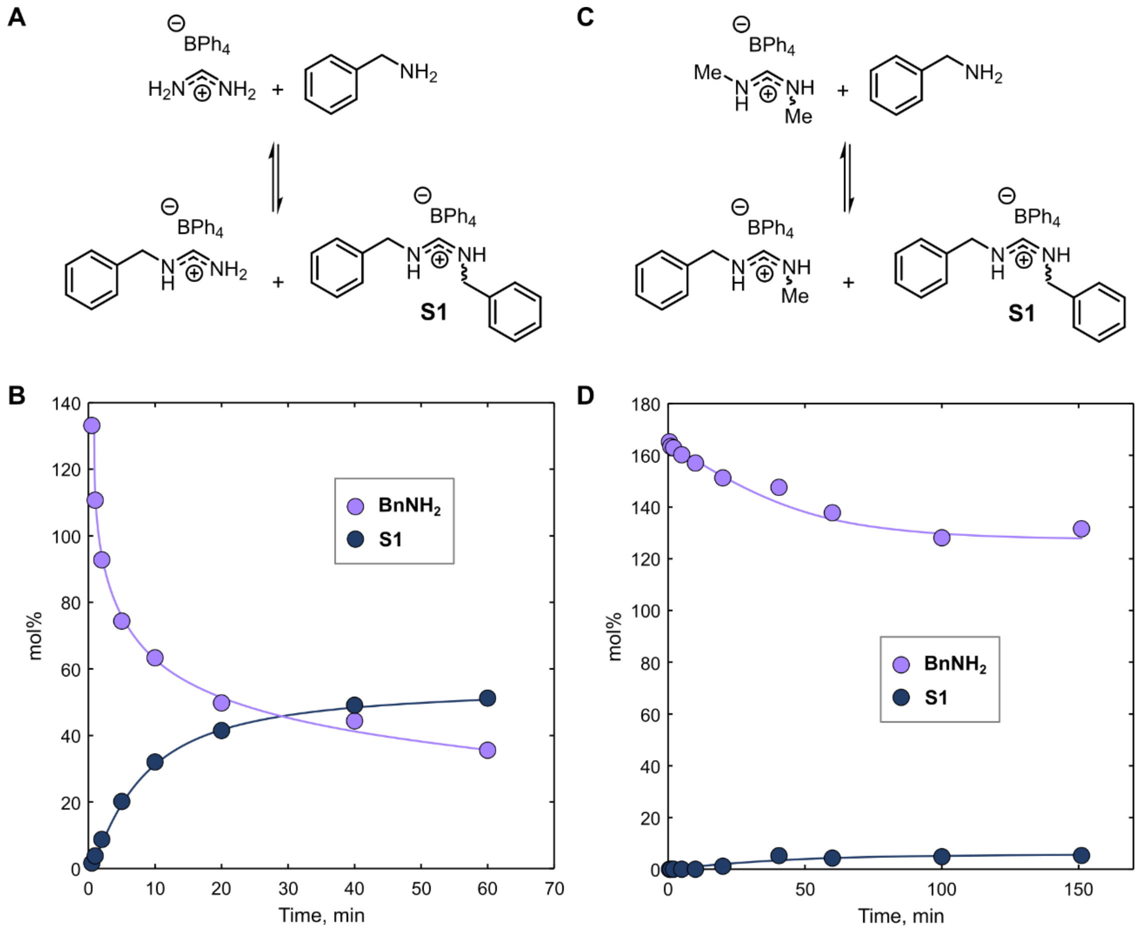

Figure S47. Comparison of the reaction rate and equilibrium composition of two amidinium exchange reactions: (A,B) **FA·BPh₄** + $BnNH_2$ and (C,D) **DMFA·BPh₄** + $BnNH_2$. Reaction conditions: (A,B) 1.0 eq. **FA·BPh₄** (11.0 mg, 30.2 µmol, 50 mM), 2.0 eq. $BnNH_2$ (6.5 mg, 60.4 µmol), solvent – THF/PhMe (480 µL / 120 µL), r.t.; (B,C) 1.0 eq. **DMFA·BPh₄** (9.9 mg, 25.2 µmol, 50 mM), 2.0 eq. $BnNH_2$ (5.4 mg, 50.3 µmol), solvent – THF/PhMe (400 µL / 100 µL). The reactions were monitored by LCMS. Amounts of $BnNH_2$ and **S1** (in mol% with respect to the initial amount of **FA·BPh₄** or **DMFA·BPh₄**) were determined using 1,2,4,5-tetramethylbenzene as an internal standard. The lines are shown to guide the eye.

7.3.3. *N,N'*-dibenzylformamidinium (S1) as an exchange substrate

We also tested the exchange substrate where the amine leaving groups have nature very similar to amine stopper **4a** (Scheme S11). In this case, amount of rotaxane **1a** was extremely low even after 4 days at 70 °C. As in case of **DMFA**, the major product was the product of the monoexchange.

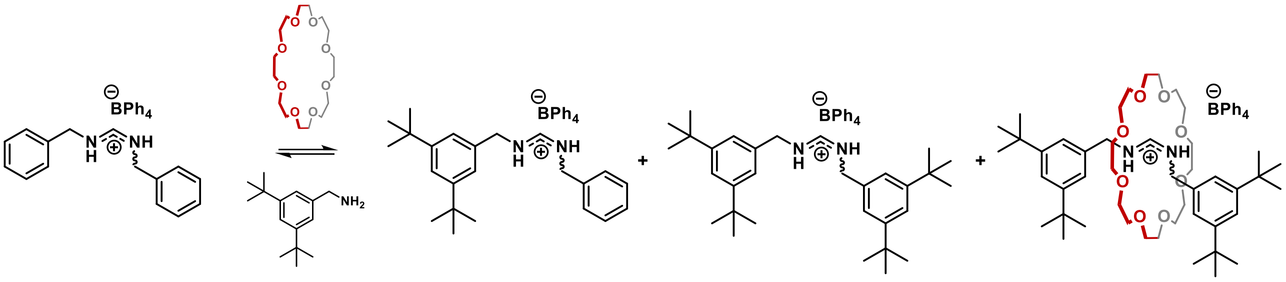

Scheme S11. Amidinium rotaxane synthesis starting from **S1**.

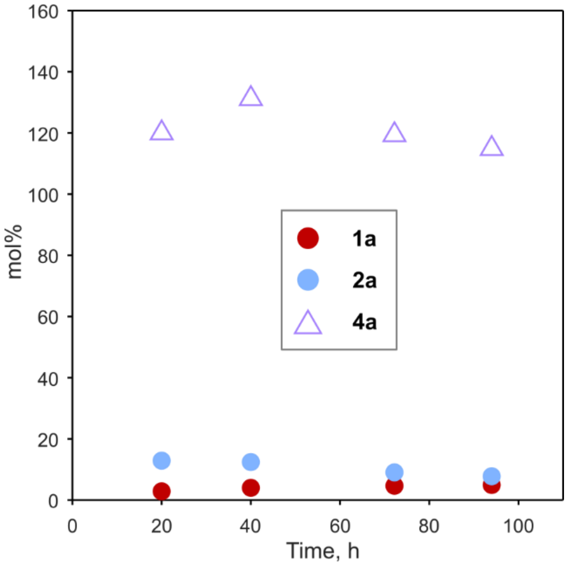

Figure S48. LCMS monitoring of the rotaxane **1a** self-assembly from **S1** (BPh_4^- salt) and amine **4a** *at elevated temperature*. Reaction conditions: 1.0 eq. **S1** (20.1 mg, 37 µmol, 0.2 M), 2.0 eq. **24C8** (26.2 mg, 74 µmol), 2.0 eq. amine **4a** (16.3 mg, 74 µmol); solvent – PhMe/THF (4:6 v/v, total volume – 160 µL); bath temperature – 70 °C. Amounts of **1a**, **2a**, and **4a** (in mol% with respect to the initial amount of **DMFA·BPh₄**) were determined using 1,2,4,5-tetramethylbenzene as an internal standard.

7.3.4. FA·OAc as an exchange substrate

To prove that coordination of the crown ether ring to the amidinium moiety is essential for success of the rotaxane formation, we conducted the reaction of the amidinium rotaxane self-assembly using formamidinium acetate (**FA·OAc**) as the exchange substrate (Scheme S12). Strongly coordinating anions can compete with **24C8** for binding to the amidinium ion and potentially prevent the rotaxane formation. We found that either at room or elevated temperature the rotaxane yield was extremely low (Table S19). We could obtain similar results when performing the rotaxane synthesis with **FA·BPh_4** in the presence of 1.0 eq. tetra-n-butylammonium acetate (NBu_4OAc). This suggests that *binding of **24C8** to the amidinium moiety plays a key role in the formation of the amidinium rotaxanes*.

To confirm the thermodynamic nature of this phenomenon, we added NBu_4OAc to the reaction mixture where substantial amount of rotaxane **1a** was already present (Figure S49). As expected, the thread was amplified while the amount of **1a** significantly decreased.

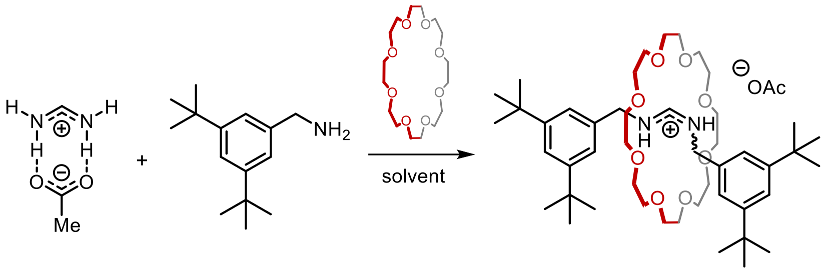

Scheme S12. Amidinium rotaxane synthesis starting from **FA·OAc**.

Table S19. Summary of amidinium rotaxane syntheses using formamidinium acetate (**FA·OAc**) as an exchange substrate. HPLC yield was determined on the 3rd day using 1,2,4,5-tetramethylbenzene as an internal standard.

Entry	Temperature, °C	Solvent	Amine amount, equiv.	24C8 amount, equiv.	HPLC yield, %
1	23	MeCN	3.0	2.0	trace amount
2	70	PhMe	2.0	2.0	<5
3	70	PhMe/THF*	2.0	2.0	trace amount**

*4:6 by volume.
HPLC yield of thread **2a after 1 day was 38%, while rotaxane was not formed at all. During the course of the reaction, amount of the thread decreased to 30%, possibly due to degradation.

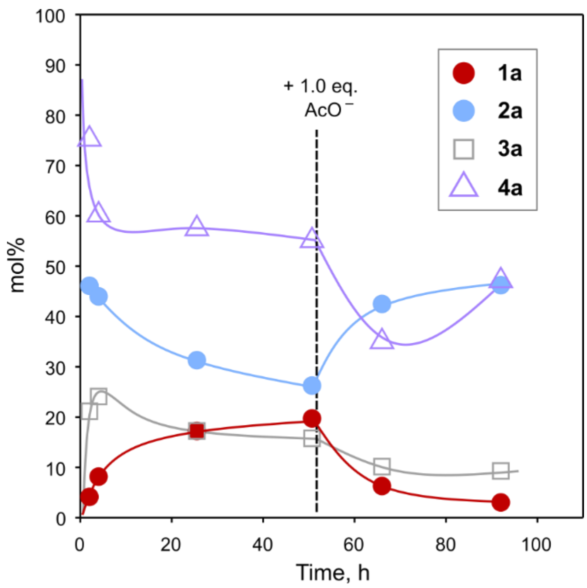

Figure S49. LCMS monitoring of the rotaxane **1a** self-assembly from **FA·BPh₄** and amine **4a** before and after addition of tetra-n-butylammonium acetate (NBu_4OAc). Reaction conditions: 1.0 eq. **FA·BPh₄** (13.5 mg, 37 µmol, 0.2 M), 2.0 eq. **24C8** (26.2 mg, 74 µmol), 2.0 eq. amine **4a** (16.3 mg, 74 µmol); solvent – PhMe/THF (4:6 v/v, total volume – 160 µL); bath temperature – 70 °C. After 51 h, solid NBu_4OAc (1.0 eq., 11.2 mg, 37 µmol; the salt was thoroughly dried before use) was added to the reaction mixture. Amounts of **1a**, **2a**, **3a** and **4a** (in mol% with respect to the initial amount of **FA·BPh₄**) were determined using 1,2,4,5-tetramethylbenzene as an internal standard. The lines are shown to guide the eye.

7.3.5. Thread 2a as an exchange substrate

Synthesis of the amidinium rotaxane starting from the free thread (Scheme S13) would prove that the key steps of the reaction pathway (i.e., reaction of the amidinium half-thread or the semirotaxane with a primary amine leading to the free thread or the rotaxane respectively; see Figure 5 in the main text) are fully reversible, thus supporting the proposed reaction pathway. As expected, when only thread **2a** and **24C8** are mixed together and stirred at elevated temperatures, virtually no rotaxane formation was observed (Table S20, Entry 4). However, addition of either amine **4a** or NH_3 led to formation of substantial amount of rotaxane **1a**. Interestingly, the most efficient method for the rotaxane synthesis starting from the free thread involved addition of NH_3. This supports our assumption about the importance of coordination of the crown ether to the half-thread (e.g., **3a**).

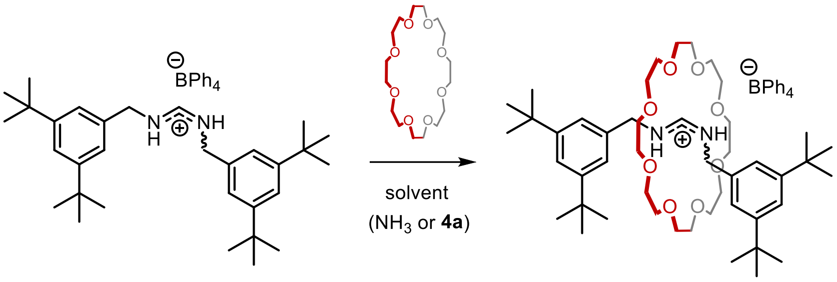

Scheme S13. Amidinium rotaxane synthesis starting from thread **2a**. In case of amine **4a** as the nucleophile, refer to Scheme S16, B.

Table S20. Summary of amidinium rotaxane syntheses using *N*,*N'*-bis(3,5-di-*tert*-butylbenzyl)formamidinium tetraphenylborate (**2a**) as an exchange substrate. Thread **2a** was an isolated compound and was prepared according to the procedure described in Section 2.4. The reactions were carried out in the presence of NH_3 *or* amine **4a** *or* neither. HPLC yields were determined using 1,2,4,5-tetramethylbenzene as an internal standard.

Entry	Temperature, °C	Solvent	Time, days	24C8 amount, equiv.	Amine amount, equiv.	NH_3 amount, equiv.	HPLC yield, %
1	80	PhMe	1	1.0	2.0	0	~8*
2†	70	PhMe/THF‡	3	2.6	0	2.0	28
3	70	PhMe/THF‡	3	2.0	1.5	0	19
4	70	PhMe/THF‡	3	2.2	0	0	<5%**

*Rotaxane molar percentage χ was 33%.

**Substantial degradation of BPh_4^- was observed.

† See Figure S41 for kinetic studies of this particular reaction.

‡4:6 by volume.

7.3.6. Electron deficient crown ether and amine

We tried to employ another commonly used crown ether, i.e. dibenzo-24-crown-8 (**DB24C8**), in the synthesis of the amidinium rotaxanes (Scheme S14). We found that on the 3rd day of the reaction at room temperature there was only trace amount of the target rotaxane. We attributed this to weaker binding of **DB24C8** to the amidinium species (since this crown ether is more electron deficient then **24C8**).

We also attempted to use 3,5-bis(trifluoromethyl)benzylamine as a stopper in the amidinium rotaxane synthesis (Scheme S15). This amine was widely used by Leigh, Fielden and co-workers in their recently developed metal-free active template approach.[20] Unfortunately, we did not observe any rotaxane formation at room temperature (even after many days of continuous stirring), which is probably due to poor

nucleophilicity of the amine that in turn has direct impact on its ability to undergo the amidinium exchange with **FA·BPh₄**.

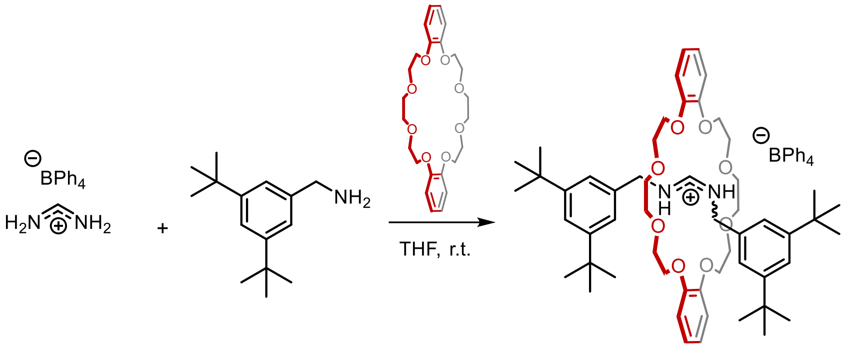

Scheme S14. Amidinium rotaxane synthesis using **DB24C8** as a ring component. Reaction conditions: 1.0 eq. **FA·BPh₄** (0.2 M), 2.0 eq. **DB24C8**, 3.0 eq. amine **4a**; solvent – THF; r.t.

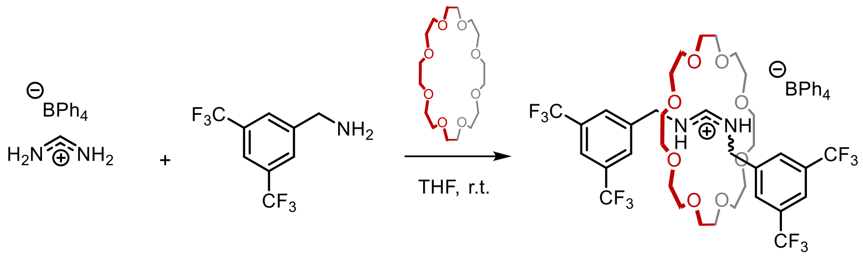

Scheme S15. Amidinium rotaxane synthesis using 3,5-bis(trifluoromethyl)benzylamine as a stopper. Reaction conditions: 1.0 eq. **FA·BPh₄** (0.2 M), 2.0 eq. **24C8**, 3.0 eq. amine; solvent – THF; r.t.

7.4. Comparison between reaction pathways of passive template, active template and "inhibitive" template approaches toward rotaxane self-assembly

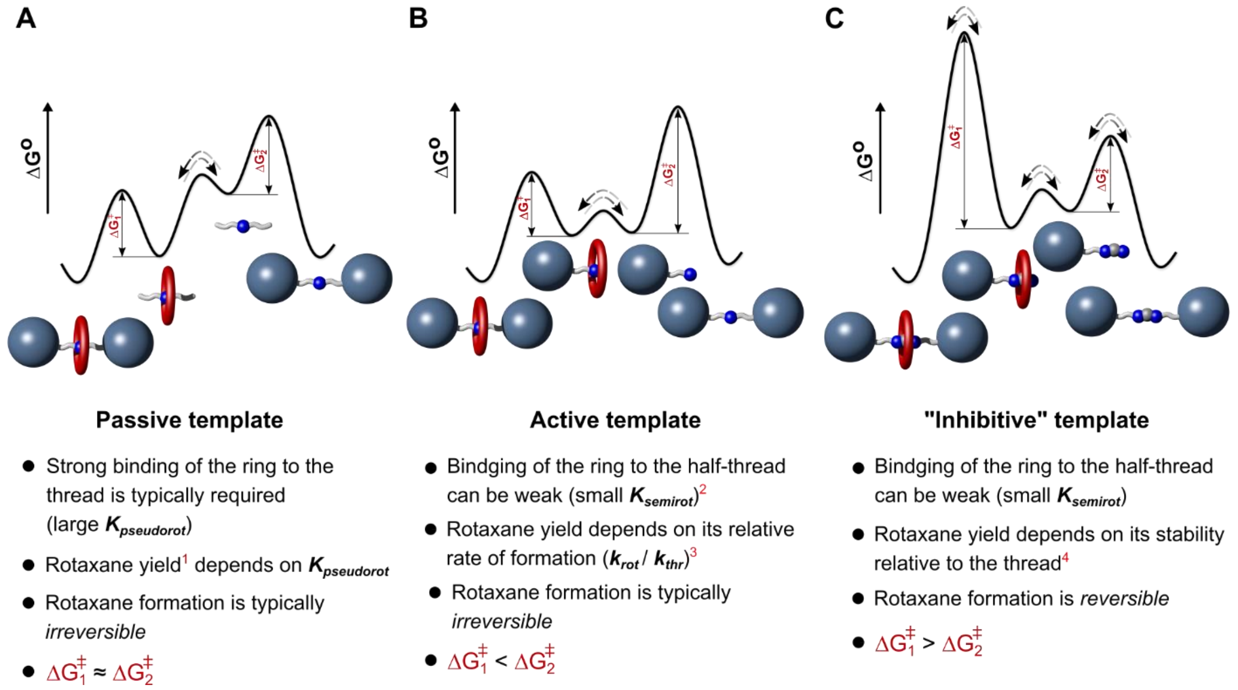

Figure S50. Simplified qualitative free energy diagrams for rotaxane self-assembly by (A) passive template, (B) active template and (C) "inhibitive" template approaches. Typical thermodynamic and kinetic features of each approach are listed below the diagrams.

[1]Assuming that the stoppering reaction has quantitative yield.

[2]This refers more to the metal-free active template.[18b] In the classical active template, the ring can (strongly) bind to all other building blocks simultaneously.[21]

[3]Strictly speaking, binding strength between the ring and the thread components also affects the ratio between the rotaxane and the thread.[21] However, kinetic factor plays here an important role in the reaction outcome, in contrast to the passive template approach.

[4]Both kinetic and thermodynamic stability. In the latter case, the measure of the rotaxane stability can be an association constant for the corresponding pseudorotaxane.

7.5. Mechanism of the rotaxane self-assembly by amidinium exchange

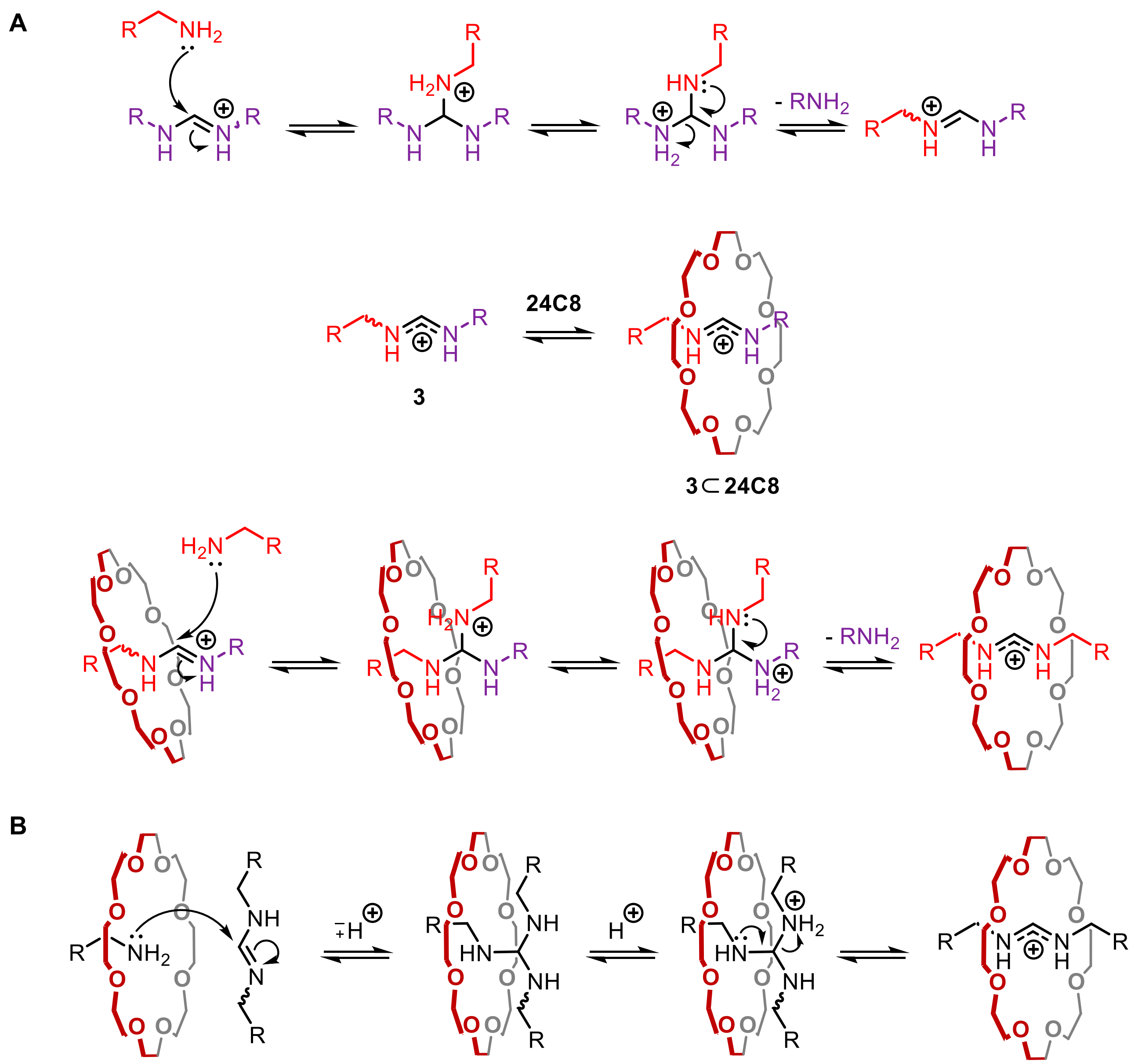

Scheme S16. (A) Mechanism of the rotaxane self-assembly by amidinium exchange. Crown ether can bind to any intermediate of the mechanism; however, only the key complexes featuring **24C8**, which are important for the rotaxane formation, are shown. Compound **3** undergoes similar transformations as **3⊂24C8** yielding the thread. Comprehensive investigation of the mechanism of the carboxylate-assisted amidinium exchange can be found in Ref 22.[22] (B) An alternative mechanism of the rotaxane self-assembly starting from the free thread, **24C8** and the amine stopper. This mechanism is the kind of metal-free active template synthesis discovered by Leigh and co-workers.[18b, 20]

8. *E,E*/*E,Z* isomerization in (mechanically interlocked) amidinium ions

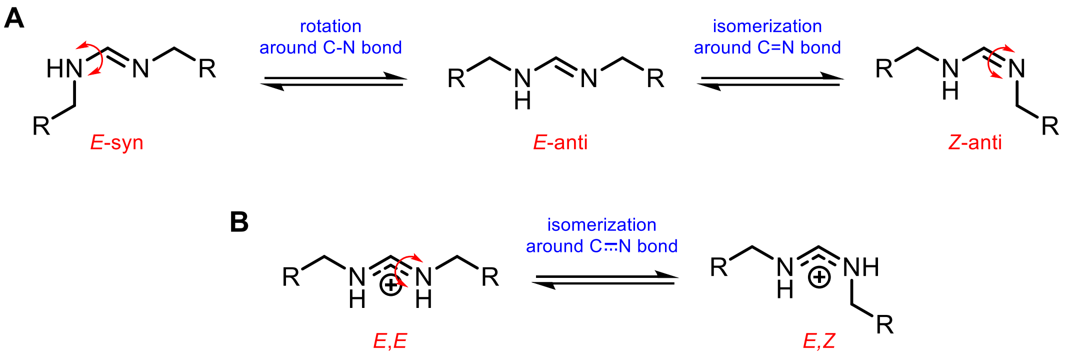

Scheme S17. Configurational and conformational isomerism in (A) amidines and (B) amidinium ions. Only the most stable conformers/isomers are shown. For amidines, interconversion between individual isomers can include tautomerization equilibria.[23] While thermodynamics and kinetics of isomerization in *N*,*N*'-disubstituted amidines has been extensively studied,[23-24] isomerization in formamidinium ions is relatively underexplored.[25]

8.1. Isomerization thermodynamics in S1 and S1⊂24C8

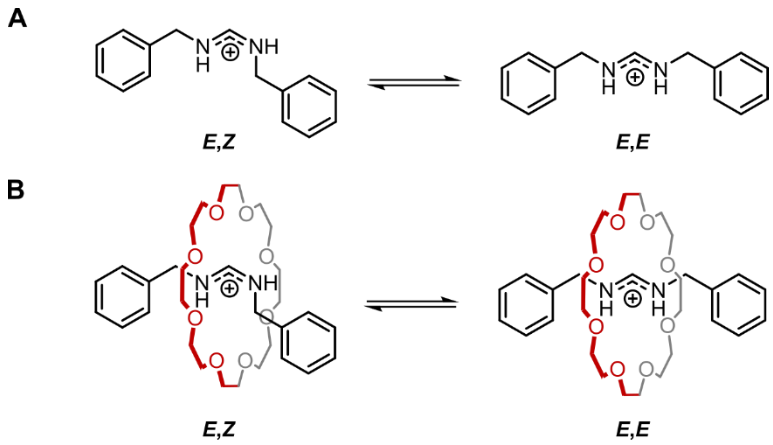

Scheme S18. *E,Z* ⇌ *E,E* equilibrium in (A) **S1** and (B) **S1⊂24C8**.

Table S21. Molar percentage ($\chi_{E,E}$) of *E,E* isomers of **S1** (BPh_4^- salt) and **S1⊂24C8** (BPh_4^- salt) in different solvents. CD_3CN: c(**S1**) ≈ 4 mM, c(**S1⊂24C8**) = 10 mM (1.0 eq. **24C8**). $CD_3CN/CDCl_3$ (5:95 v/v): c(**S1**) ≈ 6 mM, c(**S1⊂24C8**) = 8 mM (1.0 eq. **24C8**). Values of $\chi_{E,E}$ were determined by integration of benzylic signals (**S1**) or crown ether signals (**S1⊂24C8**) in the corresponding ^{1}H NMR spectra (400 MHz, 295 K).

Solvent	$\chi_{E,E}$, %	
	**S1**	**S1⊂24C8**
CD_3CN	15	17
$CD_3CN/CDCl_3$ (5:95 v/v)*	22	18

*Pure $CHCl_3$ could not be used due to poor solubility of **S1** (BPh_4^- salt).

8.2 Isomerization kinetics and thermodynamics in 1a and 2a

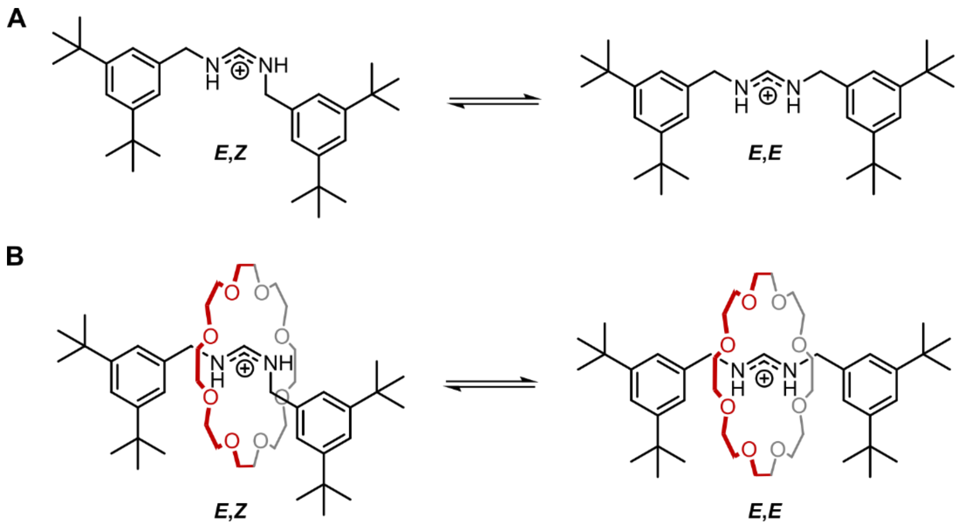

Scheme S19. *E*,*Z* ⇌ *E*,*E* equilibrium in (A) **2a** and (B) **1a**.

Table S22. Molar percentage ($\chi_{E,E}$) of *E*,*E* isomers of **2a** (BPh_4^- salt) and **1a** ($BArF^-$ salt) in different solvents. CD_3CN: c(**2a**) ≈ 10 mM, c(**1a**) = 3 mM. $CDCl_3$: c(**1a**) ≈ 5 mM. CD_2Cl_2: c(**1a**) = 5 mM. $C_2D_2Cl_4$: c(**1a**) = 5 mM. Values of $\chi_{E,E}$ were determined by integration of benzylic signals (**2a**) or crown ether signals (**1a**) in the corresponding ^1H NMR spectra (400 MHz, 295 K; for $C_2D_2Cl_4$ – 500 MHz, 300 K).

Solvent	$\chi_{E,E}$, %	
	**2a**	**1a**
CD_3CN	16	13
$CDCl_3$	N/A	13
CD_2Cl_2	N/A	13
$C_2D_2Cl_4$	N/A	14

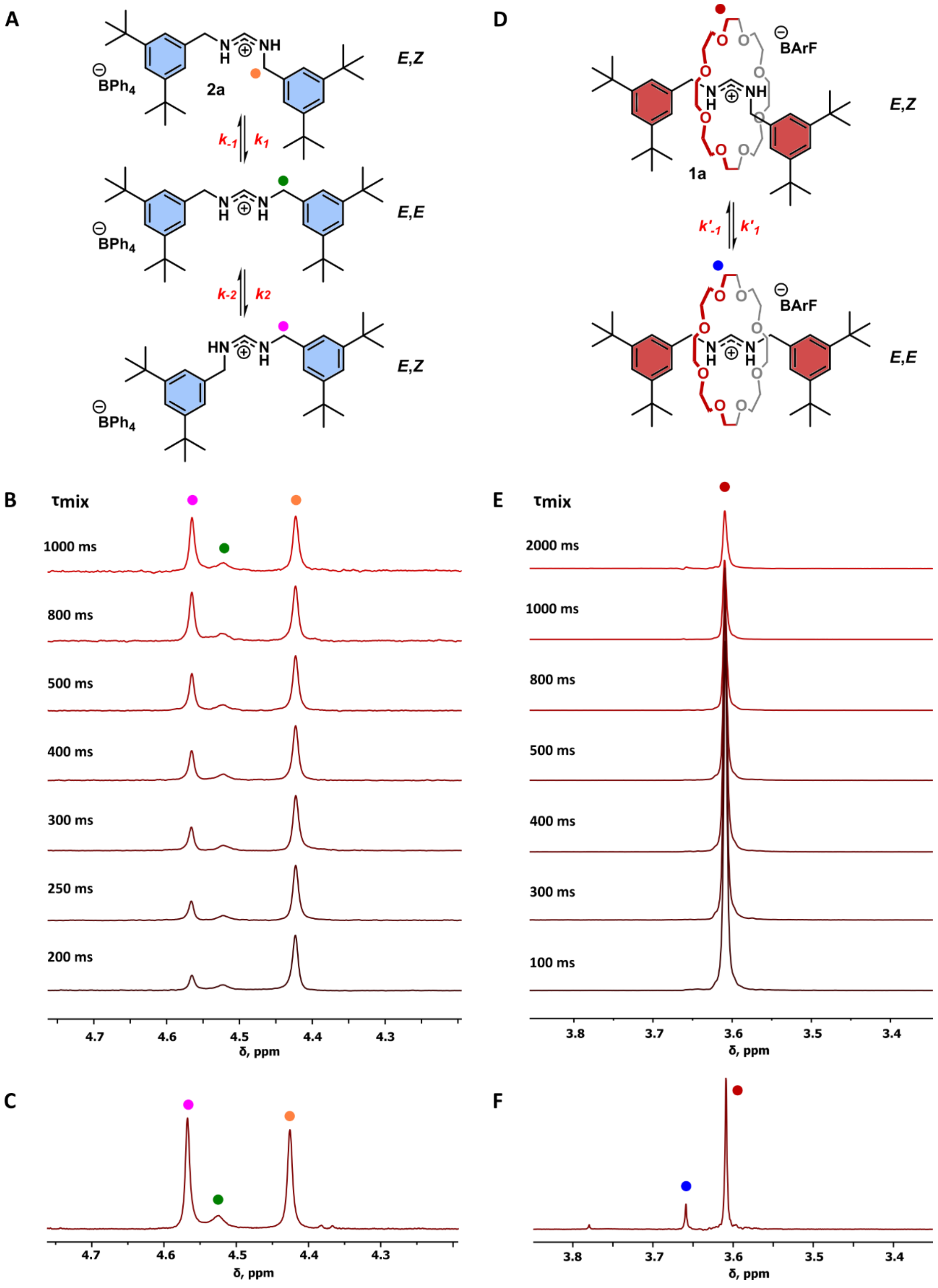

Figure S51. ^{1}H 1D EXSY measurements of **2a** (BPh_4^- salt) and **1a** ($BArF^-$ salt) in CD_3CN *at room temperature* (400 MHz, 295 K). (A) Interconversion between *E*,*Z* and *E*,*E* isomers of **2a**. (B) 1D EXSY spectra of **2a** (c = 3 mM; benzylic region is shown) recorded with different mixing times (τ_{mix}). Benzylic protons of *E*,*Z* isomer (marked orange) giving a singlet at 4.43 ppm were excited. (C) ^{1}H NMR spectrum (400 MHz, CD_3CN, 295 K; benzylic region) of **2a** (BPh_4^- salt) for comparison. (D) Interconversion between *E*,*Z* and *E*,*E* isomers of **1a**. (E) 1D EXSY spectra of **1a** (c = 5 mM; crown ether region is shown) recorded with different mixing times (τ_{mix}). Crown ether protons of *E*,*Z* isomer (marked red) giving a singlet at 3.61 ppm were excited. Benzylic signals were not resolved enough to monitor them in the EXSY experiment. (F) ^{1}H NMR spectrum (400 MHz, CD_3CN, 295 K; benzylic region) of **1a** ($BArF^-$ salt) for comparison.

The observable *E*,*Z*/*E*,*E* isomerization rate constants were obtained by fitting the experimental data from 1D EXSY experiments to the following equation:[26]

$$\frac{I_x}{I_d} = \frac{1 - e^{(-r \times x)}}{1 + e^{(-r \times x)}}\ , \qquad \text{(S3)}$$

where r = $k_{forward}$ + $k_{backward}$, I_d – integral of the excited peak, I_x – integral of the EXSY peak.

Assuming that the rate constants for forward and backward chemical processes (in our case – isomerization) are equal, one can calculate the rate constant as k = r/2.

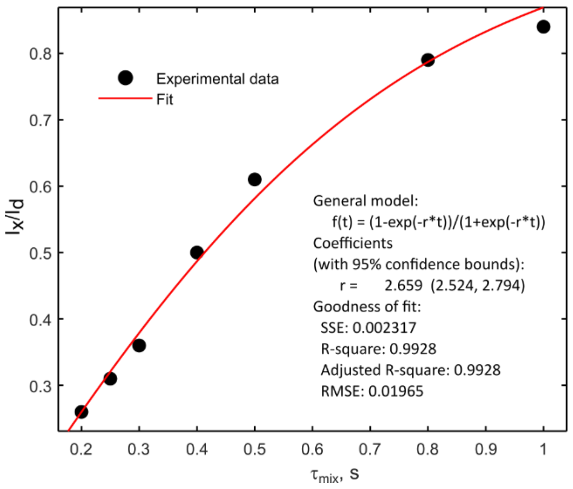

Figure S52. Fit of ^{1}H 1D EXSY experimental data for thread **2a** (BPh_4^- salt) at room temperature to Equation S3 (the corresponding NMR spectra are shown in Figure S51, B). Only integrals of benzylic signals of *E*,*Z* isomer were considered, therefore, the calculated isomerization rate constant for thread **2a** (see below Table S23) characterizes interconversion between two identical *E*,*Z* geometries (top and bottom structures in Figure S51, A) and *not* between *E*,*Z* and *E*,*E* geometries.

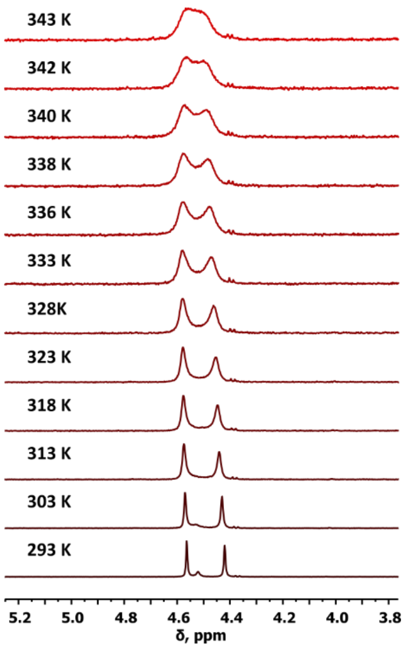

Figure S53. VT NMR stack plot for thread **2a** (BPh_4^- salt; c = 3 mM) in CD_3CN (400 MHz). Benzylic region is shown. Coalescence temperature: T_c = 343 K.

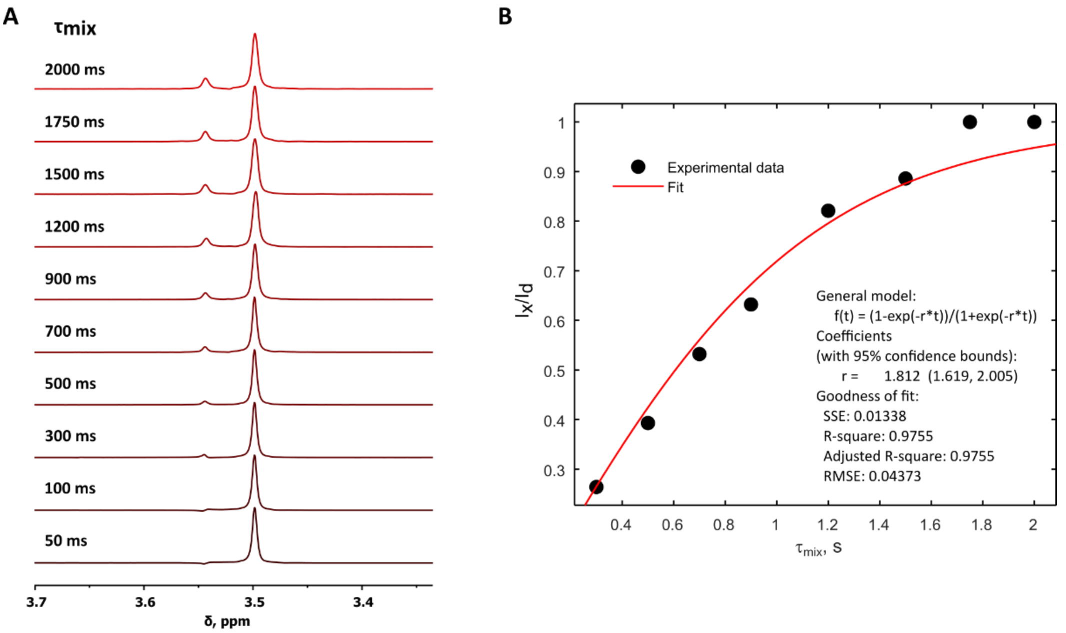

Figure S54. (A) ^{1}H 1D EXSY measurements of rotaxane **1a** (BArF⁻ salt; c = 5 mM; crown ether region is shown) in CD_3CN *at 70 °C* (400 MHz, 343 K). (B) Fit of the EXSY experimental data for **1a** at 70 °C to Equation S3. Since the ratio between exchanging crown ether signals is not 1:1, the integral of each EXSY peak was divided by the integral of EXSY peak at 2000 ms (when the chemical exchange proceeds to completion). The calculated isomerization rate constant for rotaxane **1a** (see below) characterizes interconversion between *E*,*Z* and *E*,*E* geometries (Figure S51, D).

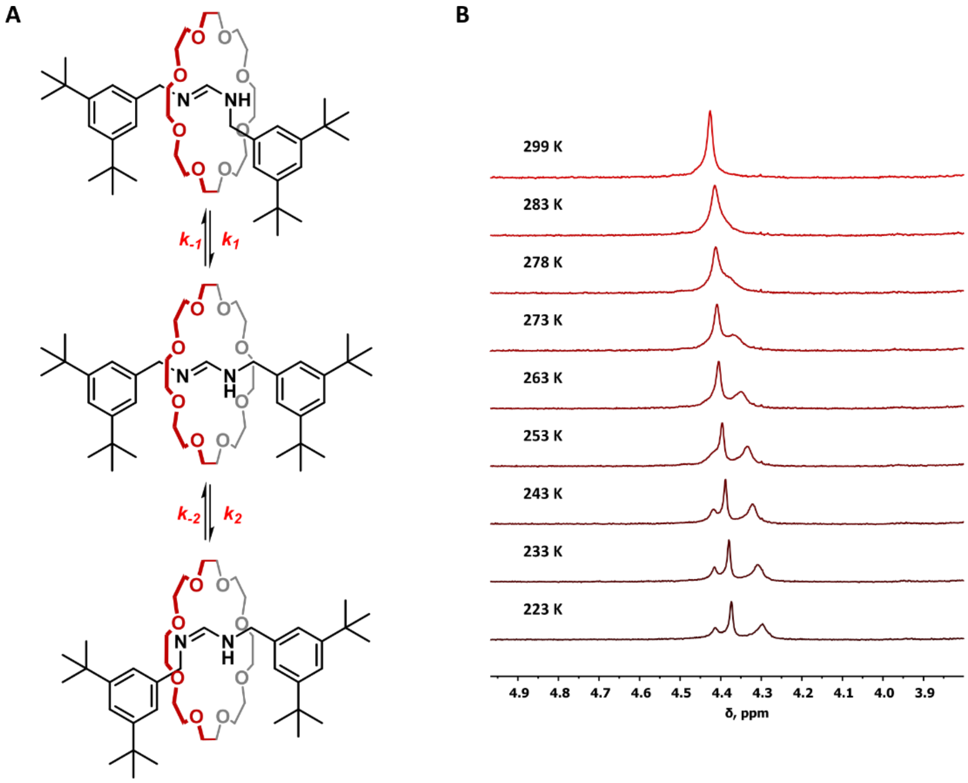

Figure S55. (A) Interconversion between major conformational and configurational isomers of deprotonated rotaxane **1a**. This scheme is rather sketchy, since the true interconversion between different geometrical isomers of amidines might involve multiple tautomeric equilibria and *is not* simple rotation around amidine C-N bonds.[23] (B) VT NMR stack plot for **1a** (BArF⁻ salt; c = 5 mM) in CD_2Cl_2 (400 MHz). Benzylic region is shown. Coalescence temperature: $T_c \approx 278$ K.

Gibbs energies of activation ($\Delta G^{\ddagger}$) for interconversion between individual geometrical isomers of thread **2a** and rotaxane **1a** were calculated using the Eyring equation:

$$k = \frac{\kappa k_B T}{h} e^{-\frac{\Delta G^{\ddagger}}{RT}}, \quad (S4)$$

$$\Delta G^{\ddagger} = -RT \times ln \frac{kh}{\kappa k_B T}, \quad (S5)$$

where k – rate constant of a chemical process, k_B – Boltzmann's constant, h – Planck's constant, R – gas constant, T – temperature, κ – the transmission coefficient (assumed to be equal to one).

Rate constants at specific temperatures were evaluated either by ^{1}H 1D EXSY or using coalescence temperature (T_c) obtained from VT NMR measurements. In the latter case, the rate constant of the interconversion of the isomeric amidinium species at T_c was calculated according to the following equation:

$$k = \frac{\pi}{\sqrt{2}} \times \Delta\nu \ , \quad (S6)$$

where Δv is the difference between two signals of interest, when the exchange rate is very slow on the NMR timescale (we used the NMR spectra at the lowest measured temperature; see Figure S53 and S55)

Table S23. Rate constants and Gibbs energies of activation ($\Delta G^{\ddagger}$) for interconversion between geometrical isomers of **1a** and **2a**.

Amidinium species	Method	Temperature, K	ΔG‡, kJ/mol	k, s^{-1}
Thread **2a**[a]	1D EXSY	**295**	72	**1.3**
	VT NMR	343	71	127[b]
		**295**		**1.6**[c]
Rotaxane **1a**[d] (protonated)	1D EXSY	343	85	0.9
		**295**		**0.006**[c]
Rotaxane **1a**[e] (deprotonated)	VT NMR	278	58	70[f]
		**295**		**300**[c]

[a]Obtained isomerization rate constants characterize interconversion between two identical *E*,*Z* geometries (top and bottom structures in Figure S51, A) and *not* between *E*,*Z* and *E*,*E* geometries.
[b]Calculated according to Equation S6. At 293 K, Δv = 57.2 Hz. This rate constant was used to calculate $\Delta G^{\ddagger}$.
[c]Calculated using the Eyring equation.
[d]Obtained isomerization rate constants characterize interconversion between *E*,*Z* and *E*,*E* geometries (Figure S51, D).
[e]Obtained isomerization rate constants characterize interconversion between undefined geometrical (co-conformational) isomers and tautomers of deprotonated **1a**.
[f]Calculated according to Equation S6. At 223 K, Δv = 31 Hz (for the middle and right signals). This rate constant was used to calculate $\Delta G^{\ddagger}$.

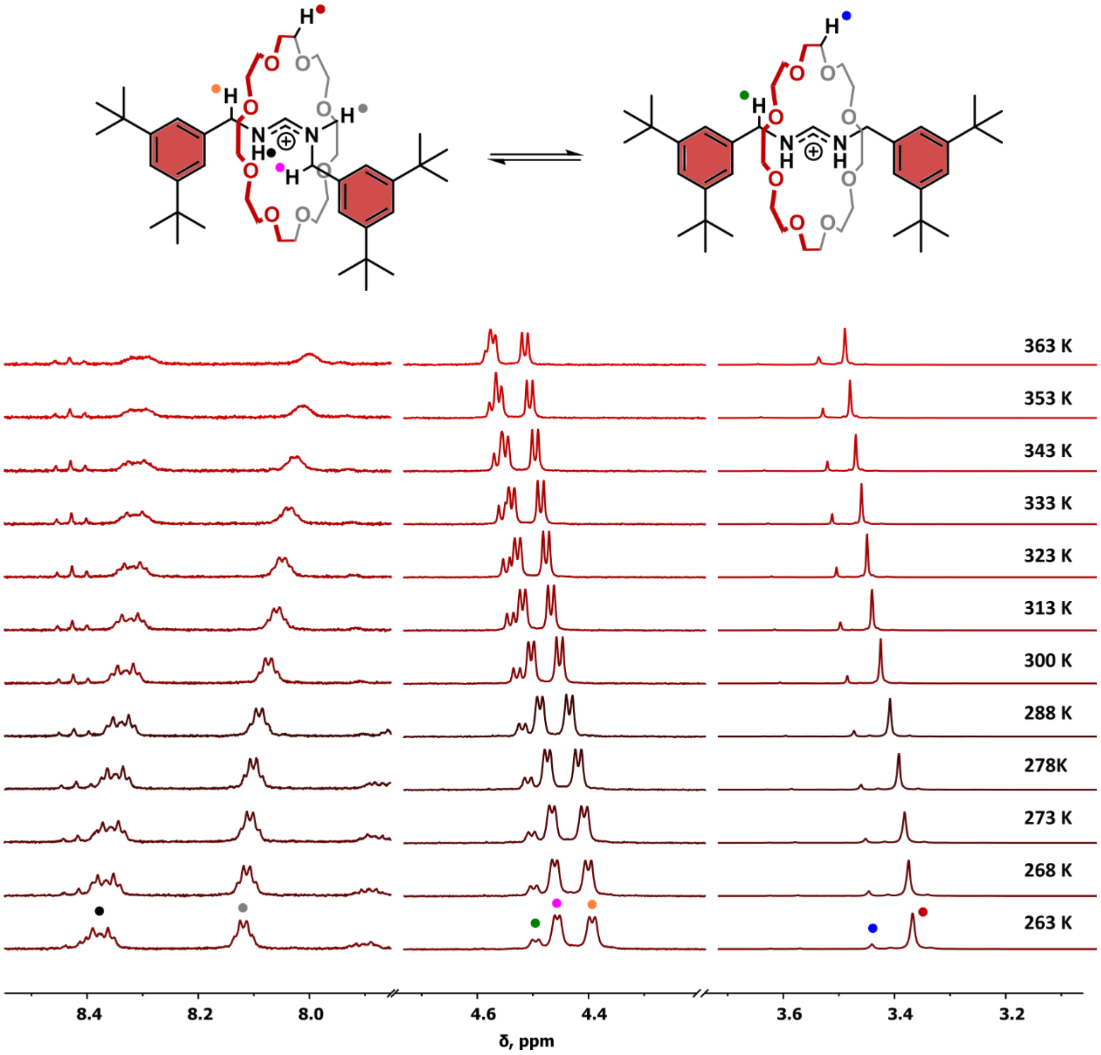

Figure S56. Partial VT NMR stack plot for **1a** (BArF⁻ salt, c = 5 mM) in $C_2D_2Cl_4$ (500 MHz).

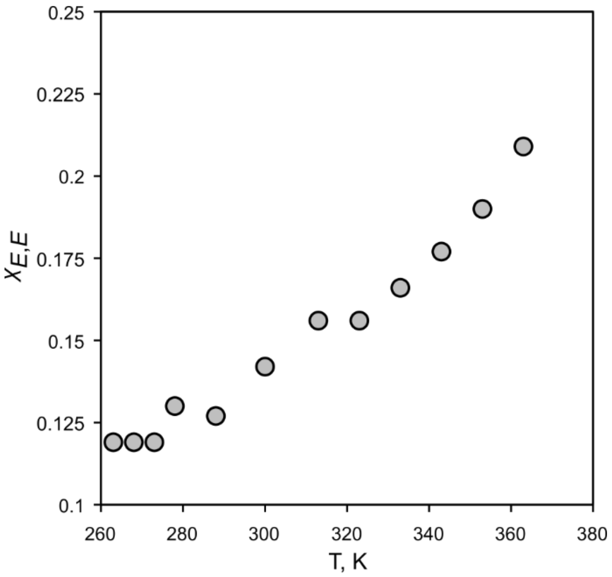

Figure S57. Temperature dependence of molar fraction **χ** of **1a** *E*,*E* isomer (BArF⁻ salt; c = 7 mM) in $C_2D_2Cl_4$. The data is obtained from the experiment shown in Figure S56 through integration of the crown ether CH_2 signals of *E*,*E*- and *E*,*Z*-isomers of **1a**.

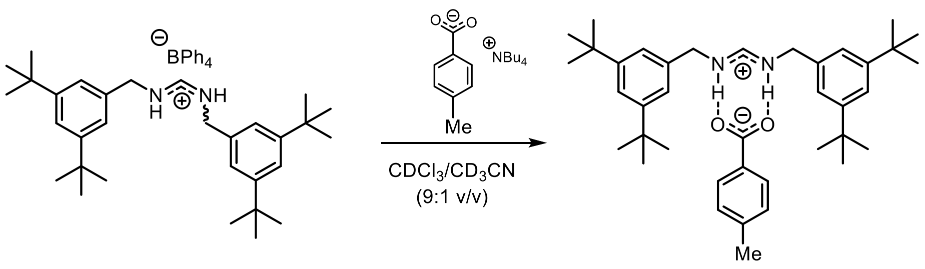

Scheme S20. Carboxylate induced geometry switching in **2a**.

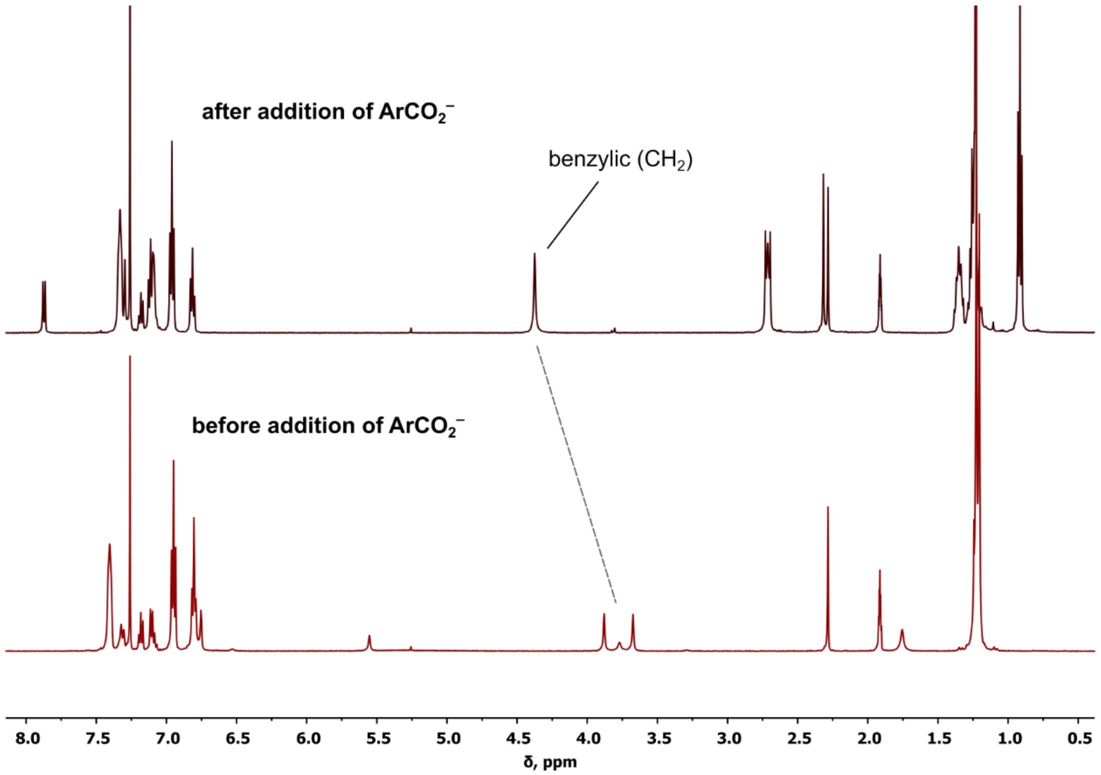

Figure S58. NMR stack plot of carboxylate induced geometry switching in **2a** using tetrabutylammonium 4-methylbenzoate (500 MHz, $CDCl_3/CD_3CN$ 9:1 v/v, 300 K). c(**2a**) = 4 mM, c(benzoate) = 4 mM. Tetrabutylammonium 4-methylbenzoate was prepared from the corresponding acid and tetrabutylammonium hydroxide (1.0 M solution in MeOH) and thoroughly dried at elevated temperature under high vacuum before use.

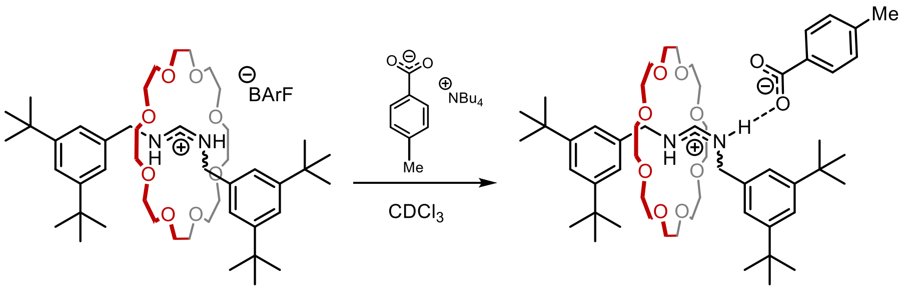

Scheme S21. Formation of a hydrogen-bonded complex between **1a** and aromatic carboxylate.

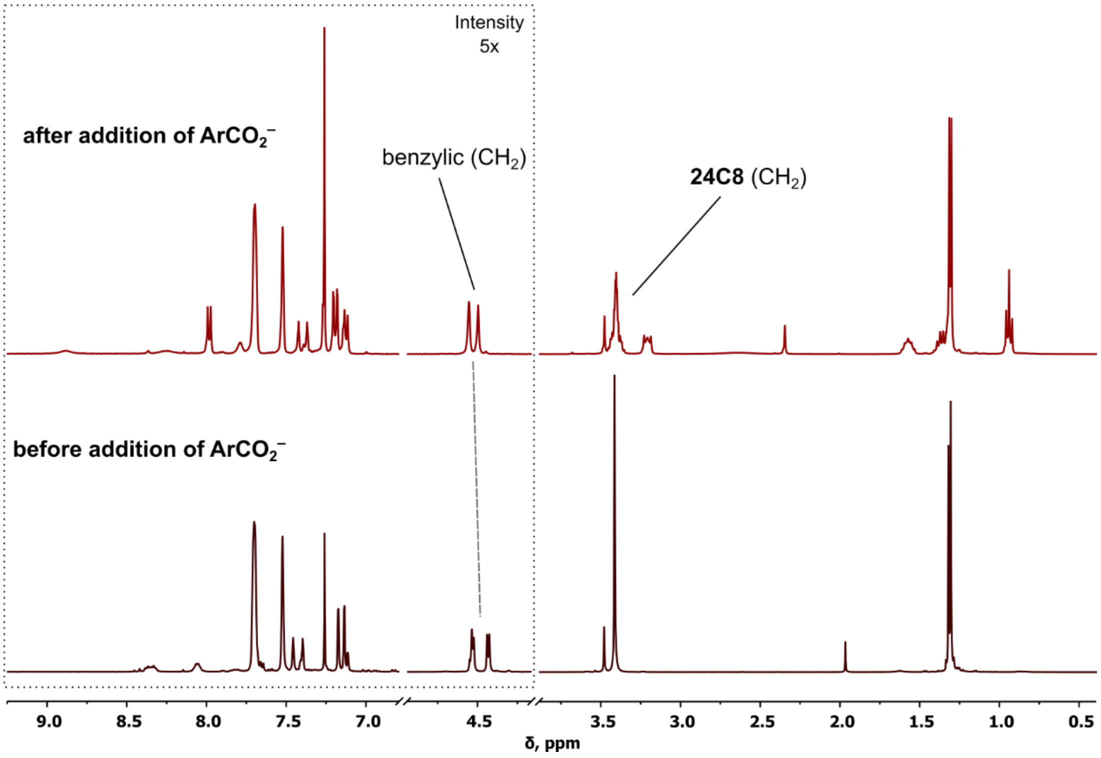

Figure S59. Stack plot of ^{1}H NMR spectra (400 MHz, $CDCl_3$, 296 K) of **1a** (*bottom*) and **1a** (BArF⁻ salt; c = 4 mM) mixed with tetrabutylammonium 4-methylbenzoate (c = 4 mM) (*top*). Tetrabutylammonium 4-methylbenzoate was prepared from the corresponding acid and tetrabutylammonium hydroxide (1.0 M solution in MeOH) and thoroughly dried at elevated temperature under high vacuum before use.

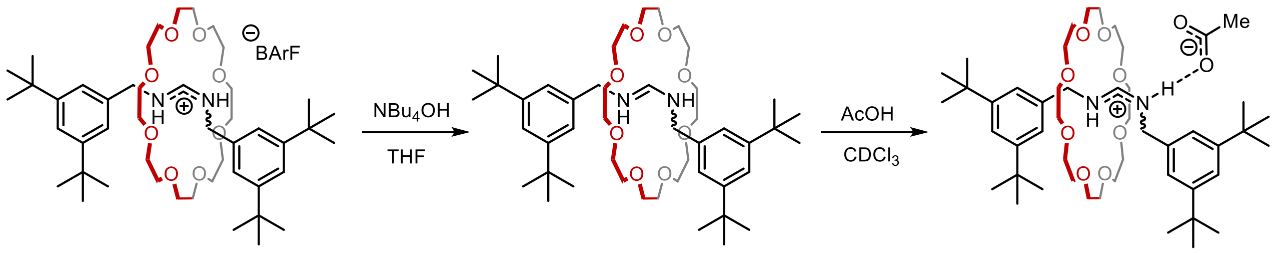

Scheme S22. Deprotonation of **1a** by NBu_4OH followed by formation of a hydrogen-bonded complex between **1a** and acetate.

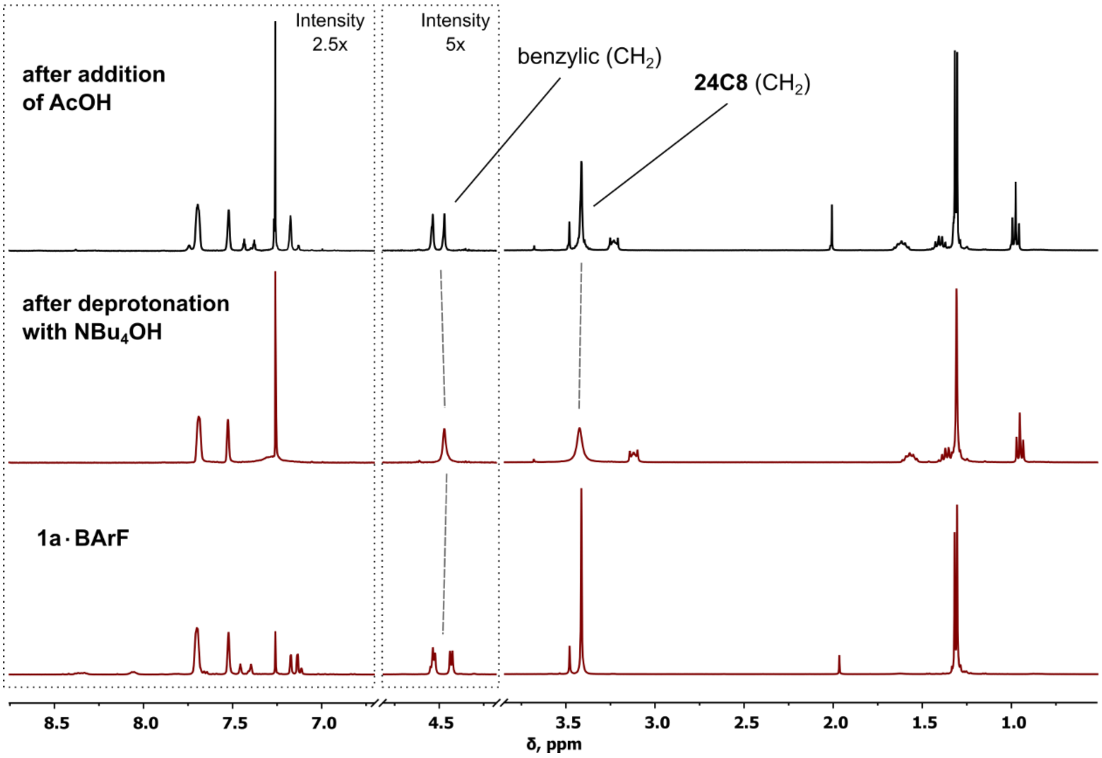

Figure S60. Stack plot of ^{1}H NMR spectra (400 MHz, $CDCl_3$, 295 K) of **1a** ($BArF^-$ salt), deprotonated form of **1a** (c = 4 mM) and a hydrogen-bonded complex between **1a** and acetate. Rotaxane **1a** was deprotonated by addition of NBu_4OH (1.0 M solution in MeOH, 3.2 µL, 3.2 µmol, 1.0 eq.) to a solution of **1a** ($BArF^-$ salt; 5.4 mg, 3.2 µmol) in anhydrous THF (0.5 mL). The resulting mixture was stirred under argon for 10 min and all volatiles were removed under reduced pressure. The residue was then dried under high vacuum and redissolved in $CDCl_3$ (0.7 mL) for NMR measurements. In order to reprotonate the amidine rotaxane, a solution of acetic acid in $CDCl_3$ (0.175 M, 18.5 µL, 1.0 eq.) was added.

8.3 Chemically fueled control over isomerization rate in 1b

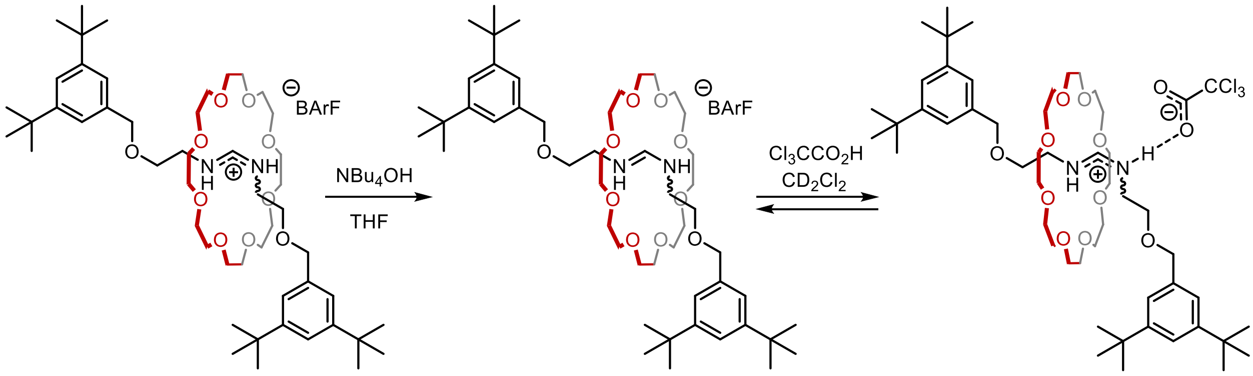

Scheme S23 Deprotonation of **1b** by NBu_4OH followed by formation of a hydrogen-bonded complex between **1b** and trichloroacetate.

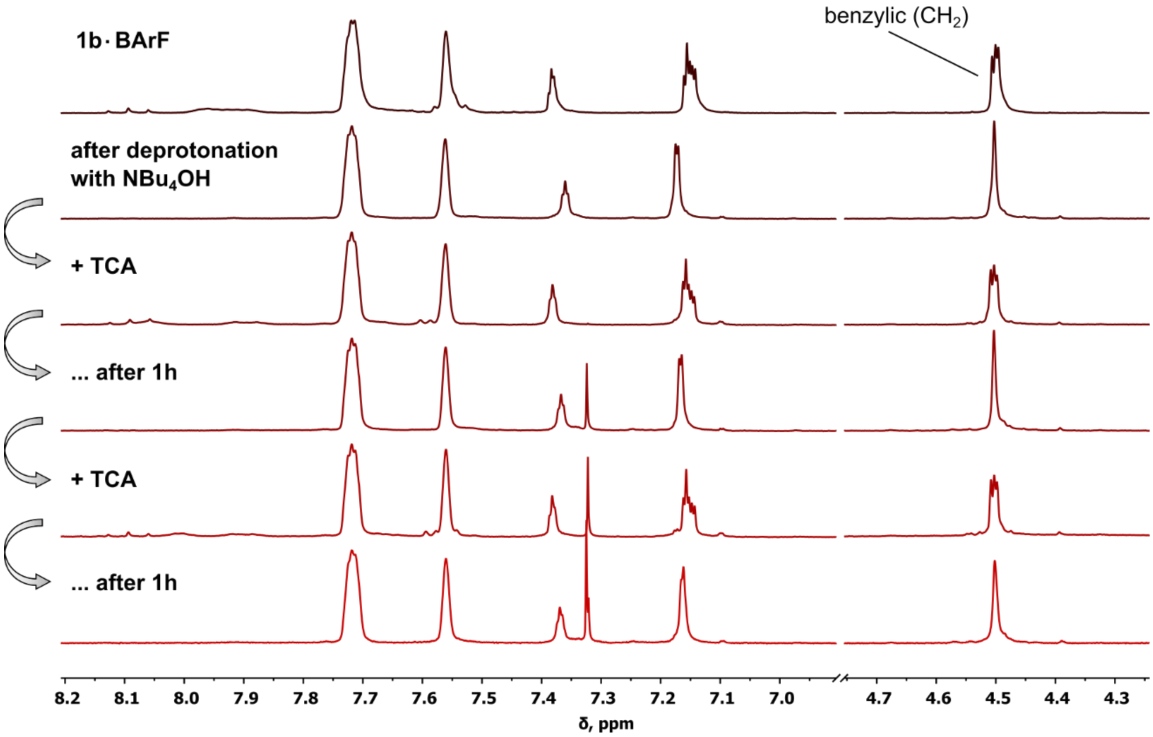

Figure S61. Stack plot of partial ^{1}H NMR spectra (400 MHz, CD_2Cl_2, 295 K) of **1b** ($BArF^-$ salt), deprotonated form of **1b** (c = 6 mM) and a hydrogen-bonded complex between **1b** and trichloroacetate. Rotaxane **1b** was deprotonated by addition of NBu_4OH (1.0 M solution in MeOH, 4.4 µL, 4.4 µmol, 1.0 eq.) to a solution of **1b** ($BArF^-$ salt; 7.8 mg, 4.4 µmol) in anhydrous THF (0.5 mL). The resulting mixture was stirred under argon for 10 min and all volatiles were removed under reduced pressure. The residue was then dried under high vacuum and re-dissolved in CD_2Cl_2 (0.7 mL) for NMR measurements. In order to re-protonate the amidine rotaxane, a solution of trichloroacetic acid (TCA) in CD_2Cl_2 (0.218 M, 20.2 µL, 1.0 eq.) was added. The resulting solution in an NMR tube was then stirred at 40 °C for 1 h (the top of the NMR tube was covered with aluminium foil to prevent significant evaporation of CD_2Cl_2, but to allow escape of CO_2, which was generated upon gradual decarboxylation of TCA). Addition of another portion of TCA (0.218 M in CD_2Cl_2, 20.2 µL, 1.0 eq.) and subsequent stirring at 40 °C were repeated once more. Another region of the NMR stack plot (with crown ether and *tert*-butyl signals) is shown in Figure S62.

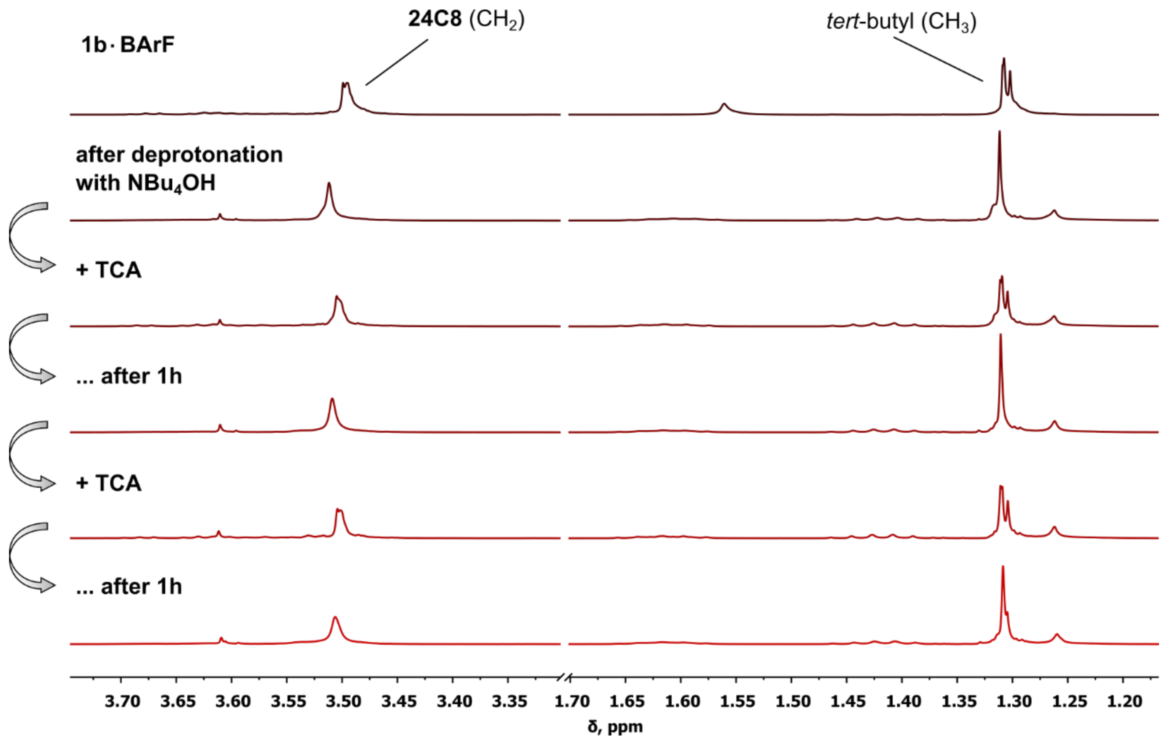

Figure S62. Stack plot of partial ^{1}H NMR spectra (400 MHz, CD_2Cl_2, 295 K) of **1b** (BArF$^-$ salt), deprotonated form of **1b** (c = 6 mM) and a hydrogen-bonded complex between **1b** and trichloroacetate. Please, refer to Figure S61 for additional comments.

9. Dynamic covalent properties of the amidinium [2]rotaxanes

9.1. Impact of the mechanical bond on the amidinium exchange

General procedure for the amidinium exchange between *N*,*N'*-disubstituted (mechanically interlocked) formamidinium ions and aliphatic primary amines

To a solution of *N*,*N'*-disubstituted formamidinium salt (1.0 eq.) in THF a stock solution of a primary aliphatic amine (3.0 eq.) in THF was added. The reaction mixture was then stirred in a closed screw-cap HPLC vial at r.t. for several days and monitored by LCMS. For details, see captions of the figures below.

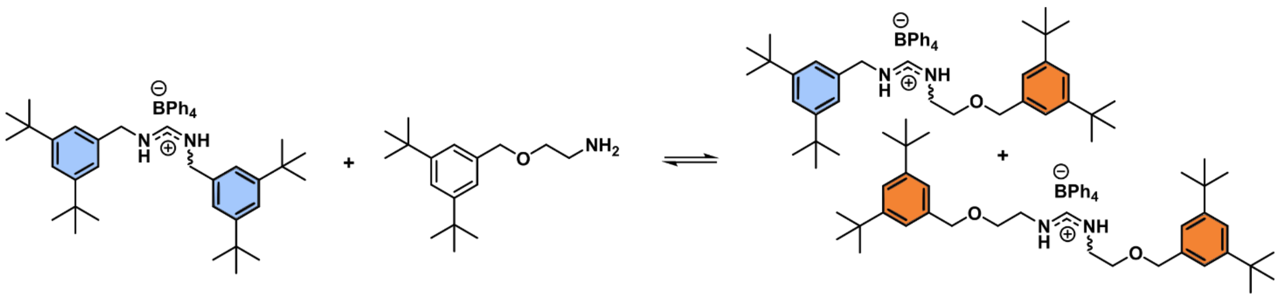

Scheme S24 Amidinium exchange between thread **2a** and amine **4b**.

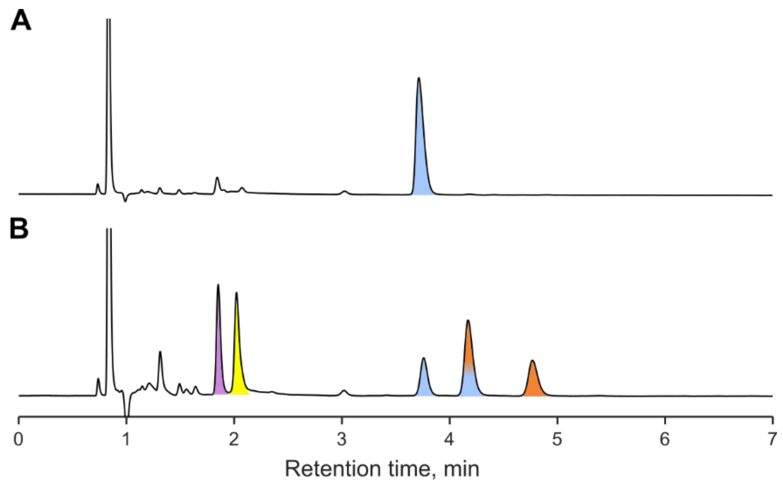

Figure S63. (A) HPLC chromatogram of thread **2a** (BPh_4^- salt). (B) HPLC chromatogram of the reaction between thread **2a** (35 µmol, *vide infra*) and amine **4b** (28 mg, 105 µmol, 3.0 eq.) in THF (total volume – 370 µL) after 1 day at r.t. Thread **2a** was obtained from **FA·BPh₄** (13 mg, 35 µmol, 1.0 eq.) and amine **4a** (23 mg, 105 µmol, 3.0 eq.) stirred in MeCN under reflux for 30 min. All volatiles were then removed under reduced pressure and the crude product was redissolved in THF (170 µL). Color code of the chromatographic peaks: purple – **4a**; yellow – **4b**; blue – **2a**; orange – **2b**; orange-blue – unsymmetrical thread formed from **4a** and **4b** (see Scheme S24).

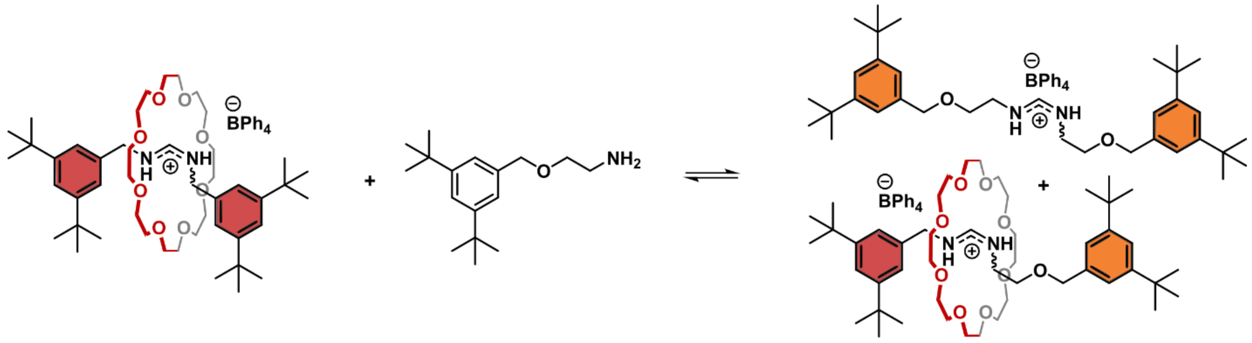

Scheme S25 Amidinium exchange between rotaxane **1a** and amine **4b**.

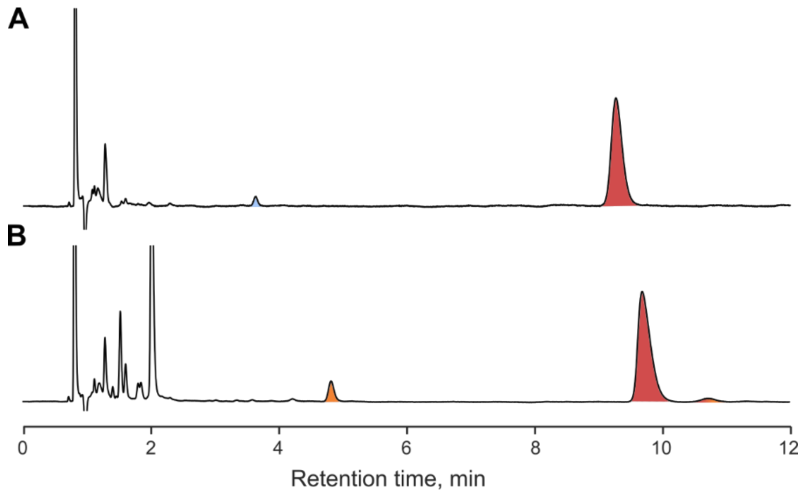

Figure S64. (A) HPLC chromatogram of rotaxane **1a** (BArF⁻ salt). The sample contains **2a** (blue peak) as an impurity. (B) HPLC chromatogram of the reaction between rotaxane **1a** (3.0 mg, 2.7 µmol, 1.0 eq.) and amine **4b** (2.1 mg, 8.0 µmol, 3.0 eq.) in THF (total volume – 50 µL) after 5 days at r.t. Orange peak (retention time 4.9 min) is **2b**. Peak at 10.8 min corresponds to the unsymmetrical rotaxane formed from **4a** and **4b** (see Scheme S25).

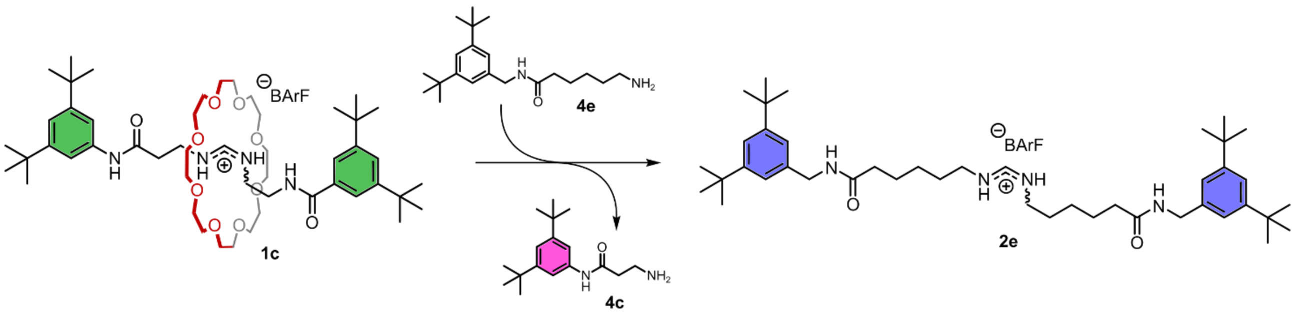

Scheme S26 Amidinium exchange between rotaxane **1a** and amine **4b**.

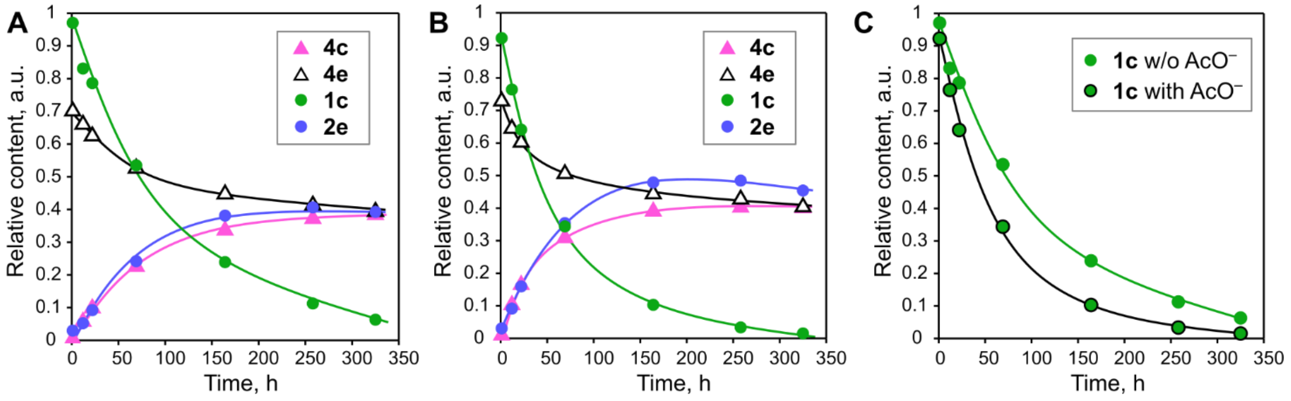

Figure S65. LCMS monitoring of the reaction between rotaxane **1c** and amine **4e** in the presence and absence of acetate. (A) Change of the relative content of **1c**, **2e**, **4c** and **4e** in the reaction mixture *not containing acetate* over time. The lines are shown to guide the eye. Formation of small amount of the unsymmetrical rotaxane **1c/e** (composed of amine stoppers **4c** and **4e**) was also observed (not plotted). (B) Change of the relative content of **1c**, **2e**, **4c** and **4e** in the reaction mixture *containing acetate* over time. The lines are shown to guide the eye. Formation of small amount of the unsymmetrical rotaxane **1c/e** (composed of amine stoppers **4c** and **4e**) was also observed (not plotted). (C) Change of the relative content of **1c** in the reaction mixture (with and without acetate) over time. This plot contains data from two previous plots (panels A and B). The lines are shown to guide the eye. *Experimental procedure:* Rotaxane **1c** (BArF⁻ salt; 5.4 mg, 3.0 µmol, 1.0 eq.) was dissolved in THF (26 µL) in an HPLC vial insert. A stock solution of amine **4e** (88.1 mg/mL in THF, 34.4 µL, 9.12 µmol, 3.0 eq.) was added and the reaction mixture was stirred in a closed screw-cap vial at r.t. for 14 days. The same experiment was repeated under identical conditions, but in the presence of tetrabutylammonium acetate (52.4 mg/mL in THF, 1.0 eq.). Relative content of **1c**, **2e**, **4c** and **4e** was calculated as a ratio between chromatographic peak areas of the species of interest and BArF⁻ as an internal standard. *Comment:* Acetate clearly increases the rate of the amidinium exchange in rotaxane **1c** (see panel C). We attribute this to partial displacement of the crown ether ring from the amidinium moiety by the carboxylate upon acetate binding to the amidinium NH groups. This would result in lesser sterical hindrance of the amidinium moiety caused by **24C8** and would give primary amines better access to the amidinium electrophilic carbon.

9.2. Dynamic combinatorial libraries (DCL) of the amidinium [2]rotaxanes

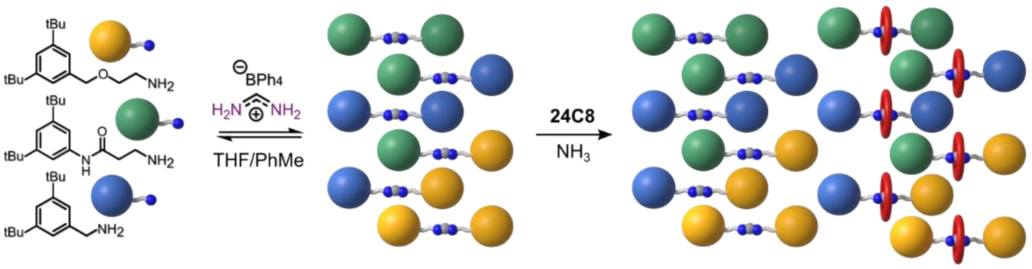

Scheme S27 Cartoon representation of the reaction affording the DCL of the amidinium [2]rotaxanes.

Typical procedure for generation of the DCL of the amidinium threads and [2]rotaxanes

A 1.5 mL screw-cap HPLC vial was charged with amines **4a** (5.4 mg, 25 µmol, 0.67 eq.), **4b** (6.5 mg, 25 µmol, 0.67 eq.), **4c** (6.8 mg, 25 µmol, 0.67 eq.) and **FA·BPh₄** (13.5 mg, 37 µmol, 1.0 eq.). The mixture was dissolved in THF (0.5 mL) and stirred at 60 °C for 2 h (a needle was inserted into the vial cap to keep the reaction mixture open-air and allow NH_3 to escape the reaction vessel). The solvent and all volatiles were then removed under reduced pressure, affording crude DCL of the amidinium threads. An internal HPLC standard – 1,2,4,5-tetramethylbenzene – was added to the residue, followed by PhMe (70 µL), **24C8** (26.0 mg, 22.8 µL, 74 µmol, 2.0 eq.) and NH_3 (0.5 M solution in THF, 110 µL, 55 µmol, 1.5 eq.). The reaction mixture was stirred in a tightly closed HPLC vial at 65 °C for 100 h. The reaction was monitored by LCMS (V_{al} = 1 µL; each aliquot was diluted in 1 mL LCMS grade acetonitrile).

Isolation of the rotaxane DCL. The reaction mixture was diluted with THF (100 µL) and 50% aq. NH_2OH (2.27 µL, 37 µmol, 1.0 eq.) was added. After stirring the mixture at r.t. for 10 min, all volatiles were removed under reduced pressure and the crude product was purified by semi-preparative HPLC (see Section 1 for details) affording the rotaxane DCL (HCO_2^- form, 6.0 mg, 6.6 µmol, 18%). *Note*: after isolation by semi-preparative HPLC, a CH_2Cl_2 solution (3 mL) of the obtained product was washed with H_2O to remove excess of ammonium formate (which came from the HPLC mobile phase).

Isolation of the thread DCL. The crude DCL of the amidinium threads (prepared from 27.0 mg **FA·BPh₄**, 10.8 mg **4a**, 13.0 mg **4b**, 13.6 mg **4c**) was purified by semi-preparative HPLC (Mobile phase A: 0.023 M HCO_2NH_4 and 0.0019 M HCO_2H in H_2O. Mobile phase B: MeCN. Isocratic elution was applied at Mobile phase A : Mobile phase B = 75:25. Flow rate: 2.0 mL/min), affording pure thread DCL (HCO_2^- form, 31 mg, 55 µmol, 75%). *Note*: after isolation by semi-preparative HPLC, a CH_2Cl_2 solution (3 mL) of the obtained product was washed with H_2O to remove excess of ammonium formate.

Quantitative analysis of the DCL of the amidinium [2]rotaxanes

Amounts of all *symmetrical* DCL members (**2a**, **2b**, **2c**, **1a**, **1b**, **1c**) were calculated from HPLC chromatograms using 1,2,4,5-tetramethylbenzene as an internal standard and the corresponding calibration curves (Figure S38 and S66). The amount of each *unsymmetrical* DCL member (**2a/b**, **2a/c**, **2b/c**, **1a/b**, **1a/c**, **1b/c**) was calculated as the mean of amounts of the *two* related *symmetrical* threads using a chromatographic peak area of an unsymmetrical DCL member and calibration curves for the related symmetrical threads (e.g., for thread **2a/b**, we used its HPLC peak area to calculate amount of thread **2a** and amount of thread **2b**; then we calculated the mean of the obtained two values). Mole percentages of all DCL members were calculated as the amount of the DCL member divided by either the amount of **FA·BPh₄** used to generate DCL of threads or the sum of initial amounts of all thread/rotaxane DCL members.

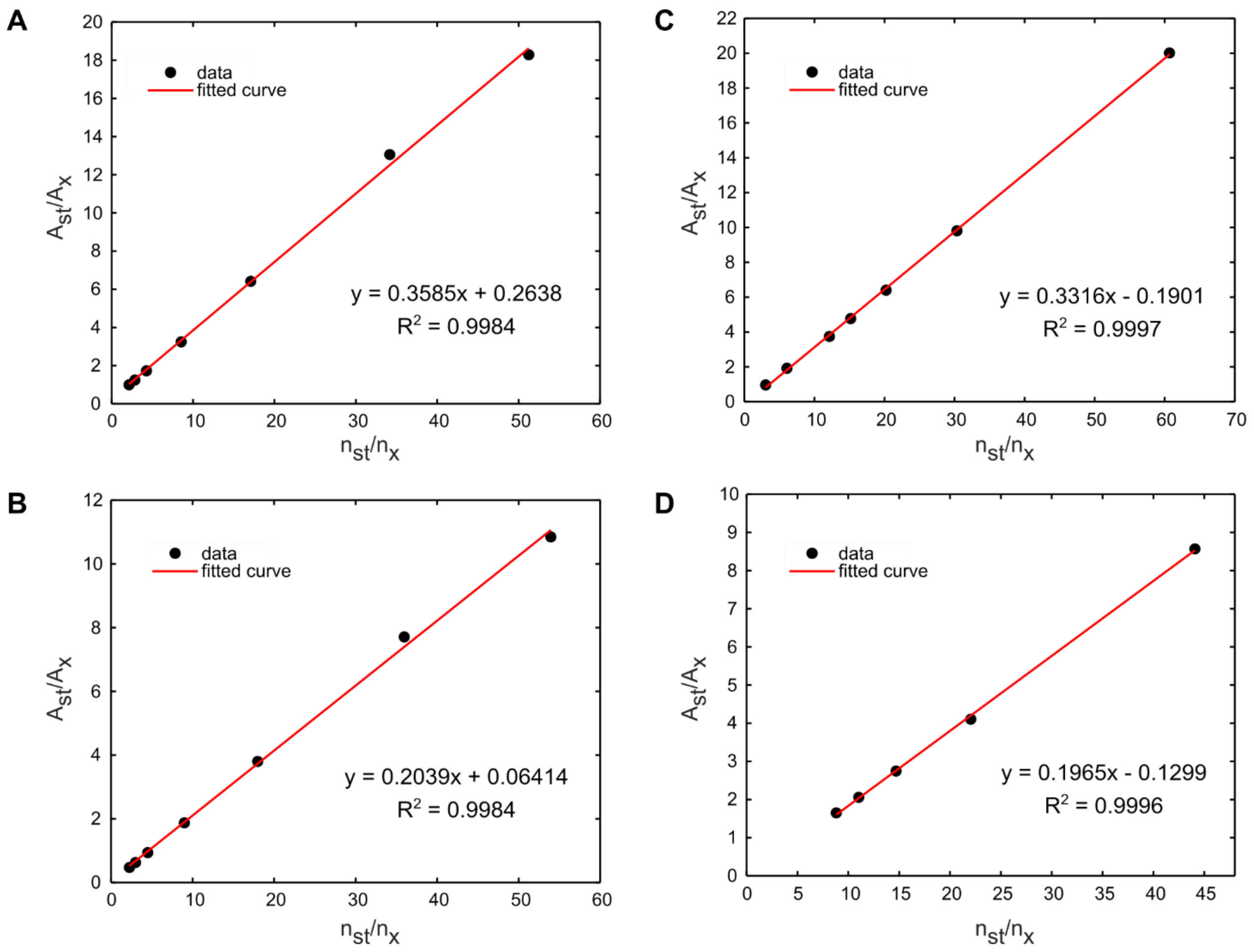

Figure S66. HPLC calibration curves for (A) thread **2b**, (B) thread **2c**, (C) rotaxane **1b**, (D) rotaxane **1c**. Internal standard – 1,2,4,5-tetramethylbenzene. n_{st}/n_x – molar ratio between the standard and the calibrated compound; A_{st}/A_x – ratio of chromatographic peak areas of the standard and the calibrated compound. For calibration curves for thread **2a** and rotaxane **1a**, see Figure S38.

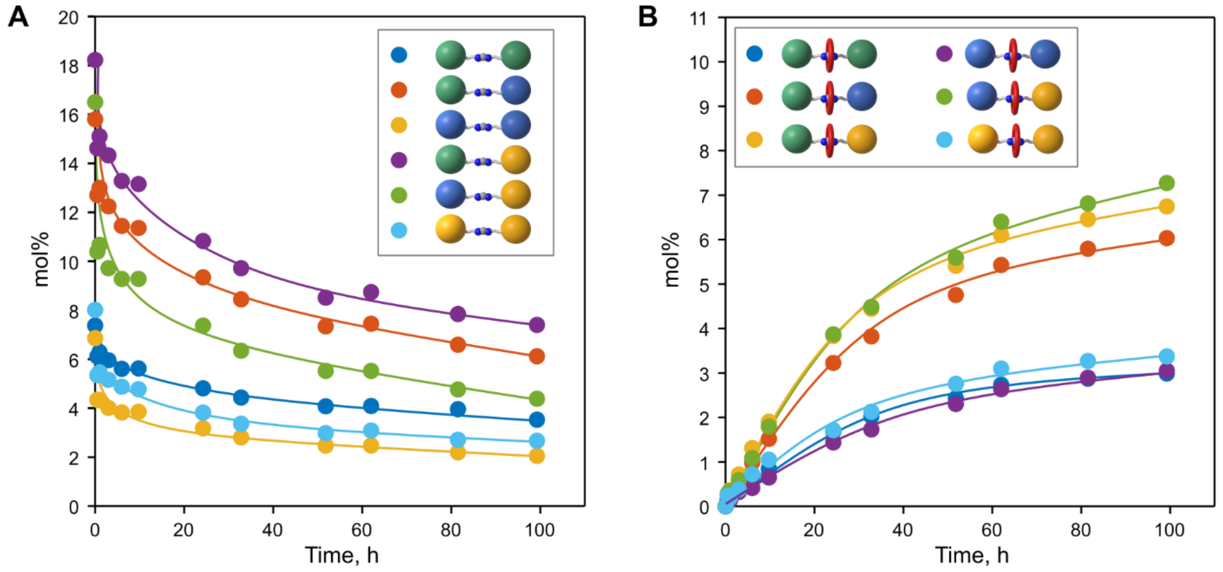

Figure S67. LCMS monitoring of the evolution of the dynamic covalent sublibraries of (A) threads (**2a**, **2b**, **2c**, **2a/b**, **2a/c**, **2b/c**) and (B) corresponding rotaxanes (**1a**, **1b**, **1c**, **1a/b**, **1a/c**, **1b/c**) upon heating DCL of threads with NH_3 and **24C8** in THF (see the typical synthesis procedure above). Mole percentages of all DCL members were calculated as the amount of a DCL member divided by the amount of **FA·BPh$_4$** used to generate DCL of threads. The lines are shown to guide the eye.

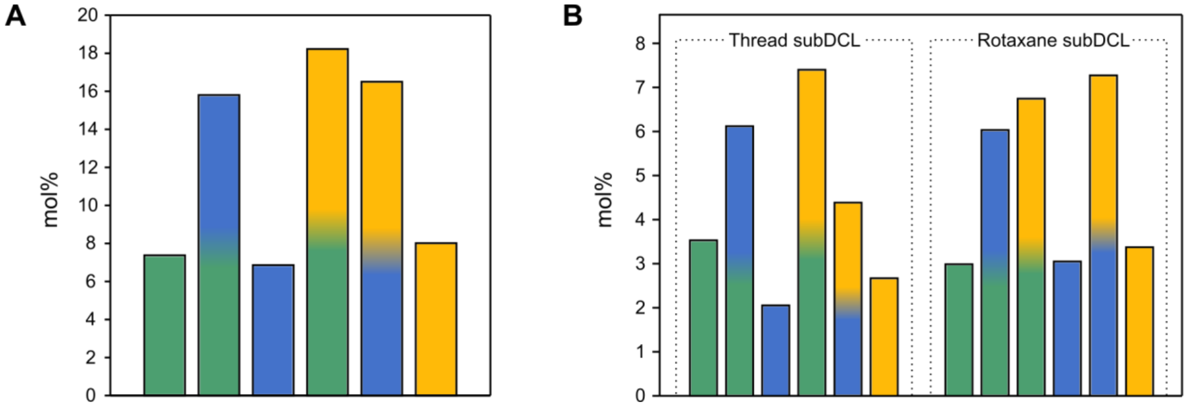

Figure S68. Bar graphs showing the composition of the amidinium DCL (A) before addition of NH_3 and **24C8** and (B) after heating the DCL with NH_3 and **24C8** for 100 h. The data shown in the bar graphs corresponds to the experiment described in Figure S67.

General procedure for degradation/reconfiguration of the amidinium DCLs with primary amines

A 1.5 mL screw-cap HPLC vial (or HPLC vial insert) was charged with the amidinium DCL (HCO_2^- form, 1.0 eq.) and 1,2,4,5-tetramethylbenzene (0.7 – 0.8 M solution in THF) as an internal standard. A primary amine (for DCL degradation: 5.0 eq., 1 M solution in THF; for DCL reconfiguration: 1.0 eq., 0.5 M solution in THF) was then added and the reaction mixture was stirred at room temperature (or 45 °C) for a few hours/days and monitored by LCMS.

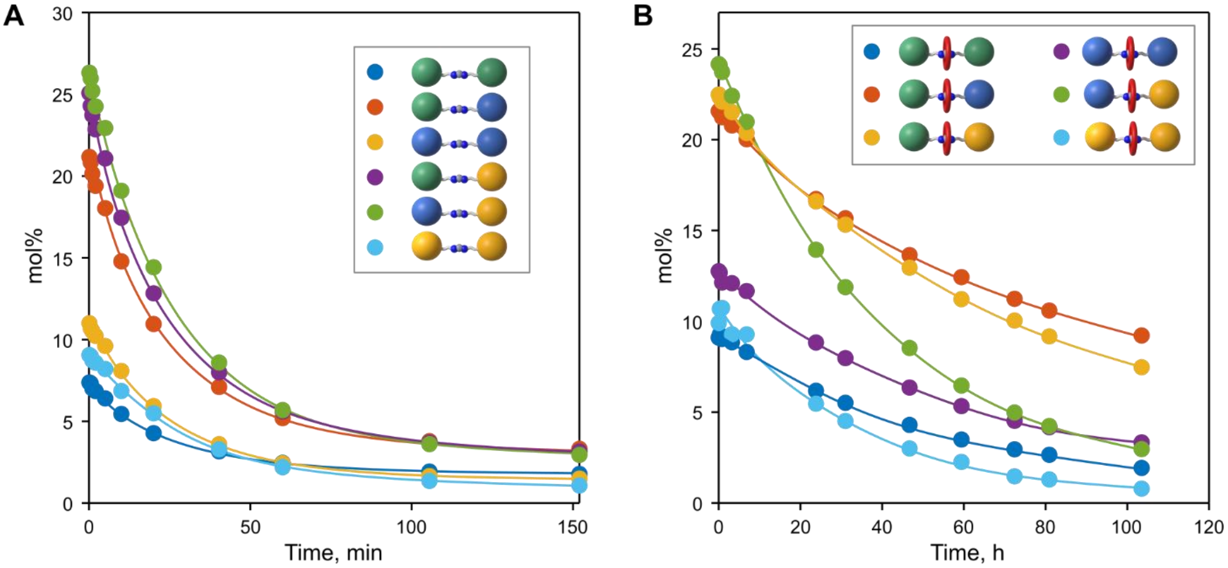

Figure S69. LCMS monitoring of the degradation of (A) the thread DCL (**2a**, **2b**, **2c**, **2a/b**, **2a/c**, **2b/c**; total amount ~ 18 µmol) and (B) the rotaxane DCL (**1a**, **1b**, **1c**, **1a/b**, **1a/c**, **1b/c**, total amount ~ 6 µmol) upon reaction with *isopropylamine* (see the general procedure above). Reaction conditions: total concentration of the amidinium species – 0.06 M; solvent – THF; room temperature. Mole percentages of all DCL members were calculated as the amount of a DCL member divided by the total amount of the amidinium species.

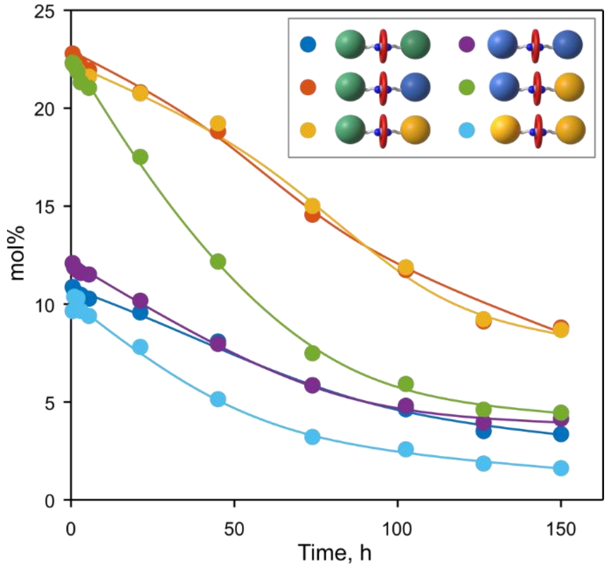

Figure S70. LCMS monitoring of the degradation of the rotaxane DCL (**1a**, **1b**, **1c**, **1a/b**, **1a/c**, **1b/c**, total amount ~ 6 µmol) upon reaction with *tert-butylamine* (see the general procedure above). Reaction conditions: total concentration of the amidinium species – 0.06 M; solvent – THF; 45 °C. Mole percentages of all DCL members were calculated as the amount of a DCL member divided by the total amount of the amidinium species.

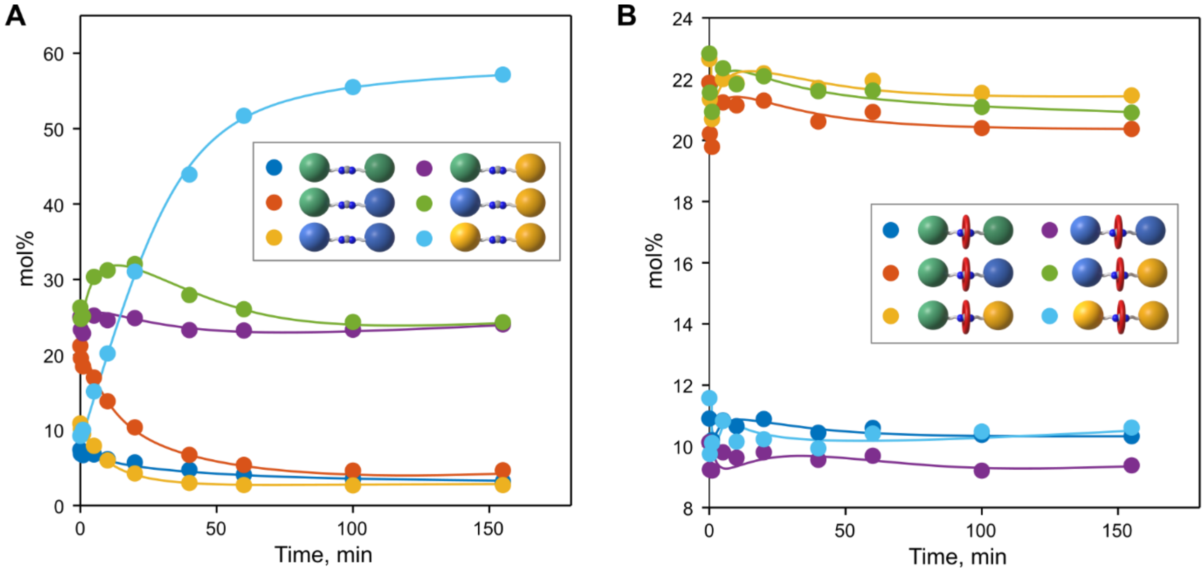

Figure S71. LCMS monitoring of the reconfiguration of (A) the thread subDCL (**2a**, **2b**, **2c**, **2a/b**, **2a/c**, **2b/c**; total amount ~ 6 µmol) and (B) the rotaxane subDCL (**1a**, **1b**, **1c**, **1a/b**, **1a/c**, **1b/c**, total amount ~ 6 µmol) upon reaction with amine **4b** (see the general procedure above). Reaction conditions: total concentration of the amidinium species (both subDCLs combined) – 0.11 M; solvent – THF; room temperature. Mole percentages of all DCL members were calculated as the amount of a DCL member divided by the total amount of the amidinium species in each subDCL. The amidinium DCL was prepared by mixing equimolar amounts (6 µmol each) of the purified thread DCL (HCO_2^- form) and rotaxane DCL (HCO_2^- form).

10. NMR spectra

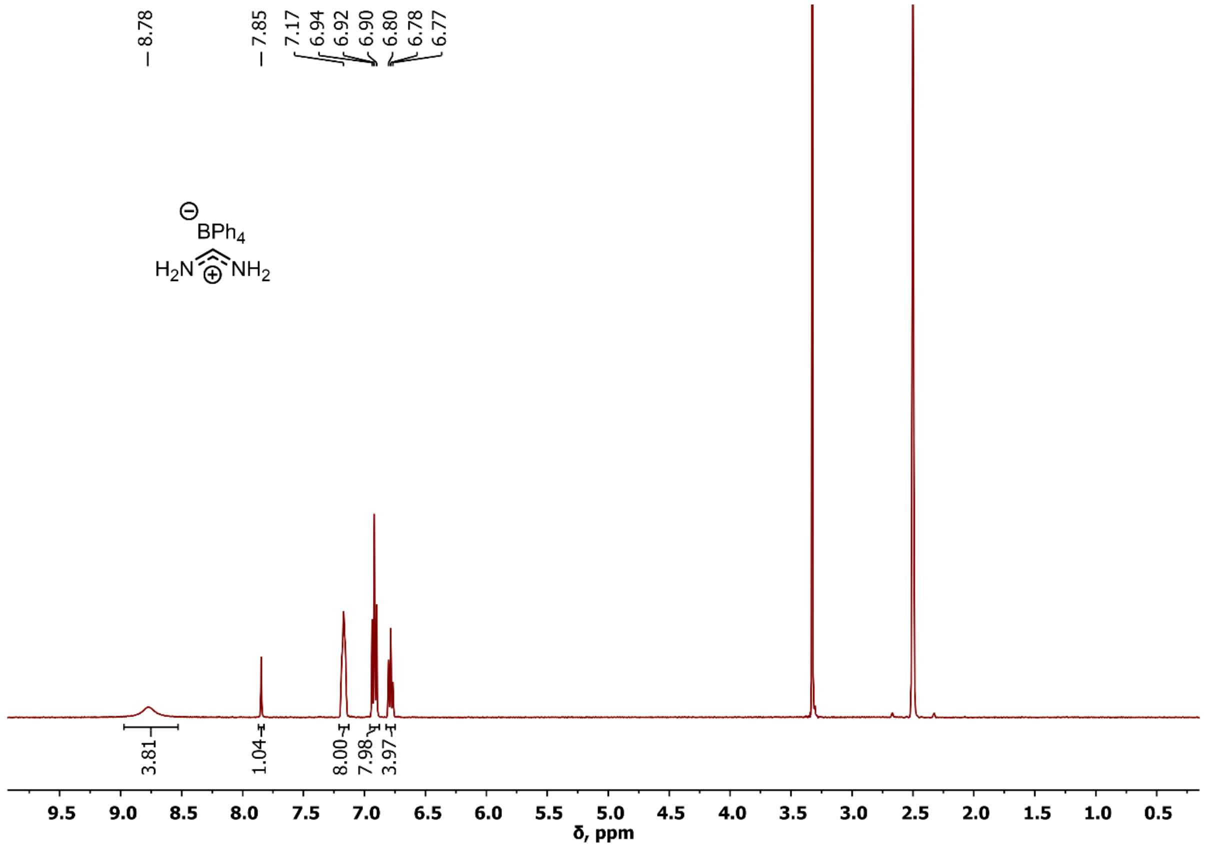

Figure S72. ^{1}H NMR spectrum of **FA·BPh$_4$** (400 MHz, DMSO-d_6, 295 K). Singlet at 3.33 ppm belongs to H_2O.

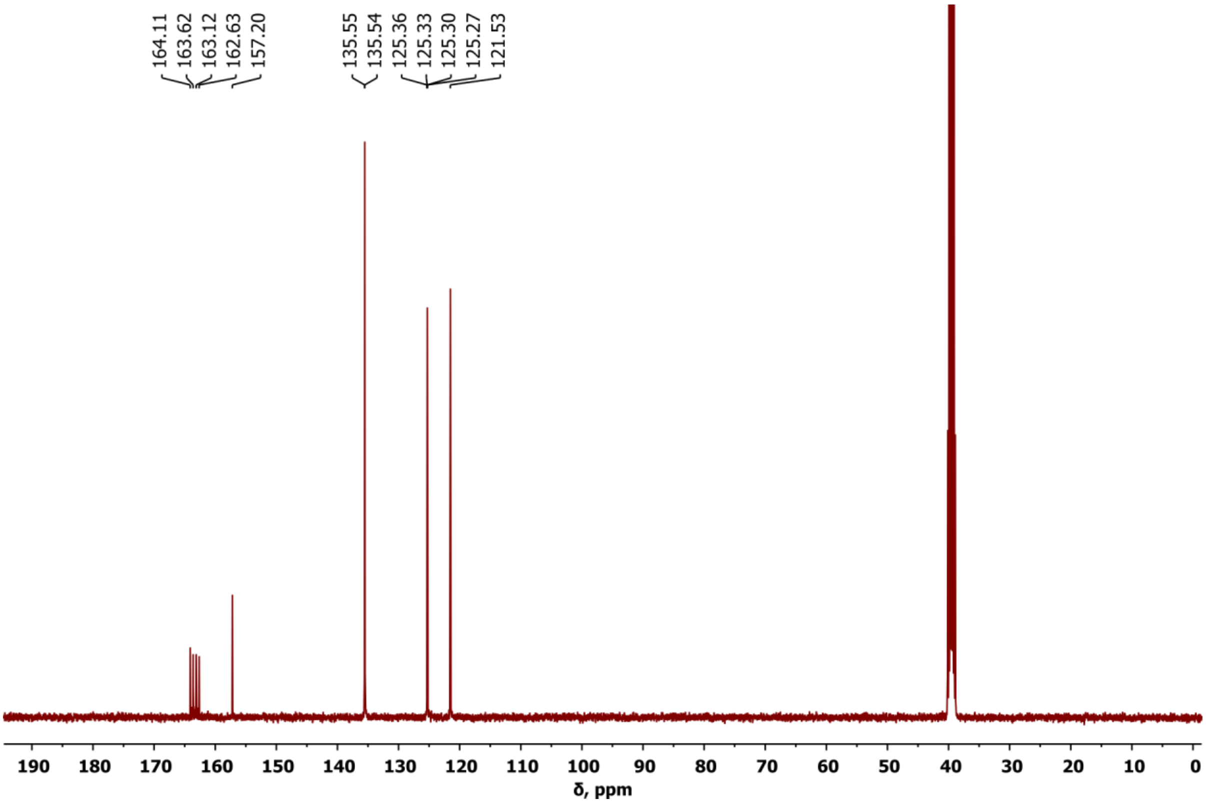

Figure S73. ^{13}C NMR spectrum of **FA·BPh$_4$** (101 MHz, DMSO-d_6, 295 K).

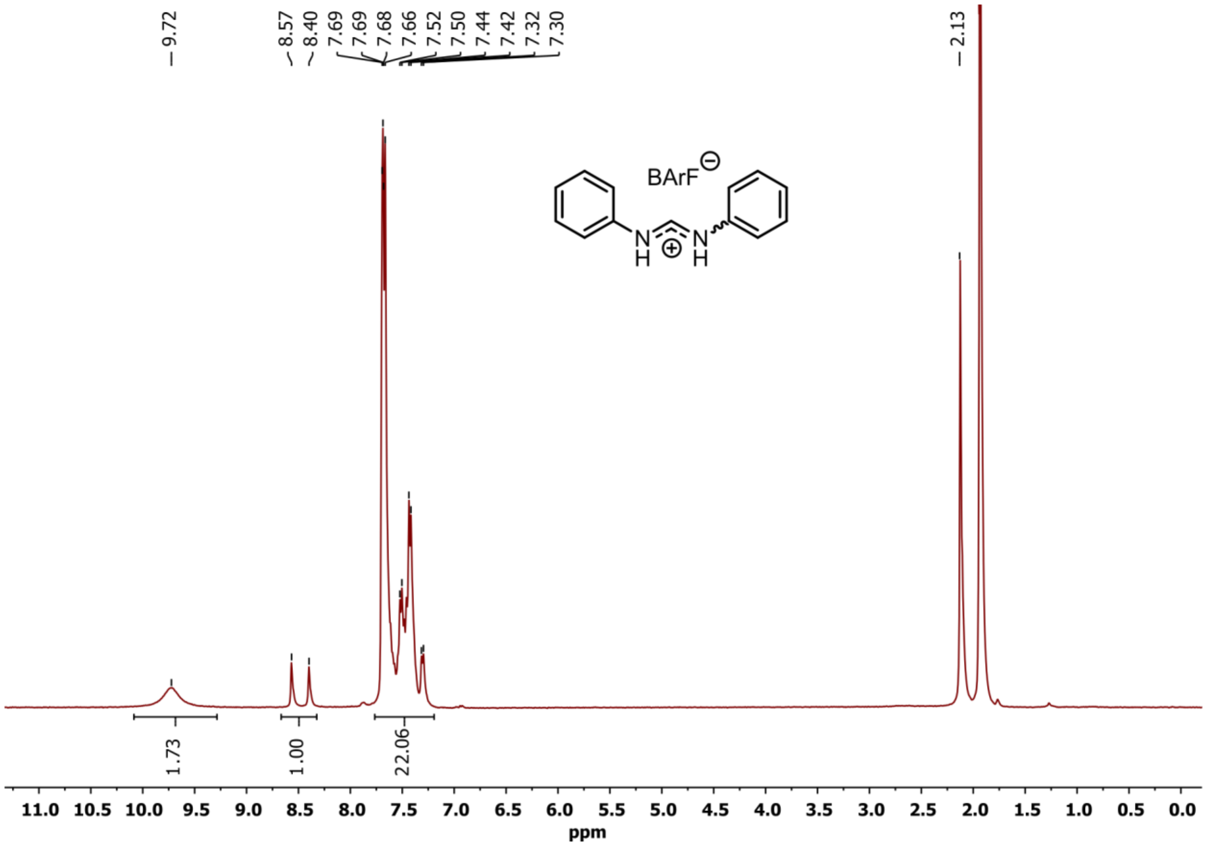

Figure S74. ^{1}H NMR spectrum of **DPFA·BArF** (400 MHz, CD_3CN, 302 K). Singlet at 2.13 ppm belongs to H_2O.

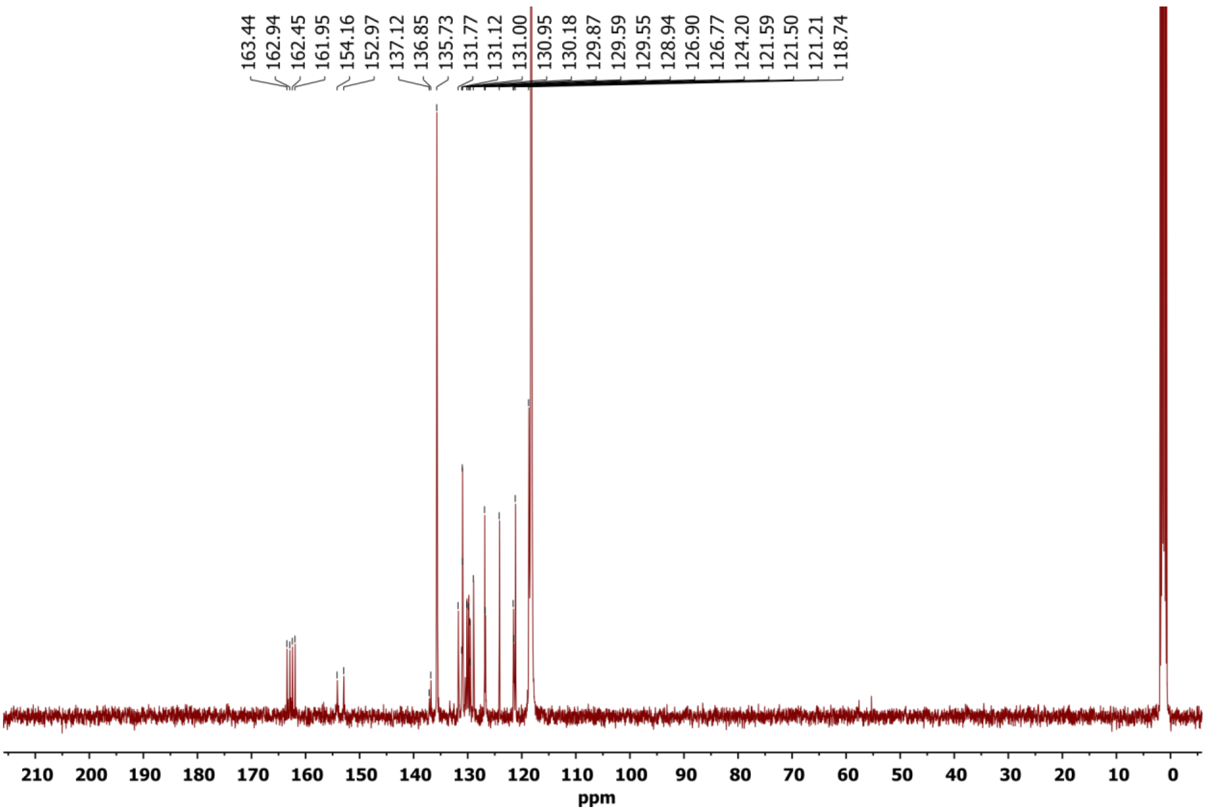

Figure S75. ^{13}C NMR spectrum of **DPFA·BArF** (101 MHz, CD_3CN, 295 K).

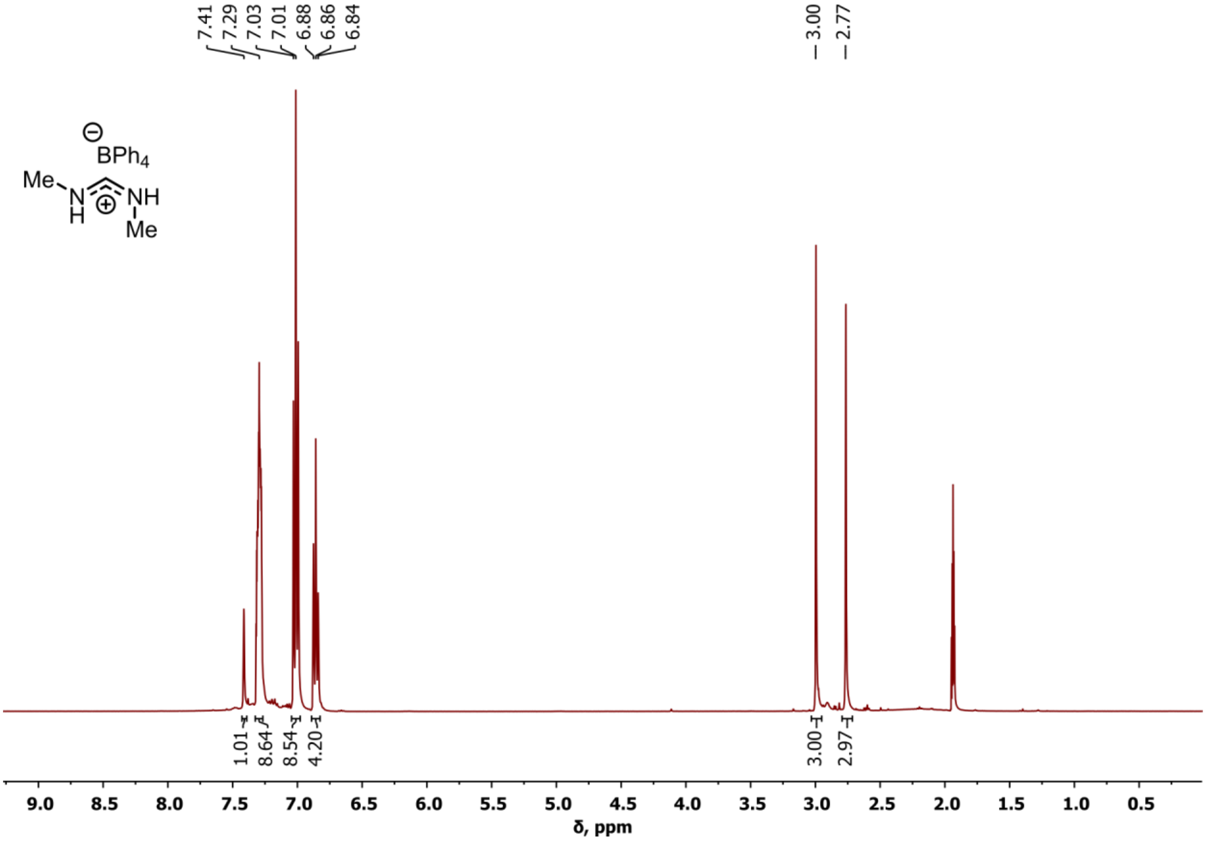

Figure S76. ^{1}H NMR spectrum of **DMFA·BPh₄** (400 MHz, CD_3CN, 295 K).

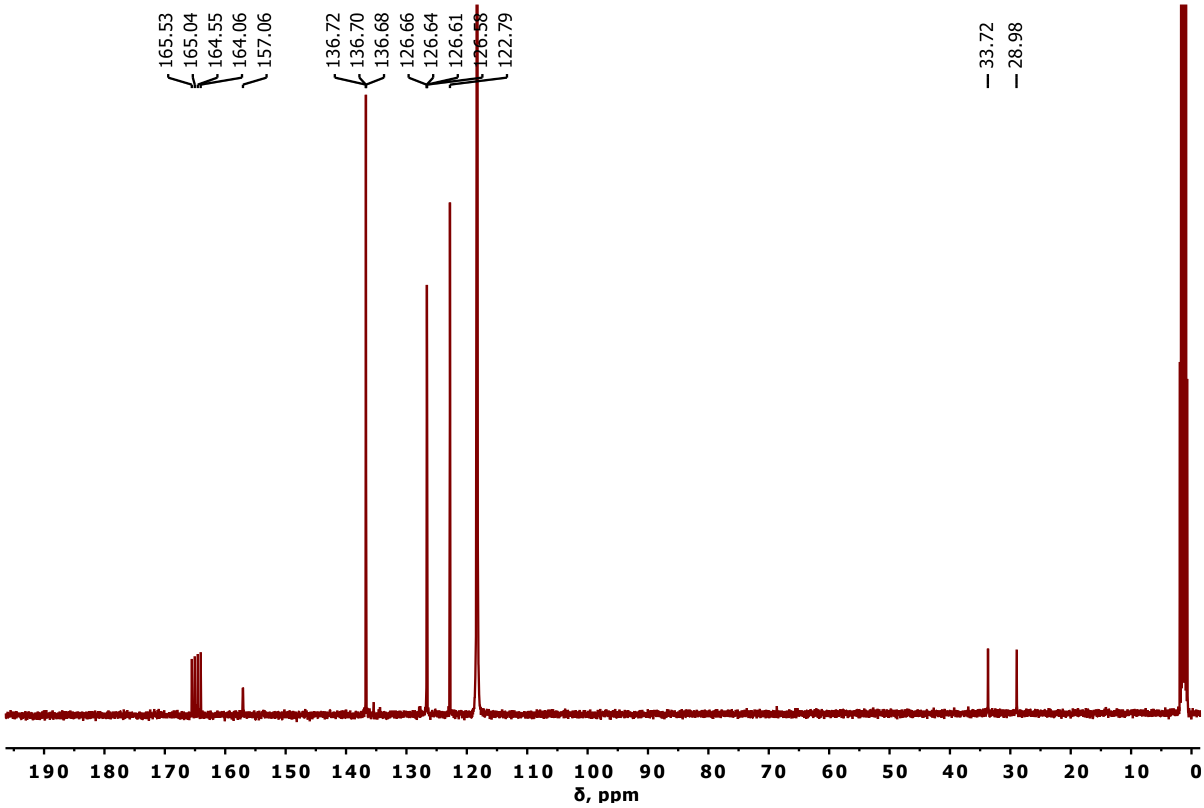

Figure S77. ^{13}C NMR spectrum of **DMFA·BPh₄** (101 MHz, CD_3CN, 295 K).

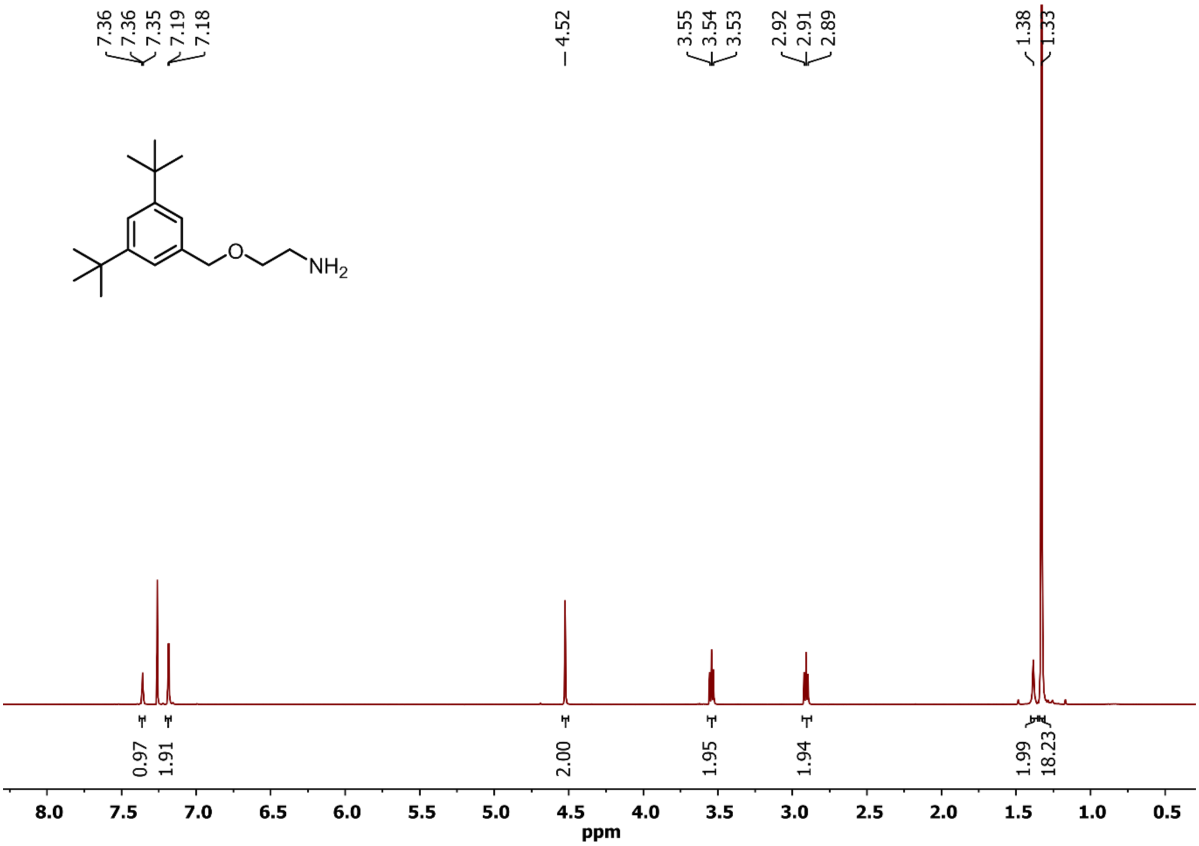

Figure S78. ^{1}H NMR spectrum of **4b** (400 MHz, $CDCl_3$, 295 K).

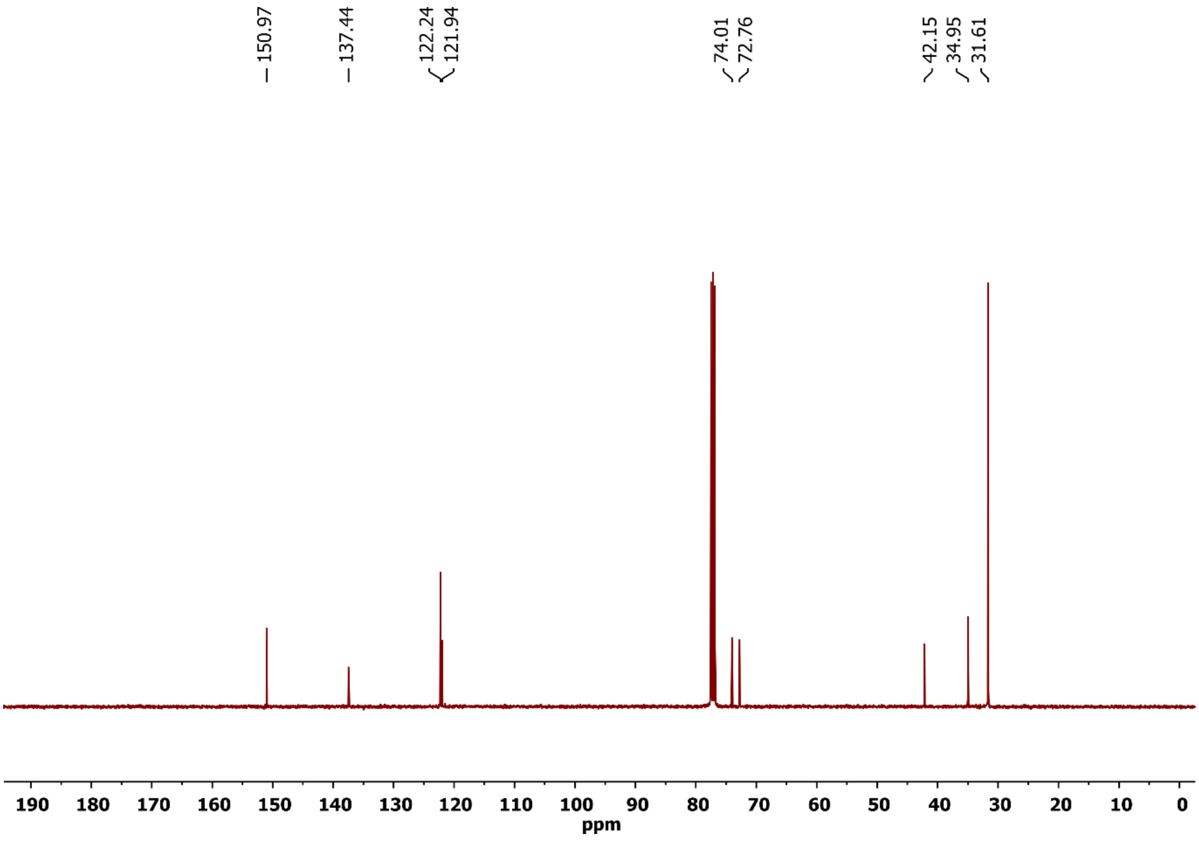

Figure S79. ^{13}C NMR spectrum of **4b** (101 MHz, $CDCl_3$, 295 K).

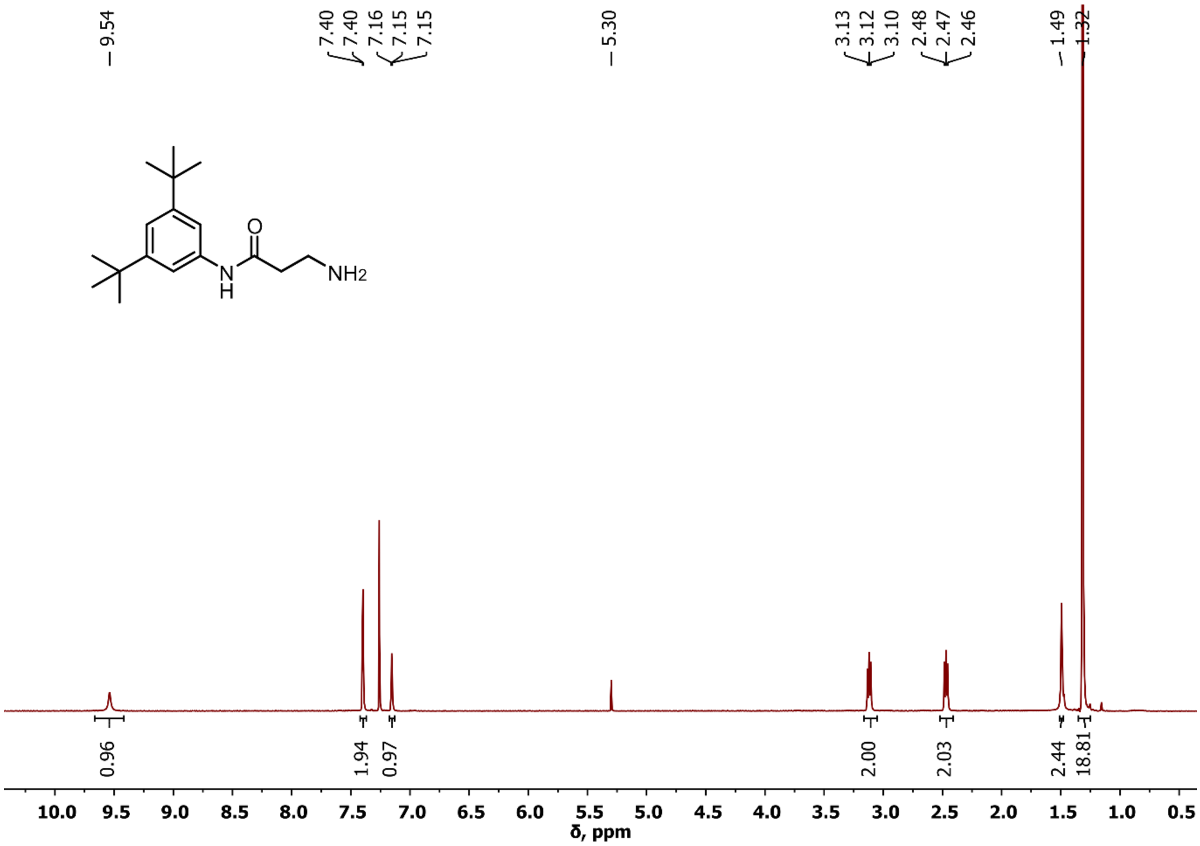

Figure S80. ^{1}H NMR spectrum of **4c** (400 MHz, $CDCl_3$, 295 K).

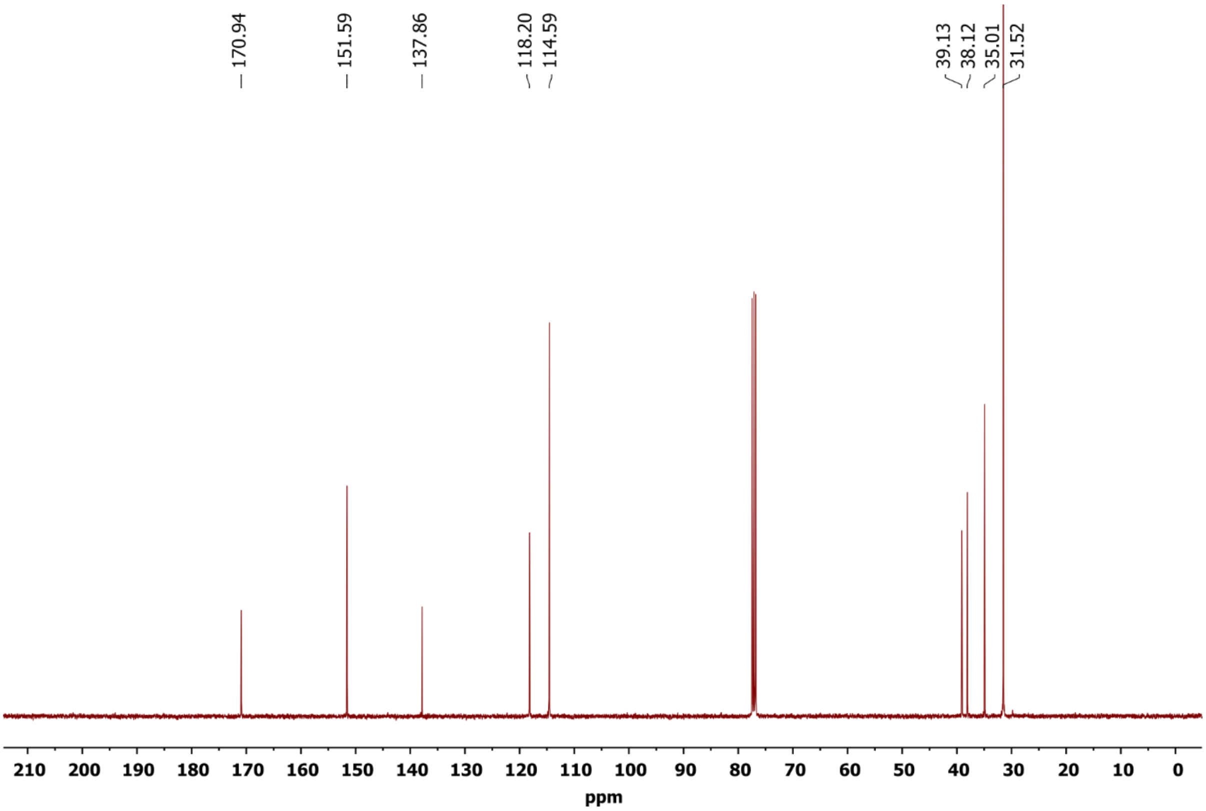

Figure S81. ^{13}C NMR spectrum of **4c** (101 MHz, $CDCl_3$, 295 K).

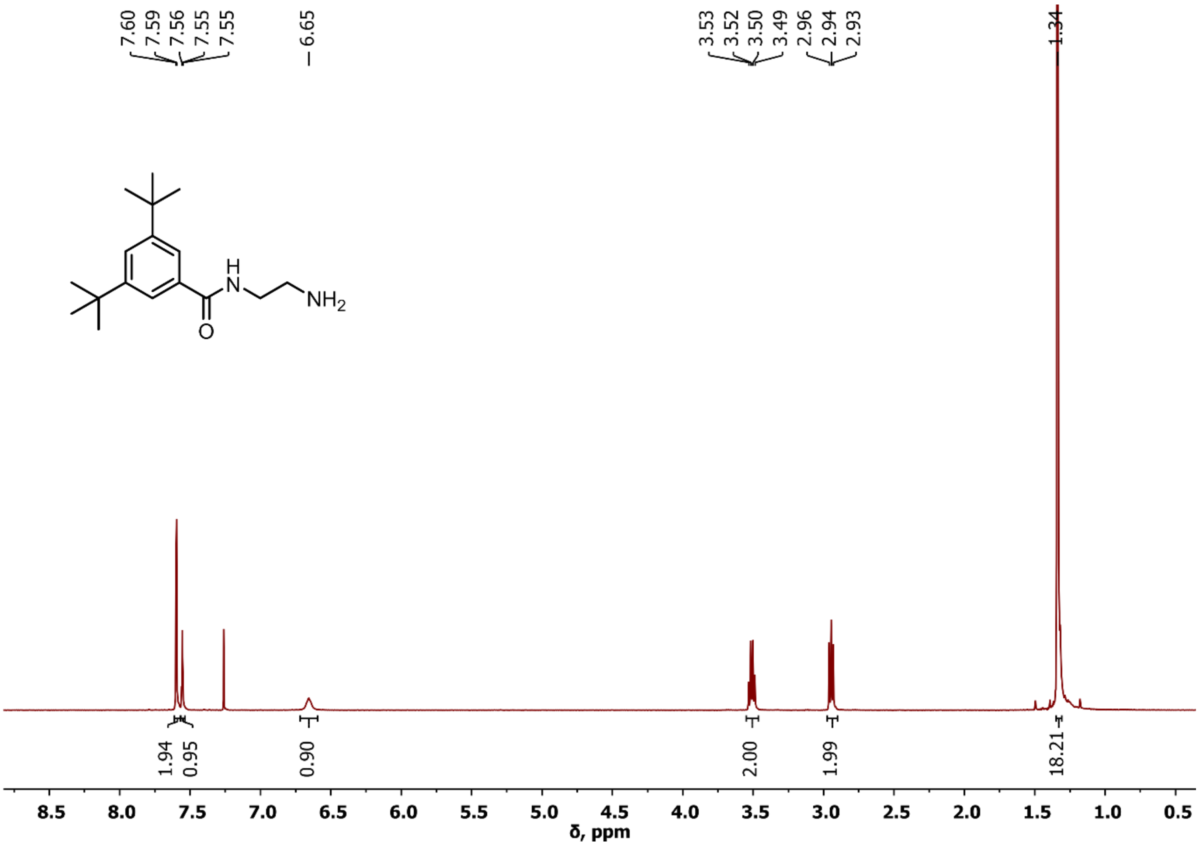

Figure S82. ^{1}H NMR spectrum of **4d** (400 MHz, $CDCl_3$, 295 K).

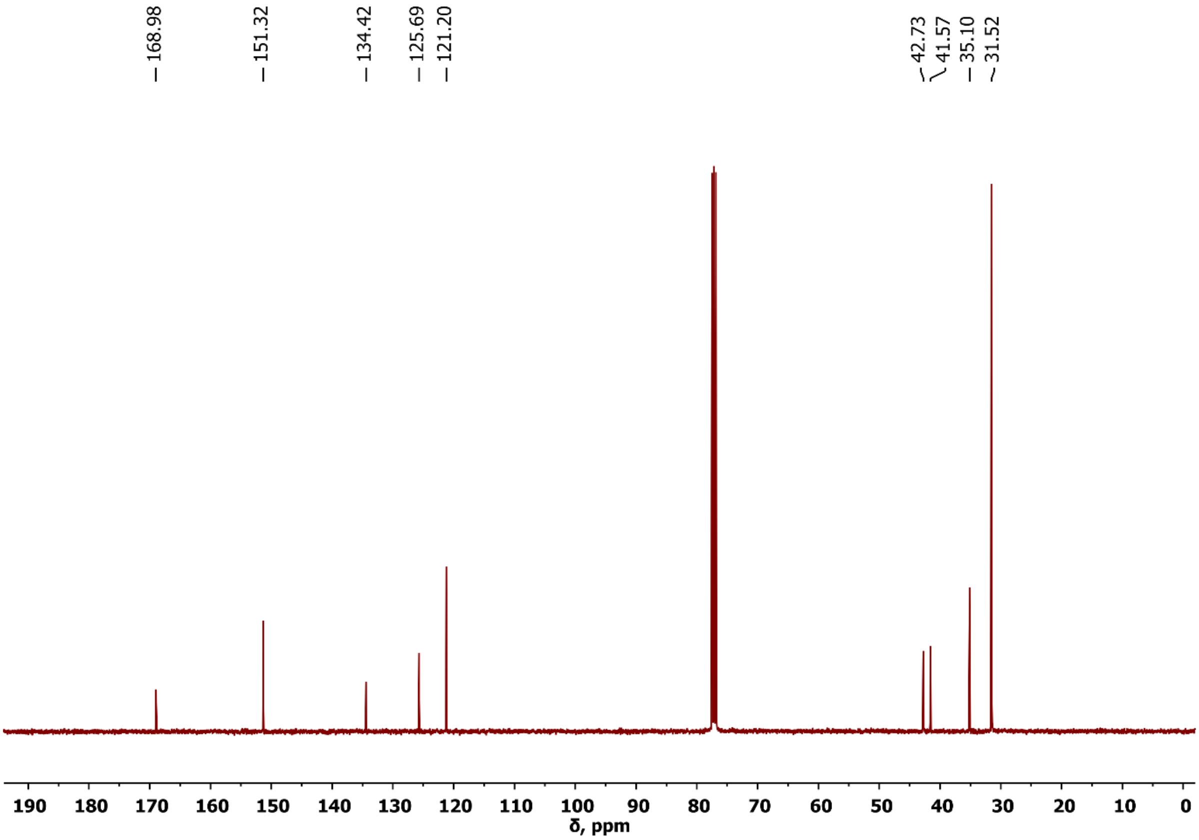

Figure S83. ^{13}C NMR spectrum of **4d** (101 MHz, $CDCl_3$, 295 K).

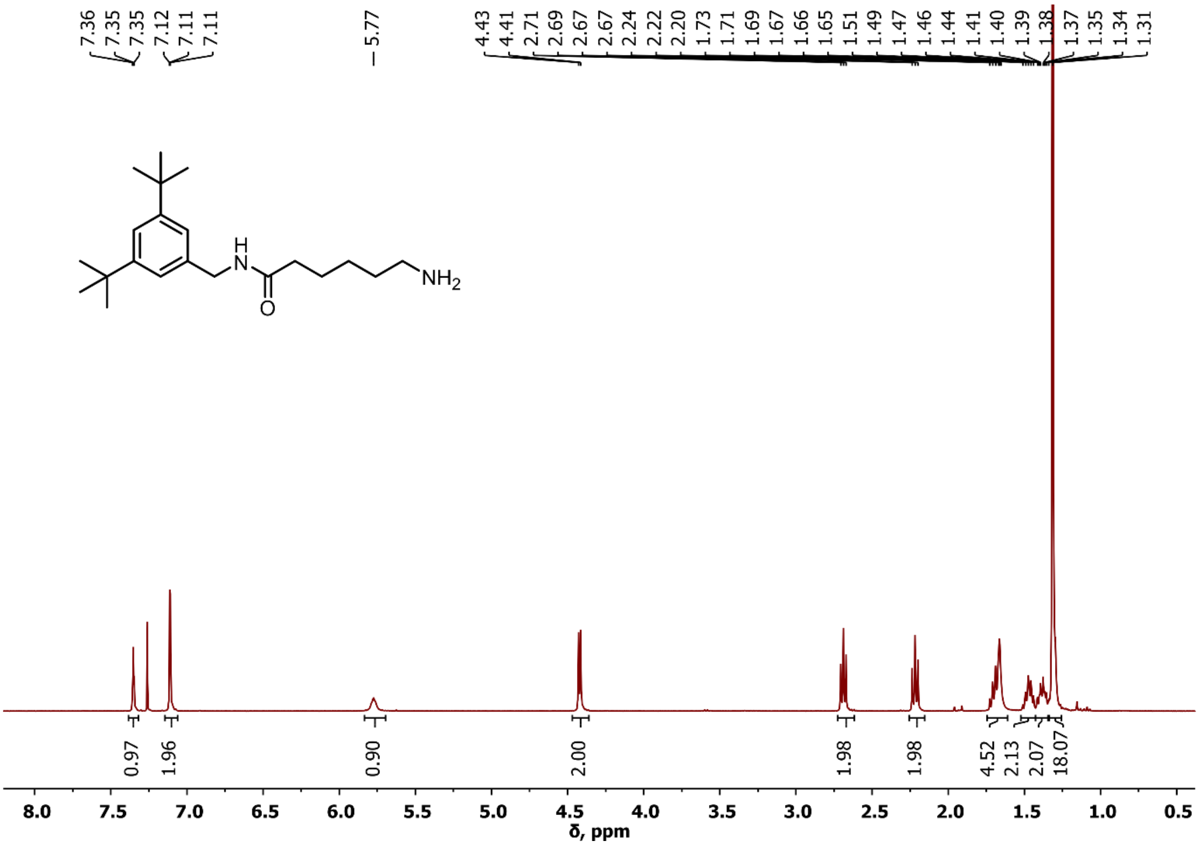

Figure S84. ^{1}H NMR spectrum of **4e** (400 MHz, $CDCl_3$, 295 K).

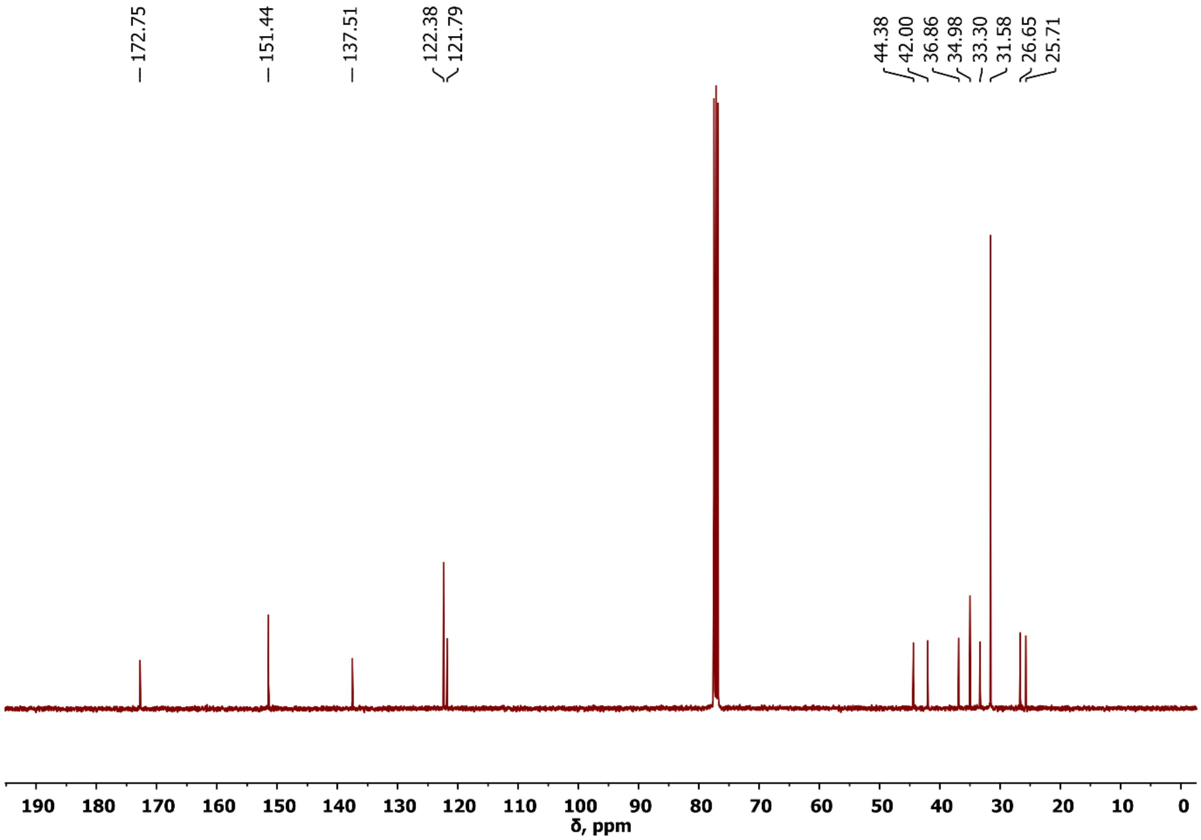

Figure S85. ^{13}C NMR spectrum of **4e** (101 MHz, $CDCl_3$, 295 K).

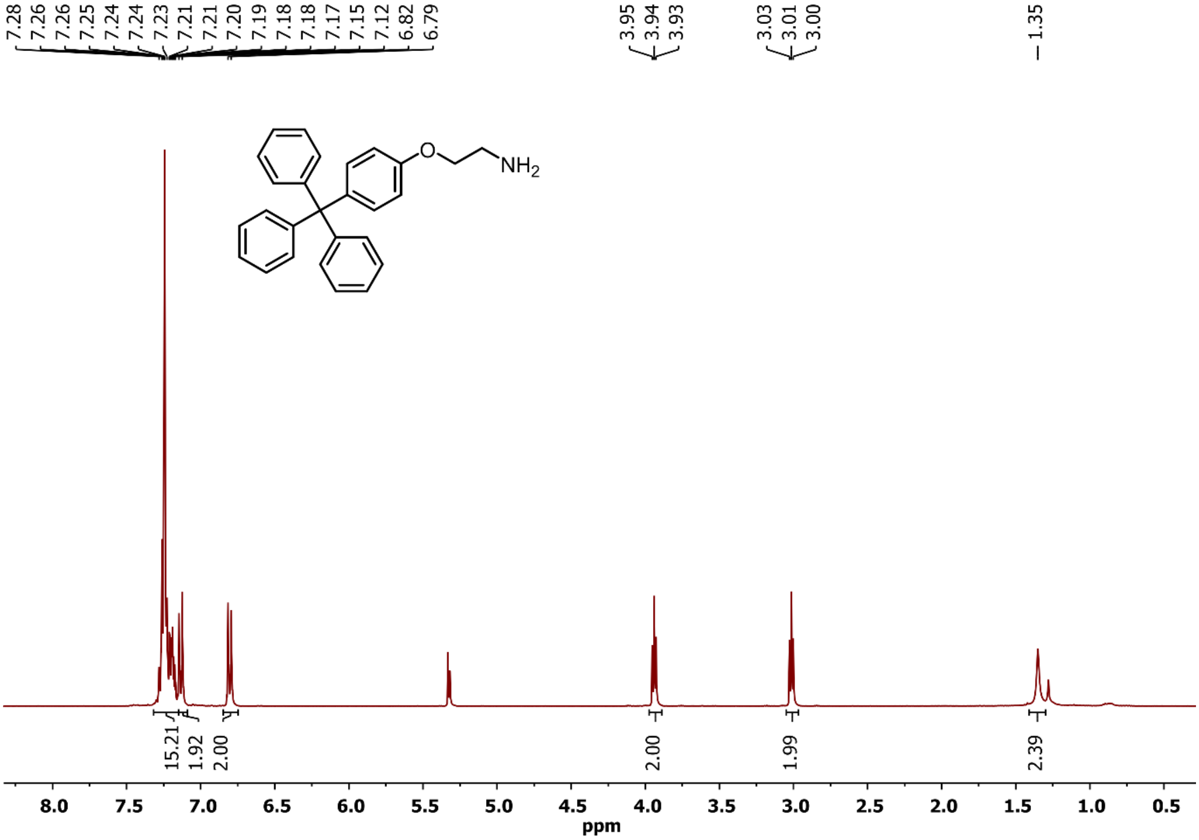

Figure S86. ^{1}H NMR spectrum of **4f** (400 MHz, CD_2Cl_2, 295 K).

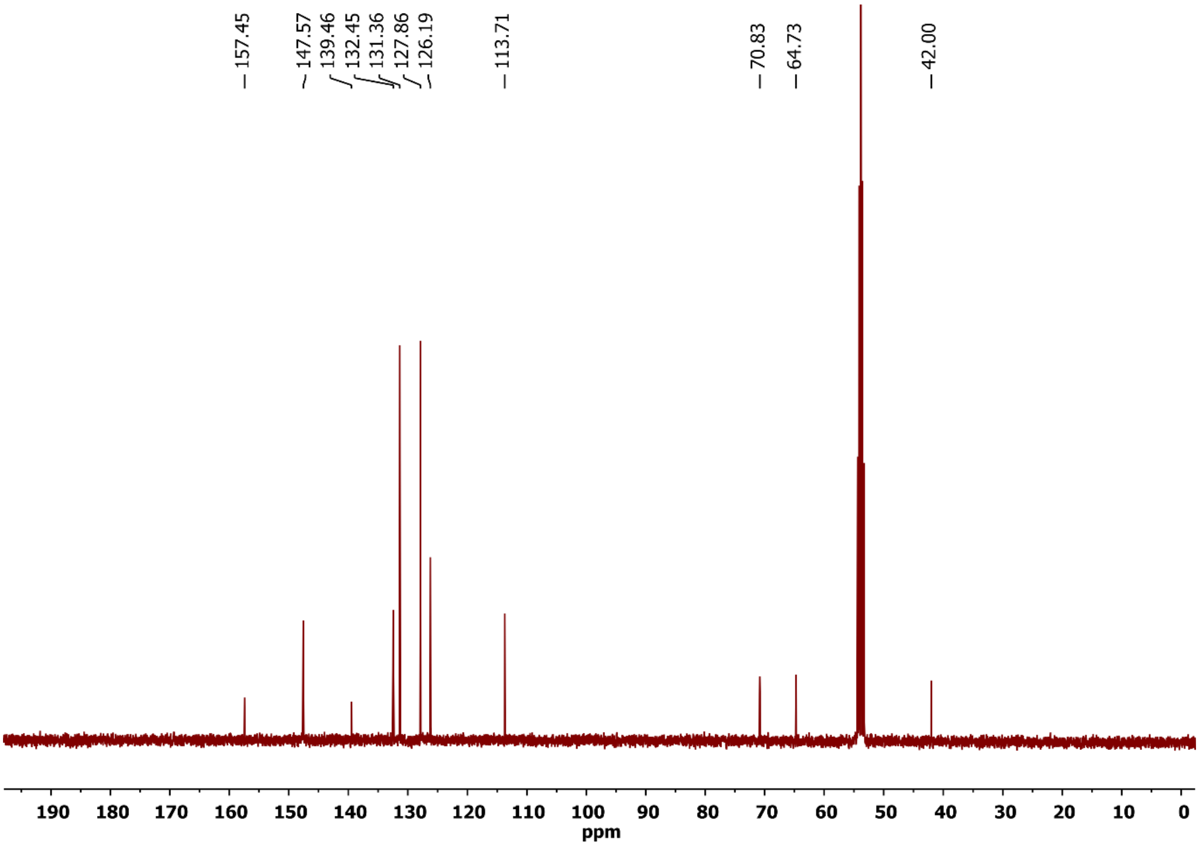

Figure S87. ^{13}C NMR spectrum of **4f** (101 MHz, CD_2Cl_2, 295 K).

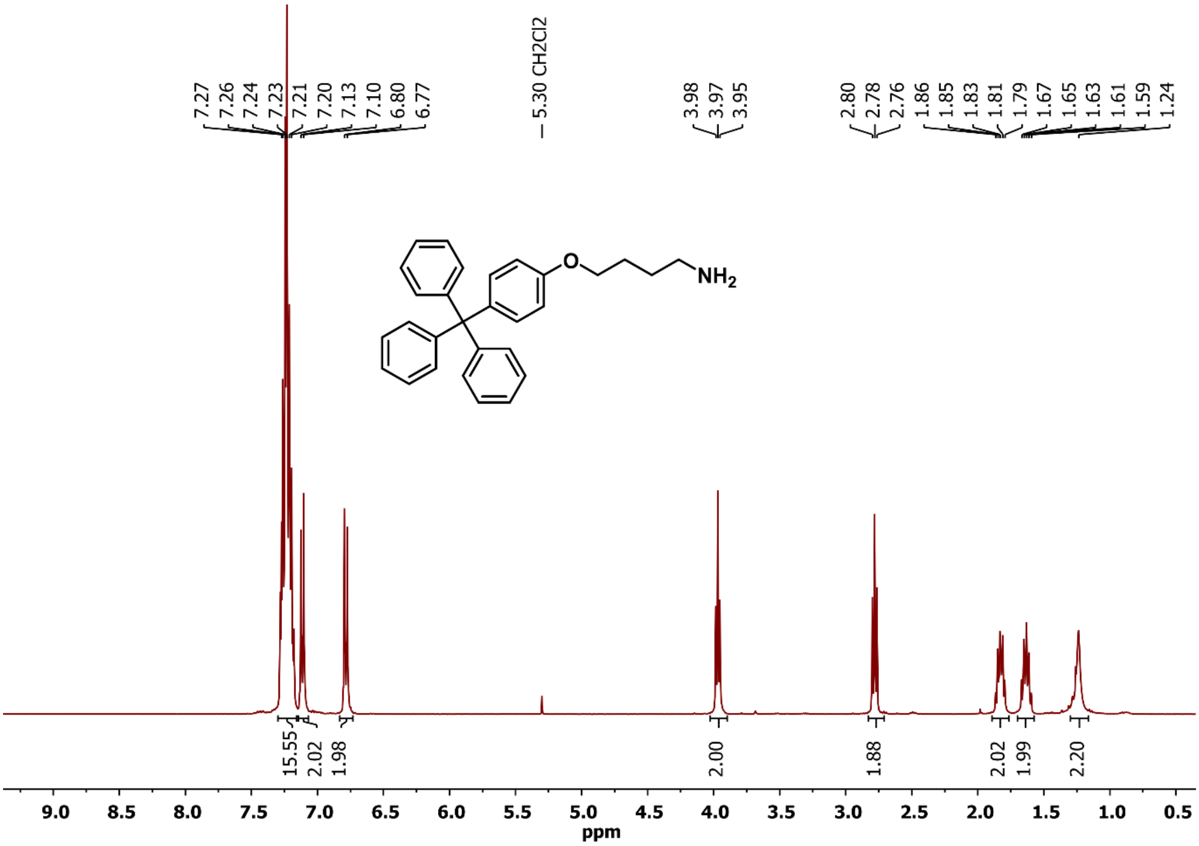

Figure S88. ^{1}H NMR spectrum of **4g** (400 MHz, $CDCl_3$, 295 K).

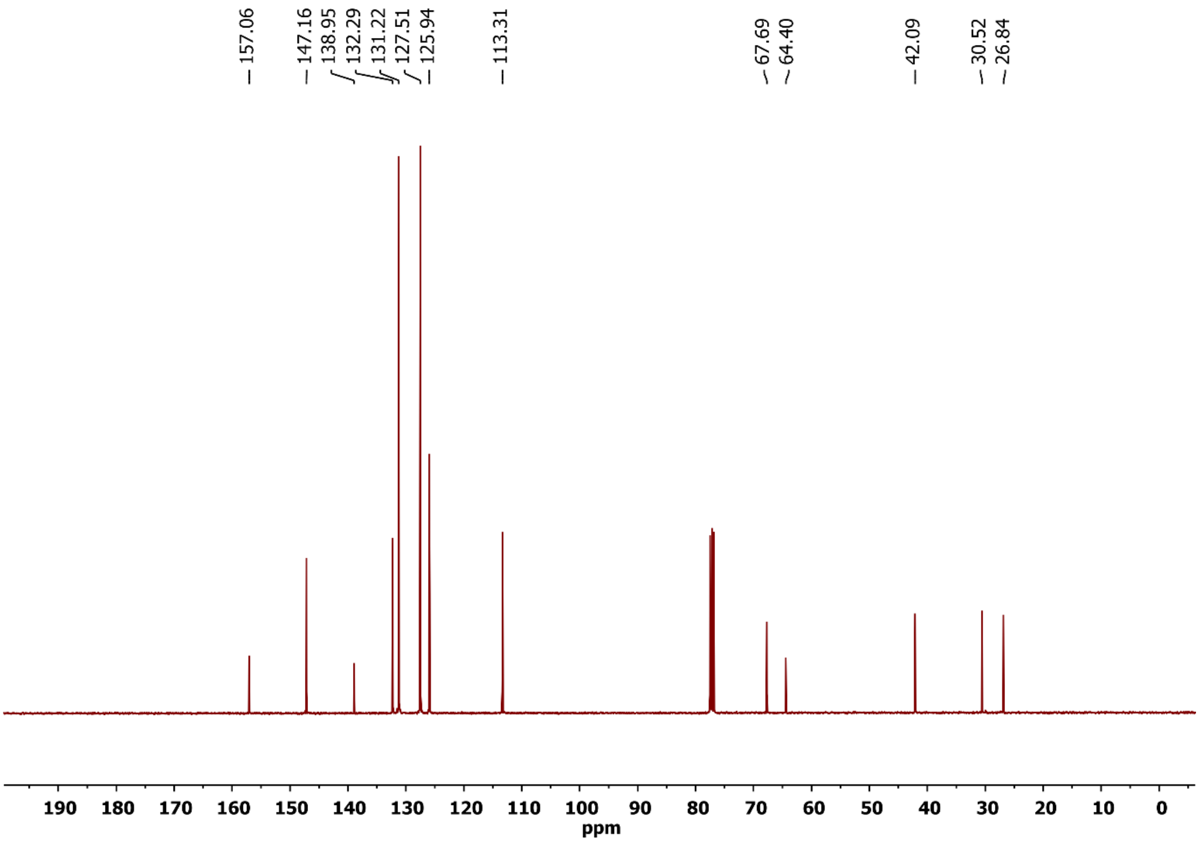

Figure S89. ^{13}C NMR spectrum of **4g** (101 MHz, $CDCl_3$, 295 K).

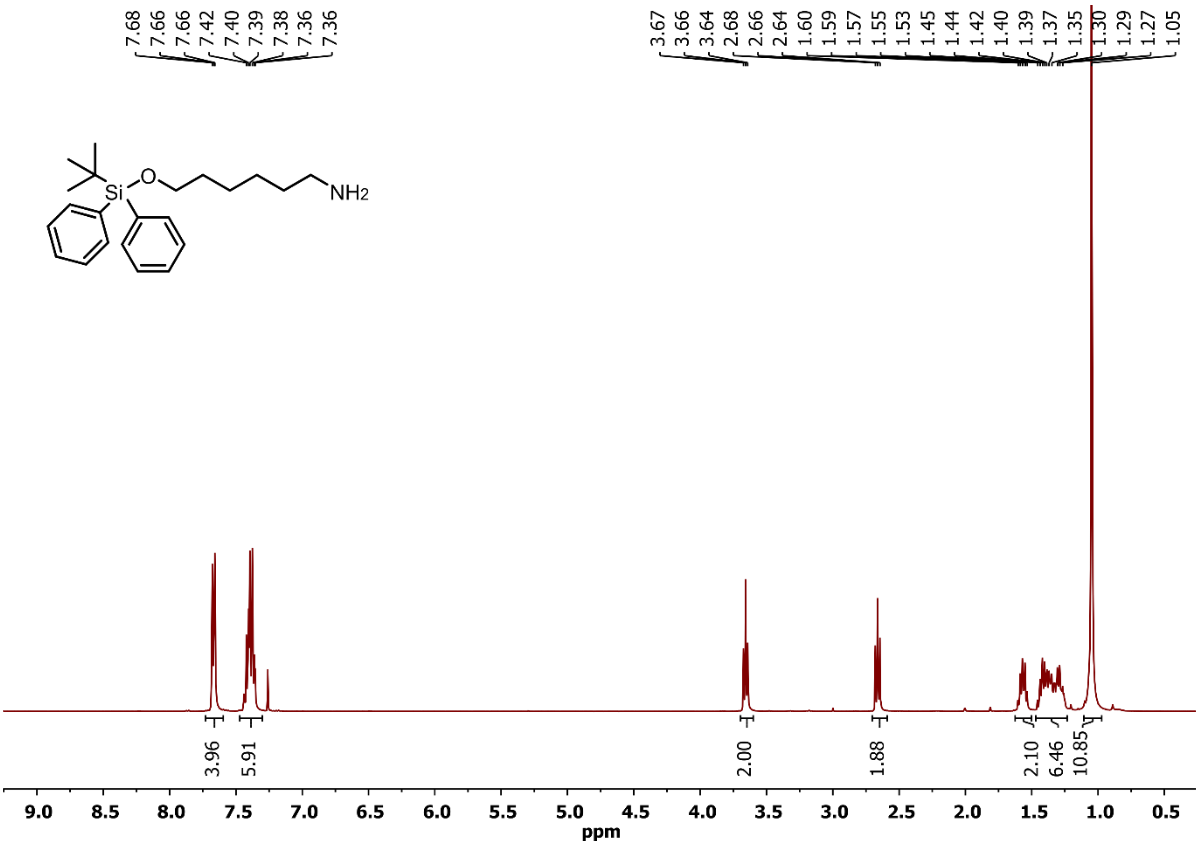

Figure S90. ^{1}H NMR spectrum of **4h** (400 MHz, $CDCl_3$, 295 K).

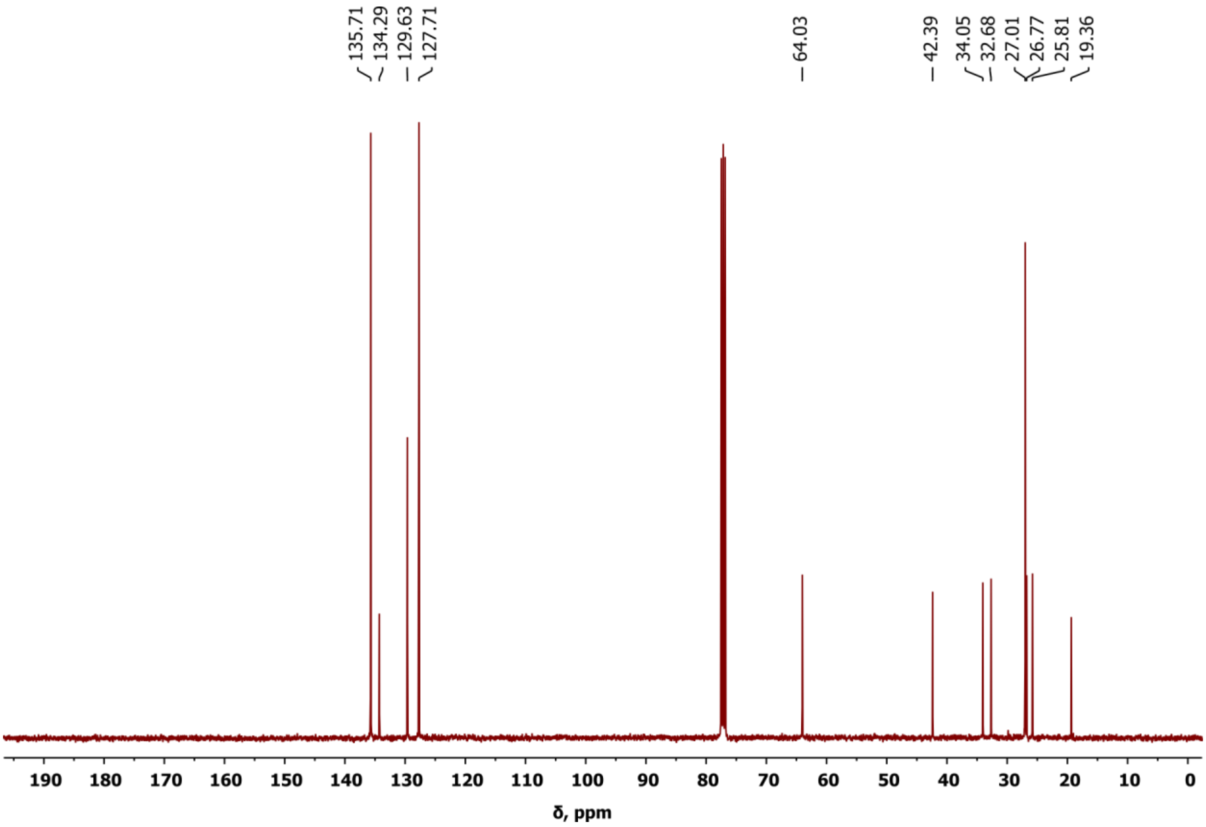

Figure S91. ^{13}C NMR spectrum of **4h** (101 MHz, $CDCl_3$, 295 K).

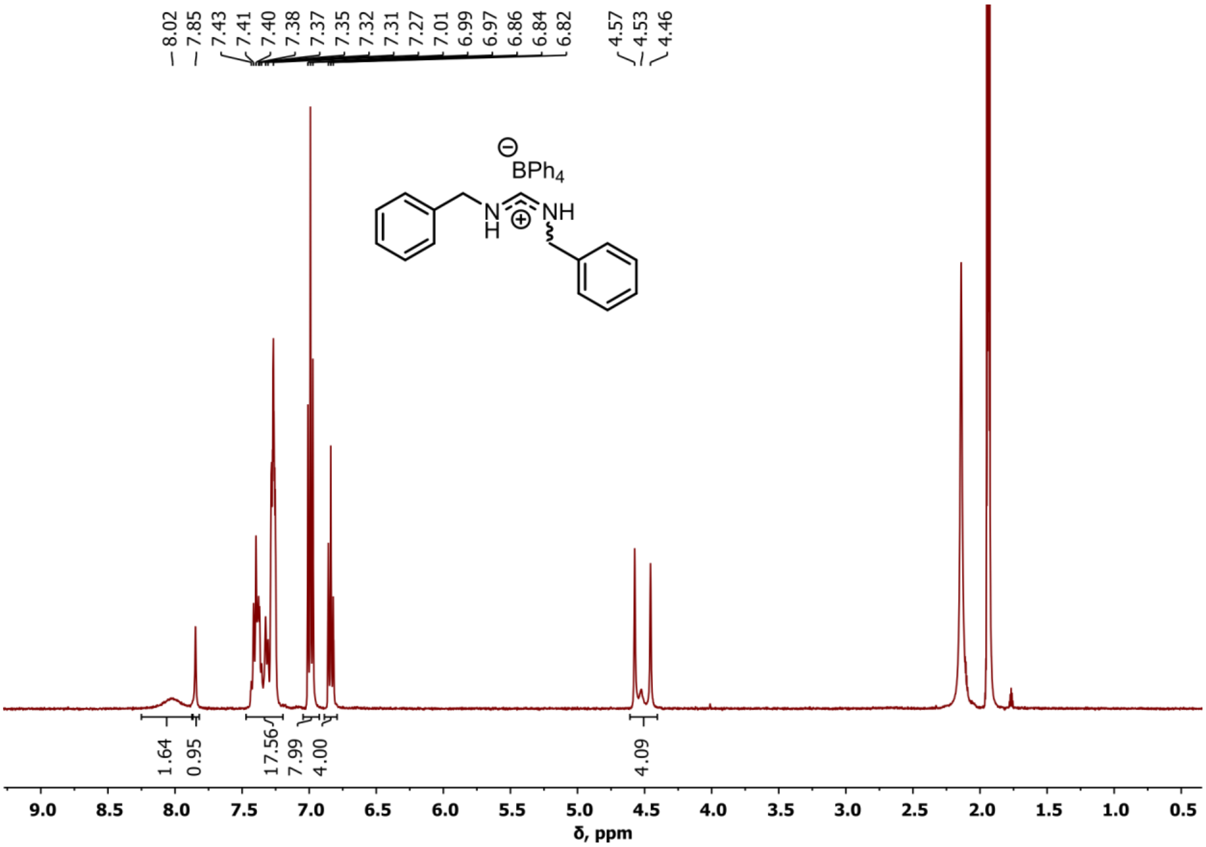

Figure S92. ^{1}H NMR spectrum of **S1** (400 MHz, CD_3CN, 295 K).

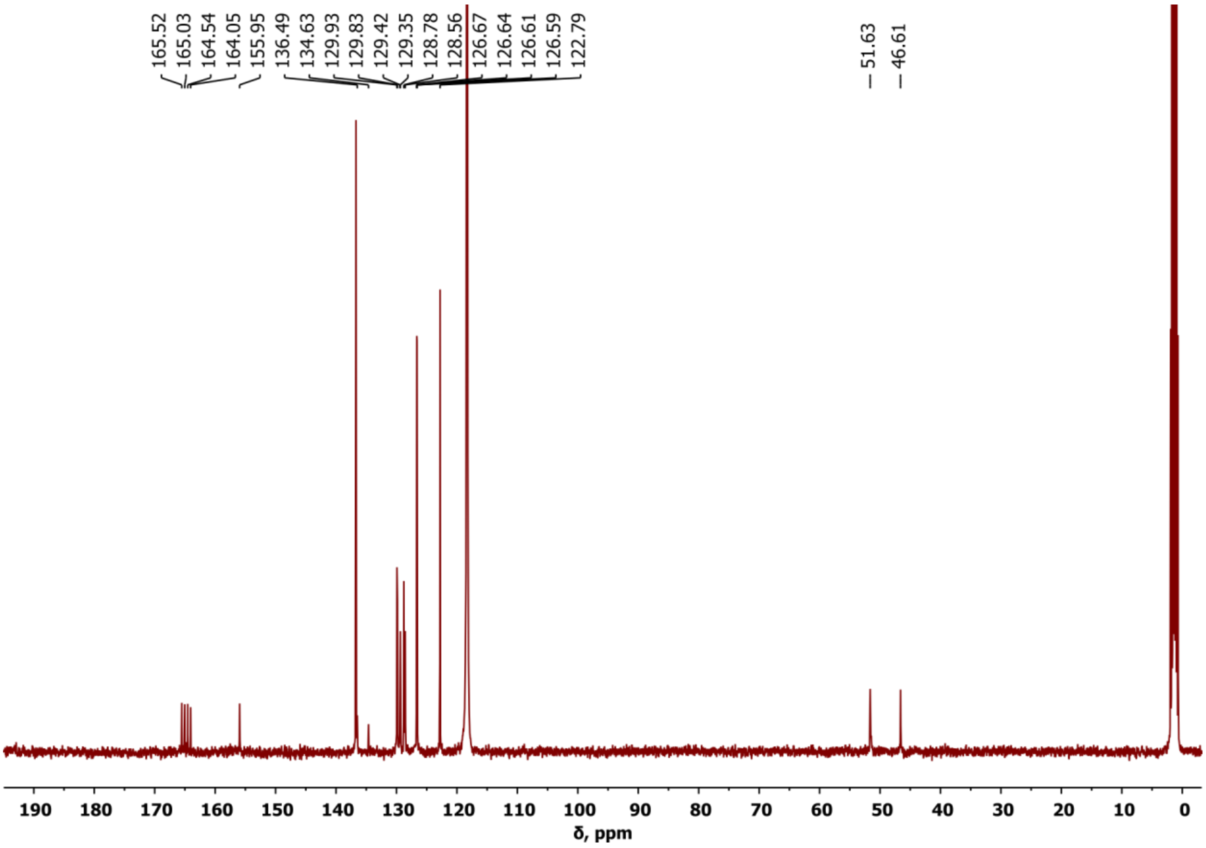

Figure S93. ^{13}C NMR spectrum of **S1** (101 MHz, CD_3CN, 295 K).

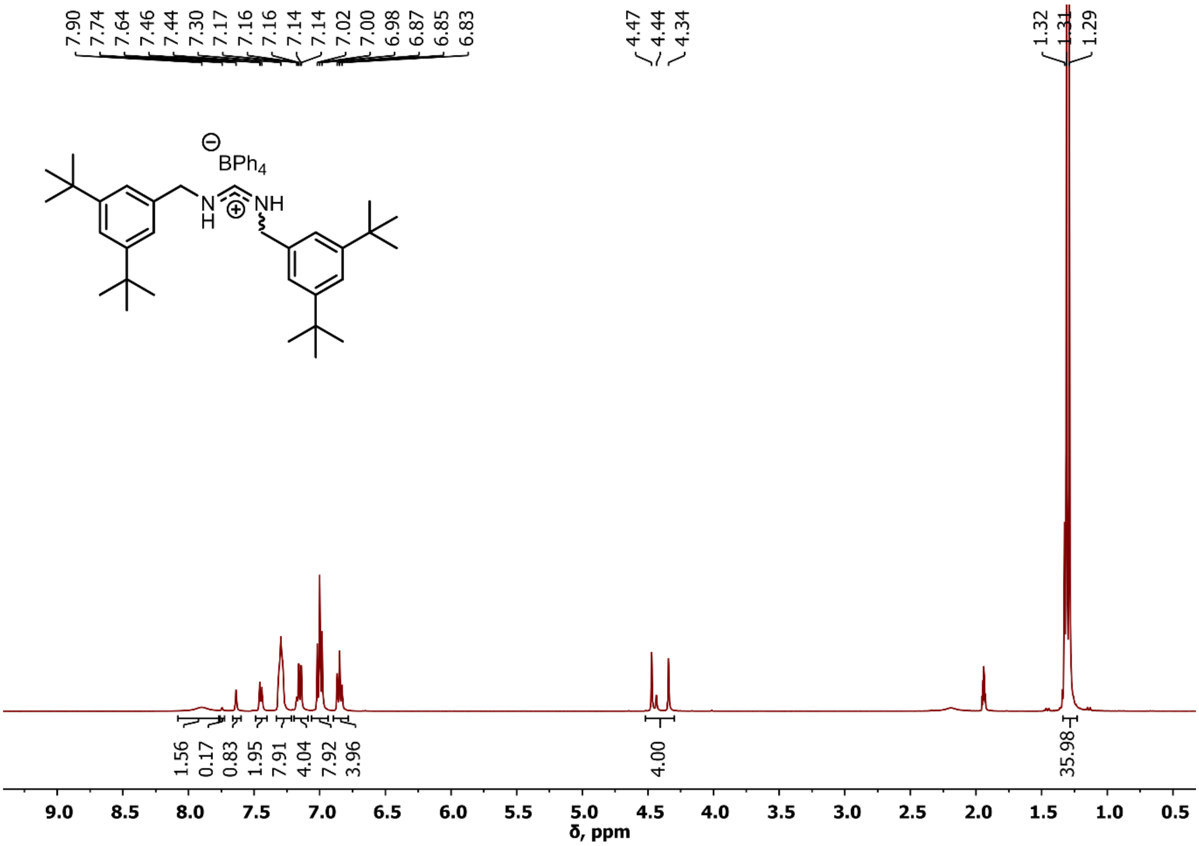

Figure S94. ^{1}H NMR spectrum of **2a** (400 MHz, CD_3CN, 295 K).

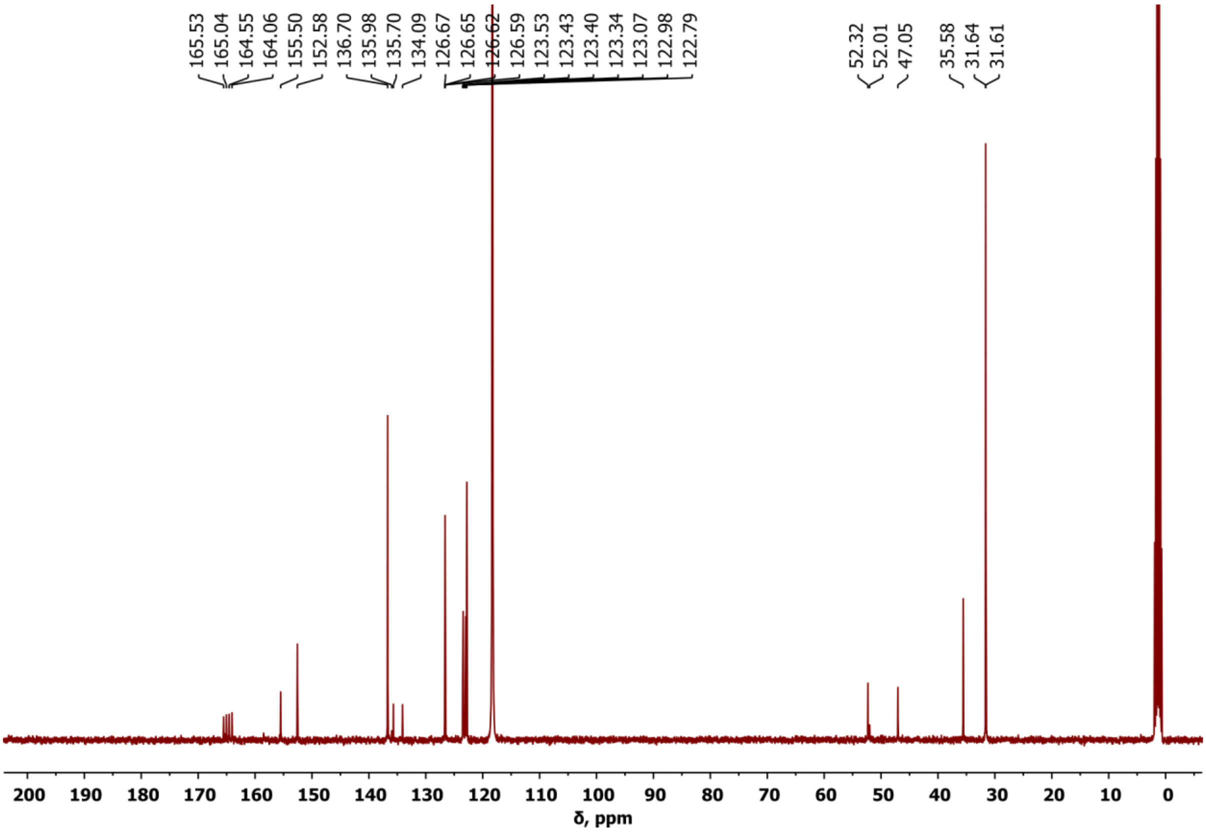

Figure S95. ^{13}C NMR spectrum of **2a** (101 MHz, CD_3CN, 295 K).

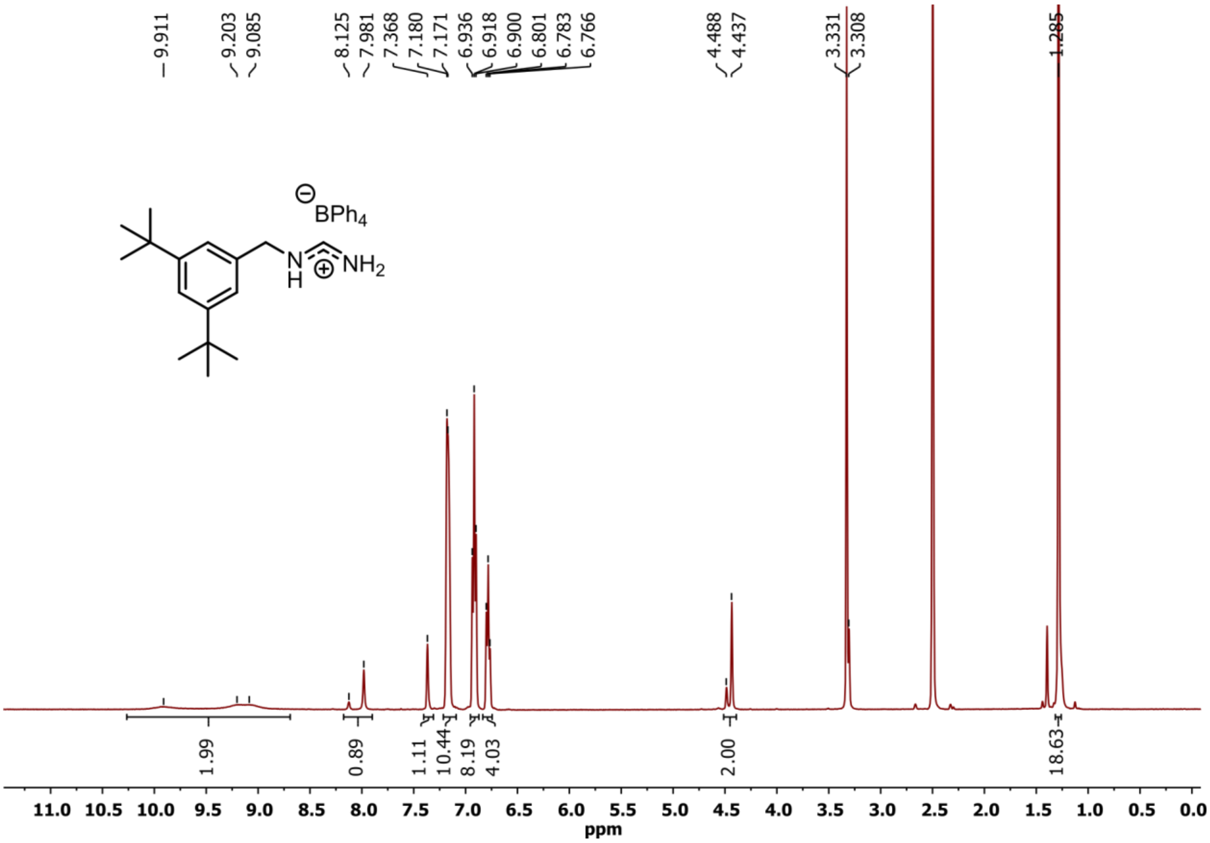

Figure S96. ^{1}H NMR spectrum of **3a** (400 MHz, DMSO-*d*$_6$, 295 K).

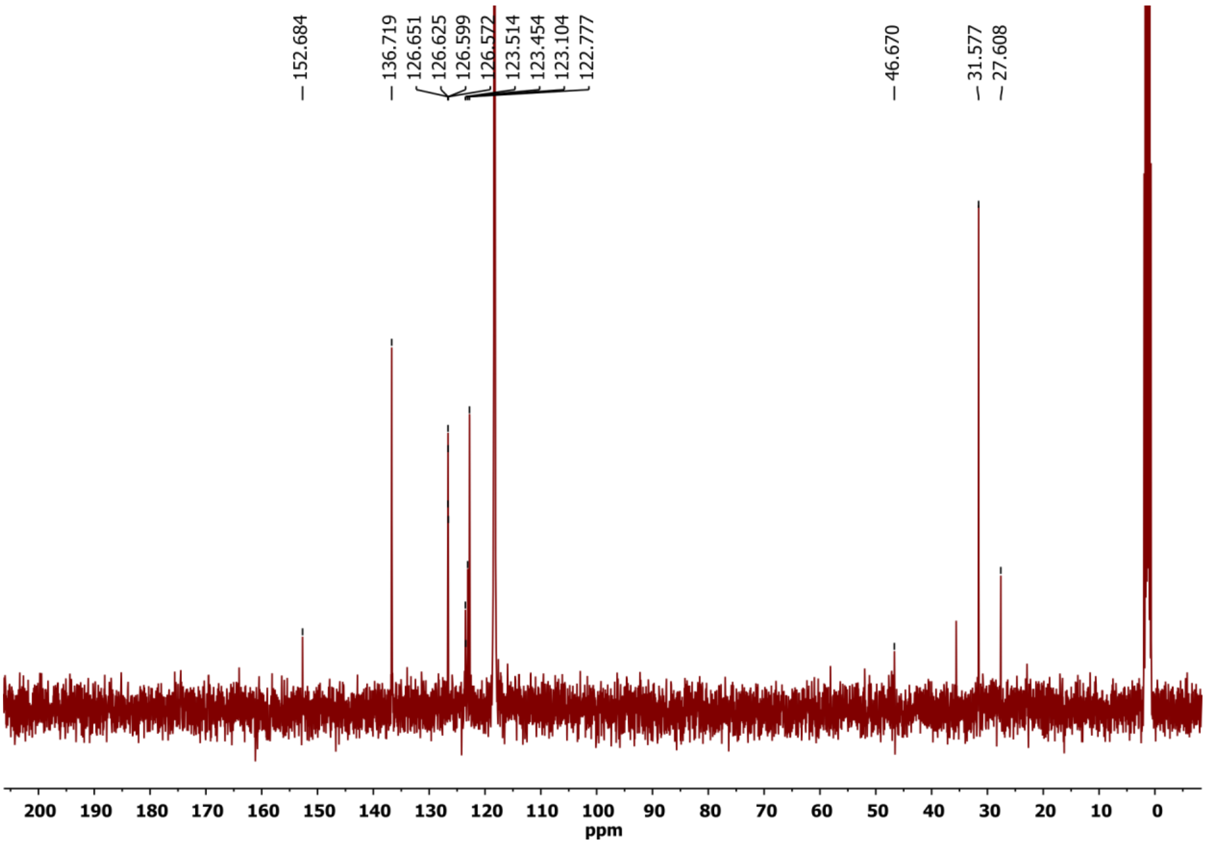

Figure S97. ^{13}C NMR spectrum of **3a** (101 MHz, CD_3CN, 296 K).

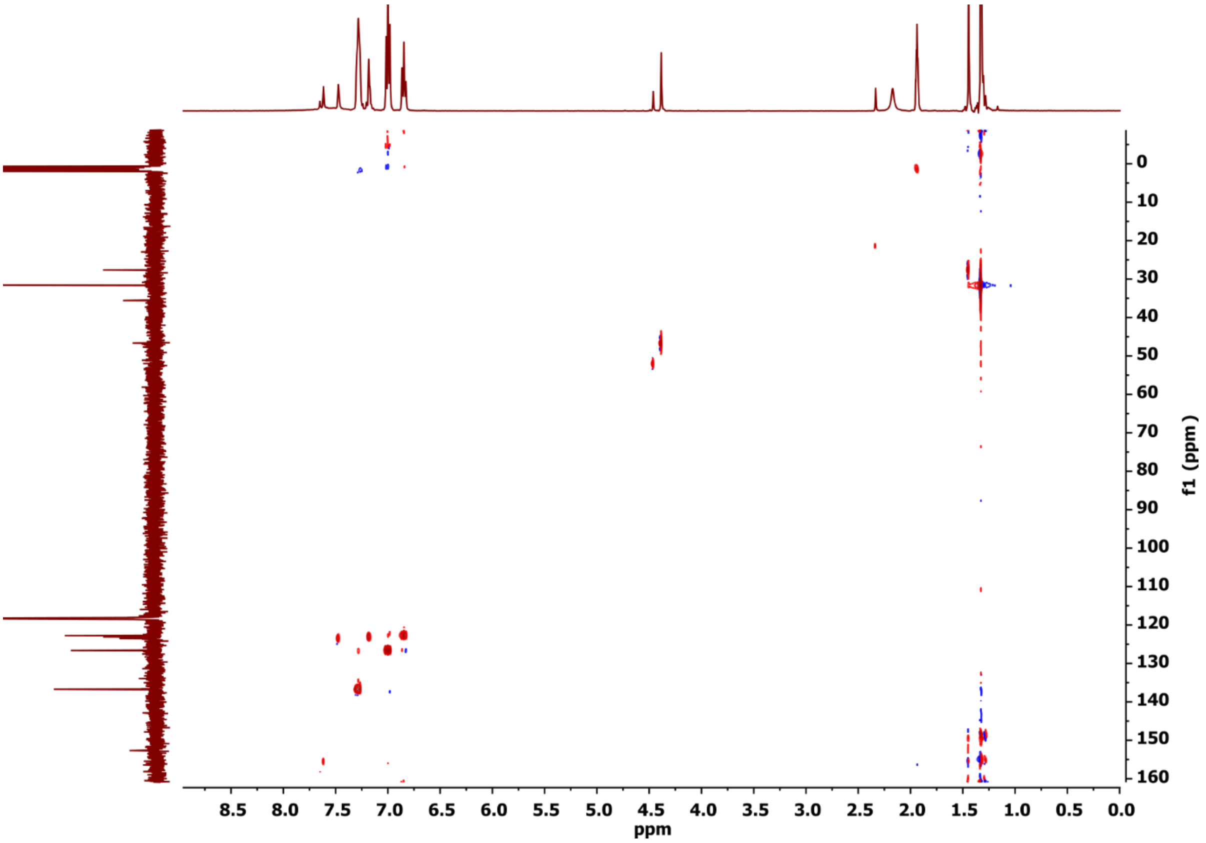

Figure S98. HSQC spectrum of **3a** (400 & 101 MHz, CD_3CN, 296 K).

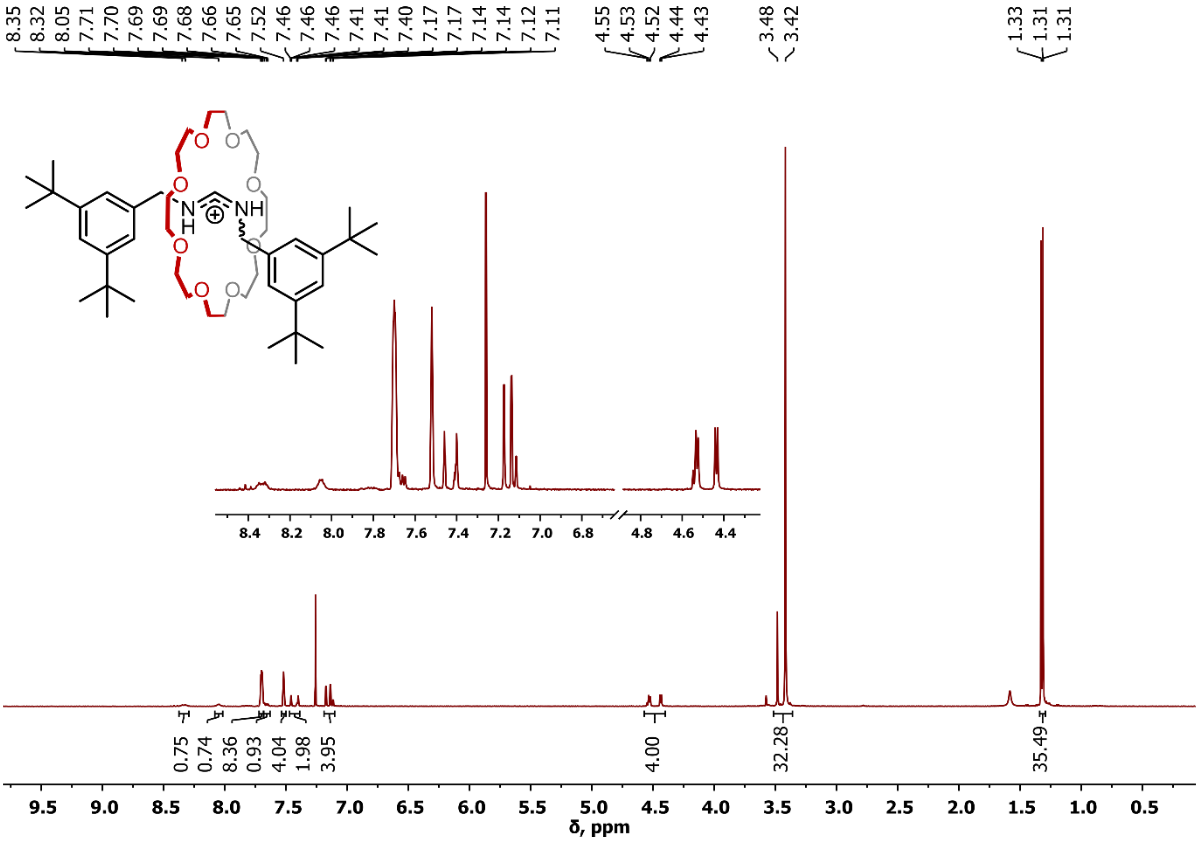

Figure S99. ^{1}H NMR spectrum of **1a** (500 MHz, $CDCl_3$, 300 K).

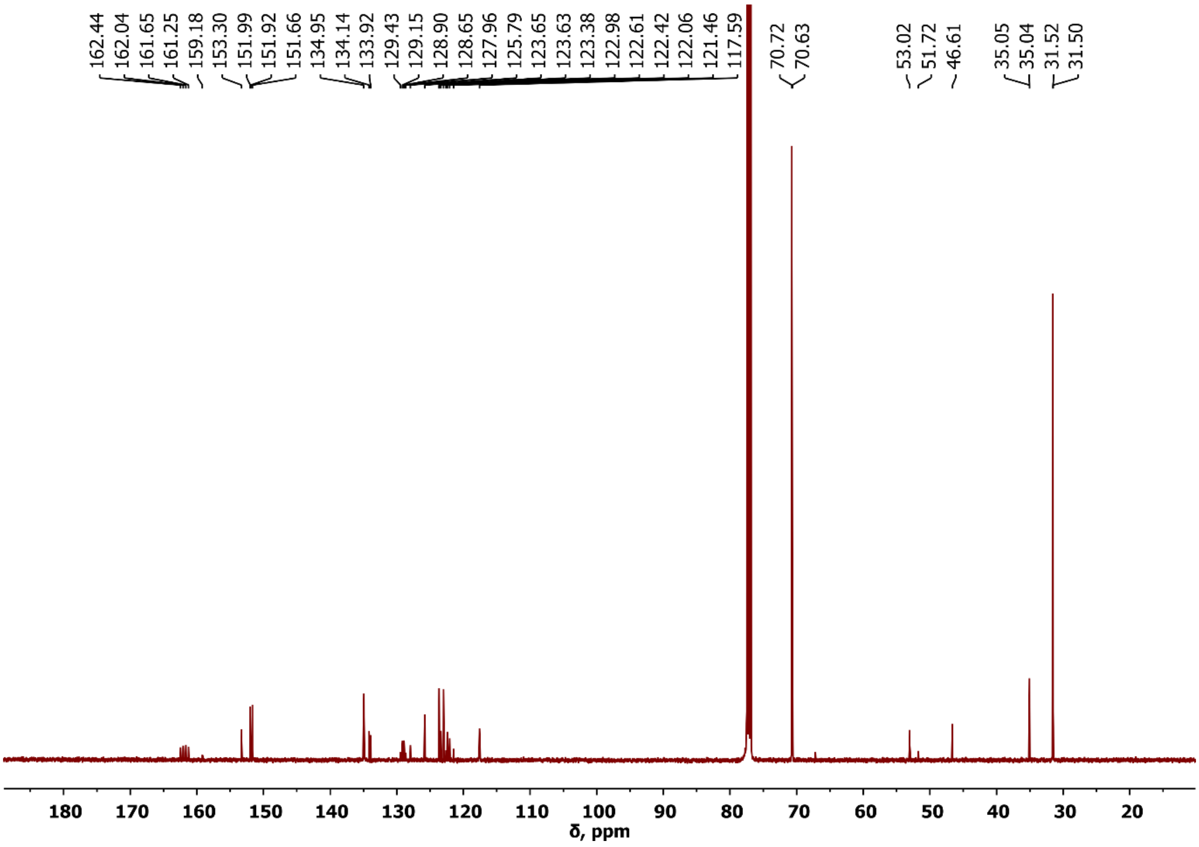

Figure S100. ^{13}C NMR spectrum of **1a** (126 MHz, $CDCl_3$, 300 K).

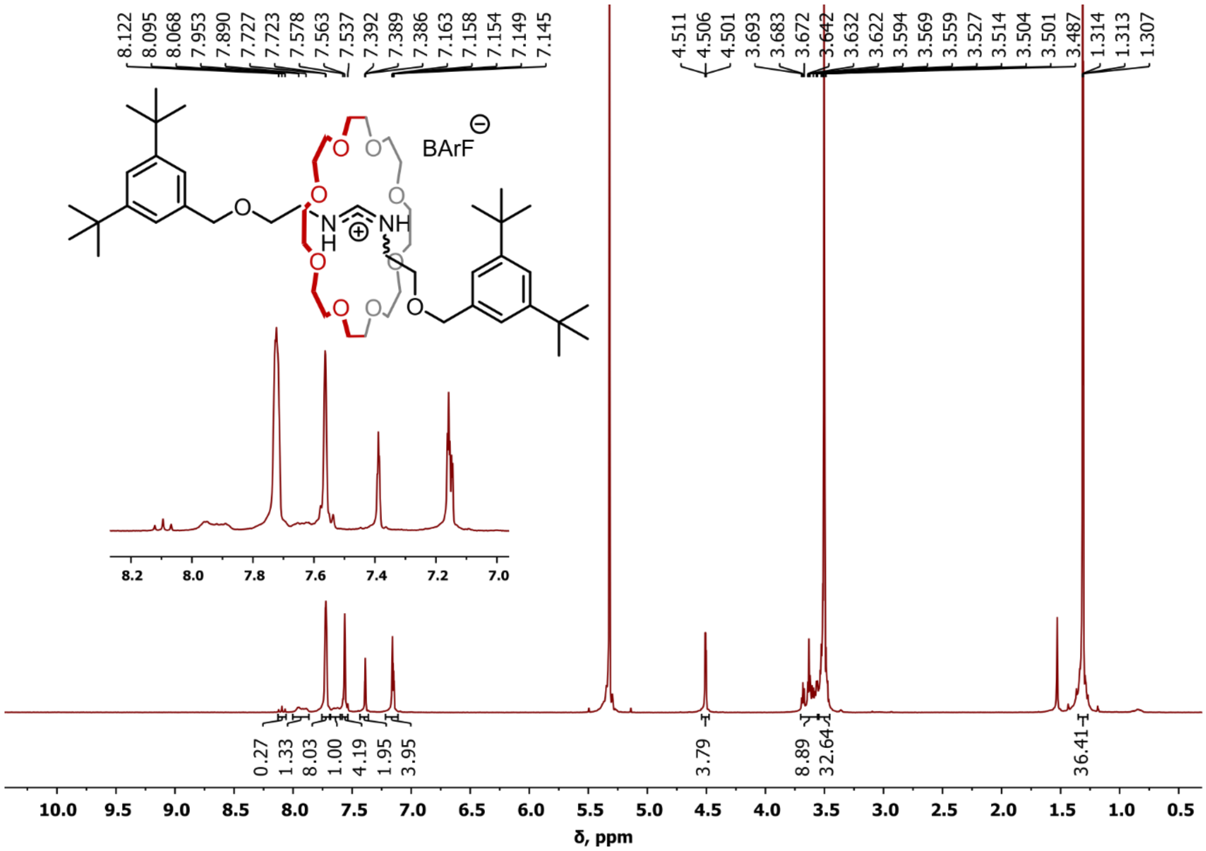

Figure S101. ^{1}H NMR spectrum of **1b** (500 MHz, CD_2Cl_2, 300 K).

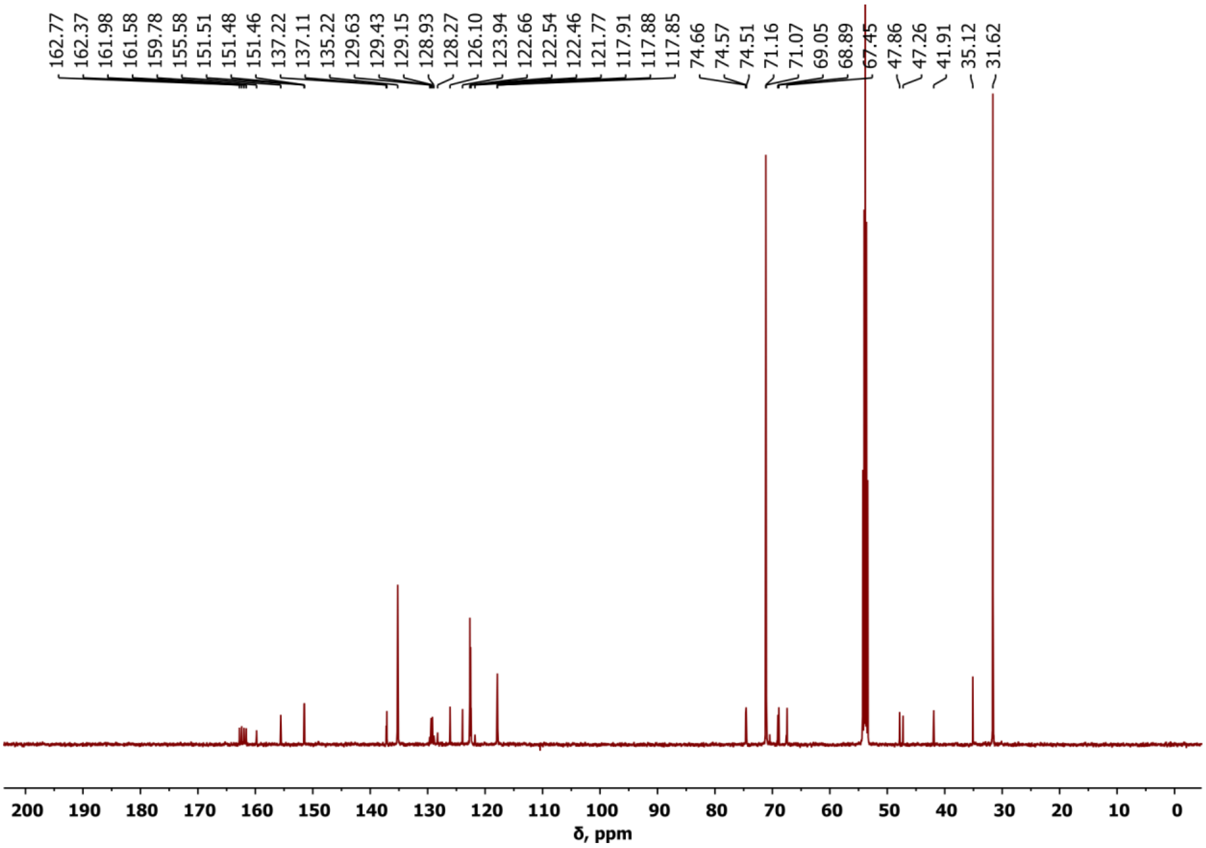

Figure S102. ^{13}C NMR spectrum of **1b** (126 MHz, $CDCl_3$, 300 K).

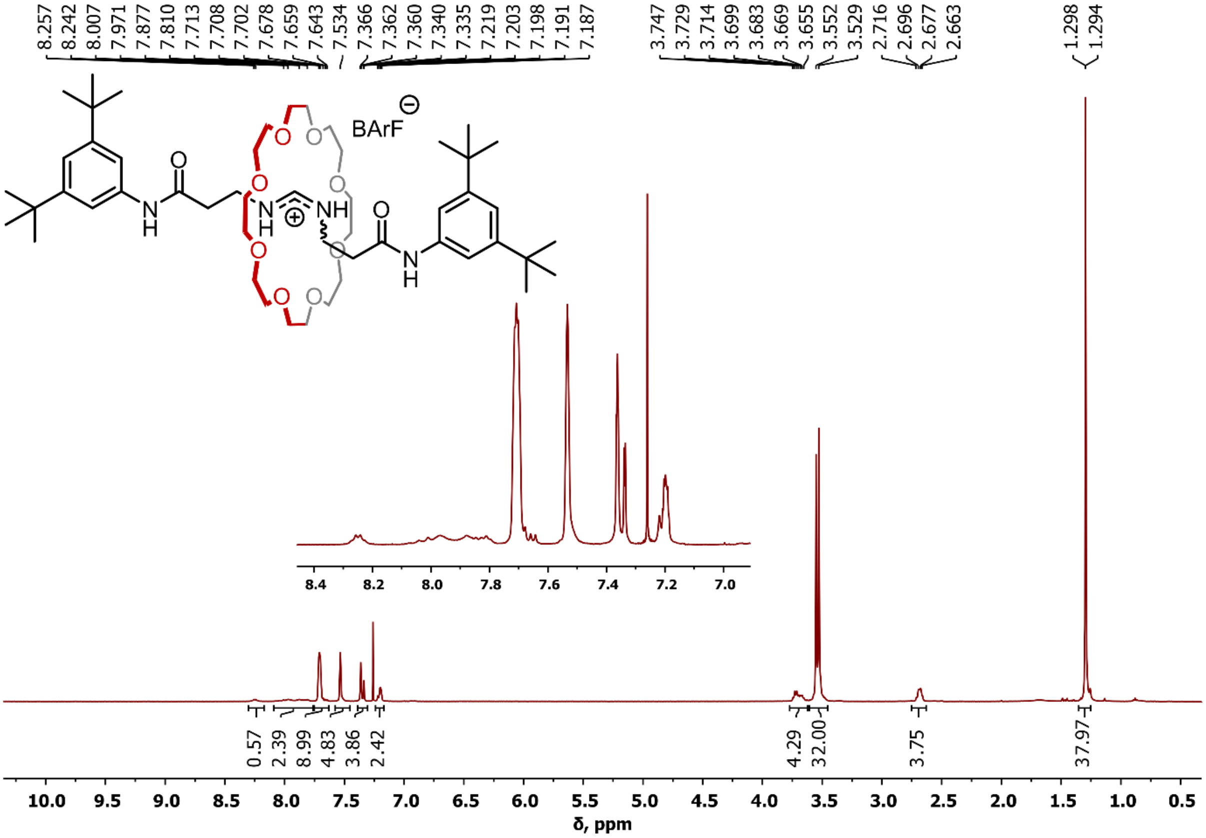

Figure S103. ^{1}H NMR spectrum of **1c** (400 MHz, $CDCl_3$, 295 K).

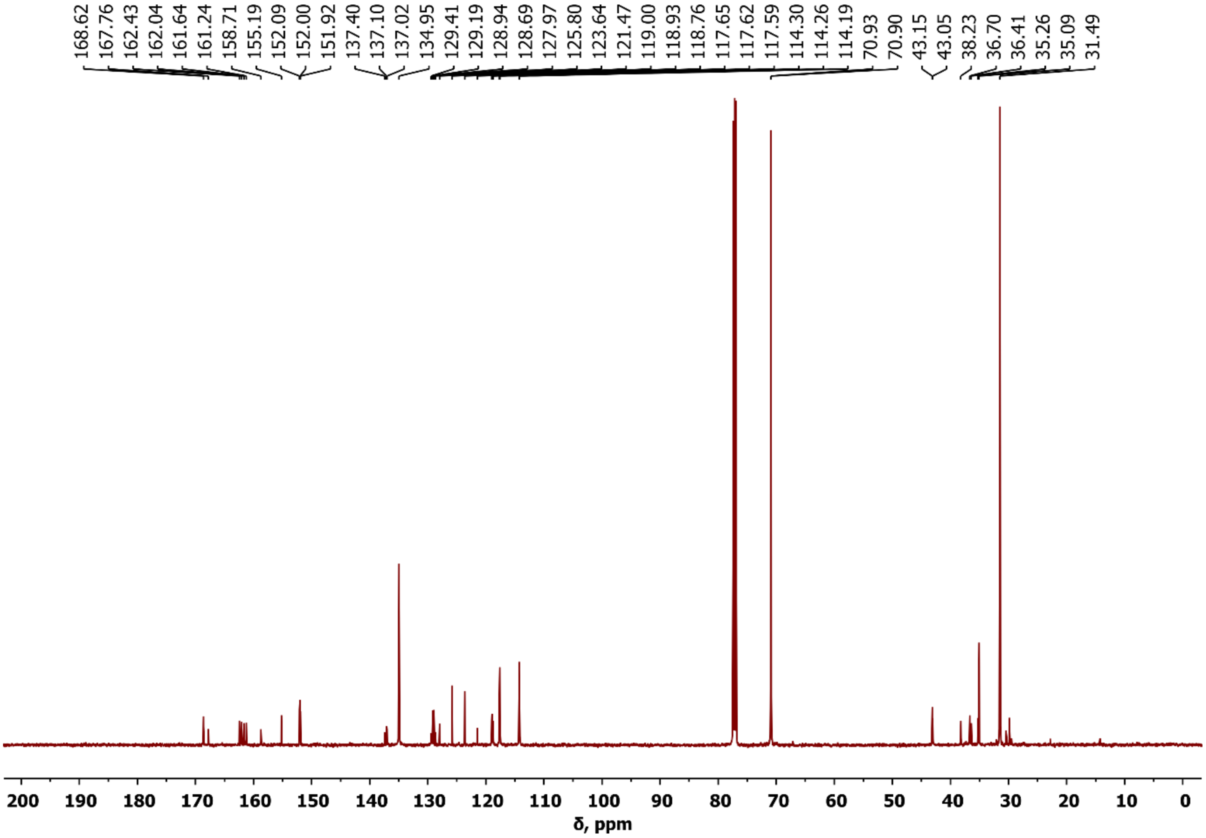

Figure S104. ^{13}C NMR spectrum of **1c** (126 MHz, $CDCl_3$, 300 K).

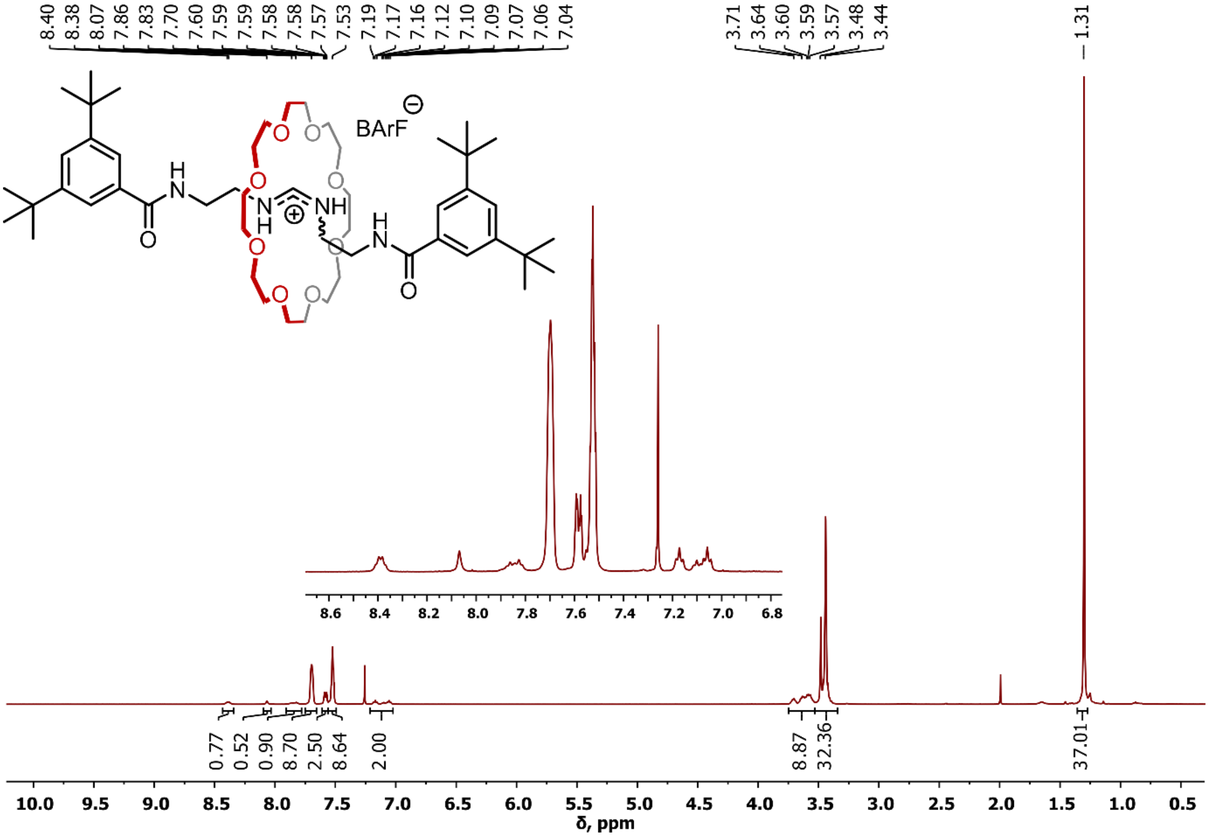

Figure S105. ^{1}H NMR spectrum of **1d** (500 MHz, $CDCl_3$, 300 K).

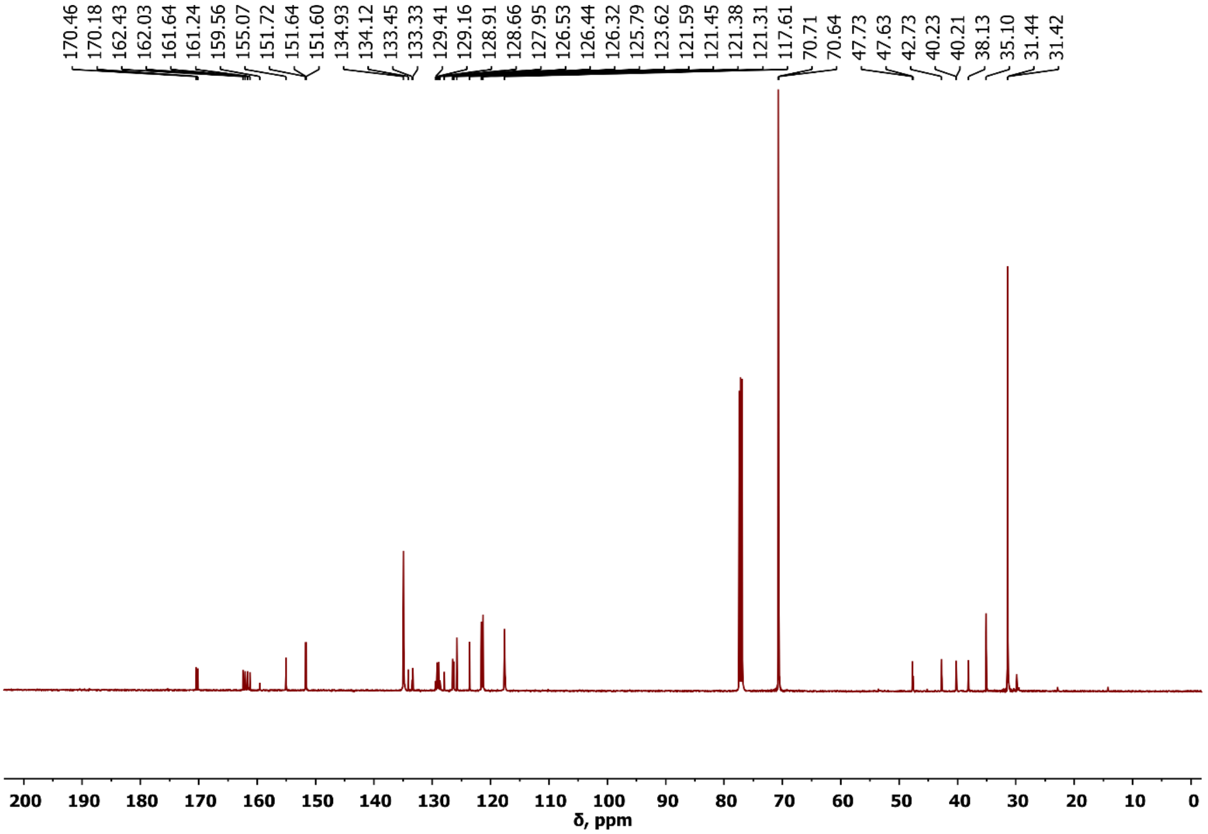

Figure S106. ^{13}C NMR spectrum of **1d** (126 MHz, $CDCl_3$, 300 K).

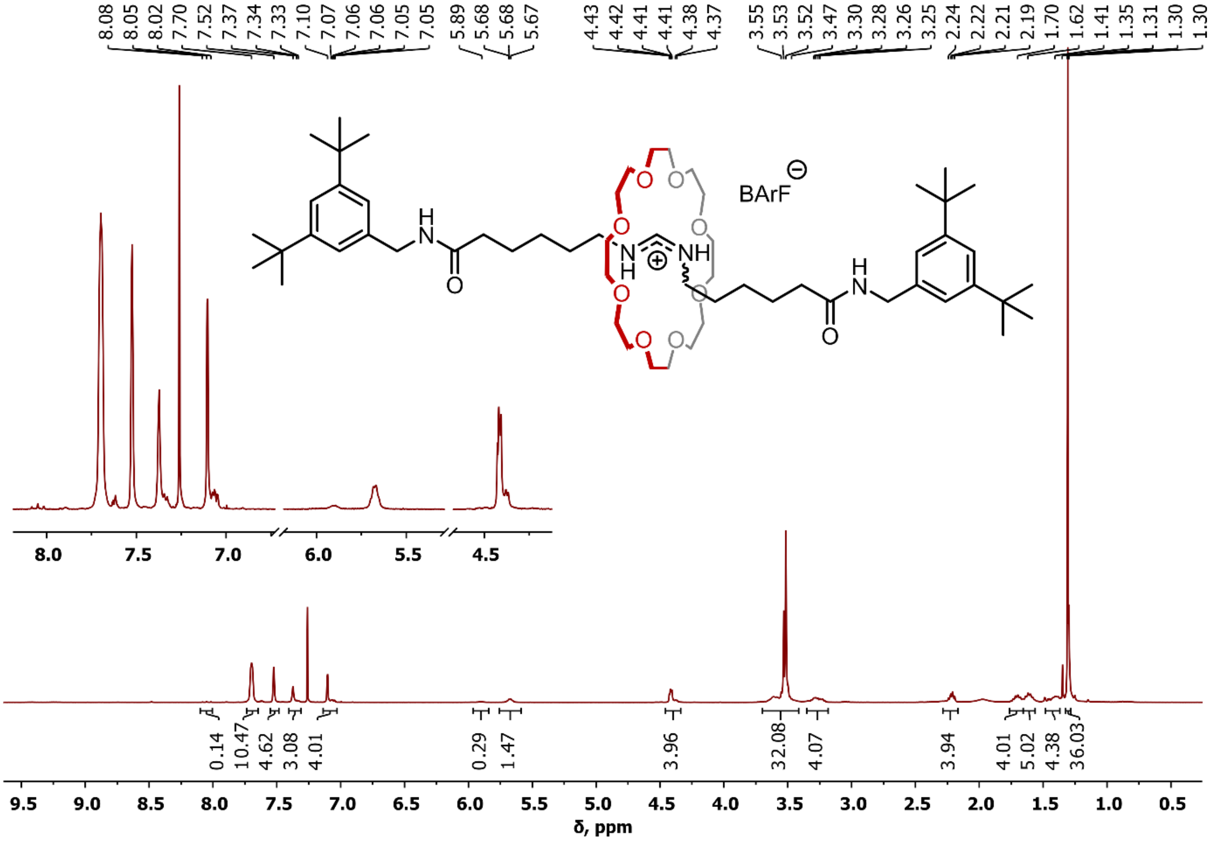

Figure S107. ^{1}H NMR spectrum of **1e** (400 MHz, $CDCl_3$, 295 K).

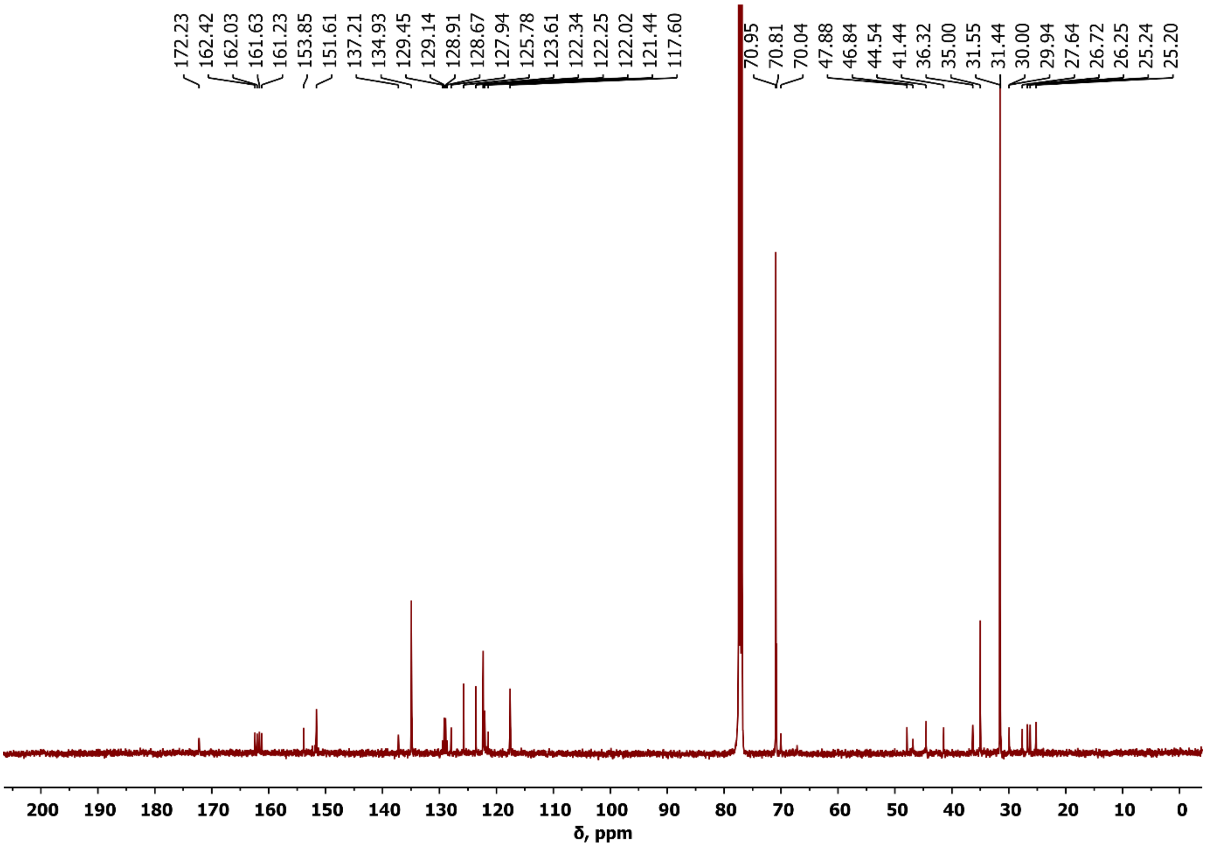

Figure S108. ^{13}C NMR spectrum of **1e** (126 MHz, $CDCl_3$, 300 K).

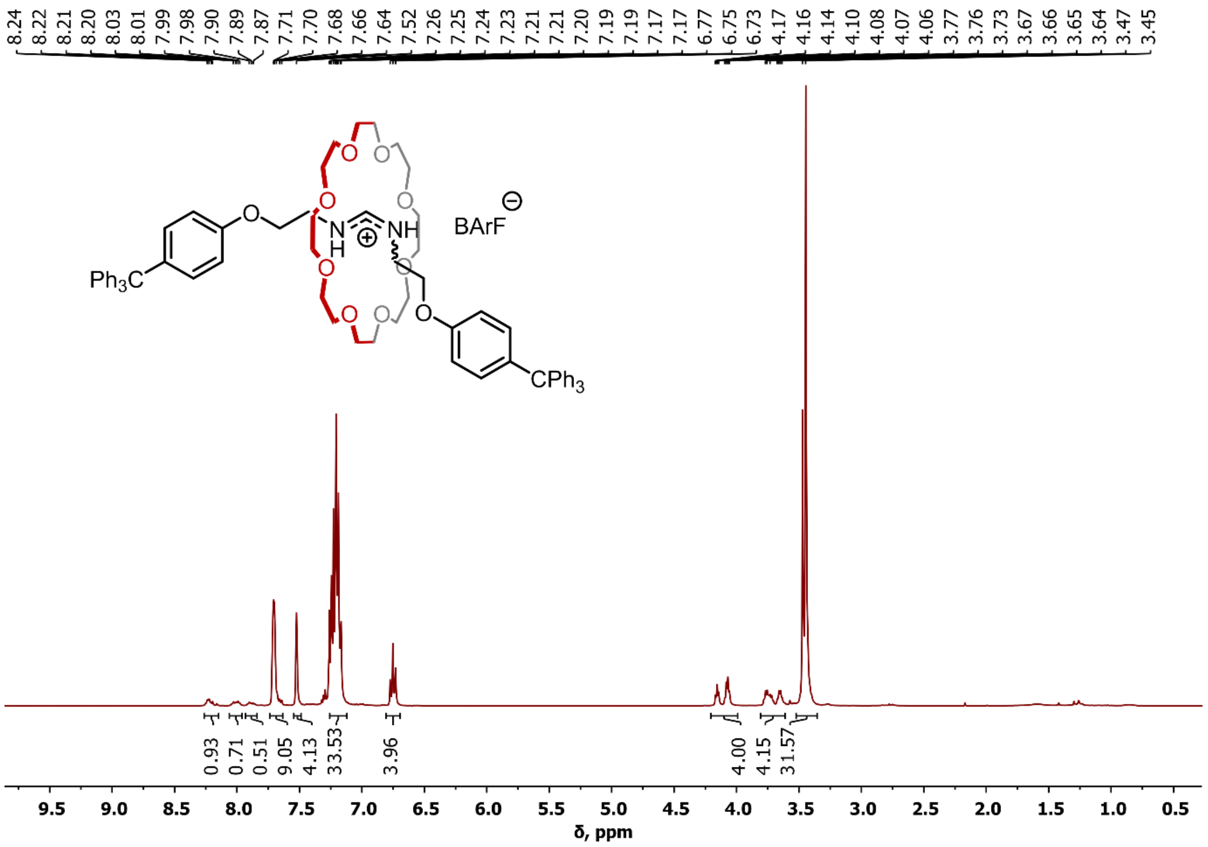

Figure S109. ^{1}H NMR spectrum of **1f** (400 MHz, $CDCl_3$, 295 K).

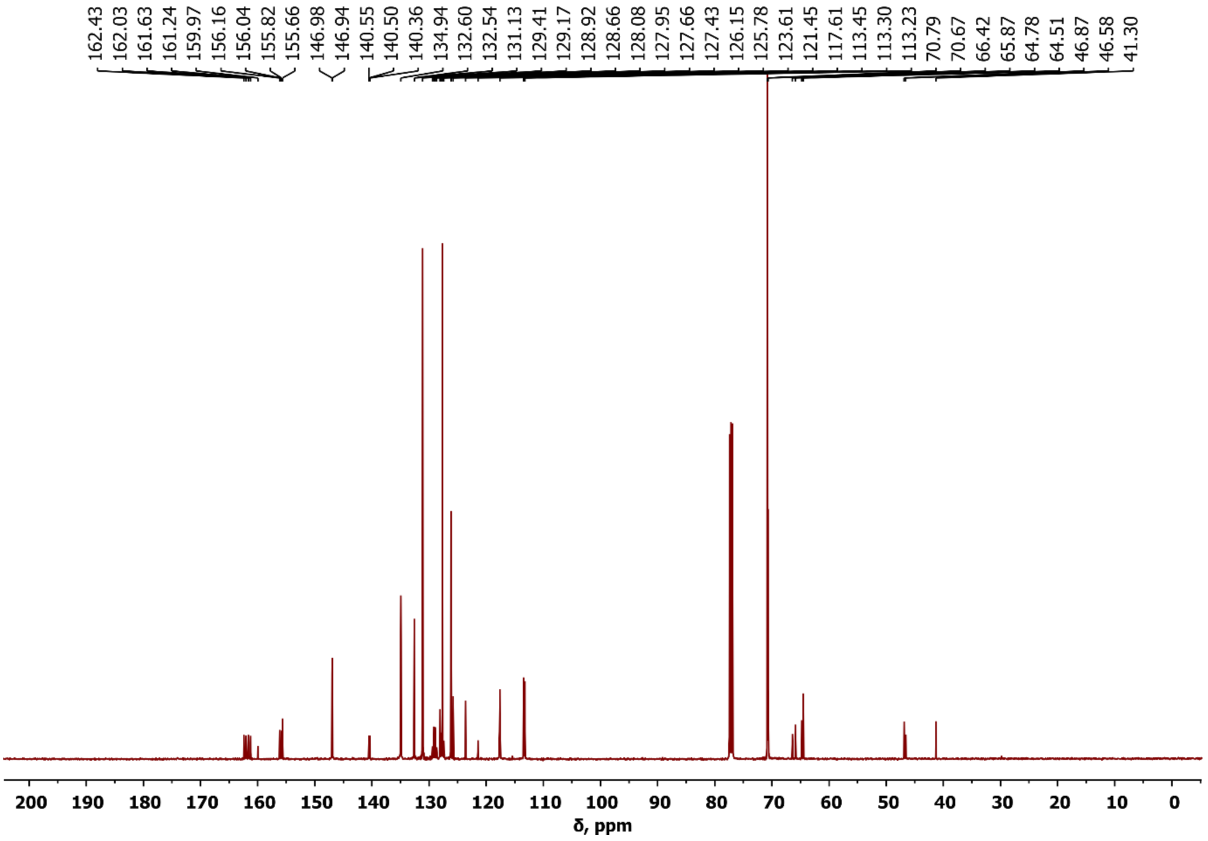

Figure S110. ^{13}C NMR spectrum of **1f** (126 MHz, $CDCl_3$, 300 K).

11. X-ray crystallographic data

Crystallographic data for thread **2a**, rotaxane **1a** and complex **FA⊂24C8** was collected at 150–158 K using Agilent SuperNova four-circle diffractometer equipped with Atlas CCD detector. The structures were solved using SHELXT[27] 2018/2 and refined by the full matrix least-squares based of F^2 using SHELXL[28] 2018/3 accessed via the Olex2 program[29] (v1.3.0). Non-hydrogen atoms were refined anisotropically. Hydrogen atoms were placed in calculated positions with idealized geometries and then refined by employing a riding model and isotropic displacement parameters.

Thread 2a (anion: BPh_4^-)

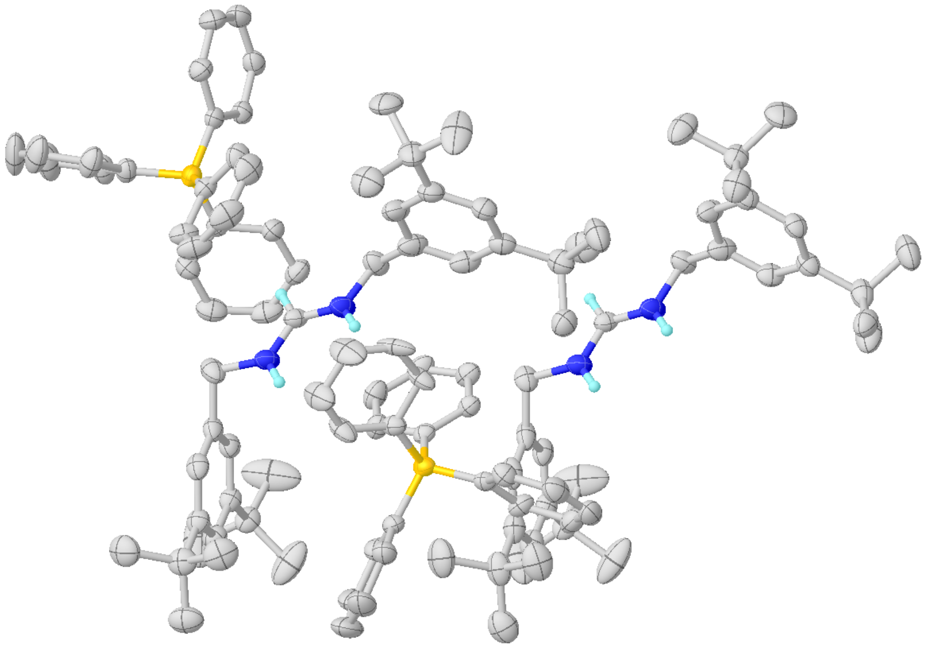

Figure S111. Solid-state structure of thread **2a**. Thermal ellipsoids are shown at the 50% probability level. Disordered parts and most of the hydrogen atoms are omitted for clarity. The single crystal of **2a** was obtained from PhMe/MeCN (9:1 v/v) upon cooling. The cif-file was deposited in the Cambridge structural database under identifier CCDC 2076127.

Comments on disorder

<u>*Amidinium moiety.*</u> Initial refinement afforded the structure with *E*,*E* configuration of the amidinium moiety. However, both amidinium molecules in the asymmetric unit still had residual electron density of relatively high intensity (1.5 – 2.0). Therefore, we made an assumption that another amidinium isomer (*E*,*Z* configuration) was also present in the crystal structure. *E*,*Z* isomer (i.e. the amidinium moiety and adjacent benzylic carbon atoms) was then successfully modelled and refined with partial electron densities. Occupancies of all disordered atoms in each amidinium molecule were linked and chemically equivalent bonds were refined using SADI restraints. In the final structure, occupancies of the disordered atoms in the amidinium moieties were equal to 0.75 (*E*,*E* isomer) and 0.25 (*E*,*Z* isomer).
<u>*tert-Butyl groups.*</u> *tert*-Butyl groups have elongated ellipsoids of carbon atoms that indicates minor (and rather typical) disorder. For one *tert*-butyl group, the disorder was modelled over two positions with using SADI restraints.
<u>*BPh_4^- anion.*</u> One of the phenyl rings was disordered and has been modelled over two positions.
<u>*Solvent.*</u> Molecules of toluene that crystallized together with **2a** were identified during the refinement, however could not be satisfactorily modeled due to high disorder. To account for the electron density, the

Olex2 implementation of BYPASS[30] was used to apply a solvent mask. It was determined that within the unit cell there are 4 solvent voids, each of 497.6 Å^3 and 101.5 electrons (totaling 406 electrons per unit cell). This is consistent with each void being occupied by 2 molecules of toluene (100 electrons) giving a total of 8 molecules of toluene within the unit cell (400 electrons).

Table S24. Crystal data and structure refinement for thread **2a**.

Empirical formula	$C_{55}H_{69}BN_2$
Formula weight	768.93
Temperature/K	150.0
Crystal system	monoclinic
Space group	$P2_1/n$
a/Å	19.15240(10)
b/Å	29.5595(2)
c/Å	19.66730(10)
α/°	90
β/°	108.3300(10)
γ/°	90
Volume/Å^3	10569.41(12)
Z	8
ρ_{calc}g/cm^3	0.966
μ/mm^{-1}	0.407
F(000)	3344.0
Crystal size/mm^3	0.351 × 0.226 × 0.204
Radiation	CuKα (λ = 1.54184)
2Θ range for data collection/°	5.598 to 148.388
Index ranges	$-23 \le h \le 21$, $-35 \le k \le 36$, $-19 \le l \le 24$
Reflections collected	77184
Independent reflections	21156 [R_{int} = 0.0292, R_{sigma} = 0.0239]
Data/restraints/parameters	21156/1108/1228
Goodness-of-fit on F^2	1.055
Final R indexes [I>=2σ (I)]	R_1 = 0.0558, wR_2 = 0.1554
Final R indexes [all data]	R_1 = 0.0666, wR_2 = 0.1662
Largest diff. peak/hole / e Å^{-3}	0.57/-0.35

Rotaxane 1a (anion: $BArF^-$)

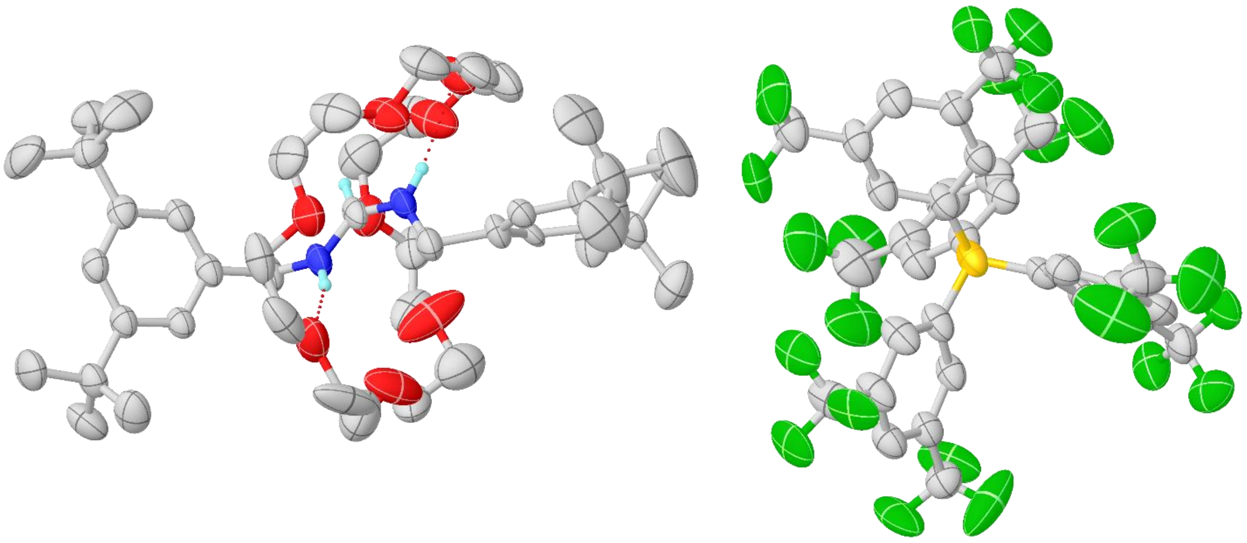

Figure S112. Solid-state structure of rotaxane **1a**. Thermal ellipsoids are shown at the 50% probability level. Disordered parts and most of the hydrogen atoms are omitted for clarity. The single crystal of **1a** was obtained by slow evaporation of a saturated rotaxane solution in n-hexane/$CHCl_3$. The cif-file was deposited in the Cambridge structural database under identifier CCDC 2076129.

Comments on disorder

Thread. One of the phenyl rings and *tert*-butyl groups on another ring were disordered and were modelled over two positions using SADI restraints.

BArF anion. CF_3 groups were highly disordered and were thus refined with independent occupancies to account for each individual CF_3 group having different positional preferences. SADI and DFIX restraints were used.

Crown ether. Some of the ellipsoids (corresponding to both oxygen and carbon atoms) are larger than others, which indicates their larger average position. However, modeling disorder for these atoms did not improve the model.

Solvent. Molecules of *n*-hexane that crystallized together with **1a** were identified during the refinement, however could not be modeled due to their high disorder. To account for the electron density, the Olex2 implementation of BYPASS[30] was used to apply a solvent mask. It was determined that within the unit cell there are 2 solvent voids, each of 199.1 $Å^3$ and 48.9 electrons (totaling 97.8 electrons per unit cell). This is in good agreement with each void being occupied by 1 molecule of *n*-hexane (50 electrons) giving a total of 2 molecules of *n*-hexane within the unit cell (100 electrons).

Comments on the hydrogen bonds

N–H···O hydrogen bonds in **1a** (H···O bond lengths 2.04 Å & 2.20 Å) are on average shorter than N–H···O hydrogen bonds in similar urea- and carbamate-based rotaxanes (H···O bond lengths 2.12 Å & 2.65 Å and 2.65 Å respectively),[20] yet very close in length to N–H···O hydrogen bonds in similar amide-based rotaxanes (2.12 Å or 2.20 Å).[20] Interestingly, in an analogue of **1a** where the amidinium moiety is replaced by a secondary ammonium (NH_2^+ group), N–H···O hydrogen bonds are shorter (1.88 Å & 2.00 Å).[18b] These observations probably reflect the diffuse nature of the positive charge in the amidinium moiety.

Table S25. Crystal data and structure refinement for rotaxane **1a**.

Empirical formula	$C_{79}H_{93}BF_{24}N_2O_8$
Formula weight	1665.36
Temperature/K	158(13)
Crystal system	monoclinic
Space group	$P2_1/c$
a/Å	23.1320(6)
b/Å	17.7563(4)
c/Å	22.3249(5)
α/°	90
β/°	110.257(3)
γ/°	90
Volume/Å^3	8602.5(4)
Z	4
ρ_{calc} g/cm^3	1.286
μ/mm^{-1}	1.020
F(000)	3464.0
Crystal size/mm^3	0.57 × 0.153 × 0.096
Radiation	Cu Kα (λ = 1.54184)
2Θ range for data collection/°	6.876 to 146.412
Index ranges	-28 ≤ h ≤ 28, -15 ≤ k ≤ 21, -27 ≤ l ≤ 27
Reflections collected	57058
Independent reflections	16885 [R_{int} = 0.0801, R_{sigma} = 0.0605]
Data/restraints/parameters	16885/1391/1429
Goodness-of-fit on F^2	1.071
Final R indexes [I>=2σ (I)]	R_1 = 0.1035, wR_2 = 0.2844
Final R indexes [all data]	R_1 = 0.1520, wR_2 = 0.3324
Largest diff. peak/hole / e Å^{-3}	0.60/-0.49

FA⊂24C8 (anion: BPh_4^-)

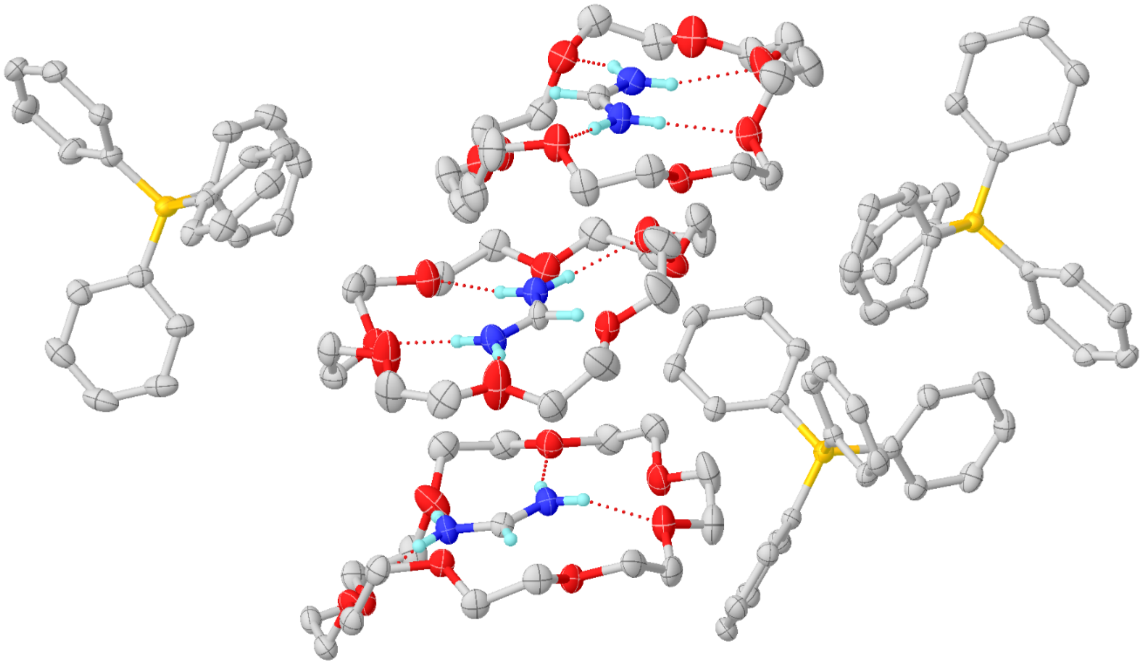

Figure S113. Solid-state structure of hydrogen bonded complex **FA⊂24C8**. Thermal ellipsoids are shown at the 50% probability level. Disordered parts and most of the hydrogen atoms are omitted for clarity. The single crystal of **FA⊂24C8** was obtained from THF upon slow evaporation. The cif-file was deposited in the Cambridge structural database under identifier CCDC 2076128.

Comments on disorder

Crown ether and formamidinium. In the asymmetric unit, the central crown ether ring and formamidinium ion were highly disordered. Therefore, the whole amidinium ion as well as some carbon and oxygen atoms of the crown ether were modelled over two positions with linked partial occupancies for all disordered atoms. For modelling disorder of the central crown ether ring, SADI restraints and EADP constraints were used.

Table S26. Crystal data and structure refinement for **FA⊂24C8**.

Empirical formula	$C_{41}H_{57}BN_2O_8$
Formula weight	716.69
Temperature/K	150
Crystal system	monoclinic
Space group	$P2_1/n$
a/Å	28.6210(3)
b/Å	15.91340(10)
c/Å	28.8528(3)
α/°	90
β/°	117.4030(10)
γ/°	90
Volume/Å^3	11666.7(2)
Z	12
ρ_{calc}g/cm^3	1.224
μ/mm^{-1}	0.673

F(000)	4632.0
Crystal size/mm^3	0.329 × 0.139 × 0.098
Radiation	CuKα (λ = 1.54184)
2Θ range for data collection/°	6.902 to 145.958
Index ranges	-35 ≤ h ≤ 34, -19 ≤ k ≤ 17, -34 ≤ l ≤ 35
Reflections collected	48389
Independent reflections	22624 [R_{int} = 0.0302, R_{sigma} = 0.0331]
Data/restraints/parameters	22624/1166/1502
Goodness-of-fit on F^2	1.029
Final R indexes [I>=2σ (I)]	R_1 = 0.0607, wR_2 = 0.1611
Final R indexes [all data]	R_1 = 0.0715, wR_2 = 0.1733
Largest diff. peak/hole / e Å^{-3}	0.78/-0.34

12. References

(1) Yamawaki, J.; Ando, T., POTASSIUM FLUORIDE ON ALUMINA AS BASE FOR CROWN ETHER SYNTHESIS. *Chem. Lett.* **1980,** *9*, 533-536.

(2) Talanov, V. S.; Bartsch, R. A., Improved Preparation of 24-Crown-8. *Synth. Commun.* **1999,** *29*, 3555-3560.

(3) Yamawaki, J.; Ando, T., POTASSIUM FLUORIDE ON INORGANIC SOLID SUPPORTS. A SEARCH FOR FURTHER EFFICIENT REAGENTS PROMOTING HYDROGEN-BOND-ASSISTED ALKYLATIONS. *Chem. Lett.* **1979,** *8*, 755-758.

(4) Chênevert, R.; D'Astous, L., Synthesis of large ring crown ethers. *J. Heterocycl. Chem.* **1986,** *23*, 1785-1787.

(5) Meade, E. A.; Sznaidman, M.; Pollard, G. T.; Beauchamp, L. M.; Howard, J. L., Anxiolytic activity of analogues of 4-benzylamino-2-methyl-7H-pyrrolo[2,3-d]pyrimidines. *Eur. J. Med. Chem.* **1998,** *33*, 363-374.

(6) Tosatti, P.; Horn, J.; Campbell, A. J.; House, D.; Nelson, A.; Marsden, S. P., Iridium-Catalyzed Asymmetric Allylic Amination with Polar Amines: Access to Building Blocks with Lead-Like Molecular Properties. *Adv. Synth. Catal.* **2010,** *352*, 3153-3157.

(7) Servín, F. A.; Romero, J. A.; Aguirre, G.; Grotjahn, D.; Somanathan, R.; Chávez, D.; Servín, F. A.; Romero, J. A.; Aguirre, G.; Grotjahn, D.; Somanathan, R.; Chávez, D., General Method for Selective Mono-Boc Protection of Diamines and Thereof. *J. Mex. Chem. Soc.* **2017,** *61*, 23-27.

(8) Inman, M.; J. Moody, C., Synthesis of Indolequinones from Bromoquinones and Enamines Mediated by Cu(OAc)2·H2O. *J. Org. Chem.* **2010,** *75*, 6023-6026.

(9) Prabhakaran, E. N. Conformationally Constrained Peptide Mimetics. 2013.

(10) Bianco, A.; Bonadies, F.; Napolitano, R.; Ortaggi, G., SYNTHESIS OF URETHANES BY A MODIFIED CURTIUS REACTION WITH ALCOHOLS. *Org. Prep. Proced. Int.* **2004,** *36*, 141-149.

(11) Bartoszewicz, A.; Kalek, M.; Nilsson, J.; Hiresova, R.; Stawinski, J., A New Reagent System for Efficient Silylation of Alcohols: Silyl Chloride-N-Methylimidazole-Iodine. *Synlett* **2008,** *2008*, 37-40.

(12) J. Kamlet, M.; Luis M. Abboud, J.; H. Abraham, M.; W. Taft, R., Linear solvation energy relationships. 23. A comprehensive collection of the solvatochromic parameters, .pi.*, .alpha., and .beta., and some methods for simplifying the generalized solvatochromic equation. *J. Org. Chem.* **2002,** *48*, 2877-2887.

(13) Common Solvents Used in Organic Chemistry: Table of Properties.

(14) Cataldo, F., A REVISION OF THE GUTMANN DONOR NUMBERS OF A SERIES OF PHOSPHORAMIDES INCLUDING TEPA. *Eur. Chem. Bull.* **2015,** *4*, 92-97.

(15) Reichardt, C., Empirical Parameters of the Polarity of Solvents. *Angew. Chem. Int. Ed.* **1965,** *4*, 29-40.

(16) Adrian, J. C.; Wilcox, C. S., Chemistry of synthetic receptors and functional group arrays. 15. The effects of added water on thermodynamic aspects of hydrogen bond based molecular recognition in chloroform. *J. Am. Chem. Soc.* **1991,** *113*, 678-680.

(17) (a) Brynn Hibbert, D.; Thordarson, P., The death of the Job plot, transparency, open science and online tools, uncertainty estimation methods and other developments in supramolecular chemistry data analysis. *Chem. Commun.* **2016,** *52*, 12792-12805; (b) Thordarson, P., Determining association constants from titration experiments in supramolecular chemistry. *Chem. Soc. Rev.* **2011,** *40*, 1305-1323.

(18) (a) Ashton, P. R.; Bartsch, R. A.; Cantrill, S. J.; Hanes, R. E.; Hickingbottom, S. K.; Lowe, J. N.; Preece, J. A.; Stoddart, J. F.; Talanov, V. S.; Wang, Z.-H., Secondary dibenzylammonium ion binding by [24]crown-8 and [25]crown-8 macrocycles. *Tetrahedron Lett.* **1999,** *40*, 3661-3664; (b) P. Fielden, S. D.; A. Leigh, D.; T. McTernan, C.; Pérez-Saavedra, B.; J. Vitorica-Yrezabal, I., Spontaneous Assembly of Rotaxanes from a Primary Amine, Crown Ether and Electrophile. *J. Am. Chem. Soc.* **2018,** *140*, 6049-6052.

(19) Cotton, F. A.; Haefner, S. C.; Matonic, J. H.; Wang, X.; Murillo, C. A., Structural studies of formamidine compounds : From neutral to anionic and cationic species. *Polyhedron* **1997,** *16*, 541-550.

(20) Tian, C.; Fielden, S. D. P.; Whitehead, G. F. S.; Vitorica-Yrezabal, I. J.; Leigh, D. A., Weak functional group interactions revealed through metal-free active template rotaxane synthesis. *Nat. Commun.* **2020**.

(21) Denis, M.; Goldup, S. M., The active template approach to interlocked molecules. *Nat. Rev. Chem.* **2017,** *1*, 0061.

(22) Capela, M. d.; Mosey, N. J.; Xing, L.; Wang, R.; Petitjean, A., Amine Exchange in Formamidines: An Experimental and Theoretical Study. *Chem. Eur. J.* **2011,** *17*, 4598-4612.

(23) Kalz, K. F.; Hausmann, A.; Dechert, S.; Meyer, S.; John, M.; Meyer, F., Solution Chemistry of N,N′-Disubstituted Amidines: Identification of Isomers and Evidence for Linear Dimer Formation. *Chem. Eur. J.* **2016,** *22*, 18190-18196.

(24) (a) Kosturkiewicz, Z.; Ciszak, E.; Tykarska, E., Configuration of amidines and factors governing their hydrogen-bonding patterns. *Acta Crystallographica Section B* **1992,** *48*, 471-476; (b) Xing, L.; Wiegert, C.; Petitjean, A., Stereochemical and Conformational Exchanges in N,N′-Di(2-pyridyl)formamidines: An X-ray and 1H NMR Study. *J. Org. Chem.* **2009,** *74*, 9513-9516; (c) Pastor, M. B.; Feng, C.; Tsai, Y.-J.; Watson, A.; Zhao, Q., N,N′-diarylformamidines: Solid state structural analysis and steric-induced stereochemical exchange in solution. *Tetrahedron* **2021,** *84*, 131995.

(25) Dacosta, A.; Pekerar, S. V.; Núñez, O., N-Alkyl-N-methylacetamidinium Ions. Isomerization and Water Catalyzed Exchange Rates in D2O. *Molecules* **2000,** *5*, 309-310.
(26) (a) Brachvogel, R.-C.; Maid, H.; Von Delius, M., NMR Studies on Li+, Na+ and K+ Complexes of Orthoester Cryptand o-Me2-1.1.1. *International Journal of Molecular Sciences* **2015,** *16*, 20641-20656; (b) Perrin, C. L.; Dwyer, T. J., Application of two-dimensional NMR to kinetics of chemical exchange. *Chem. Rev.* **1990,** *90*, 935-967.
(27) Sheldrick, G. M., {\it SHELXT} {--} Integrated space-group and crystal-structure determination. *Acta Cryst. A* **2015,** *71*, 3-8.
(28) Sheldrick, G. M., Crystal structure refinement with {\it SHELXL}. *Acta Cryst. C* **2015,** *71*, 3-8.
(29) Dolomanov, O. V.; Bourhis, L. J.; Gildea, R. J.; Howard, J. A. K.; Puschmann, H., OLEX2: a complete structure solution, refinement and analysis program. *J. Appl. Crystallogr.* **2009,** *42*, 339-341.
(30) van der Sluis, P.; Spek, A. L., BYPASS: an effective method for the refinement of crystal structures containing disordered solvent regions. *Acta Cryst. A* **1990,** *46*, 194-201.
